# Sequential one-pot *N*-alkylation and aminocarbonylation of primary amines catalyzed by heterobimetallic Ir/Pd complexes

**DOI:** 10.1039/d5sc03892h

**Published:** 2025-09-15

**Authors:** Amin Abdolrahimi, Philipp Woite, Konrad Kretschmar, Michael Roemelt, Thomas Braun, Ouchan He

**Affiliations:** a Department of Chemistry, Humboldt-Universität zu Berlin Brook-Taylor Str. 2 12489 Berlin Germany thomas.braun@cms.hu-berlin.de

## Abstract

The paper introduces bimetallic Ir/Pd complexes as catalysts to initiate an *N*-methylation coupled with an aminocarbonylation in a one-pot approach, in order to advance the field of carboxyamide synthesis. The iridium center initiates the reaction by selectively facilitating the *N*-methylation through amination, while the palladium center, in a complementary role, drives the carbonylation step. This bimetallic synergy not only streamlines the reaction sequence but also surpasses the efficiency and selectivity of monometallic Ir or Pd catalysts. Mechanistic studies suggest the presence of catalytically active hydride species within the *N*-methylation cycle, which were characterized experimentally and *via* quantum chemical calculations. The developed synthetic routes offer a sustainable, cost-effective, scalable and also unprecedented preparation method, with the bimetallic catalysts being robust and versatile.

## Abstract 2

Heterobimetallic Ir/Pd complexes were synthesized to initiate a sequential *N*-methylation followed by an aminocarbonylation in a one-pot approach. Mechanistic studies indicate hydride species as intermediates in the methylation cycle.

## Graphical abstract

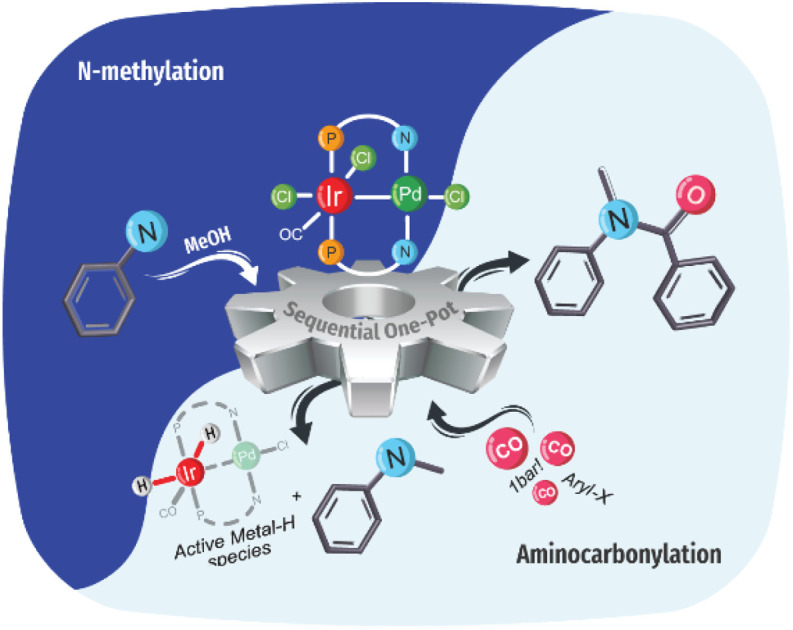

## Introduction

Sequential one-pot catalysis involves multiple catalytic steps in a set sequence, allowing intermediates from one step to serve as substrates for the next without isolation or purification. This method enhances efficiency in organic synthesis by linking different reactions in one-pot, reducing solvents, catalysts, workup procedures, and reaction times.^1–4^ However, catalysts must be compatible with both reaction steps with respect to reaction conditions and lifetimes.^1–9^

Bimetallic catalysts, particularly heterobimetallic ones, are promising for sequential catalysis, where two different metal centers synergistically catalyze multiple reaction steps. This can led to improved or unique reactivities and selectivities compared to homobimetallic or monometallic catalysts.^10–32^ While processes enhancing the activity of heterobimetallic complexes compared to their monometallic counterparts are well-established, the one-pot activity for sequential reaction pathways remains limited.^33–38^ An array of late–late bimetallic transition metal complexes has been synthesized to serve as unique catalysts in diverse C–N/C–C bond coupling reactions.^39–43^ Notably, bimetallic Pd(i) and Pd(ii) complexes exhibit activity in aminocarbonylation of primary amines and Buchwald–Hartwig aminations.^44^ A heterobimetallic catalyst featuring Ir/Pd centers and a 1,2,4-trimethyltriazolyldiylidene ligand (ditz) was employed to catalyze tandem imine formation from corresponding nitroarenes.^45^ Furthermore, three sets of Ir/Pd complexes with the mentioned bridging ligand were synthesized and utilized in tandem reactions involving various transformations of halo-acetophenones. These transformations encompass (i) dehalogenation and transfer hydrogenation, (ii) Suzuki coupling and transfer hydrogenation, and (iii) Suzuki coupling and α-alkylation with primary alcohols.^46^

**Fig. 1:**
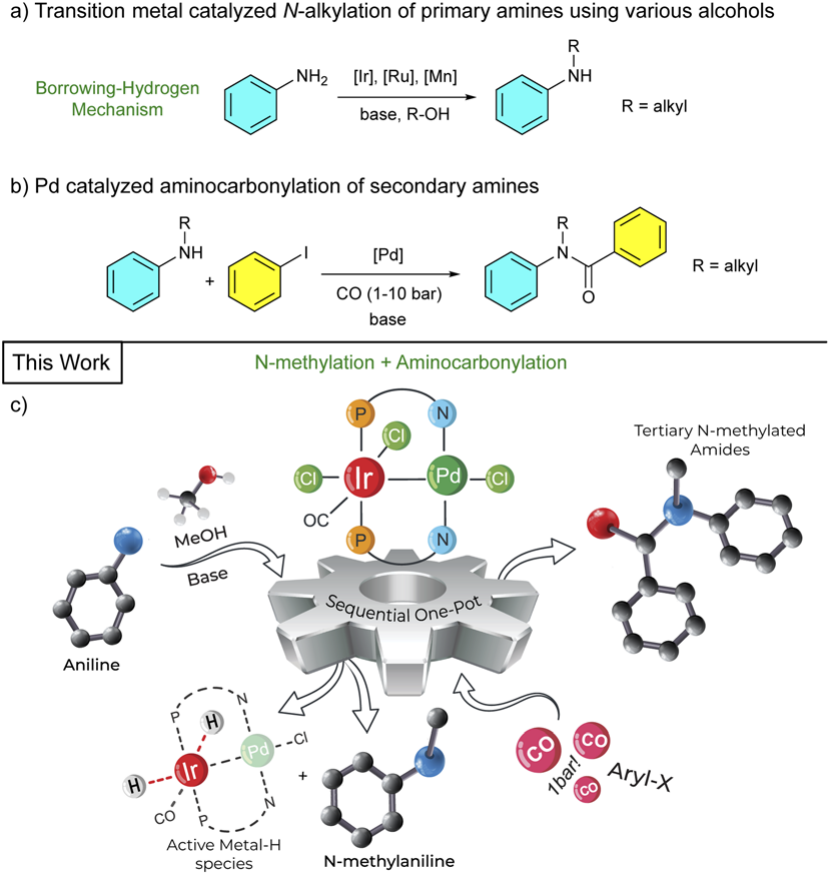
(a) Transition metal-catalyzed *N*-alkylation. (b) Pd-catalyzed aminocarbonylation. (c) Sequential one-pot *N*-alkylation followed by aminocarbonylation of primary amines.

In this paper we report on the synthesis of Ir/Pd bimetallic complexes and their application in a one-pot process for mono-*N*-methylations of primary amines under air, coupled with aminocarbonylations of the resulting secondary amine under atmospheric CO pressure. Model studies gave mechanistic insights by identifying catalytically relevant hydride species, which were also characterized by means of quantum chemical calculations (Fig. 1).

**Scheme 1:**
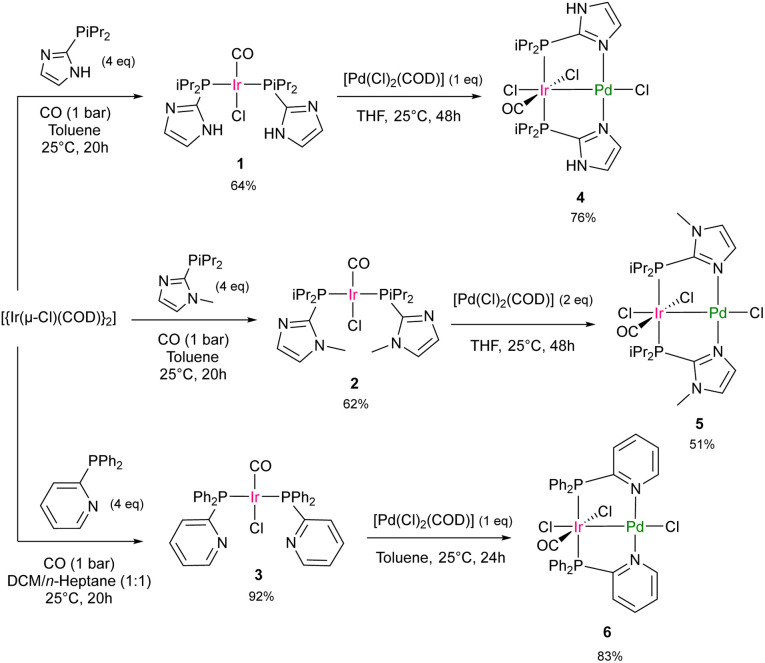
Routes to the complexes 4, 5, 3 and 6.^47^

Routes to bimetallic Ir/Pd complexes bearing imidazole or pyridine based bridging phosphine ligands were developed. The bimetallic compounds [IrPd(Cl)_3_(CO)(P*i*Pr_2_Im)_2_] (Im = imidazole) (4), [IrPd(Cl)_3_(CO)(P*i*Pr_2_Im^Me^)_2_] (Im^Me^ = 1-methyl-1*H*-imidazole) (5) and [IrPd(Cl)_3_(CO)(PPh_2_Py)_2_] (Py = pyridine) (6) were synthesized by treatment of the corresponding mononuclear Ir complexes *trans*-[Ir(Cl)(CO)(P*i*Pr_2_Im)_2_] (1), *trans-*[Ir(Cl)(CO)(P*i*Pr_2_Im^Me^)_2_] (2) and *trans*-[Ir(Cl)(CO)(PPh_2_Py)_2_] (3) with [Pd(Cl)_2_(COD)] (Scheme 1). The complexes 3 and 6 (Scheme 1) have been reported before, but 6 was synthesized *via* an alternative route.^47^ Other binuclear complexes featuring 2-(diphenylphosphino)pyridine (Ph_2_PPy) as a bridging ligand with iridium centers have also been described.^49–51^ The bimetallic Ir/Pd complexes were characterized by ^1^H NMR, ^31^P{^1^H} NMR, and IR spectroscopy (see SI). Notably, the IR spectrum of 4 displays a very broad absorption band at *ν̃* = 3091 cm^−1^ for the NH unit, which is shifted to lower frequencies compared to the NH absorption frequency of 1 at 3343 cm^−1^. The NMR and IR data suggest the presence of hydrogen bonding.^52,53^ Strong absorption bands for the terminal-bound CO were detected between 1950 and 2016 cm^−1^ for all three complexes 4–6 in the ATR-IR spectra (4 (*ν̃* = 2000 cm^−1^), 5 (*ν̃* = 1950 cm^−1^) and 6 (*ν̃* = 2013 cm^−1^).^47^Table 1 shows these IR frequencies together with their corresponding ^31^P NMR data.

**Table 1:** ^31^P NMR data and CO stretching frequencies for complexes 1–6

Compound	CO stretching frequencies (*ν̃*)	^31^P NMR chemical shift (ppm)
1	1944	28.9
2	1942	25.9
3	1971	24.4
4	2000	−0.76
5	1950	10.54
6	2013	−12.50

**Fig. 2:**
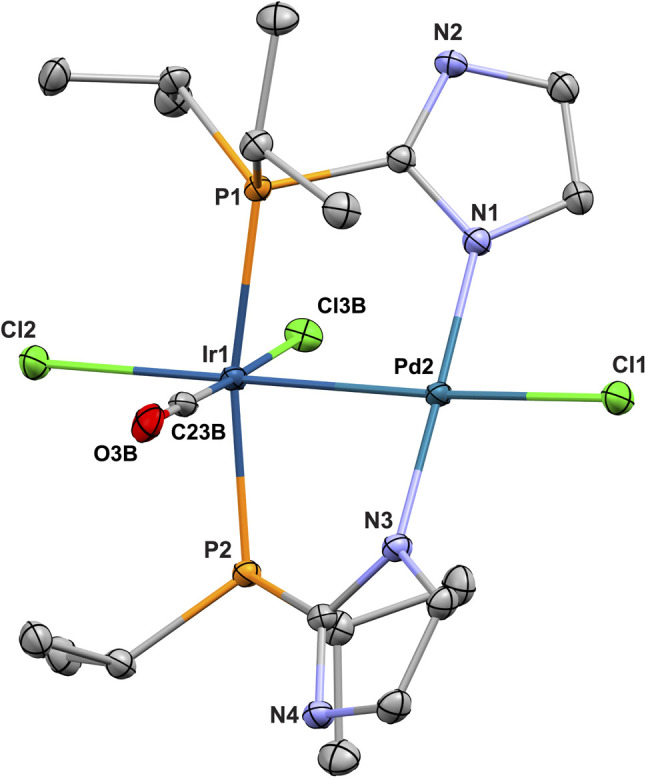
Structure in the solid state of [IrPd(Cl)_3_(CO)(P*i*Pr_2_Imd)_2_] (4)·2 DMSO. DMSO molecules and hydrogen atoms have been omitted for clarity. Selected bond lengths [Å] and bond angles [°]: Ir1–Pd2 2.6259(2), Ir1–Cl2 2.5223(5), Ir1–Cl3B 2.308(5), Ir1–C23B 1.884(14), Ir1–P2 2.3473(5), Ir1–P1 2.3470(5), Pd2–Cl1 2.4098(5), Pd2–N1 2.0349(16), Pd2–N3 2.0247(16), C23B–O3B 1.20(2), P2–Ir1–P1 167.616(17), Cl2–Ir1–Pd2 178.509(14), Cl1–Pd2–Ir1 173.004(15), N3–Pd2–N1 178.00(6), C23B–Ir1–Cl3B 175.4(5). Torsion angles [°]: N1–Pd2–Ir1–P1 −36.86(5), P2–Ir1–Pd2–N3 −34.19(5).

The structure of the complex [IrPd(Cl)_3_(CO)(P*i*Pr_2_Im)_2_] (4) in the solid state was determined by X-ray crystal structure analysis (Fig. 2).

The distance between Ir and Pd (2.6259(4) Å) is in the typical range of Ir–Pd metal bonds of similar complexes, for instance 2.614(1) Å for 6 (ref. 47) and 2.694(2) Å for [IrPd(Cl)(CO)(dpmp)_2_][PF_6_]_2_ (ref. 48) (dpmp: bis-((diphenylphosphino)methyl)-phenylphosphine). The amine functions (NH) of the two imidazolylphosphines each form a hydrogen bond to two DMSO solvent molecules in the asymmetric unit.^52,53^

**Fig. 3:**
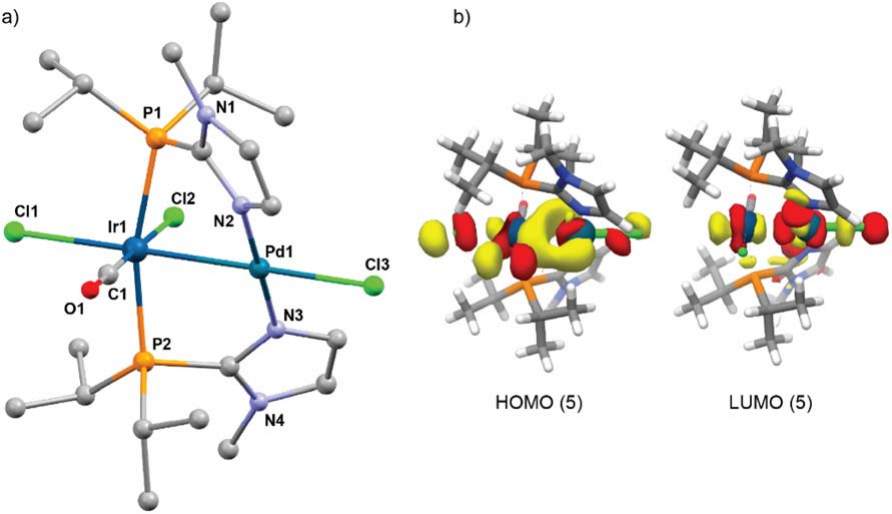
(a) DFT calculated structure of [IrPd(Cl)_3_(CO)(P*i*Pr_2_Imd^Me^)_2_] (5). (b) HOMO and LUMO for the complex 5; DFT calculations were performed at the TPSSh/zora-def2-tzvp(-f), sarc-zora-tzvpp(Ir, Pd), zora-def2-tzvpp(Cl)[CPCM_DMSO_] level of theory.

Efforts to get single crystals of the heterobimetallic complex 5 for X-ray analysis proved to be unsuccessful. DFT calculations using a well-tested hybrid-functional (TPSSh) were conducted to determine the structure of 5, which is illustrated in Fig. 3a. The predicted Ir–Pd bond length in complex 5 (2.601 Å) is shorter than the corresponding distances in the complexes 4 and 6.^47^ In the heterobimetallic complex 5, the highest occupied molecular orbital (HOMO) is predominantly distributed between the two metal centers, indicating significant metal–metal interaction (Fig. 3b, see SI for the corresponding molecular orbitals of 4 and 6).

In initial catalytic studies aniline was selectively methylated using methanol, KO*t*Bu, and 0.5 mol% catalyst at 100 °C under air atmosphere, to yield *N*-methylaniline (10a) in 4 hours. The catalytic activities of the monometallic Ir complexes 1, 2 and 3 were compared to those of the bimetallic catalysts 4, 5 and 6. The performance of bimetallic catalysts is for all cases under the given conditions slightly better over the monometallic Iridium complexes (entries 2–7, Table 2). Kinetic profiles for the bimetallic catalysts 4, 5 and 6 revealed that catalyst 5 gave rise to 99% yield within 3.5 hours. Catalysts 4 and 6 showed a comparable activity, reaching similar yields at a slower rate. Note that with [Ir(Cl)(COD)]_2_ (ref. 70) as a catalyst, double methylation occurred (entry 1, Table 2).^54,55^

**Table 2:** Catalytic *N*-methylation of aniline^a^

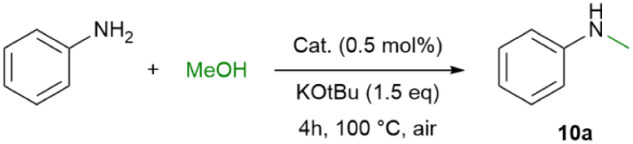				
Entry	Cat.	Base	*T* (°C)	Yield^b^ (%)
1^c^	[Ir(Cl)(COD)]_2_	KO*t*Bu	100	14
2	1	KO*t*Bu	100	83
3	2	KO*t*Bu	100	85
4	3	KO*t*Bu	100	83
5	4	KO*t*Bu	100	96
6	5	KO*t*Bu^d^	100	**99**
7	6	KO*t*Bu	100	93
8	1 + 7	KO*t*Bu	100	53
9	2 + 8	KO*t*Bu	100	74
10	3 + *cis*/*trans*-9	KO*t*Bu	100	75
11	5	K_2_CO_3_	100	47
12	5	Cs_2_CO_3_	100	59
13	5	KO*t*Bu	60	55
14	5	KO*t*Bu	25	30

a Reaction conditions: aniline (1 mmol), methanol (0.2 ml), catalyst (0.5 mol%), base (150 mol%), at 100 °C for 4 h under air atmosphere.

b Yields were determined by GC-MS with mesitylene as the internal standard.

c Reacting for 6 h, the selectivites towards *N*-methylaniline and *N*,*N*-dimethyl-aniline were 10% and 90% respectively.

d Within 3.5 h.

**Fig. 4:**
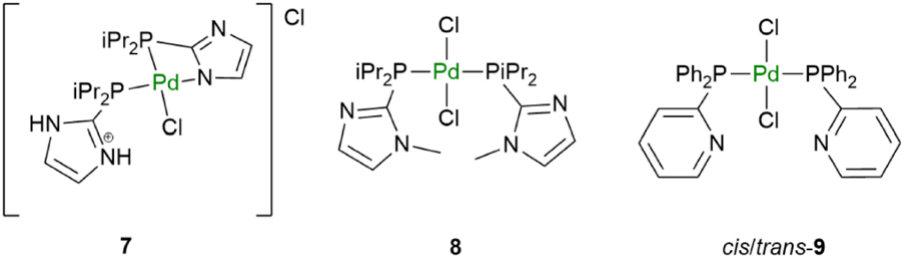
Mononuclear palladium catalysts.

Importantly, mixtures of the monometallic Ir (1–3) and Pd (7–9)^56,57^ catalysts (Fig. 4) showed much lower conversions under the same conditions (entries 8–10, Table 2). Replacing KO*t*Bu with K_2_CO_3_ or Cs_2_CO_3_ as the base resulted in significantly lower conversion when using 5 as the catalytic precursor (entries 11–12, Table 2). The transformation with 5 was also performed at 60 °C and room temperature (entries 13 and 14, Table 2). Although, the activities were lower showing 30% yield for the latter, homogeneous *N*-methylation at room temperature is unprecedented.^58,59^

**Scheme 2:**
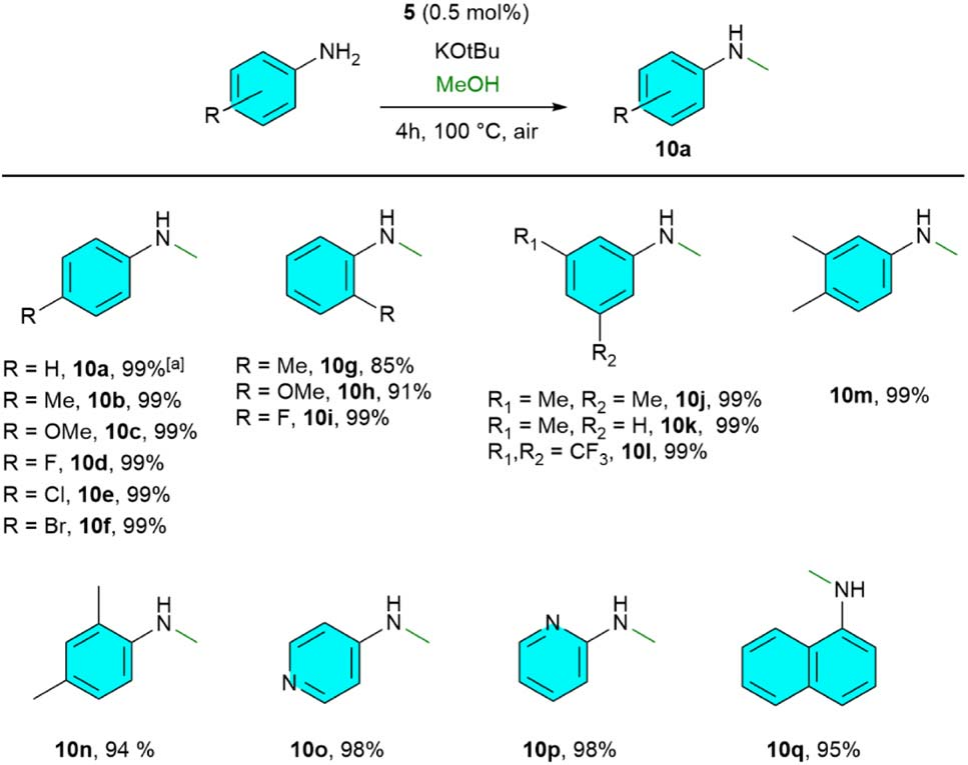
*N*-methylation of primary aromatic amines. Reaction conditions: amine (1 mmol), methanol (0.2 ml), base (150 mol%), 5 (0.5 mol%) at 100 °C for 4 h under air atmosphere. [a] yields for all *N*-methylated products were obtained from GC-MS analysis using mesitylene as the internal standard.

The scope of *N*-methylation was tested with various aniline derivatives, and catalyst 5 was efficiently able to mono-*N*-methylate diverse aromatic amines (Scheme 2). This includes *p*-substituted aromatic anilines with *p*-Me (10b), *p*-OMe (10c), and various halides (10d–10f), producing excellent yields (99%). The sterically hindering *o*-Me (10g) and *o*-OMe (10h) moieties slightly reduced reactivity but still produced excellent yields for *o*-F (10i). Additionally, 3,5-substituted anilines showed a complete conversion (10j–10l). Other aniline derivatives, such as 3.4-dimethyl, 2,4-dimethyl, pyridine, and naphthalene (10m–10q), also achieved nearly excellent yields (94–99%) of mono-*N*-methylated products.

**Scheme 3:**
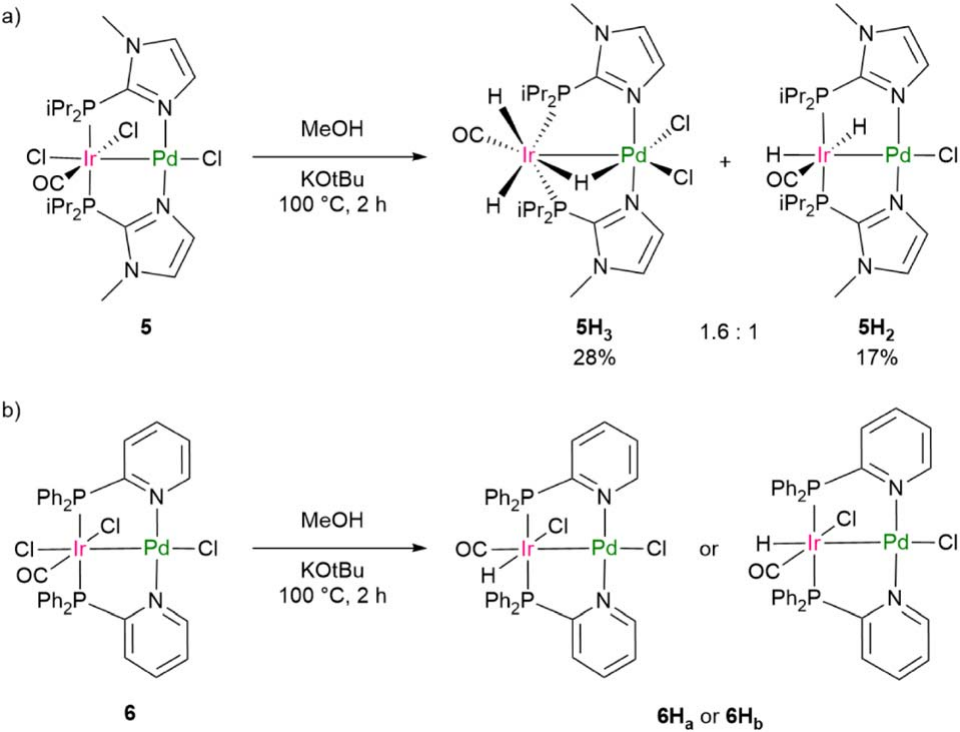
Hydrido complex formation by treatment of 5 (a) and 6 (b) with MeOH.

Mechanistically it can be assumed that methanol undergoes hydrogen transfer dehydrogenation to form formaldehyde by a borrowing hydrogen mechanism mediated by the Ir center.^60–74^ The *N*-methylamine product is then obtained by hydrogenation of the *in situ* generated imine.^70,74^ To gain mechanistic insights into the *N*-methylation catalytic cycle, we examined the reactivity of complexes 4, 5, and 6 in independent reactions. When complex 5 was treated with MeOH under the optimized catalytic conditions, it produced the unique binuclear trihydrido complex 5H_3_ and a dihydrido complex 5H_2_ in a 1.6 : 1 ratio based on the ^1^H NMR data (Scheme 3a). Complex 6 yielded a hydrido complex (6H_a_ or 6H_b_, Scheme 3b), which exhibits a hydrido ligand in the *cis* position to the CO ligand (Scheme 3b; (^1^H NMR) *δ* = −16.58 ppm). The reaction of complex 4 with MeOH generated a dihydrido complex in low yields, which could not be characterized further.

**Scheme 4:**
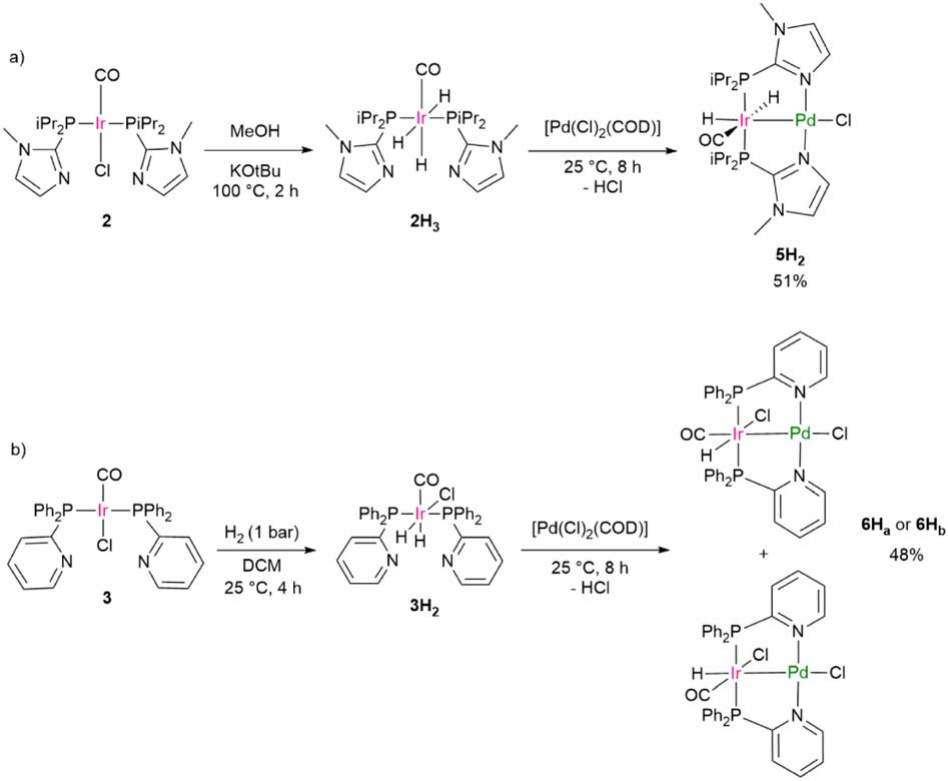
Independent synthesis of 5H_2_ (a), 6H_a_ and 6H_b_ (b).

Independent routes to generate the complexes 5H_2_ and 6H_a_/6H_b_ were also developed (Scheme 4). Complex 2 generated with MeOH and KO*t*Bu to give the mononuclear hydrido iridium complex 2H_3_, which further reacted with [Pd(Cl)_2_(COD)] to yield the bimetallic complex 5H_2_ (Scheme 4a). Note that a synthesis of the complex 5H_3_ proved unfeasible by treatment of 5H_2_ with HCl. Upon exposure of a solution of complex 3 to H_2_, the dihydrido complex 3H_2_ formed, as verified by X-ray crystallography (see SI). 3H_2_ was then treated with [Pd(Cl)_2_(COD)], producing in this case two isomeric monohydride complexes of 6H_a_/6H_b_ (Scheme 4b), one of which is the one that is also produced from 6 by reaction with MeOH.

^1^H NMR data for 5H_3_ showed that all the hydrido ligands are located in a *cis* position to the phosphine ligands. Two signals appear as triplet of doublets and triplet of triplets signals at (*δ* = −11.97 and −13.07 ppm, respectively) which integrate 2 : 1 (see SI). ^2^*J*_H,P_ coupling constants of 14.7 Hz and 19.8 Hz confirm the *cis* positions to phosphines. However, the latter hydride has a *trans* position to the CO ligand as confirmed by NMR data of the ^13^CO labeled isotopomer of 5H_3_ (see SI).^47^ For 5H_2_ two sets of triplet of doublet signals (*δ* = −8.91 and −20.43 ppm) with an 1 : 1 integration indicate the presence of two hydrido ligands (^2^*J*_H,P_ coupling constants of 18.5 Hz and 13.0 Hz, respectively).

The IR spectra of 5H_2_ and 5H_3_ showed three bands each: 1947, 1982, and 2097 cm^−1^ for 5H_2_, and 2115, 1997, and 2158 cm^−1^ for 5H_3_. These can be assigned to CO and two hydrido ligands at the iridium centers;^47^ the data fit to the calculated IR spectra for 5H_2_ and 5H_3_ (1940/1998/2099 cm^−1^ and 2133/2003/2169 cm^−1^, see below).

**Fig. 5:**
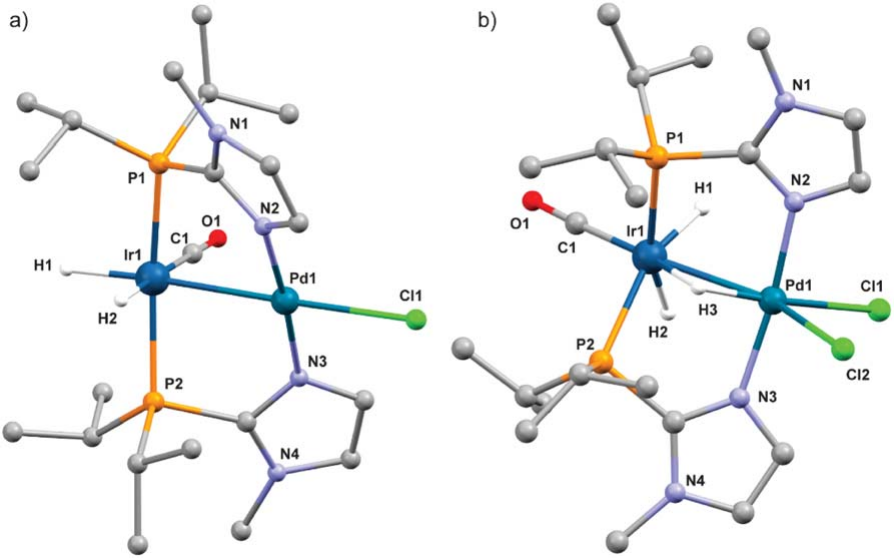
DFT optimized structures of 5H_2_ (a) and 5H_3_ (b).

Several attempts to obtain single crystals from the 5H_3_/5H_2_ mixture were unsuccessful due to their low stability. Therefore, DFT was used to analyze the intermediates. Fig. 5 shows the optimized structures for 5H_2_ and 5H_3_. Based on these structures the Ir–Pd bond lengths are 2.655 Å and 2.644 Å for 5H_3_ and 5H_2_ respectively. The Ir center in 5H_3_ features a distorted coordination environment resulting in two hydrido ligands that could be considered magnetically equivalent as well as a bridging hydrido ligand. A comparable configuration has also been reported for binuclear Ir/Ir and Ir/Rh complexes.^25^ Other possible isomers for 5H_3_ did not converge.

The ^1^H NMR spectra of the two isomers 6H_a_/6H_b_ displayed two triplet signals at *δ* = −15.39 and −16.58 ppm (^2^*J*_H,P_ = 11.5 and 15.0 Hz respectively) in a 1 : 1 ratio. ^1^H NMR spectra of the ^13^C isotopologues confirm the hydrides to be in the *cis* positions to the respective ^13^CO ligands (^2^*J*_H,C_ = 5.2 and 3.1 Hz, respectively).^47^ The IR spectrum of a dichloromethane solution containing the isomers 6H_a_ and 6H_b_ revealed CO absorption bands at 2047 and 1994 cm^−1^,^47^ along with bands for the hydrido ligands at 2180 and 2192 cm^−1^. The relative energies of the two isomers were calculated by DFT. 6H_a_, which is defined to be the complex with the hydrido ligand *trans* to Cl, is 3.15 kcal mol^−1^ more stable than 6H_b_. Another hypothetical structure with the hydride in the *trans* position to the CO ligand 6H_c_ is 2.33 kcal mol^−1^ less stable than 6H_a_. However, 6H_c_ was ruled out by NMR spectroscopy (see above), despite thermodynamic accessibility.

**Scheme 5:**
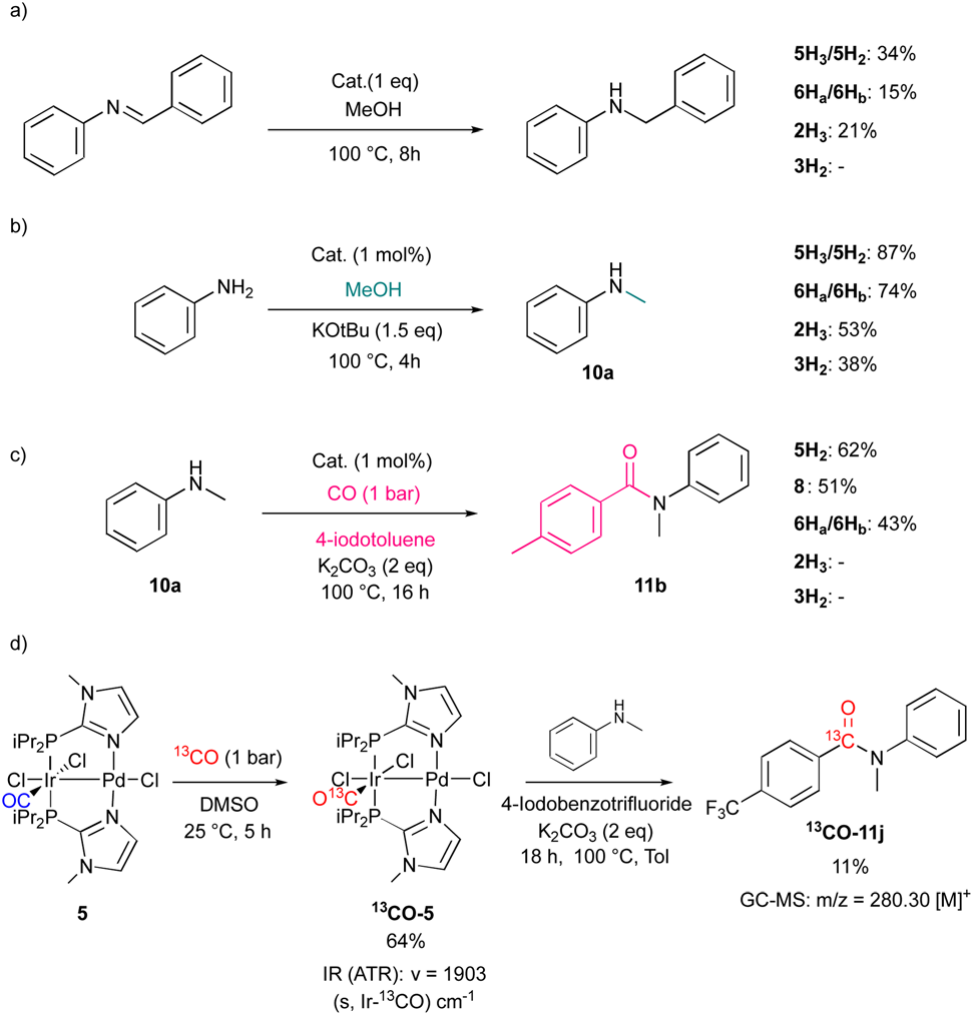
Model reactivity studies of hydrido or carbonyl complexes; (a) imine bond hydrogenation. (b) *N*-methylation of aniline using hydrido complexes. (c) Aminocarbonylation of 10a. (d) Reactivity of 5 toward ^13^CO and its activity in the aminocarbonylation of *N*-methylaniline.

Monitoring the reaction solution for the conversion of 4-fluoroaniline to give 10d with 5 as pre-catalyst by ^1^H NMR and ^19^F NMR spectroscopy revealed the presence of the hydrido species 5H_3_ and 5H_2_, indeed (see SI). Another model reaction showed that the hydrides can act as hydrogen sources for imine hydrogenation. Thus, treatment of *N*-benzylideneaniline with a solution of 5H_3_/5H_2_ or 6H_a_/6H_b_ in the presence of methanol led to the generation of the corresponding amine with 34% and 15% yield, as confirmed by GC-MS (Scheme 5a). Note that 5H_2_ is more reactive as at the end of the reaction only small amounts of 5H_3_ remained. The mononuclear species 2H_3_, however, was slightly less reactive under these conditions, while 3H_2_ showed no detectable conversion.^64–69^

Notably, catalytic *N*-methylation experiments using methanol solutions of 5H_3_/5H_2_, 6H_a_/6H_b_ produced 10a in marginally higher yields than those reactions employing the monometallic iridium hydrides 2H_3_ and 3H_2_ (Scheme 5b). Experiments with methanol-d_4_ and aniline resulted in the formation of labeled secondary amine. The kinetic isotope effect (KIE) for the *N*-methylation of aniline was also studied on using CH_3_OH and CD_3_OD. The reactions were monitored by GC-MS with mesitylene as an internal standard and a KIE value of 2.04 was determined from the ratio of initial rates (see SI).^70,74^

Under the optimized conditions using the bimetallic catalysts for *N*-methylation (entries 5–7, Table 3), we then investigated the aminocarbonylation of *N*-methylaniline. Thus, reactions of 10a with 4-iodotoluene were run under CO atmosphere in the presence of a base using 4, 5, and 6 as pre-catalysts (entries 1–3, Table 3). With 1 mol% of 5 amide 11f was formed with 85% yield when the reaction was run in the presence of K_2_CO_3_ as base in toluene at 100 °C within 16 h under atmospheric CO pressure (entry 1, Table 3). 5 showed a slightly better activity than 4 or 6 (entries 1 and 3, Table 3). As the carbonylation steps are presumably mainly mediated by the Pd center,^75–94^ the bimetallic systems were compared with mononuclear Pd catalysts (7, 8 and 9), all showing a significant decrease in the 11f generation (entries 4–6, Table 3). Remarkably, testing mixtures of mononuclear Ir and Pd complex corresponding to the bimetallic complexes also resulted in reduced yields (entries 7 and 8, Table 3). Moreover, using K_3_PO_4_ as a base with 5 as the catalyst or DMF as a solvent also gave a reduced yield (entry 9–11, Table 3). Overall, bimetallic systems outperform mononuclear counterparts in carbonylation of secondary amines, suggesting on a cooperative effect between two metal centers. Presumably, the carbonylation process is predominantly mediated by the Pd center. A classic reaction sequence involves an oxidative addition of the aryl halide, an amido complex formation, insertion of CO into the Pd–C bond, and subsequent reductive elimination of the C–N bond to yield the amide product;^79,81,83^ however, the involvement of the iridium centre in this transformation remains uncertain.

**Table 3:** Catalytic aminocarbonylation of *N*-methylaniline^a^

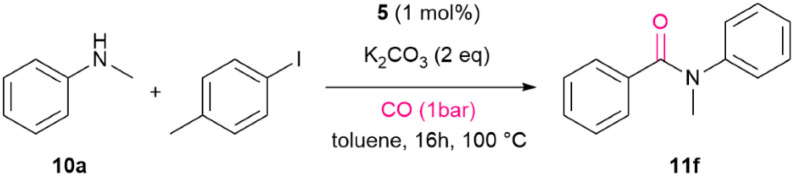				
Entry	Cat.	Base	Solvent	Yield^b^ (%)
1	4	K_2_CO_3_	Toluene	80
2	5	K_2_CO_3_	Toluene	99
3	6	K_2_CO_3_	Toluene	77
4	7	K_2_CO_3_	Toluene	50
5	8	K_2_CO_3_	Toluene	51
6	9	K_2_CO_3_	Toluene	45
7	2 + 8	K_2_CO_3_	Toluene	30
8	3 + 9	K_2_CO_3_	Toluene	35
9	5	K_3_PO_4_	Toluene	70
10	5	K_2_CO_3_	DMF	35
11	5	Et_3_N	DMF	40

a Reaction conditions: cat. (1 mol%), amine (1 mmol), aryl iodide (1.2 mmol), base (2 eq) and CO (1 bar).

b Yields were determined by GC-MS with mesitylene as the internal standard.

In model reactions, toluene solutions of 5H_2_ and 5H_3_ were then reacted with 4-iodobenzotrifluoride. The formation of trifluorotoluene as a hydrodehalogenated product with 35% yield was observed, whereas the ^1^H NMR spectra showed various hydride complexes, which were not characterized further. Repeating the reaction with 6H_a_/6H_b_ did not produce trifluorotoluene.

The complexes 5H_2_ and 6H_a_/6H_b_ were then tested in the carbonylation of 10a with 4-iodotoluene to yield successfully 11b with 62% and 43% yield, respectively (Scheme 5c). The mononuclear Pd catalyst 8 delivered a slightly lower yield (51%) than 5H_2_ (Scheme 5c). The Ir hydride species 2H_3_ and 3H_2_ showed no catalytic activity. Note that 5H_3_ is not stable in the presence of CO, and the monohydrido complex [IrPdCl_2_(H)(CO)(P*i*Pr_2_Im^Me^)_2_] (5H) is formed under H_2_ release, as confirmed by the ^1^H NMR spectrum. However, after five days the generation of 5H_2_ was observed (see SI). Furthermore, upon exposure of complex 5 to 1 bar atmosphere of ^13^CO, the formation of ^13^CO-5 was observed. Note that ^13^CO-5 could also be generated alternatively from ^13^CO-2 carbonyl ligand exchange (see SI). *In situ*–formed ^13^CO-5 was then treated in a stoichiometric reaction with 4-iodobenzotrifluoride and 10a in the absence of additional CO. The generation of the ^13^C labelled carbonylation product ^13^CO-11j was observed (Scheme 5d). This indicates a partial participance of the Ir centre during the carbonylation step as it can provide the CO moiety for carbonylation.

**Scheme 6:**
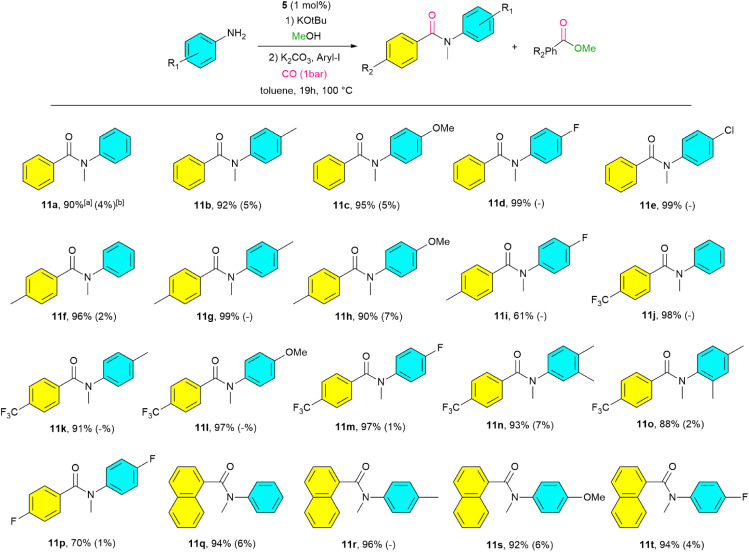
Catalyzed *N-*methylation and subsequent carbonylation using various aryl iodides. Reaction conditions: amine (1 mmol), MeOH (0.2 ml), KO*t*Bu (1.5 eq), K_2_CO_3_ (2 eq), aryl iodide (1.2 eq), 5 (1 mol%), toluene (3 ml). ^*a*^All yields are isolated yields. ^*b*^Yields for esterification are shown in the parentheses.

The scope of the catalytic synthesis of carboxyamides was then explored on running consecutive one-pot *N*-methylation and aminocarbonylation reactions (Scheme 6). It has to be emphasized that these conversions can also be classified as assisted-relay catalysis.^7^ Complex 5 enabled the sequential *N*-methylation of aniline derivatives with MeOH and subsequent carbonylation with iodobenzene to achieve 90–99% yields for 11a–11e. Note that the presence of unreacted MeOH can hamper the aminocarbonylation step resulting in an esterification (Scheme 6).^75,76^

Changing the substrate to 4-iodotoluene yielded 11f–11i, with a reduced yield for 11i. Using 4-iodobenzotrifluoride increased yields for 11j–11m with fully converted aniline and 4-fluoroaniline to 11j, 11l and 11m, with decreased esterification and maintaining good yields for 11n–11o. Note that 11l–11o were not described before. In contrast, 4-fluoroaniline and 4-fluoroiodobenzene gave significantly lower yields (11p). The use of 1-iodonaphthalene^77^ as the aryl halide afforded high yields (11q–11t), with excellent selectivity for naphthamides formation. We also exploited bromobenzene and chlorobenzene as aryl halide substrates. With aniline as substrate only traces of 11a were produced. However, 1-bromonaphthalene yielded 55% of 11p. Note that, in contrast, two literature reported examples for nitroarene methylation and a subsequent carbonylation using a palladium acetate/phosphine system under higher CO pressure proceeded *via* a two-step process.^95^

## Conclusions

In conclusion, we developed a unique one-pot process for the selective mono*-N*-methylation of primary amines that is coupled with an aminocarbonylation. Bimetallic iridium/palladium complexes were used as catalytic precursors. The approach introduces both alkyl and carbonyl functionalities in two coupled catalytic cycles, offering advantages concerning atom economy and synthetic efficiency. Remarkably, the bimetallic catalysts outperform their monometallic counterparts in both *N*-methylation and carbonylation. The former is presumably mainly mediated by the iridium center, whereas for the latter the palladium center seems to be crucial. In model reactions hydrido complexes could be identified as possible intermediates. In addition, the developed method requires only 1 bar of CO and there is no need for high-pressure setups.^81,93^

## Author contributions

Conceptualization, A. A., T. B. and M. R.; experimental and theoretical studies, A. A., K. K. and P. W.; X-ray crystallography, O. H.; writing – original draft preparation, A. A.; writing – review and editing, A. A., P. W. and T. B.; funding acquisition, T. B.

## Conflicts of interest

There are no conflicts to declare.

## Supplementary Material

SC-OLF-D5SC03892H-s001

SC-OLF-D5SC03892H-s002

## Data Availability

CCDC 2430139 (2), 2430140 (3H_2_) and 2430141 (4) contain the supplementary crystallographic data for this paper.^96*a*–*c*^ Supplementary information: details of the experimental procedures, characterization of the complexes and the DFT calculated details. See DOI: https://doi.org/10.1039/d5sc03892h.
