# Supplementary material for: Sequential one-pot N-alkylation and aminocarbonylation of primary amines catalyzed by heterobimetallic Ir/Pd complexes
Source: Chem Sci. 2025 Sep 15;16(41):19414–22. doi: 10.1039/d5sc03892h (PMC12459202; doi:10.1039/d5sc03892h)
Supplement: SC-016-D5SC03892H-s001 [file SC-016-D5SC03892H-s001.pdf]

Supplementary Information (SI) for Chemical Science.
This journal is © The Royal Society of Chemistry 2025

Supporting Information

Sequential One-Pot *N*-Alkylation and Aminocarbonylation of Primary Amines Catalyzed by Heterobimetallic Ir/Pd Complexes

Amin Abdolrahimi, [a] Philipp Woite, [a] Konrad Kretschmar, [a] Michael Roemelt, * [a] Thomas Braun*[a] and Ouchan He [a]

[a] A. Abdolrahimi, P. Woite, Dr. K. Kretschmar, Prof. Dr. M. Roemelt, Prof. Dr. T. Braun, O. He
Humboldt-Universität zu Berlin,
Institut für Chemie
Brook-Taylor-Straße 2, 12489 Berlin (Germany)
E-mail: thomas.braun@cms.hu-berlin.de

Table of contents

1. Experimental Procedures 3

1.1. General methods 3

1.2. Synthesis and characterization of [IrCl(CO)(P*i*Pr$_2$Im^{Me})$_2$] (Im^{Me} = 1-methyl-1H-imidazole) (**2**) 4

1.3. Synthesis and characterization of *cis*-[PdCl(P*i*Pr$_2$Im)(P*i*Pr$_2$Im-κ*P*,κ*N*^{1})$_2$]Cl (Im = imidazole) (**7**) 7

1.4. Synthesis and characterization of *trans*-[PdCl$_2$(P*i*Pr$_2$Im^{Me})$_2$] (Im^{Me} = 1-methyl-1H-imidazole) (**8**) 10

1.5. Synthesis and characterization of *cis/trans*-[PdCl$_2$(PPh$_2$py)$_2$] (*cis/trans*-**9**)[14] 11

1.6 Synthesis and characterization of [IrPdCl$_3$(CO)(P*i*Pr$_2$Im)$_2$] (**4**) 13

1.7 Synthesis and characterization of [IrPdCl$_3$(CO)(P*i*Pr$_2$Im^{Me})$_2$] (Im^{Me} = 1-methyl-1H-imidazole) (**5**) 15

1.8 Synthesis and characterization of [IrPdCl$_3$(CO)(PPh$_2$py)$_2$] (**6**) (New synthetic route) [5]. 18

1.9 General procedure for the *N*-Alkylation of aniline with methanol. 19

1.10 General procedure for the one-pot aminocarbonylation of primary amines 19

1.11 Methanol dehydrogenation with **5** 20

1.12 Monitoring the *N*-mehtylation process 24

1.13 Independent synthesis of **5H$_2$** 26

1.14 ^{13}CO labeling experiments 30

1.15 Methanol dehydrogenation with **^{13}CO-5** 36

1.16 Formation of the species **5H** 39

1.17 Synthesis of **3H$_2$** [5] 41

1.18 Methanol dehydrogenation with **6** 44

1.19 Independent synthesis of **6H$_a$/6H$_b$** 45

2. Quantum chemical calculations 51

3. Characterization data of organic products 73

3.1 Analytical data of organic products 73

3.2 Copies of ^{1}H, ^{13}C NMR and ^{19}F NMR spectra of the *N*-methylated amines and amides. 80

3.3 Copies of HRMS and IR spectra of the newly synthesized amides 132

Crystallographic data for the complexes **2**, **4** · 2 DMSO and **3H$_2$** 142

5. References 144

1. Experimental Procedures

1.1. General methods

The synthetic work was carried out at a Schlenk line or in glove box under an atmosphere of argon. Solvents were dried by the usual procedures and, prior to use, distilled under argon. [{IrCl(COD)}$_2$] (COD = 1,5-Cyclooctadiene),[1] P*i*Pr$_2$Im[2] (Im = imidazole), [IrCl(CO)(P*i*Pr$_2$Im)$_2$] (**1**)[3], [Ir(CO)Cl(PPh$_2$py)$_2$] (**3**)[5] and *cis/trans*-[PdCl$_2$(PPh$_2$py)$_2$] (*cis-trans* **9**)[4] were prepared as described in the literature. [IrPdCl$_3$(CO)(PPh$_2$py)$_2$] (**6**)[5] was reported before, but was synthesized in a different manner. All other reagents were obtained from commercial sources. The NMR spectra were recorded on a Bruker Avance III 500, a Bruker Avance 400, a Bruker DPX 300, a Bruker Avance II 300 or a Bruker Avance 300 NMR spectrometer. The ^{1}H NMR chemical shifts were referenced to residual CD_2Cl_2 at δ = 5.32 ppm, CD_3OD at δ = 3.31 ppm, $CDCl_3$ at δ = 7.26 ppm or DMSO-d$_6$ at δ = 2.50 ppm. The ^{13}C NMR chemical shifts were referenced to residual CD_2Cl_2 at δ = 53.84 ppm, $CDCl_3$ at δ = 77.16 ppm, CD_3OD at δ = 49.00 ppm or DMSO-d$_6$ at δ = 39.52 ppm. The ^{19}F NMR spectra were referenced to external $CFCl_3$ at δ = 0.0 ppm. The ^{31}P{^{1}H} NMR chemical shifts were referenced to external H_3PO_4 at δ = 0.00 ppm. High resolution mass spectra (ESI-MS) were recorded with an Agilent Technologies 6230 LC/TOF instrument with electro spray ionization (ESI-MS) or with an ADVION EXPRESSION CMS spectrometer. Attenuated total reflection (ATR) ATR-IR spectra were recorded inside a glovebox on a Bruker ALPHA II spectrometer equipped with an ATR-module (diamond). Elemental analyses (EA) were performed with a HEKAtech Euro EA Elemental Analyzer. GC–MS measurements were conducted using an Agilent 6890N gas chromatograph with a capillary column (Agilent 19091S-433 Hewlett-Packard 5 MS: 30 m length, 0.25 mm inside diameter, 0.25 µm film thickness) and an Agilent 5973 Network mass-selective detector. Helium (0.74 bar, 1.2 mL min^{-1}, 40 cm s^{-1}) was used as the carrier gas. The electron impact ionization was carried out with an ionization voltage of 70 eV. HR-MS measurements were performed on a Xevo G3 QTof.

Structure determination of the complexes 2, **4** · 2 DMSO and **3H$_2$** (CCDC deposition numbers 2430139 for **2**, 2430141 for **4** · 2 DMSO, and 2430140 for **3H$_2$**) were obtained by gas phase diffusion of *n*-pentane into a saturated DCM or DMSO solutions at room temperature. The diffraction data were collected at a Bruker D8 Venture diffractometer at 100 K using Mo-Kα (λ = 0.71073 Å) radiation. Multi-scan absorption corrections using the implemented SADABS software were applied to the data.[6] The structures were solved by intrinsic phasing method (SHELXT 2014/5)[7] and refined by full-matrix least-squares methods on F2 (SHELXL 2016/4 or SHELXL-2018/3)[8].

1.2. Synthesis and characterization of [IrCl(CO)(P*i*Pr2Im^{Me})2] (Im^{Me} = 1-methyl-1H-imidazole) (**2**)

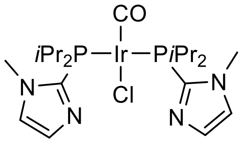

A suspension of [{IrCl(COD)}$_2$] (1000 mg, 1.489 mmol) in toluene (10 mL) was treated with P*i*Pr$_2$Im^{Me} (1180 mg, 5.956 mmol) and stirred for 15 min. The reaction mixture was degassed two times, and the vessel was filled with CO (1 bar) and stirred for 20 h. All volatiles were removed *in vacuo* and the residue was washed with Et_2O (3x10 mL). After recrystallisation in toluene **2** (1210 mg, 1.855 mmol, 62%) was obtained as pale-yellow solid. **^{1}H NMR:** (300 MHz, DCM-d$_2$) δ = 7.20 (m, 2H, Im-H), 7.12 (m, 2H, Im-H), 4.21 (s, 6H, N-CH_3), 3.06 (m, hept in the ^{1}H{^{31}P} NMR spectrum, $^{3}J_{H,H}$ = 7 Hz, 4H, CH), 1.30-1.22 (m, d in ^{1}H{^{31}P} NMR spectrum, $^{3}J_{H,H}$ = 7 Hz, 12H, *i*Pr-CH_3) 1.19-1.12 (m, d in ^{1}H{^{31}P} NMR spectrum, $^{3}J_{H,H}$ = 7 Hz, 12H, *i*Pr-CH_3) ppm. **^{31}P NMR:** (121 MHz, DCM-d$_2$) δ = 25.9 (s) ppm. **ESI-MS** (+) (m/z) [M-Cl]$^+$ calculated for $C_{21}H_{38}IrN_4OP_2$, 617.2; found, 617.3. **IR (ATR):** $\tilde{\nu}$ = 1942 (s, CO) cm^{-1}. **Elemental analysis (%)** calculated for $C_{21}H_{38}ClIrN_4OP_2$ (652.18): C 38.68, H 5.87, N 8.59; found: C 38.96, H 5.99, N 8.52.

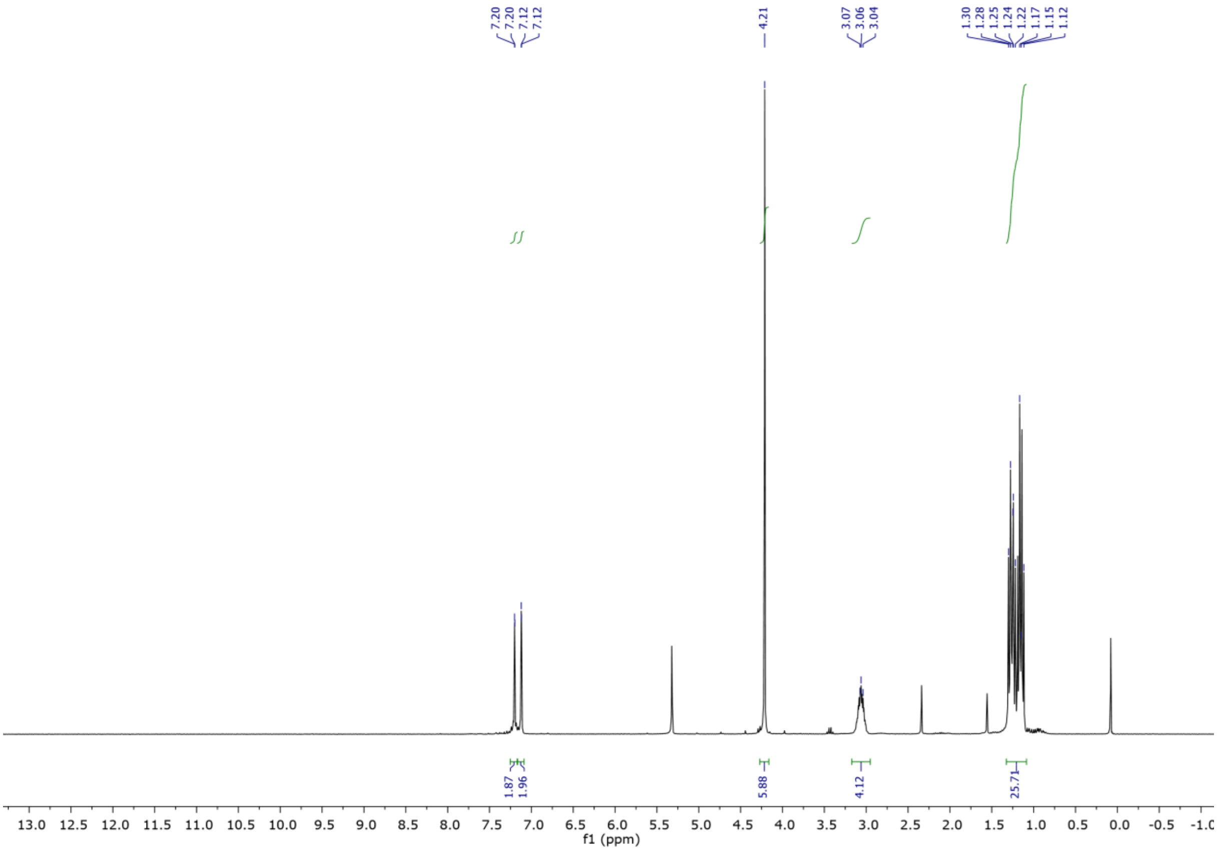

Figure S1. ^{1}H NMR spectrum of complex [IrCl(CO)(P*i*Pr$_2$Im^{Me})$_2$] (**2**) (DCM-d$_2$, 300 MHz)

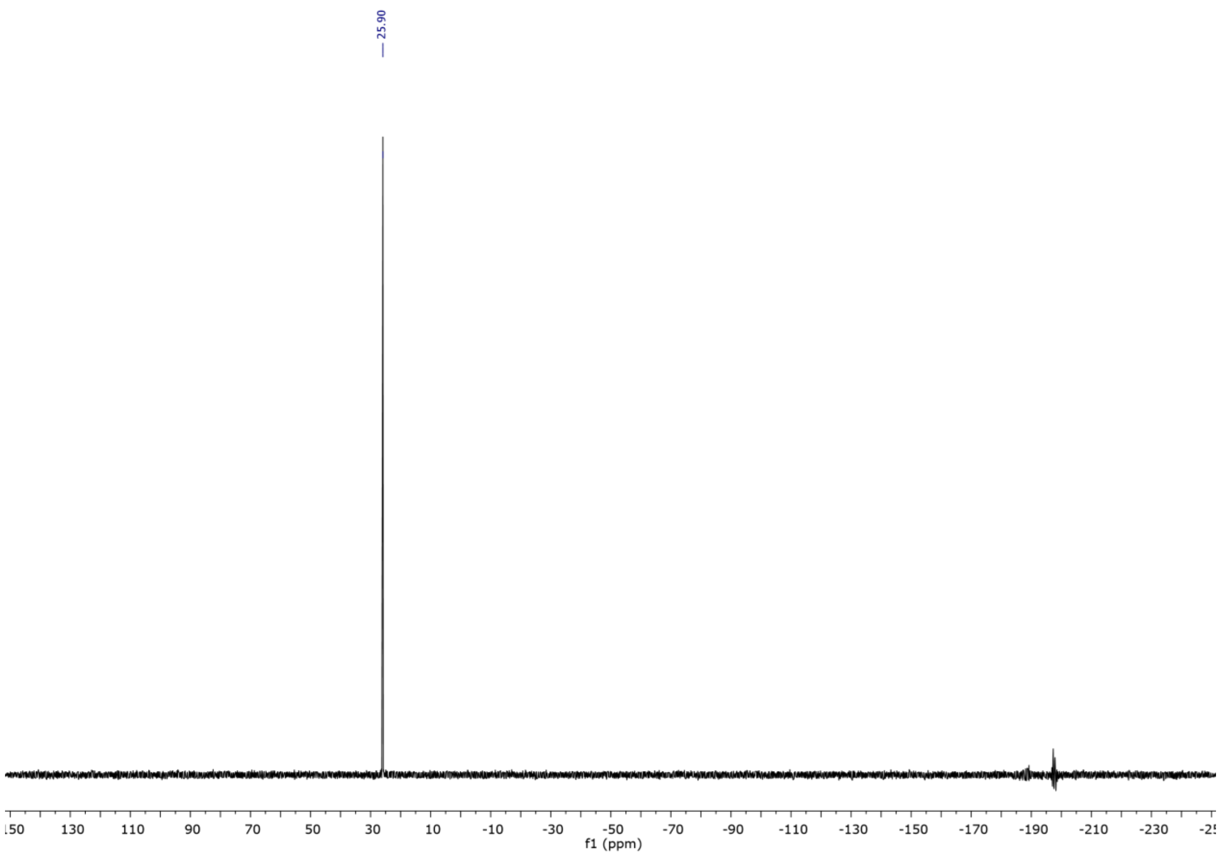

Figure S2. $^{31}P\{^{1}H\}$ NMR spectrum of complex $[IrCl(CO)(P\textit{i}Pr_2Im^{Me})_2]$ (**2**) (DCM-d_2, 121 MHz)

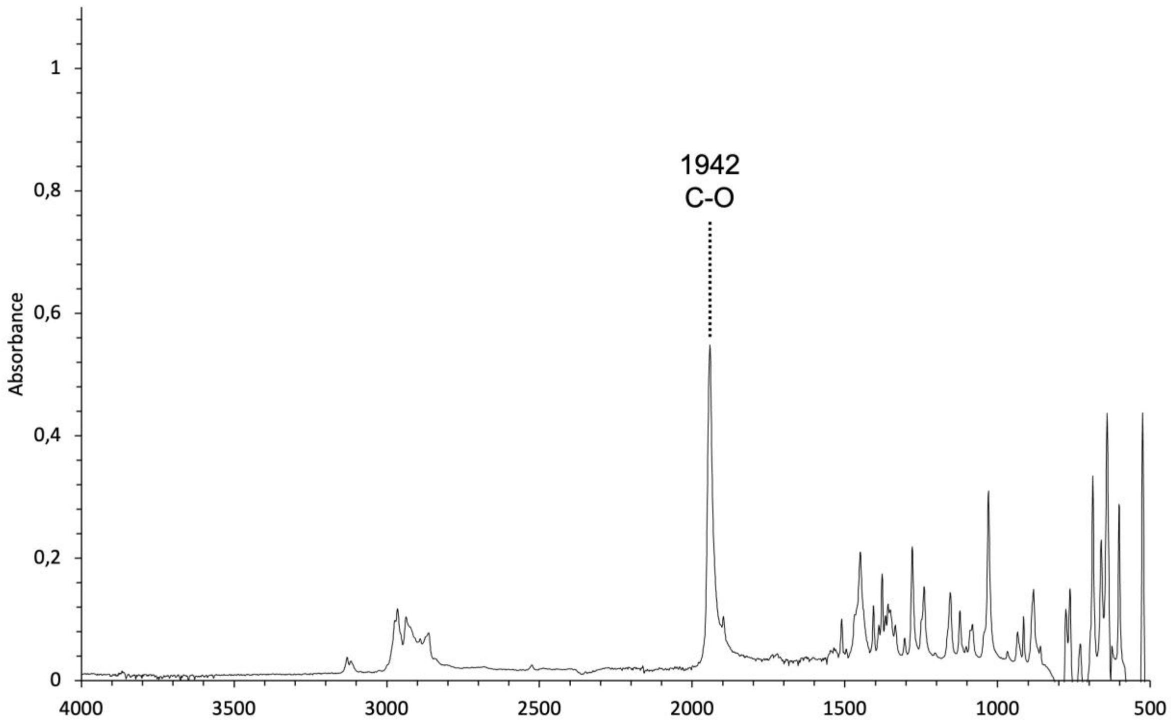

Figure S3. IR spectrum of complex $[IrCl(CO)(P\textit{i}Pr_2Im^{Me})_2]$ (**2**)

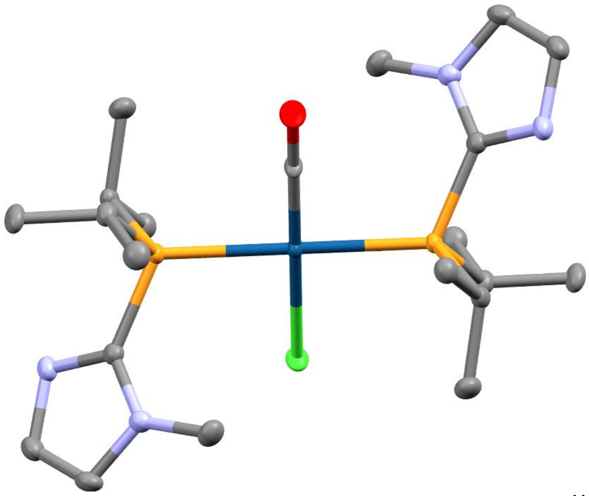

Figure S4. Molecular structure of complex [IrCl(CO)(P*i*Pr$_2$Im^{Me})$_2$] (**2**)

1.3. Synthesis and characterization of *cis*-[PdCl(P*i*Pr$_2$Im)(P*i*Pr$_2$Im-κ*P*,κ*N*^{1})$_2$]Cl (Im = imidazole) (**7**)

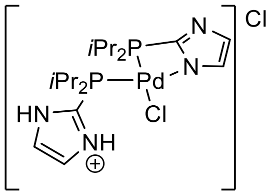

A suspension of [PdCl$_2$(COD)] (300 mg, 1.051 mmol) in DCM (10 mL) was treated with P*i*Pr$_2$Im (387 mg, 2.102 mmol) and stirred for 1 h. The solvent was filtered off, and the residue was washed first with EtOH (1x2 mL), then with Et$_2$O (3x10 mL) and lastly with *n*-pentane (3x10 mL). All volatiles were removed *in vacuo* to yield **7** (480 mg, 0.876 mmol, 83%) as pale-yellow solid.

^{1}H NMR: (300 MHz, DMSO-d$_6$): δ = 13.06 (s, br, 2H, NH), 7.99 (m, 1H, Im-H), 7.81 (m, 1H, Im-H), 7.57 (m, 2H, Im$^+$-H), 2.83 (m, 4H, CH); 1.38-1.11 (m, 18H, *i*Pr-CH$_3$), 0.85 (dd, $^2J_{H,P}$ = 20 Hz, $^2J_{P,P}$ = 7 Hz, 5H, *i*Pr-CH$_3$) ppm.**^{31}P NMR:** (121 MHz, DMSO-d$_6$): δ = 120.96 (d, $^2J_{P,P}$ = 5 Hz), 39.73 (d, $^2J_{P,P}$ = 5 Hz) ppm. **^{1}H,^{15}N HMBC NMR** (300 MHz/30 MHz, DMSO-d$_6$): δ = 13.1/-193 ppm (d/s, $^1J_{N,H}$ = 97 Hz, NH) ppm. **^{1}H,^{15}N HMBC NMR** (300 MHz/30 MHz, DMSO-d$_6$): δ = 8.0/-195 ppm (s, Im-H), 7.8/-95 (s, Im-H), 7.6/-193 (s, Im$^+$-H) ppm. **IR (ATR):** $\tilde{\nu}$ = 3177 (br, NH) cm^{-1}. **ESI-MS** (+) (m/z) [M-Cl]$^+$ calculated for $C_{18}H_{36}ClN_4P_2Pd$, 511.1; found, 511.2. **Elemental analysis (%)** calculated for $C_{18}H_{36}Cl_2N_4P_2Pd$ (547.78): C 39.47, H 6.62, N 10.23; found: C 39.04, H 6.24, N 10.87.

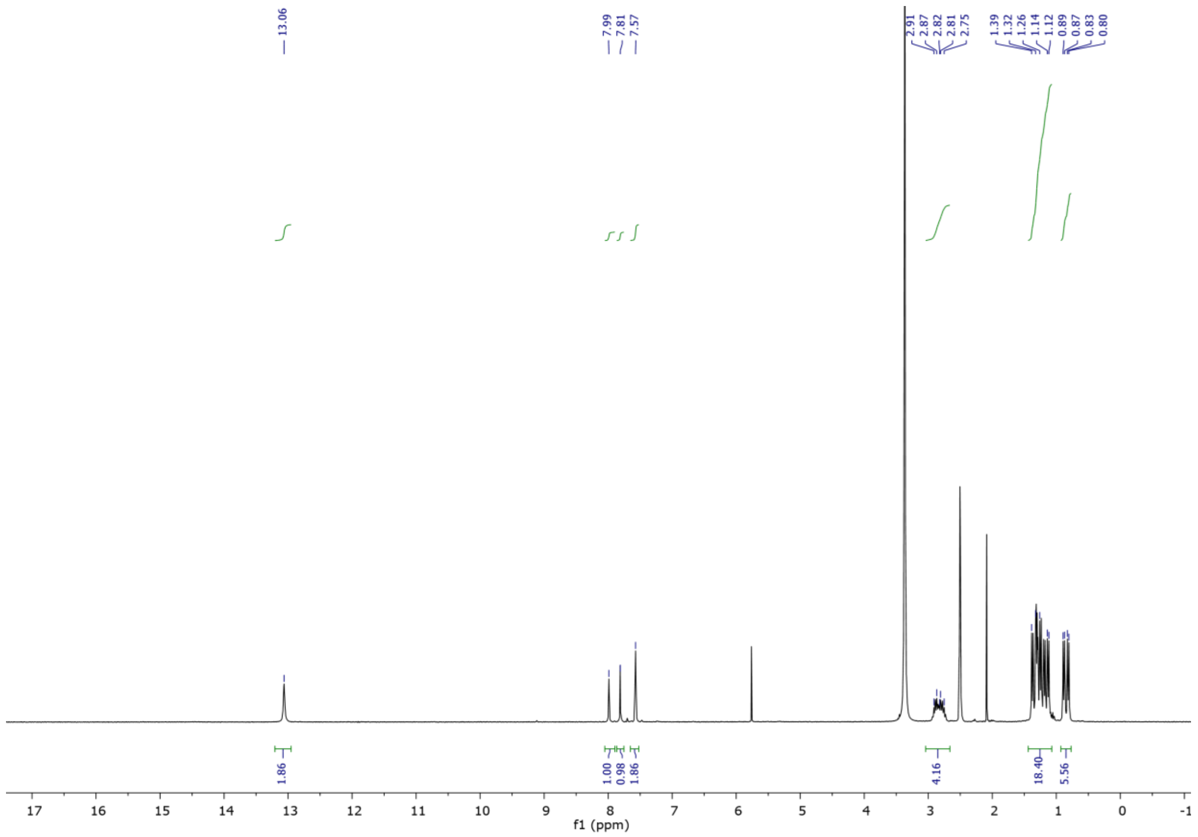

Figure S5. ^{1}H NMR spectrum of complex *cis*-[PdCl(P*i*Pr$_2$Im)(P*i*Pr$_2$Im-κ*P*,κ*N*^{1})$_2$]Cl (**7**) (DMSO-d$_6$ 300 MHz)

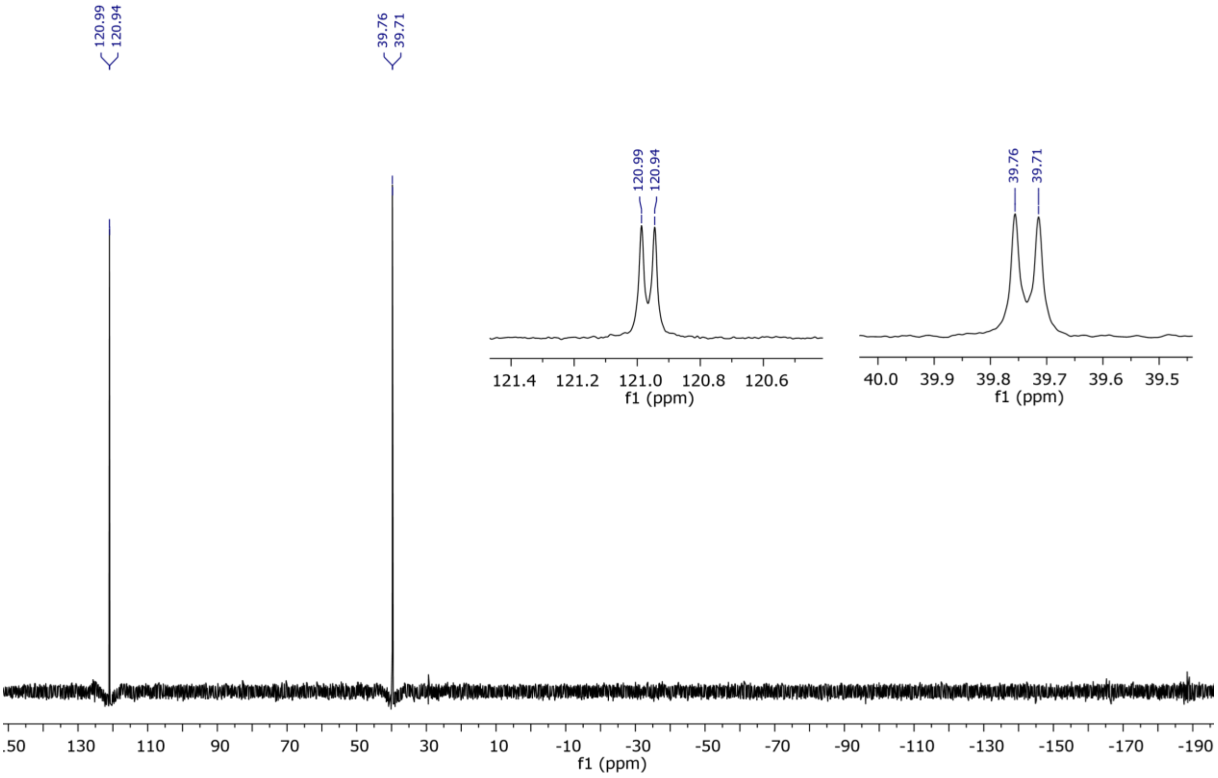

Figure S6. $^{31}P\{^1H\}$ NMR spectrum of complex *cis*-[PdCl(P*i*Pr$_2$Im)(P*i*Pr$_2$Im-κ*P*,κ*N^1*)$_2$]Cl (**7**) (DMSO-d$_6$, 121 MHz)

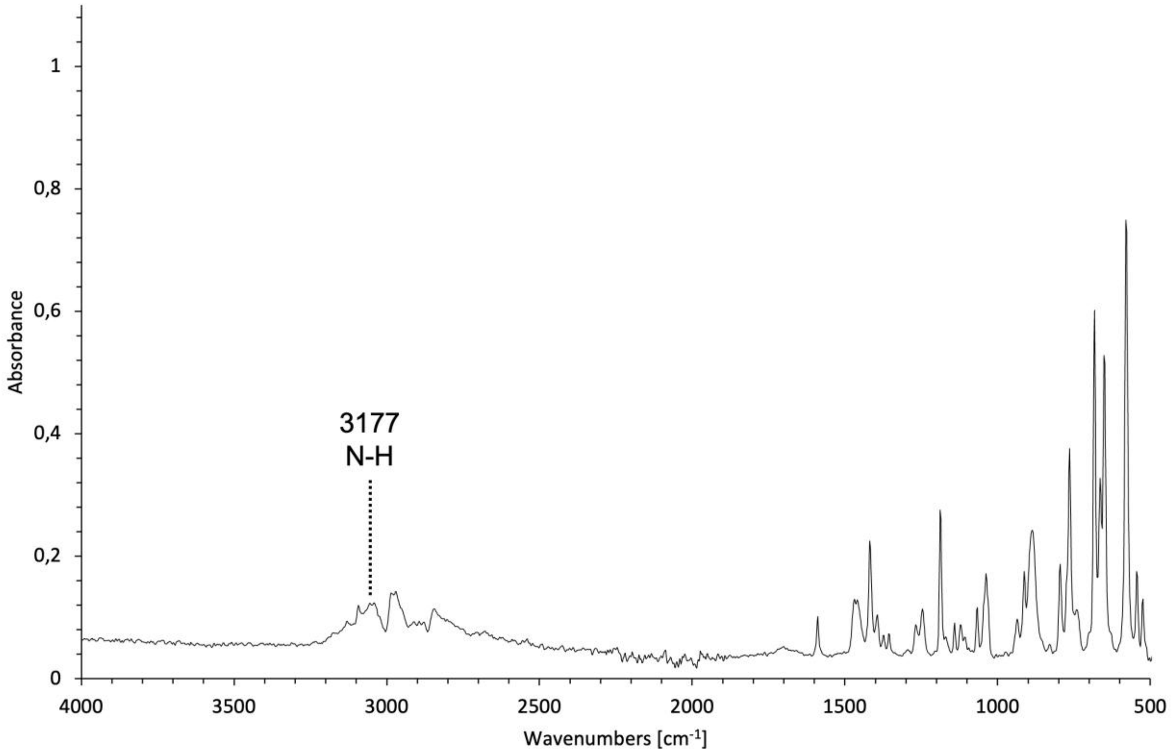

Figure S7. IR spectrum of complex *cis*-[PdCl(P*i*Pr$_2$Im)(P*i*Pr$_2$Im-κ*P*,κ*N^1*)$_2$]Cl (**7**)

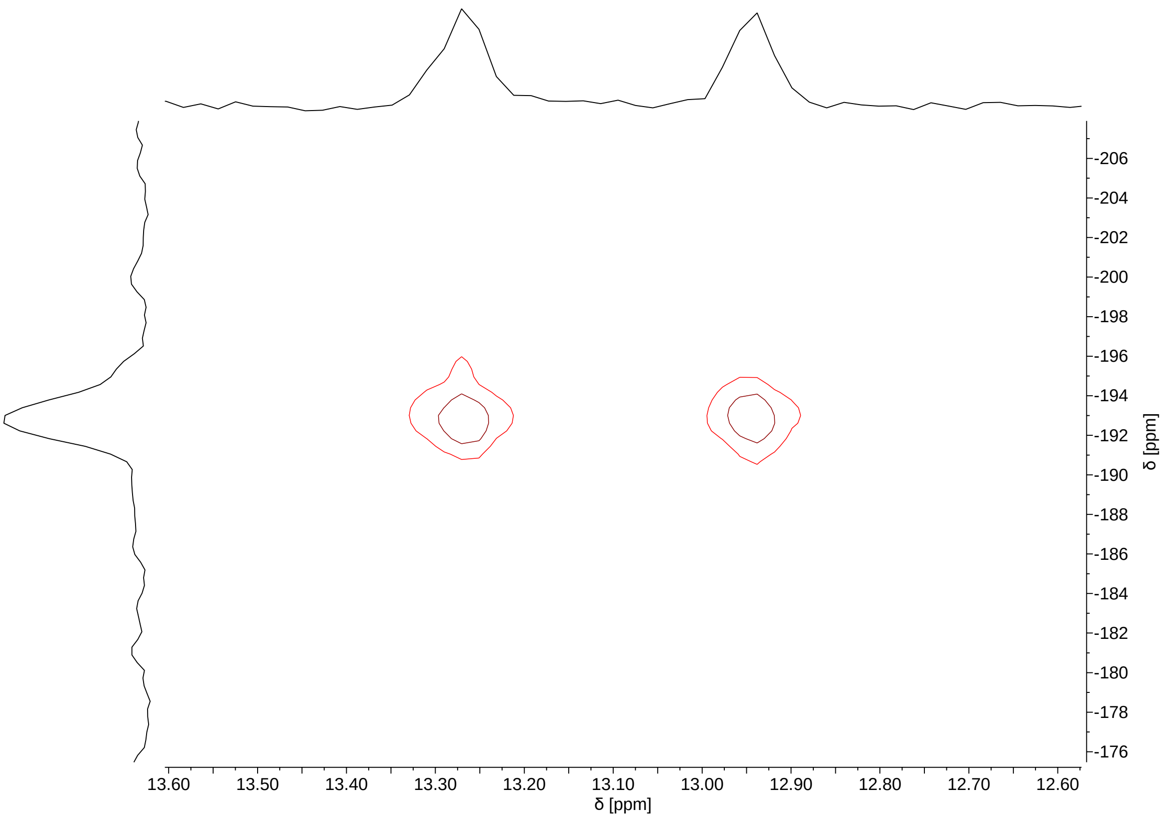

Figure S8. ^{1}H,^{15}N HMBC NMR spectrum of complex *cis*-[PdCl(P*i*Pr$_2$Im)(P*i*Pr$_2$Im-κ*P*,κ*N*^{1})$_2$]Cl (**7**)

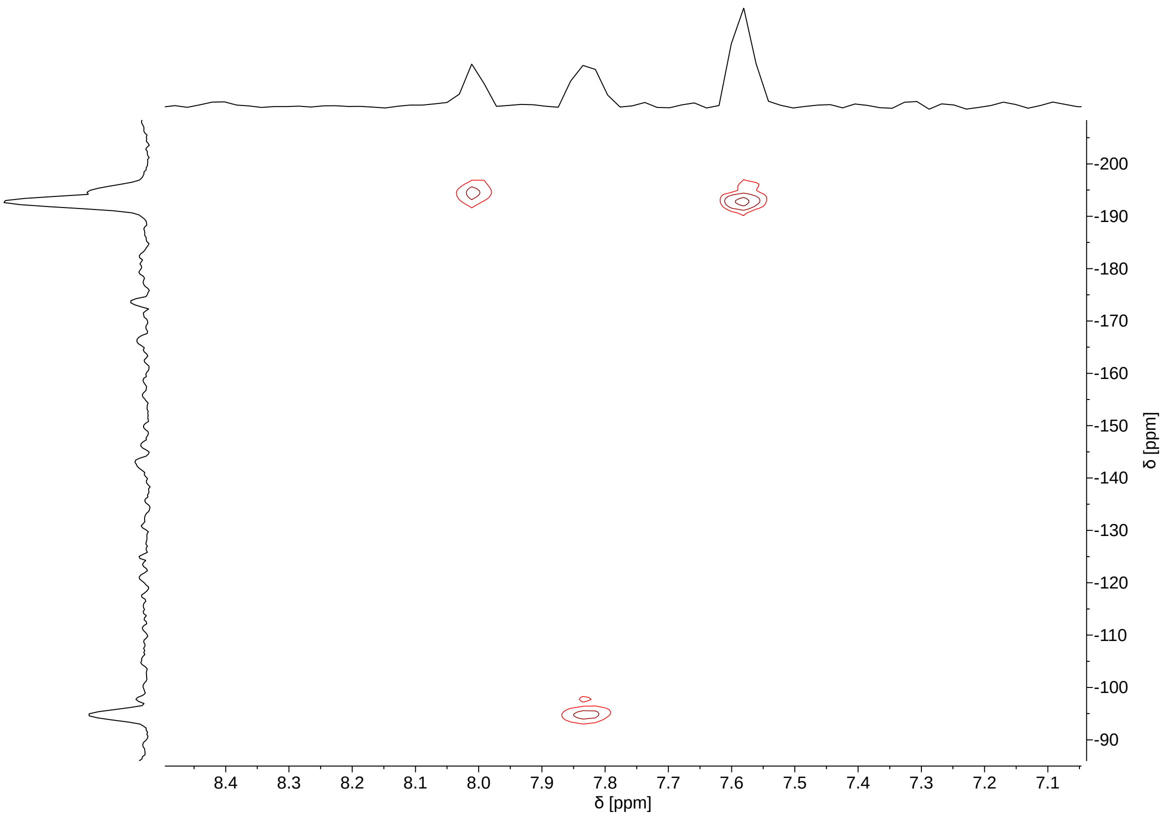

Figure S9. ^{1}H ^{15}N HMBC NMR spectrum of complex *cis*-[PdCl(P*i*Pr$_2$Im)(P*i*Pr$_2$Im-κ*P*,κ*N*^{1})$_2$]Cl (**7**)

1.4. Synthesis and characterization of *trans*-[$PdCl_2(PiPr_2Im^{Me})_2$] (Im^{Me} = 1-methyl-1H-imidazole) (**8**)

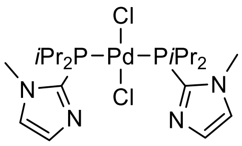

A suspension of [$PdCl_2$(COD)] (300 mg, 1.051 mmol) in DCM (10 mL) was treated with P*i*Pr_2Im^{Me} (417 mg, 2.102 mmol) and stirred for 1 h. The solvent was filtered off, and the residue was washed first with EtOH (1x2 mL), then with Et_2O (3x10 mL) and lastly with *n*-pentane (3x10 mL). All volatiles were removed *in vacuo* to yield **8** (415 mg, 0.721 mmol, 68%) as pale-yellow solid.

^{1}H NMR: (300 MHz, DCM-*d*$_2$) δ = 7.24 (s, 2H, Im-H), 7.09 (s, 2H, Im-H), 4.14 (s, 6H, N-CH_3), 3.06 (m, 4H, CH), 1.37-1.19 (m, 24H, *i*Pr-CH_3) ppm. **^{31}P NMR:** (121 MHz, DCM-*d*$_2$) δ = 24.23 (s) ppm; **ESI-MS** (+) (m/z) [M-Cl]$^+$ calculated for $C_{20}H_{38}ClN_4P_2Pd$, 537.1; found, 537.1. **Elemental analysis (%)** calculated for $C_{20}H_{38}Cl_2N_4P_2Pd$ (573.82): C 41.86, H 6.68, N 9.76; found: C 40.96, H 6.65, N 8.97.

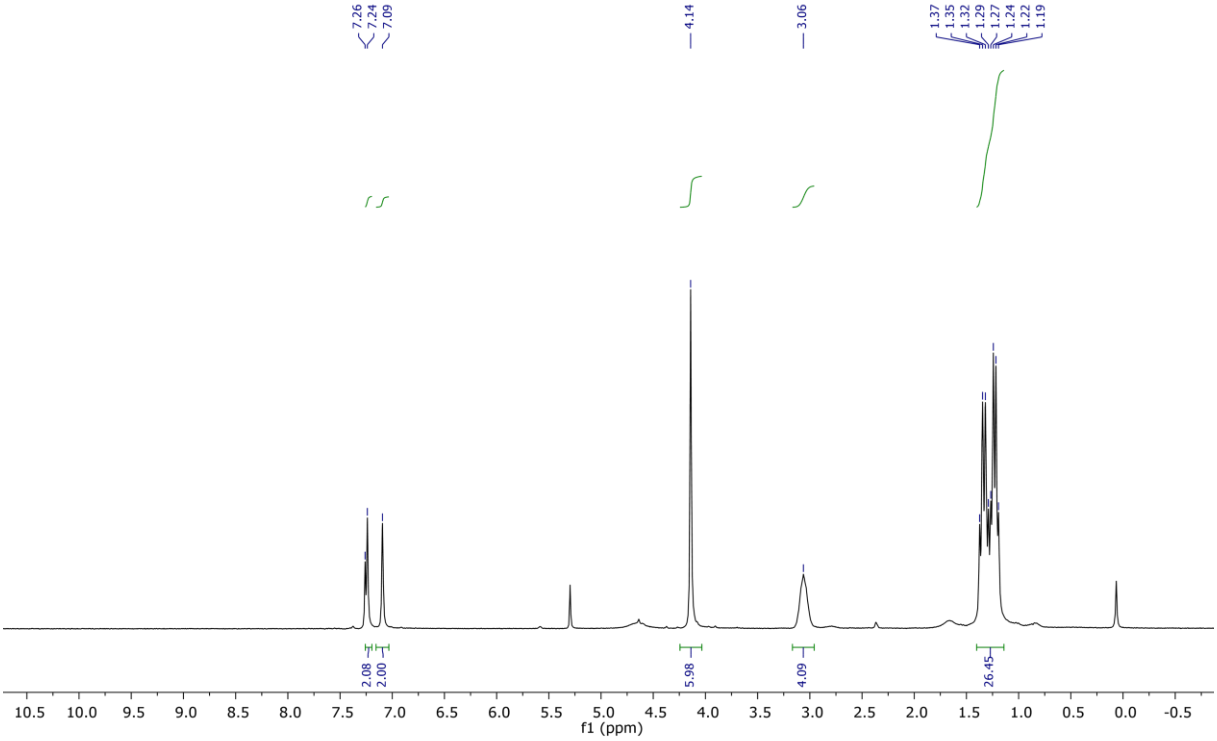

Figure S10. ^{1}H NMR spectrum of complex *trans*-[$PdCl_2(PiPr_2Im^{Me})_2$] (**8**) ($CDCl_3$, 300 MHz)

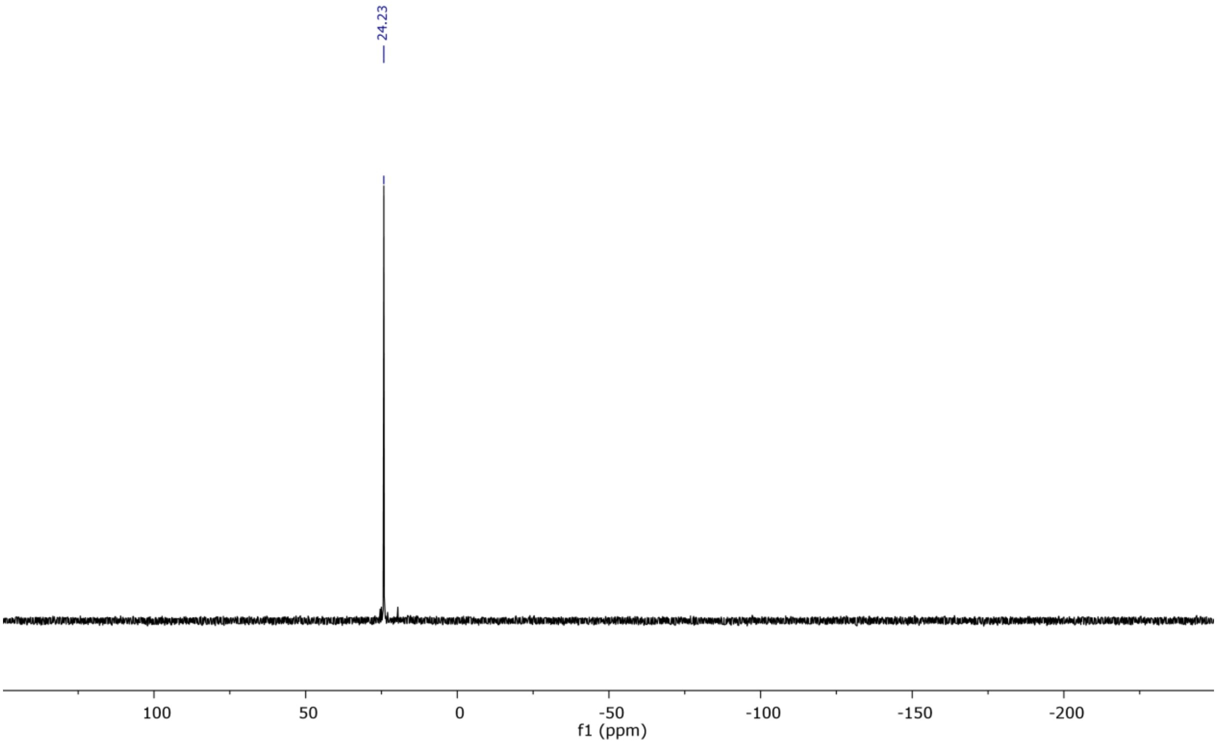

Figure S11. $^{31}P\{^1H\}$ NMR spectrum of complex *trans*-[$PdCl_2(PiPr_2Im^{Me})_2$] (**8**) ($CDCl_3$, 121 MHz)

1.5. Synthesis and characterization of *cis/trans*-[$PdCl_2(PPh_2py)_2$] (*cis/trans*-**9**)[14]

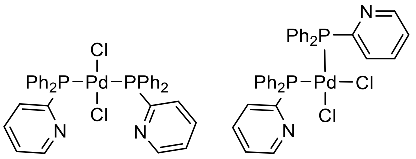

trans-**9** *cis*-**9**

A suspension of [$PdCl_2$(COD)] (300 mg, 1.051 mmol) in DCM (10 mL) was treated with PPh_2Py (554 mg, 2.102 mmol) and stirred for 1 h. The solvent was filtered off, and the residue was washed first with EtOH (1x2 mL), then with Et_2O (3x10 mL) and lastly with *n*-pentane (3x10 mL). All volatiles were removed *in vacuo* to yield cis/trans-**9** (630 mg, 0.895 mmol, 85%) as yellow solid.

^{31}P NMR: (121 MHz, DMSO-d$_6$): δ = 28.98 (s), 22.77 (s) ppm.

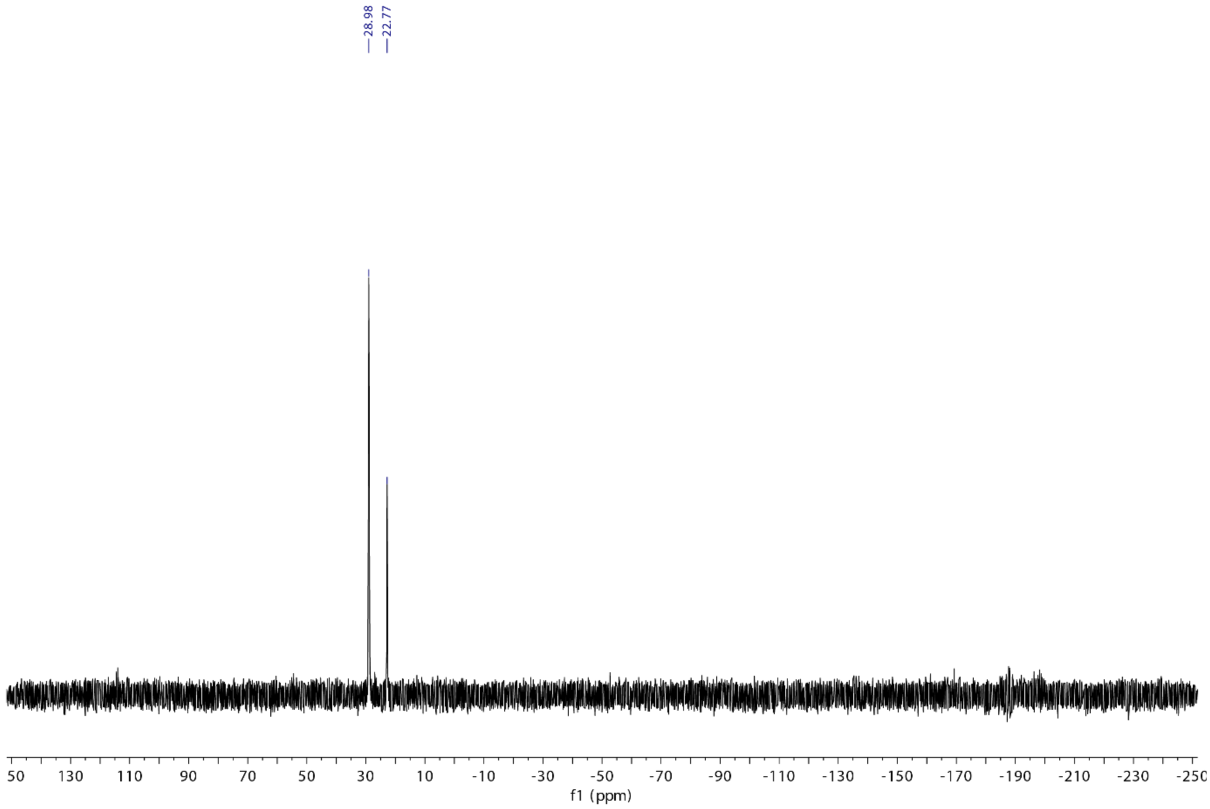

Figure S12. $^{31}P\{^1H\}$ NMR spectrum of complex *cis/trans*-$[PdCl_2(PPh_2py)_2]$ (**9**) (DCM-d_2, 121 MHz)

1.6 Synthesis and characterization of $[IrPdCl_3(CO)(P\textit{i}Pr_2Im)_2]$ (**4**)

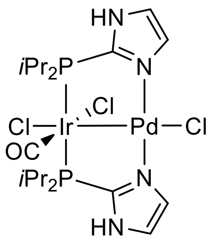

$[Ir(CO)(PiPr_2Im)_2]Cl$ (**1**) (609 mg, 0.976 mmol) and *cis*-$[PdCl_2(COD)]$ (279 mg, 0.976 mmol) were stirred in THF (10 mL) for 2 d. The solvent was filtered off and the residue was washed with THF (2x5 mL). After drying under high vacuum, **4** (279 mg, 0.763 mmol, 76%) was obtained as an orange solid. Orange single crystals were obtained by gas phase diffusion of *n*-pentane into a saturated DMSO solution of **4**.

^{1}H NMR: (300 MHz, DMSO-d_6): δ = 13.47 (s, br, 2H, NH), 7.54 (m, 2H, Im-H), 7.40 (m, 2H, Im-H), 3.26 (m, hept in $^1H\{^{31}P\}$ NMR spectrum, $^3J_{H,H}$ = 7 Hz, 2H, CH), 3.08 (m, hept in $^1H\{^{31}P\}$ NMR spectrum, $^3J_{H,H}$ = 7 Hz, 2H, CH), 1.68-1.52 (m, two d in $^1H\{^{31}P\}$ NMR spectrum, $^3J_{H,H}$ = 7 Hz, 12H, CH_3), 1.38-1.25 (m, two d in $^1H\{^{31}P\}$ NMR spectrum, $^3J_{H,H}$ = 7 Hz, 12H, CH_3) ppm. **^{31}P NMR:** (121 MHz, DMSO-d_6) δ = -0.76 (s) ppm; **IR (ATR):** $\tilde{\nu}$ = 3091 (br, NH), 2000 (s, CO) cm^{-1}. **ESI-MS** (+) (m/z) $[M-Cl]^+$ calculated for $C_{19}H_{36}Cl_2IrN_4OP_2Pd$, 767.0; found, 767.0. **Elemental analysis (%)** calculated for $C_{19}H_{34}Cl_3IrN_4OP_2Pd$ (801.44): C 28.47, H 4.28, N 6.99; found: C 28.67, H 4.45, N 6.91.

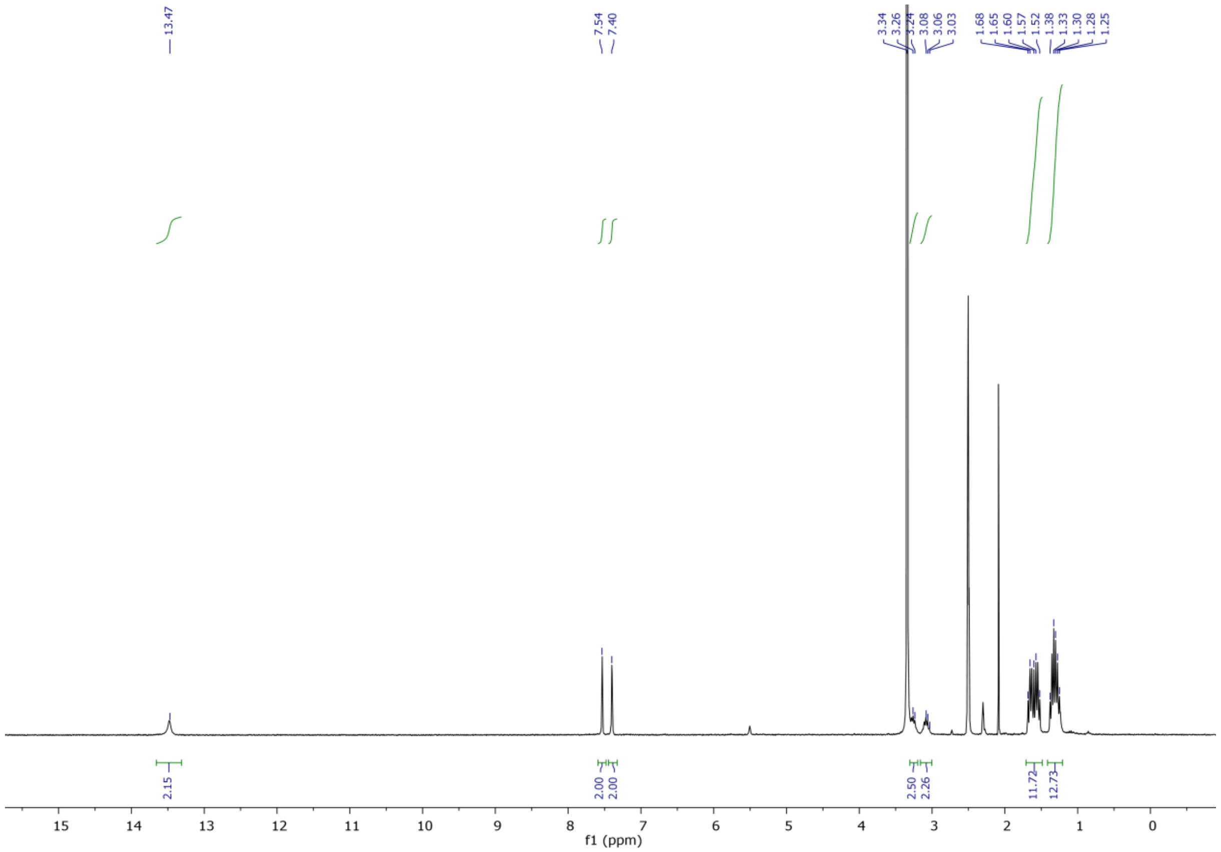

Figure S13. ^{1}H NMR spectrum of complex $[IrPdCl_3(CO)(PiPr_2Im)_2]$ (**4**) (DMSO-d_6, 300 MHz)

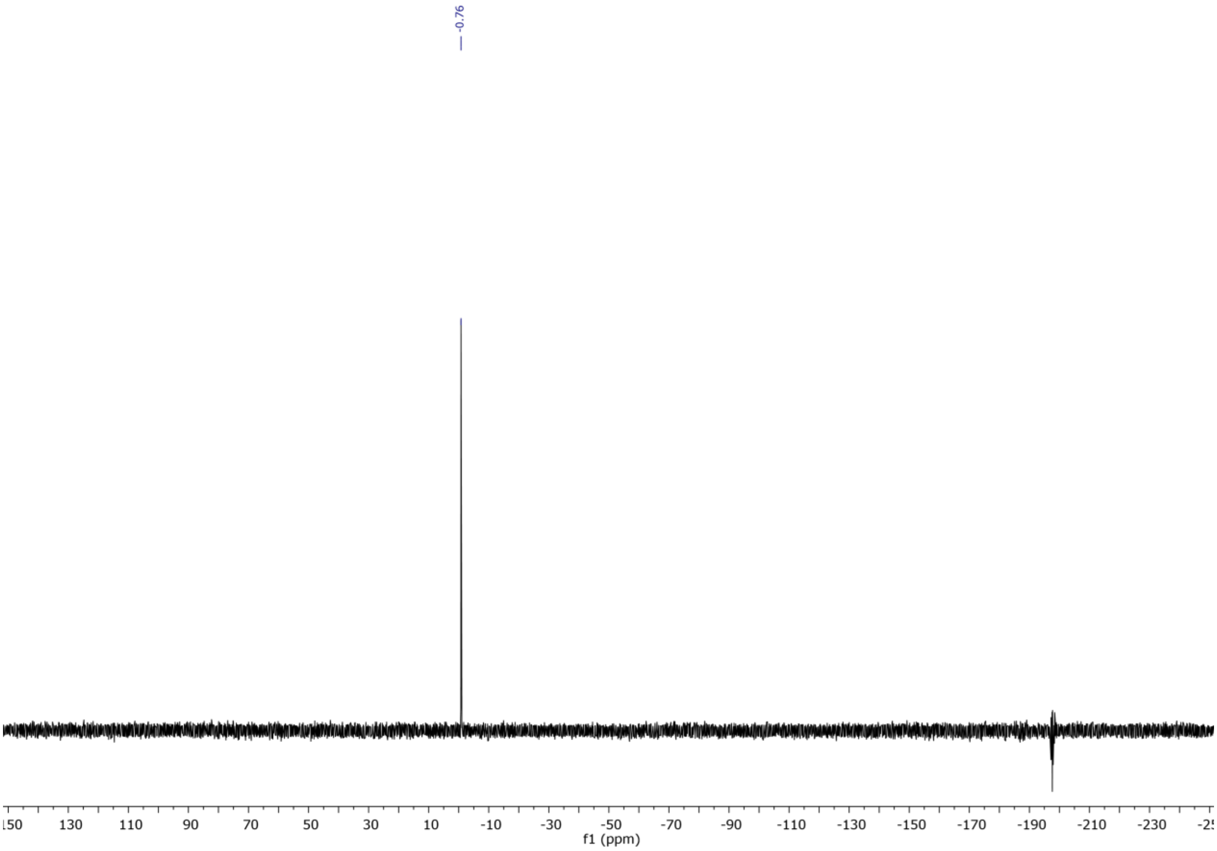

Figure S14. $^{31}P\{^{1}H\}$ NMR spectrum of complex $[IrPdCl_3(CO)(PiPr_2Im)_2]$ (**4**) (DMSO-d_6, 121 MHz)

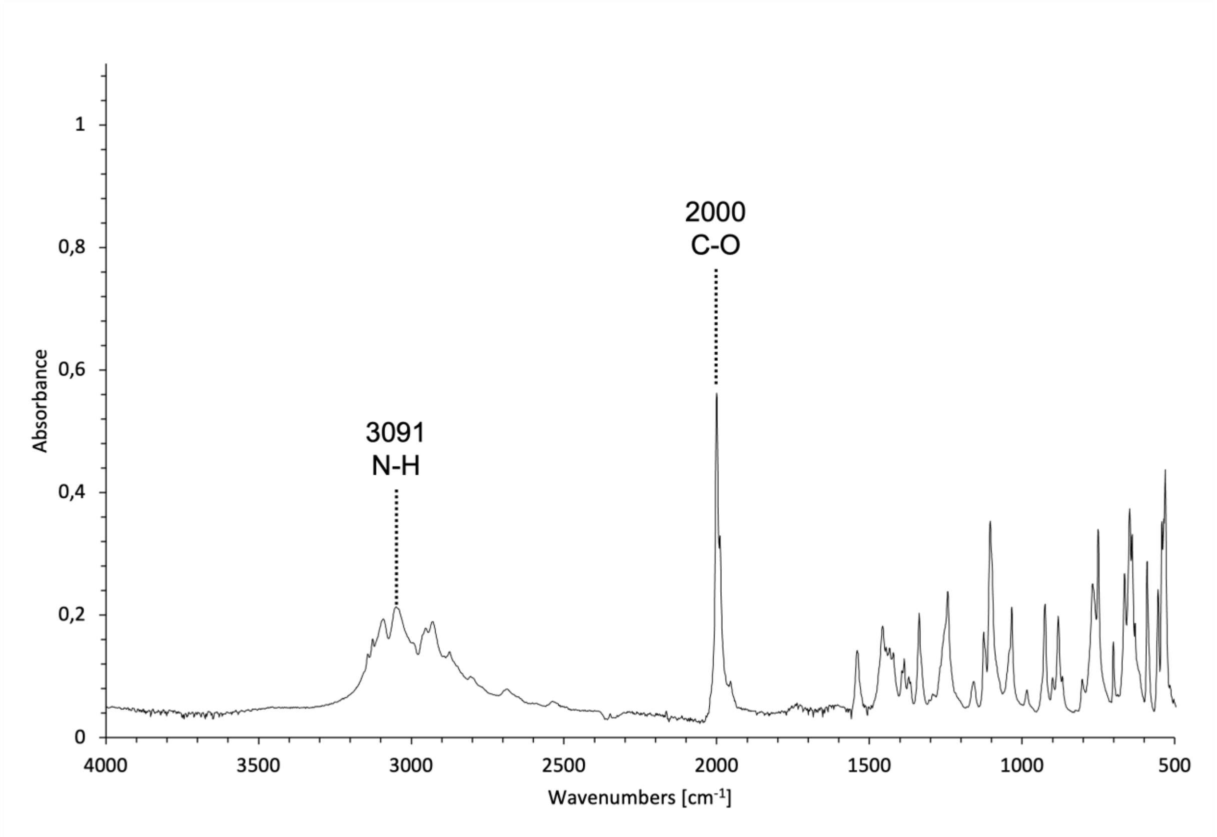

Figure S15. IR spectrum of complex $[IrPdCl_3(CO)(PiPr_2Im)_2]$ (**4**)

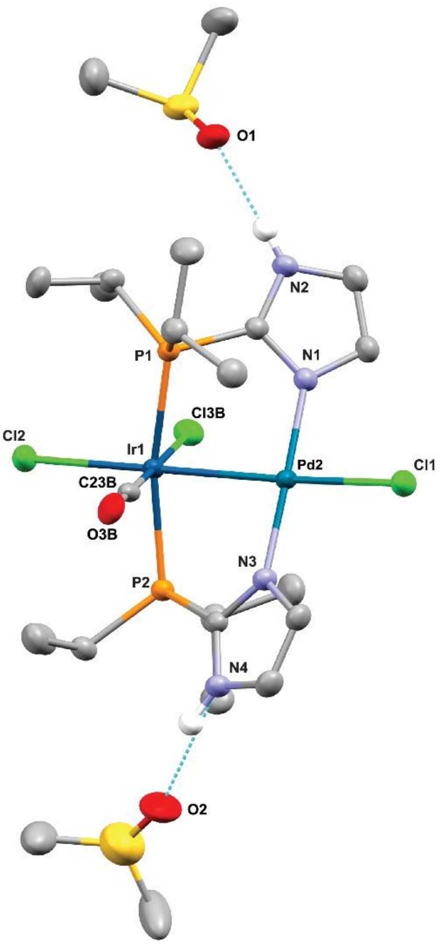

Figure S16. Molecular structure of complex [$IrPdCl_3$(CO)(P*i*Pr_2Im)$_2$].DMSO (**4** · 2 DMSO)

1.7 Synthesis and characterization of [$IrPdCl_3$(CO)(P*i*Pr_2Im^{Me})$_2$] (Im^{Me} = 1-methyl-1H-imidazole) (**5**)

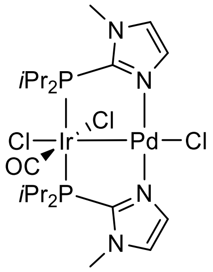

[Ir(CO)(P*i*Pr_2Im^{Me})$_2$]Cl (**2**) (200 mg, 0.307 mmol) and *cis*-[$PdCl_2$(COD)] (88 mg, 0.157 mmol) were stirred in THF (10 mL) for 2 days. The solvent was removed under high vacuum, and the residue was washed first with EtOH (2x3 mL) and then with Et_2O (2x3 mL). After drying under high vacuum, **5** (130 mg, 0.157 mmol, 51%) was obtained as an orange solid.

^{1}H NMR: (300 MHz, DMSO-d$_6$): δ = 7.54 (m, 2H, Im-CH), 7.34 (m, 2H, Im-CH), 3.88 (s, 6H, N-CH_3), 3.41 (m, 2H, CH), 3.20 (m, 2H, CH), 1.76-1.63 (m, two doublets in ^{1}H{^{31}P} NMR spectrum, $^3J_{H,H}$ = 7 Hz, 12H, CH_3), 1.47-1.32 (m, two doublets in ^{1}H{^{31}P} NMR Spectrum, $^3J_{H,H}$ = 7 Hz, 12H, CH_3) ppm.**^{31}P NMR:** (121 MHz, DMSO-d$_6$): δ = 10.54 (s) ppm; **IR (ATR)**: $\tilde{\nu}$ = 1950 (s, CO) cm^{-1}. **ESI-MS** (+) (m/z) [M-Cl_2]$^+$

calculated for $C_{21}H_{40}ClIrN_4OP_2Pd$, 760.0; found, 759.1; $[M-Cl]^+$ calculated for $C_{21}H_{40}Cl_2IrN_4OP_2Pd$, 795.0; found, 795.0. **Elemental analysis (%)** calculated for $C_{27}H_{60}ClIrOP_4$ (752.34): C 30.41, H 4.62, N 6.75; found: C 30.99, H 4.72, N 6.25.

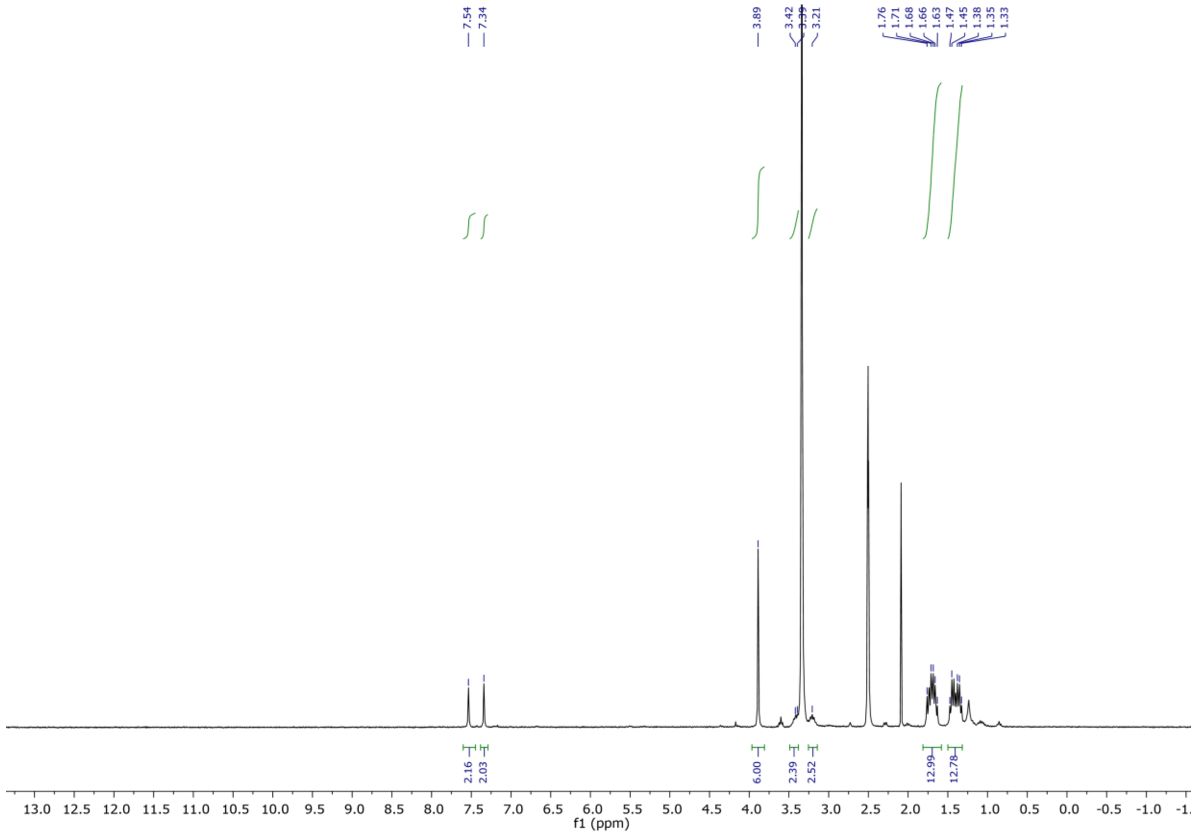

Figure S17. ^{1}H NMR spectrum of complex [IrPdCl$_3$(CO)(P*i*Pr$_2$Im^{Me})$_2$] (**5**) (DMSO-d$_6$, 300 MHz)

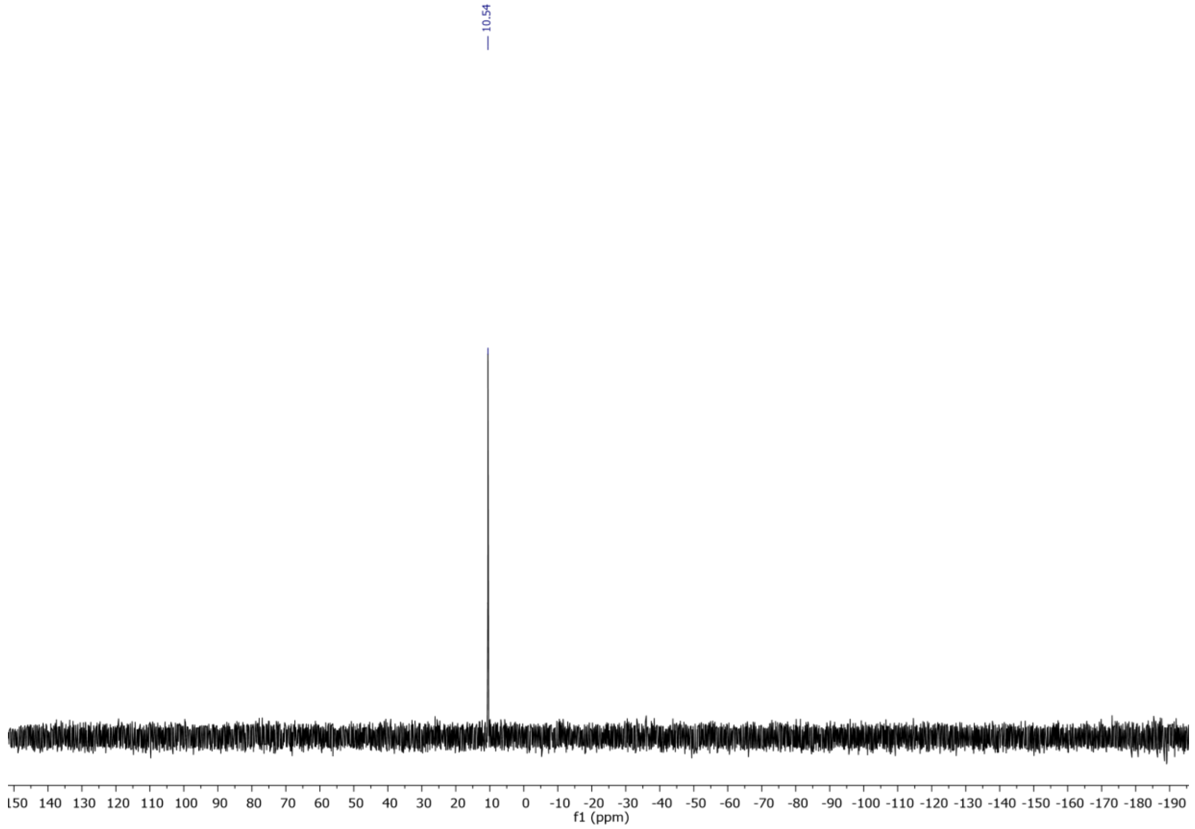

Figure S18. $^{31}P\{^{1}H\}$ NMR spectrum of complex $[IrPdCl_3(CO)(P\textit{i}Pr_2Im^{Me})_2]$ (**5**) (DMSO-d_6, 121 MHz)

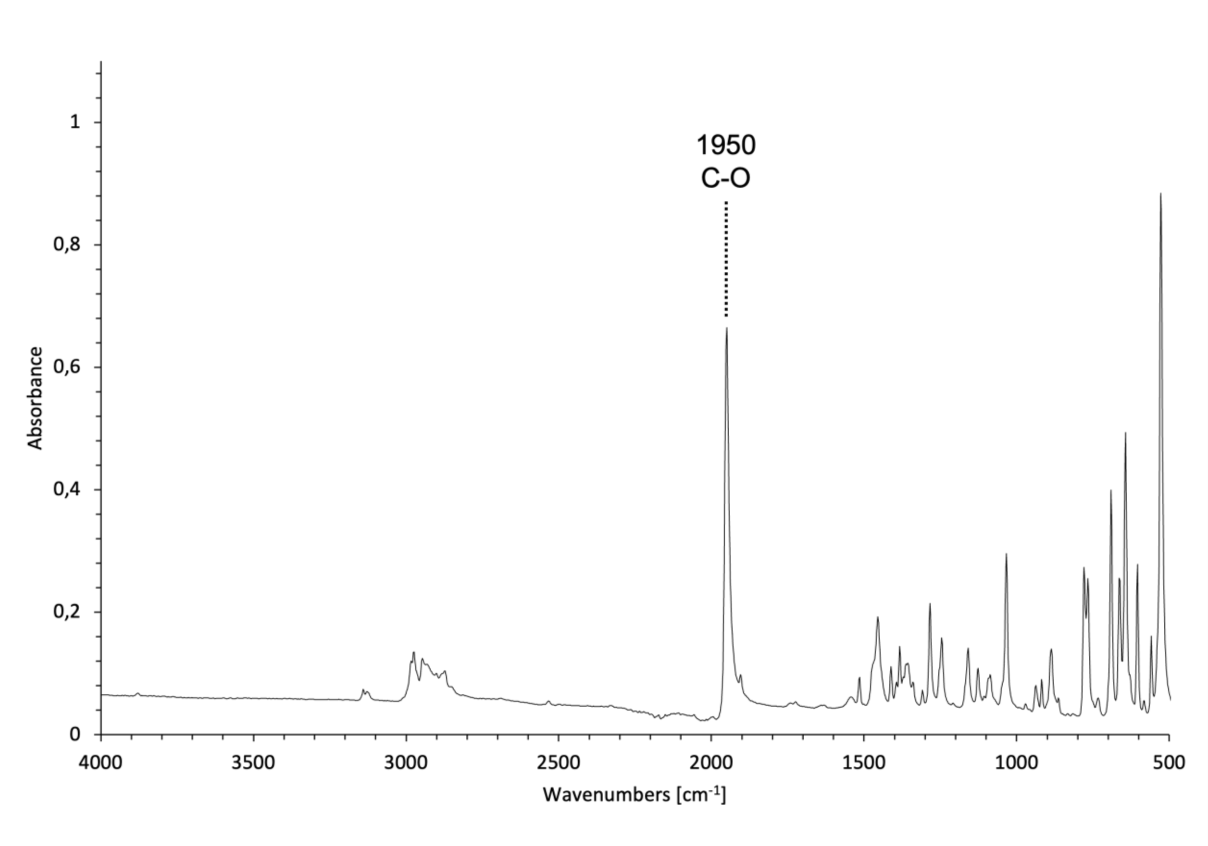

Figure S19. IR spectrum of complex $[IrPdCl_3(CO)(P\textit{i}Pr_2Im^{Me})_2]$ (**5**)

1.8 Synthesis and characterization of [IrPdCl$_3$(CO)(PPh$_2$py)$_2$] (**6**) (New synthetic route) [5]

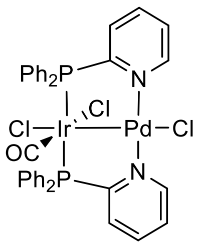

[IrCl(CO)(PPh$_2$py)$_2$] (**3**) (300 mg, 0.383 mmol) and *cis*-[PdCl$_2$(COD)] (109 mg, 0.383 mmol) were stirred in toluene (10 mL) for 1 d. The solvent was removed in high vacuum and the residue was washed first with EtOH (2x3 mL) and then with *n*-pentane (2x3 mL). After drying under high vacuum, **6** (304 mg, 0.318 mmol, 83%) was obtained as an orange solid.

^{31}P NMR (121 MHz, DCM-d$_2$): δ = -12.50 (s) ppm; IR **(ATR)**: $\tilde{\nu}$ = 2013 (s, CO) cm^{-1}.

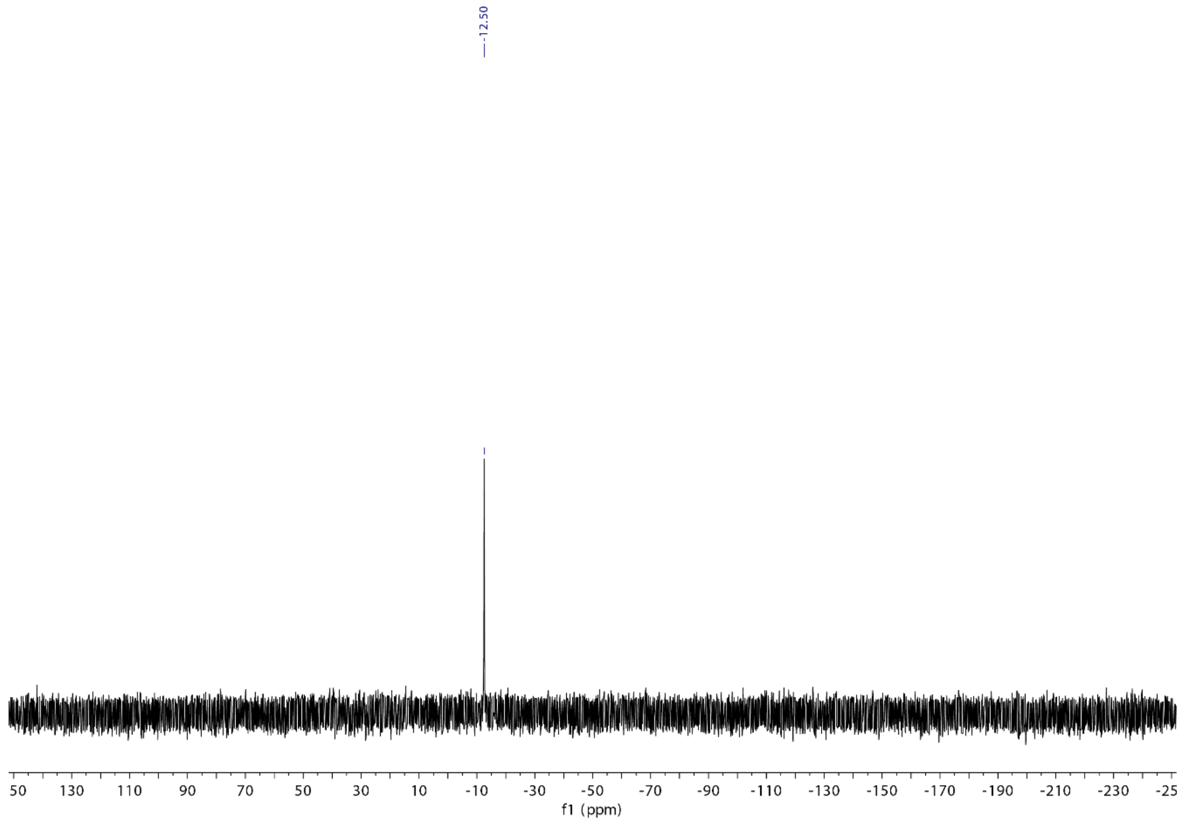

Figure S20. ^{31}P{^{1}H} NMR spectrum of complex [IrPdCl$_3$(CO)(PPh$_2$Py)$_2$] (**6**) (DCM-d$_2$, 121 MHz)

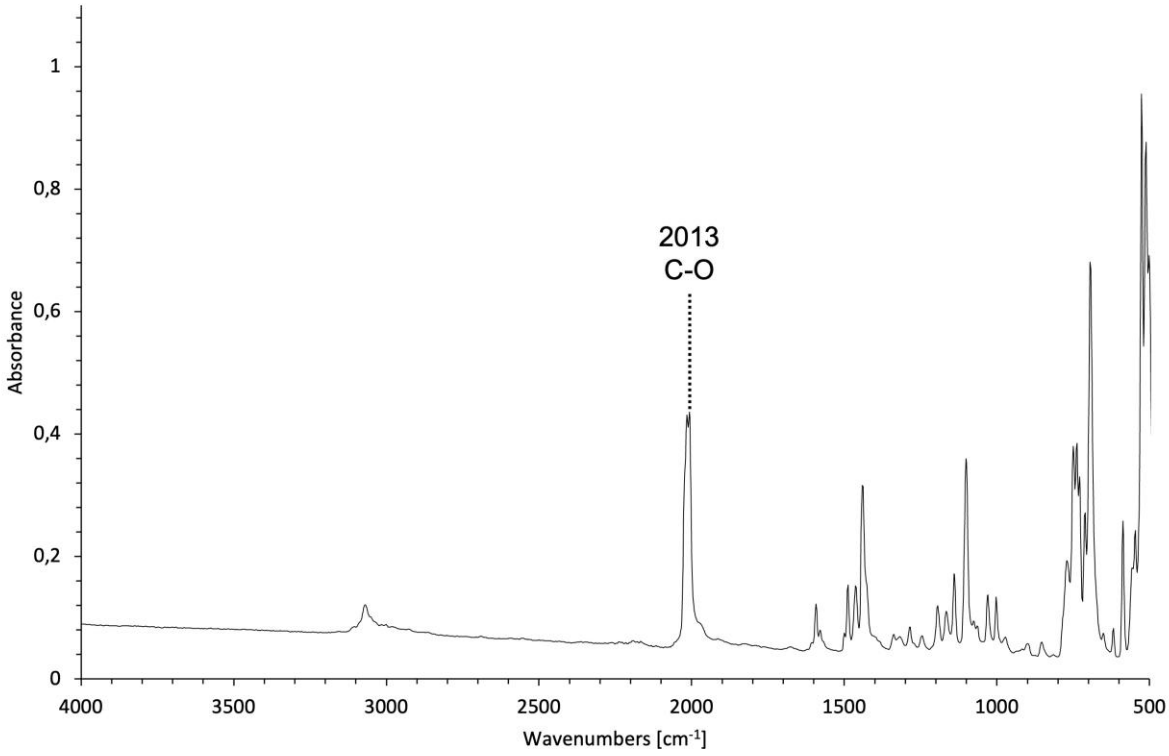

Figure S21. IR spectrum of complex [$IrPdCl_3(CO)(PPh_2py)_2$] (**6**)

1.9 General procedure for the *N*-Alkylation of aniline with methanol.

An oven dried Schlenk flask was charged with aniline (1.0 mmol), methanol (0.2 ml), KO*t*Bu (1.5 mmol), and catalyst (0.5 mol %). The mixture was stirred to 100 °C for 4h under air atmosphere. The reaction mixture was allowed to cool to room temperature, filtered, and analyzed using GC-MS with mesitylene (10 µL) as the internal standard.

1.10 General procedure for the one-pot aminocarbonylation of primary amines

In a 50 ml young-flask aniline (1.0 mmol), methanol (0.2 ml), KO*t*Bu (1.5 mmol), and catalyst (1 mol %) were added. The mixture was stirred to 100 °C for 4h under air atmosphere. Then, without any further isolation or workup, toluene (3 ml), aryl iodide (1.2 mmol) and K_2CO_3 (2.0 mmol) were added and the flask was putted under atmospheric pressure of CO to be stirred at 100 °C for 15h. The reaction mixture was passed through a short column eluting with diethyl ether to remove the catalyst and inorganic materials and analyzed by means of GC-MS and by using mesitylene as the internal standard.

1.11 Methanol dehydrogenation with **5**

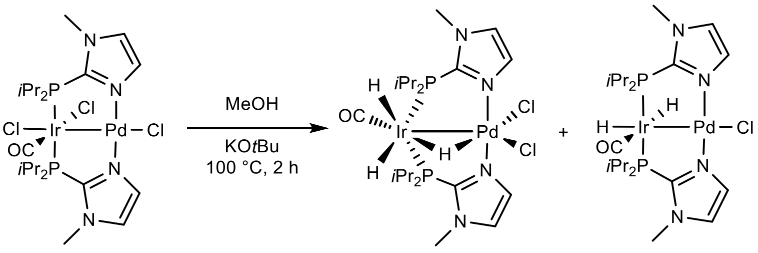

5 5H$_3$ 5H$_2$

5 (40 mg, 0.048 mmol) and KO*t*Bu (13 mg, 0.117 mmol) were added to a Schlenk flask. Then 0.8 ml of MeOH were added to give immediately a dark orange suspension. The suspension was stirred for 2 hours at 100°C and then allowed to cool to room temperature. The brown solution was filtered through celite, and the filtrate contained a methanol solution of **5H$_3$** and **5H$_2$**, and was used for further analysis. However, putting the filtrate under vacuum and further work-up led to the decomposition of the complexes according to ^{1}H NMR data. The intermediates in solution remained stable overnight. The isomers **5H$_3$** (28%, 0.013 mmol) and **5H$_2$** (17%, 0.008 mmol) are in a 1.6:1 ratio based on the ^{1}H NMR data. In a catalytic reaction both isomers are present in the solution. **^{1}H NMR** for **5H$_3$** (300 MHz, CD$_3$OD) δ = -11.97 (td, $^2J_{H,P}$ = 14.7, $^2J_{H,H}$ = 4.8, 2H), -13.07 (tt, $^2J_{H,P}$ = 19.6, $^2J_{H,H}$ = 4.8, 1H); **^{1}H NMR** for **5H$_2$** (300 MHz, CD$_3$OD) δ = -8.91 (td, $^2J_{H,P}$ = 18.5, 5.2, 1H), -20.43 (td, $^2J_{H,P}$ = 13.0, $^2J_{H,H}$ = 5.2, 1H); **^{1}H{^{31}P} NMR** for **5H$_3$** (300 MHz, CD$_3$OD) δ = -11.97 (d, $^2J_{H,H}$ = 4.8 Hz, 2H), -13.07 (t, $^2J_{H,H}$ = 4.8 Hz, 1H); **^{1}H{^{31}P} NMR** for **5H$_2$** δ = -8.91 (d, $^2J_{H,H}$ = 5.2, 1H), -20.43 (d, $^2J_{H,H}$ = 5.2, 1H); **^{1}H,^{1}H COSY NMR** (300 MHz/300 MHz, CD$_3$OD): δ = -8.91/-20.43 (td/td), -11.97/-13.07 (td/tt); **^{31}P NMR** for **5H$_3$** (121 MHz, CD$_3$OD) δ = 150.62 (s). **^{31}P NMR** for **5H$_2$** (121 MHz, CD$_3$OD) δ = 138.58 (s) ppm; **IR (ATR)** for **5H$_3$**: $\tilde{\nu}$ = 1997 (s, Ir-CO), 2056 (s, Ir-H), 2124 (s, Ir-H) cm^{-1}.

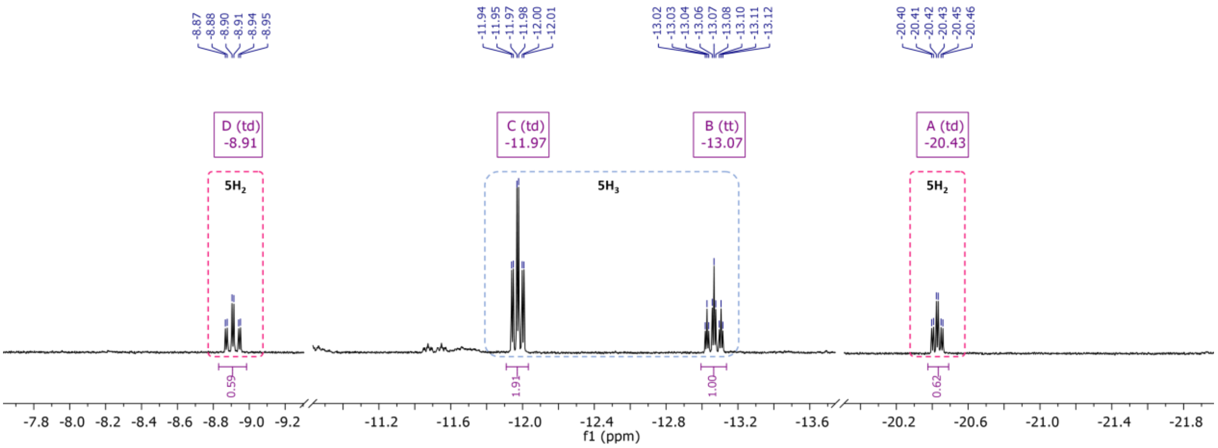

Figure S22. hydride region of ^{1}H NMR of methanol dehydrogenation by **5** in CD$_3$OD

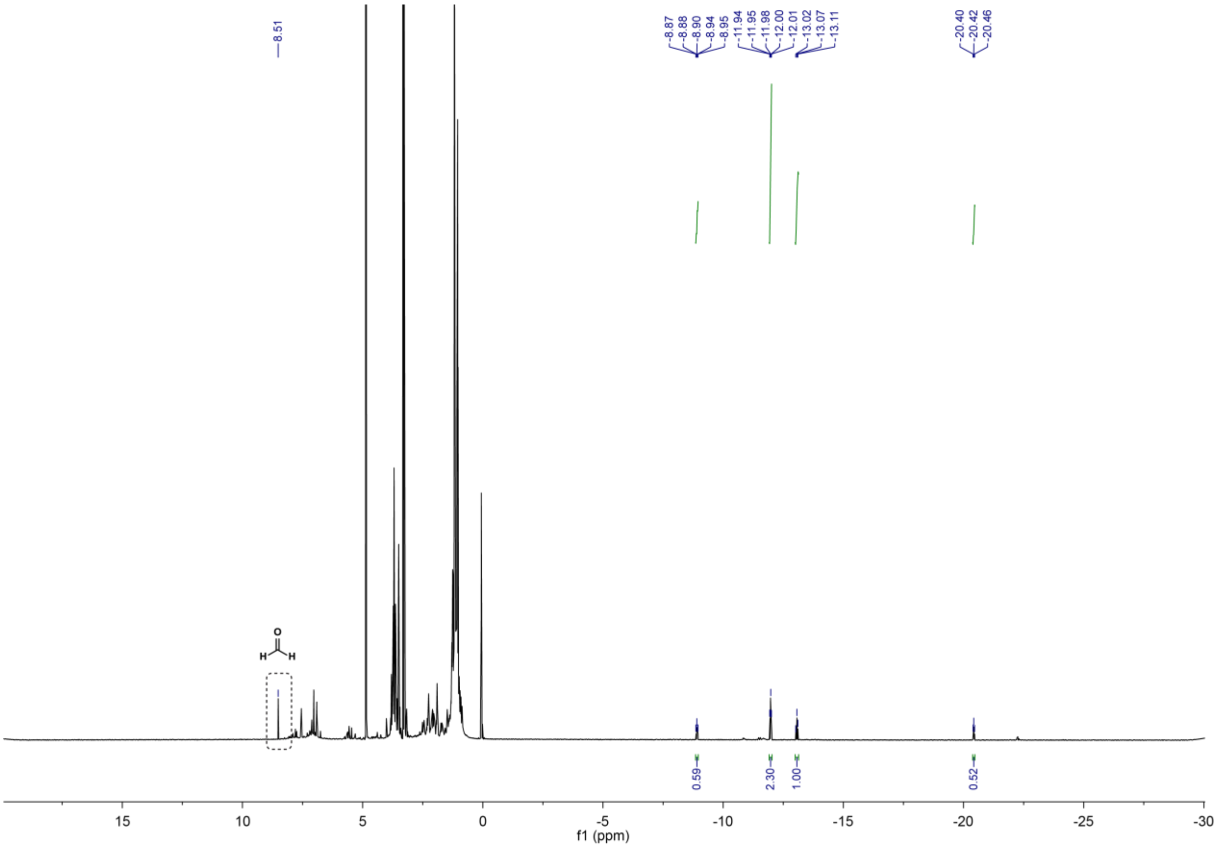

Figure S23. ^{1}H NMR spectrum of methanol dehydrogenation by **5** in CD_3OD

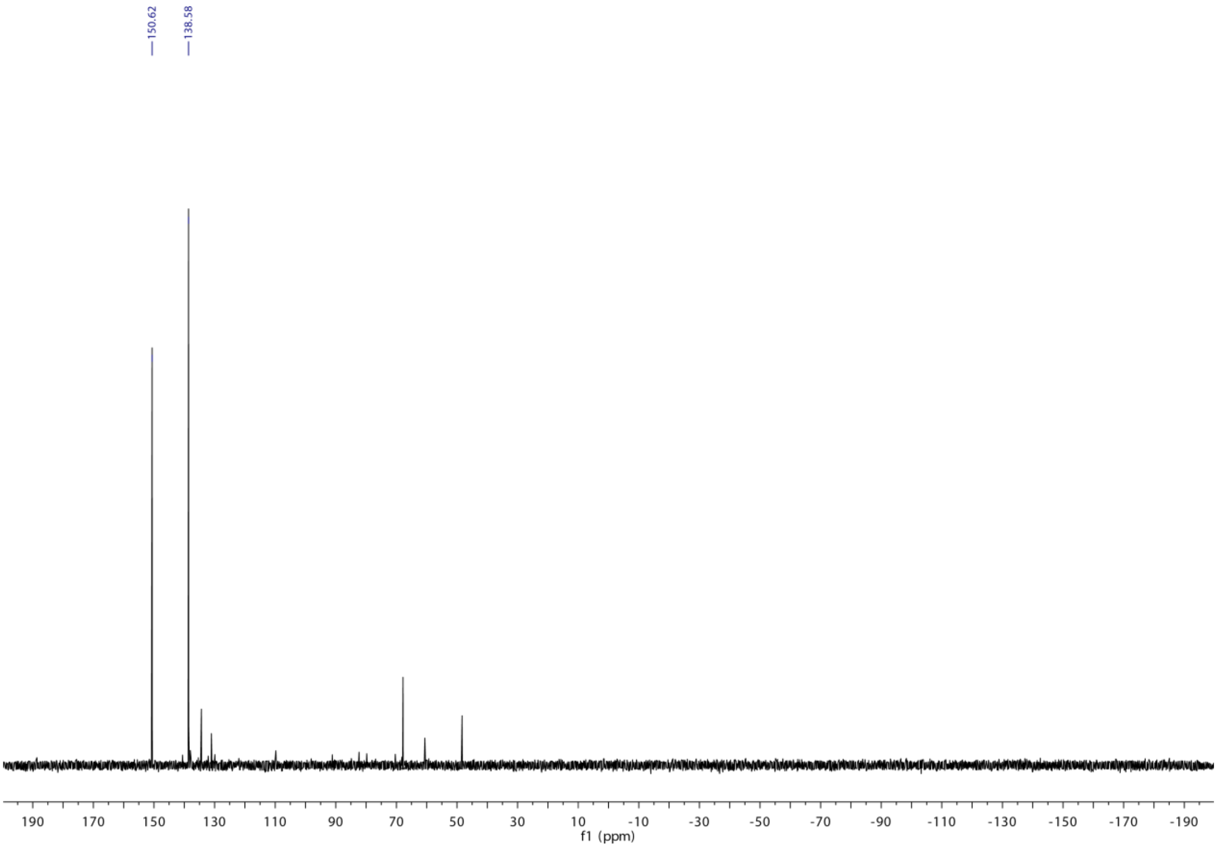

Figure S24. $^{31}P\{^{1}H\}$ NMR spectrum of methanol dehydrogenation by **5** in CD_3OD

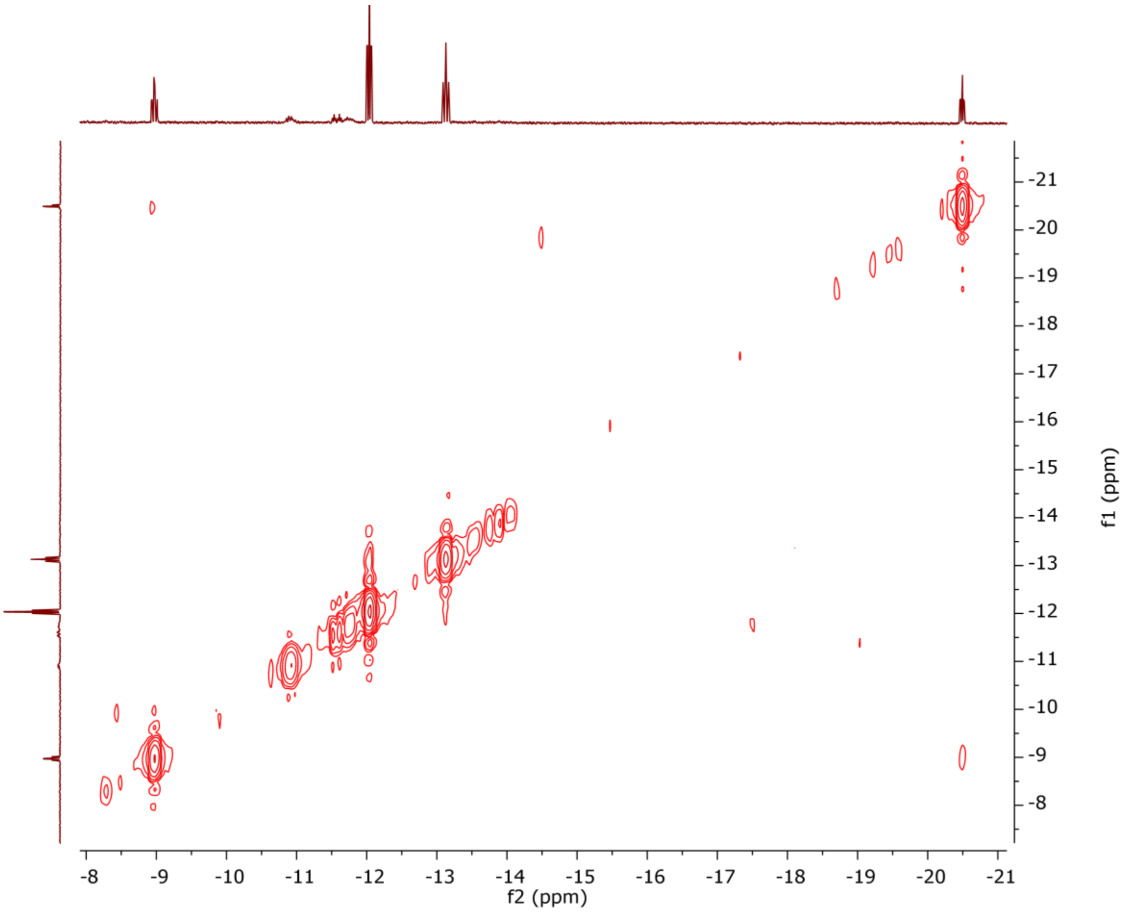

Figure S25. $^1H,^1H$ COSY NMR spectrum of methanol dehydrogenation by **5** in CD_3OD

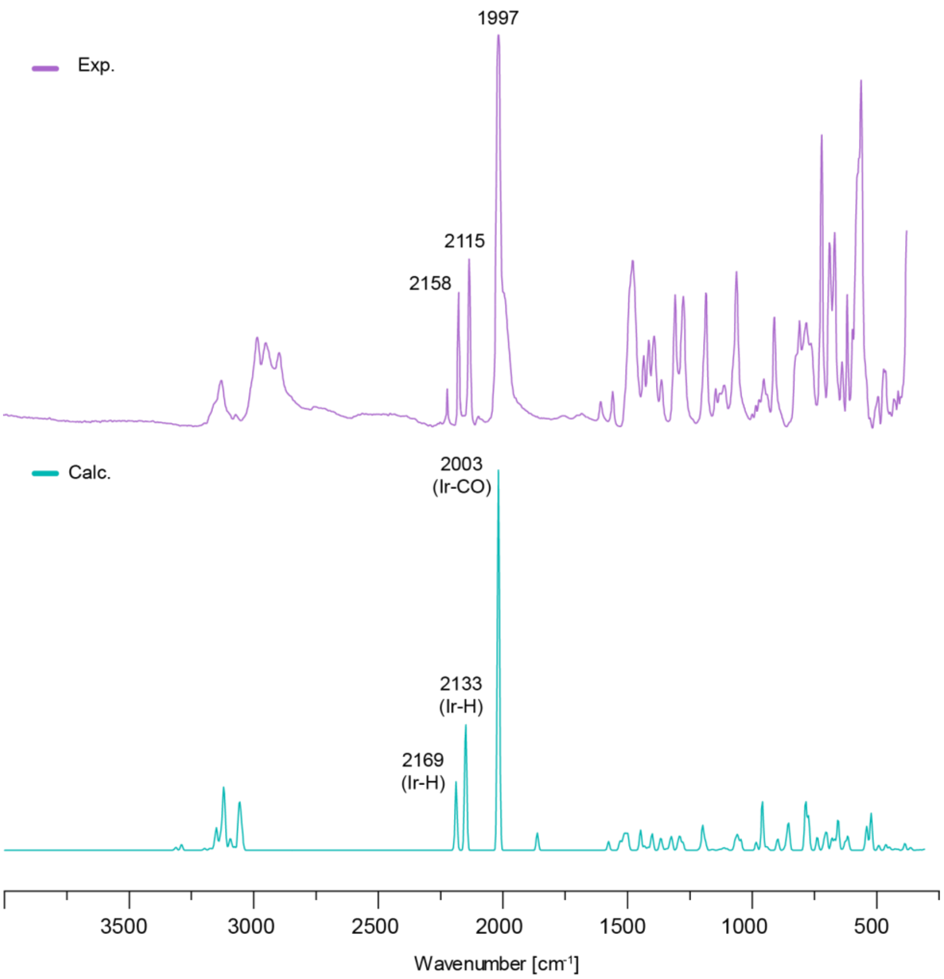

Figure S26. IR spectra comparison for **$5H_3$**

1.12 Monitoring the *N*-mehtylation process

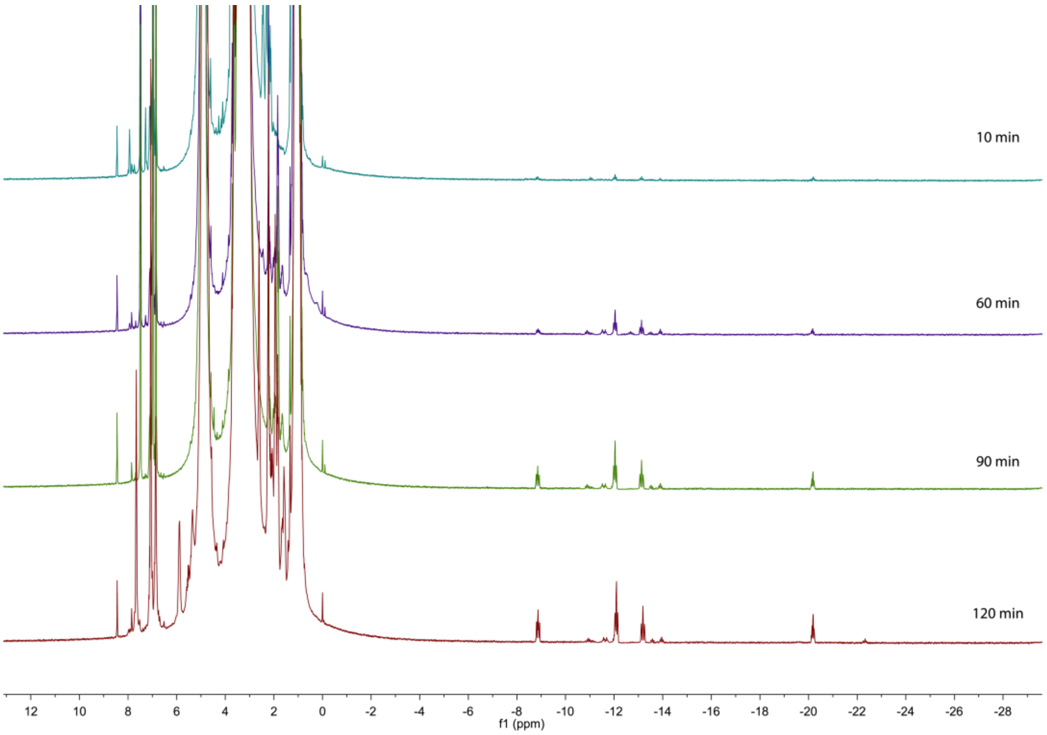

Figure S27. ^{1}H NMR spectra monitoring the formation of **$5H_3$** and **$5H_2$** during the *N*-methylation of aniline at 100 °C in CD_3OD

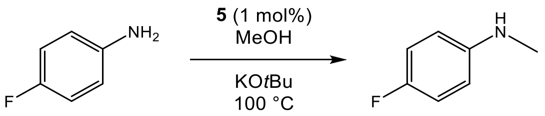

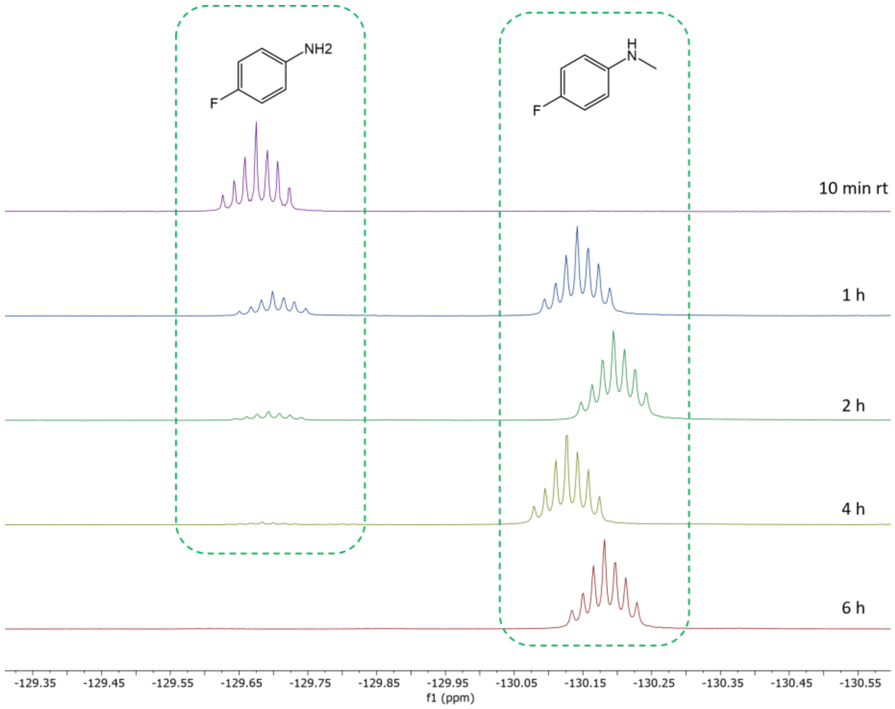

Figure S28. ^{19}F NMR spectra **m**onitoring of the *N*-methylation reaction of 4-fluoroaniline in DMSO-d_6

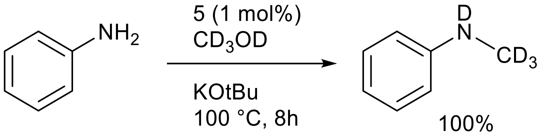

Scheme S1. Deuterium labeling experiment in the *N*-methylation of aniline catalyzed by **5** in CD_3OD with optimized condition (as described in Section 1.9, but run with deuterated methanol).

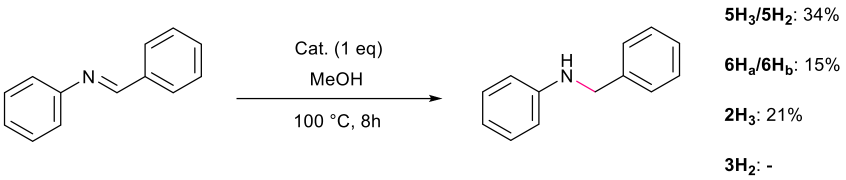

Scheme S2. Hydrogenation of imine bond in *N*-benzylideneaniline

In a 10 ml young-flask, *N*-benzylideneaniline (0.045 mmol), catalyst (0.045 mmol) and methanol (0.5 ml) were heated at 100 °C for 8 hours. The reaction mixture was allowed to cool to room temperature, filtered, and analyzed using GC-MS with mesitylene as the internal standard.

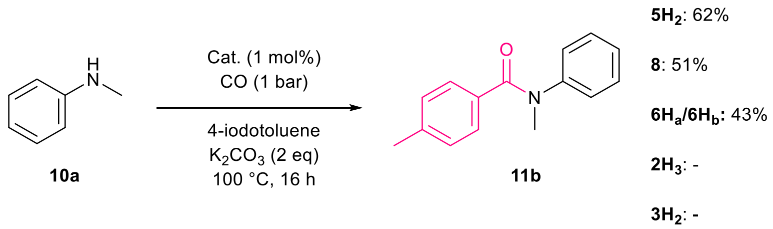

Scheme S3. Aminocarbonylation of *N*-methylaniline catalyzed by hydride complexes under optimized conditions (similar to the procedure described in section 1.10).

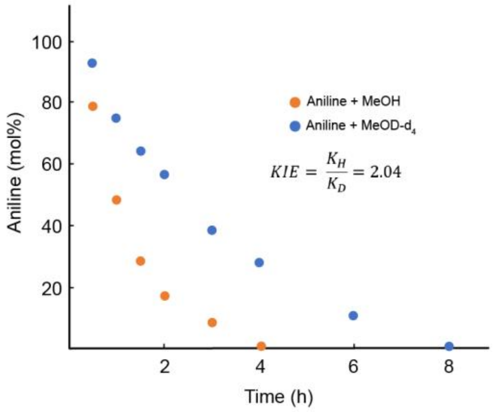

Figure S29. Kinetic isotope effect (KIE) for the *N*-methylation of aniline using methanol and methanol-d_4, monitored via GC-MS with mesitylene as an internal standard. Aniline (1 mmol), KO*t*Bu (1.5 mmol), **5** (0.5 mol%) and MeOH or MeOD-d_4 (0.8 ml) at 100 °C

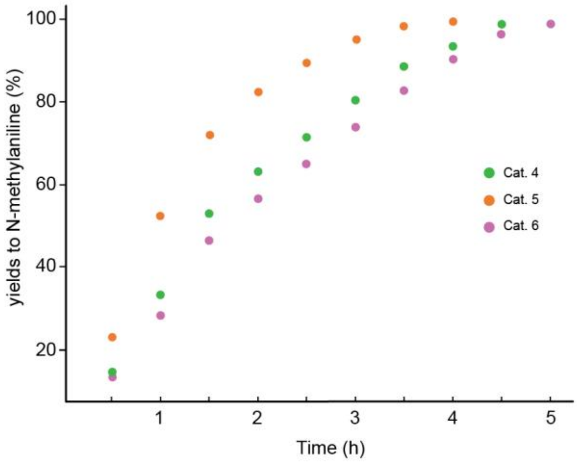

Figure S30. The profiles for the formation of *N*-methylaniline for the catalysts **4**, **5**, and **6** in the *N*-methylation of aniline with methanol under optimized condition.

1.13 Independent synthesis of **5H2**

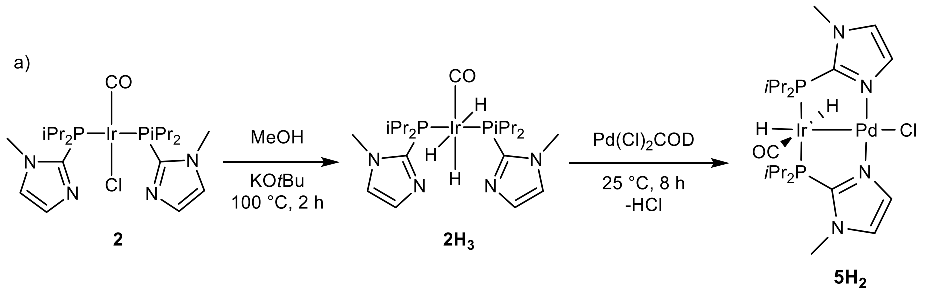

A methanol solution (1 ml) of **2** (35 mg, 0.054 mmol) in the presence of KO*t*Bu (13 mg, 0.117 mmol) was heated up to 100 °C for 2 hours until the formation of a clear orange solution. After cooling down to room temperature 0.1 ml of the solution was transferred into a NMR tube and the solvent was evaporated. The remaining orange liquid containing compound **2H3** was then analysed by NMR spectroscopy. Subsequently, 1 eq of the [$PdCl_2$(COD)] (15 mg, 0.054 mmol) was added to the main methanol solution containing **2H3** and the mixture was stirred at room temperature for 8 hours, yielding **5H2** (51%) in a brownish methanol solution. Both compounds **2H3** and **5H2** are stable only in methanol solution overnight.

Analytical data for **2H3**: **^{1}H NMR** (300 MHz, CD_3OD) δ = -11.91 (td, $^2J_{H,P}$ = 14.7, $^2J_{H,H}$ = 4.8, 2H), -12.99 (tt, $^2J_{H,P}$ = 19.6, $^2J_{H,H}$ = 4.9, 1H); **^{31}P NMR** (121 MHz, CD_3OD) 117.82 (s) ppm; **IR (ATR)**: $\tilde{\nu}$ = 1787, 1958, 2079 cm^{-1}.

Analytical data for **5H2**: **^{1}H NMR** (300 MHz, CD_3OD) *δ* = -8.91 (td, $^2J_{H,P}$ = 18.5, $^2J_{H,H}$ = 5.2, 1H), -20.45 (td, $^2J_{H,P}$ = 13.1, $^2J_{H,H}$ = 5.2, 1H); **^{31}P NMR** (121 MHz, CD_3OD) 138.60 (s) ppm; **IR (ATR)**: $\tilde{\nu}$ = 1947 (s, Ir-H), 1982 (s, Ir-CO), 2097 (s, Ir-H) cm^{-1}.

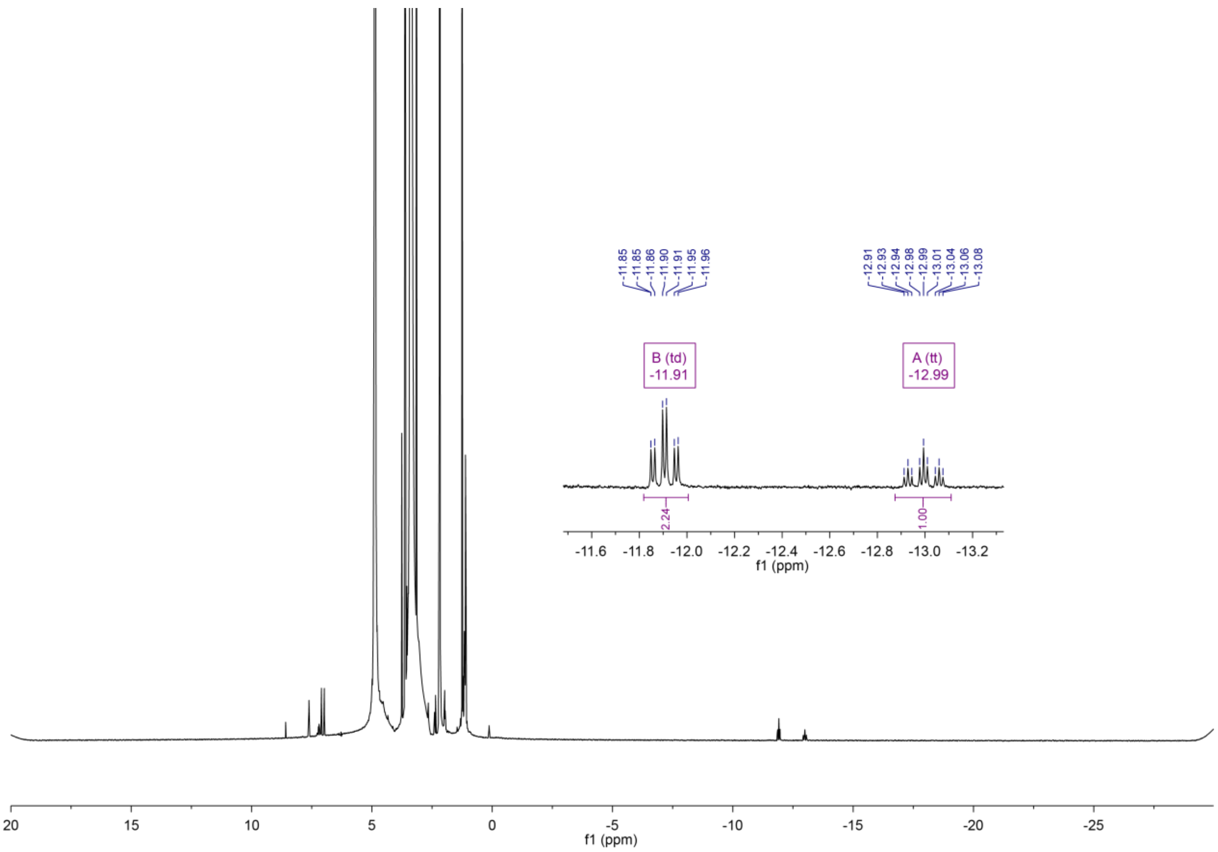

Figure S31. ^{1}H NMR spectrum of **$2H_3$** in CD_3OD

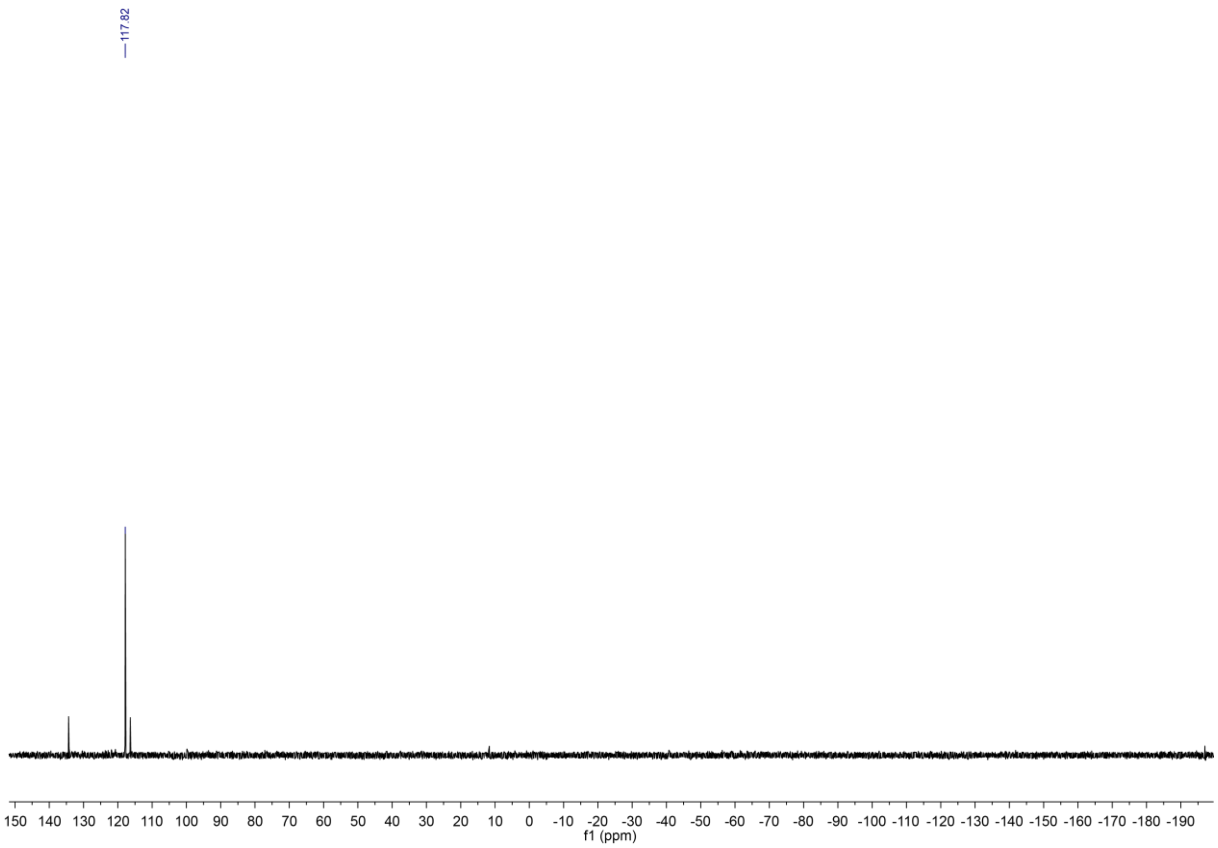

Figure S32. ^{31}P NMR spectrum of **$2H_3$** in CD_3OD

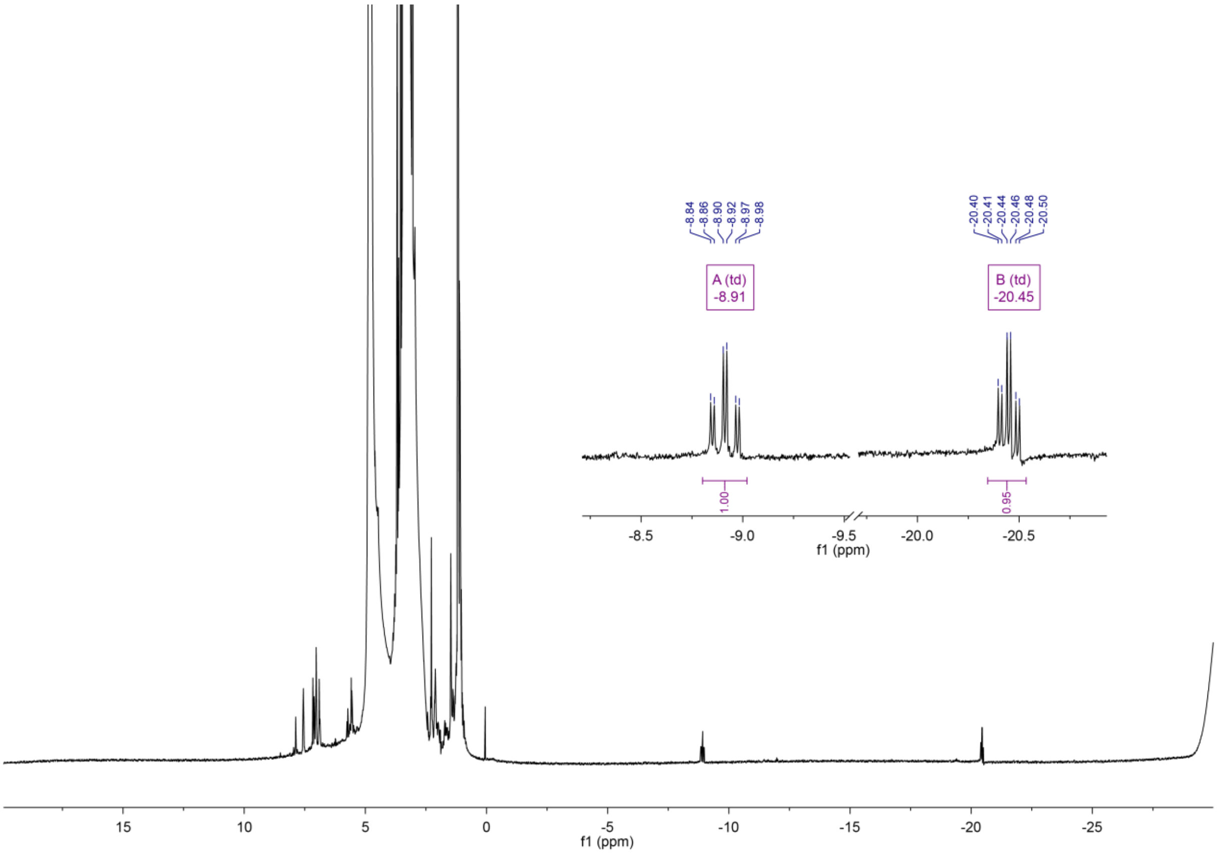

Figure S33. ^{1}H NMR spectrum of **$5H_2$** in CD_3OD

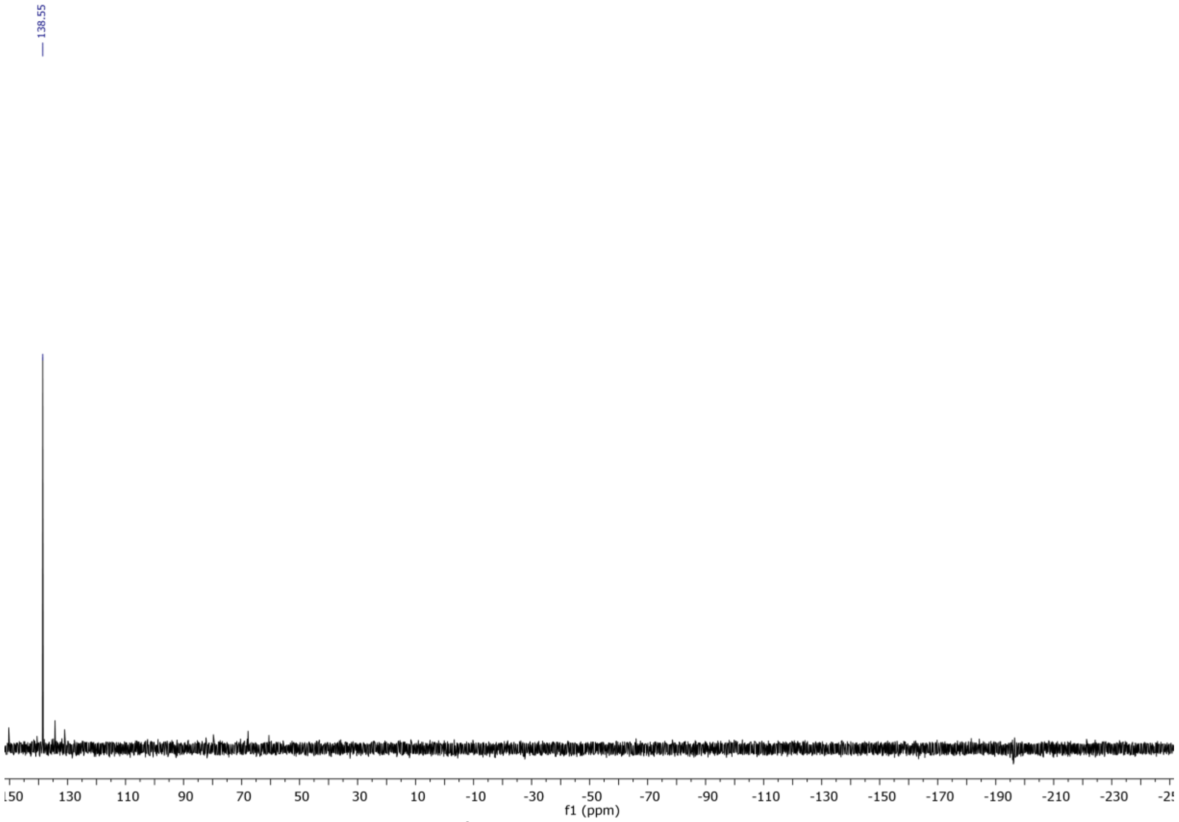

Figure S34. ^{31}P NMR spectrum of **$5H_2$** in CD_3OD

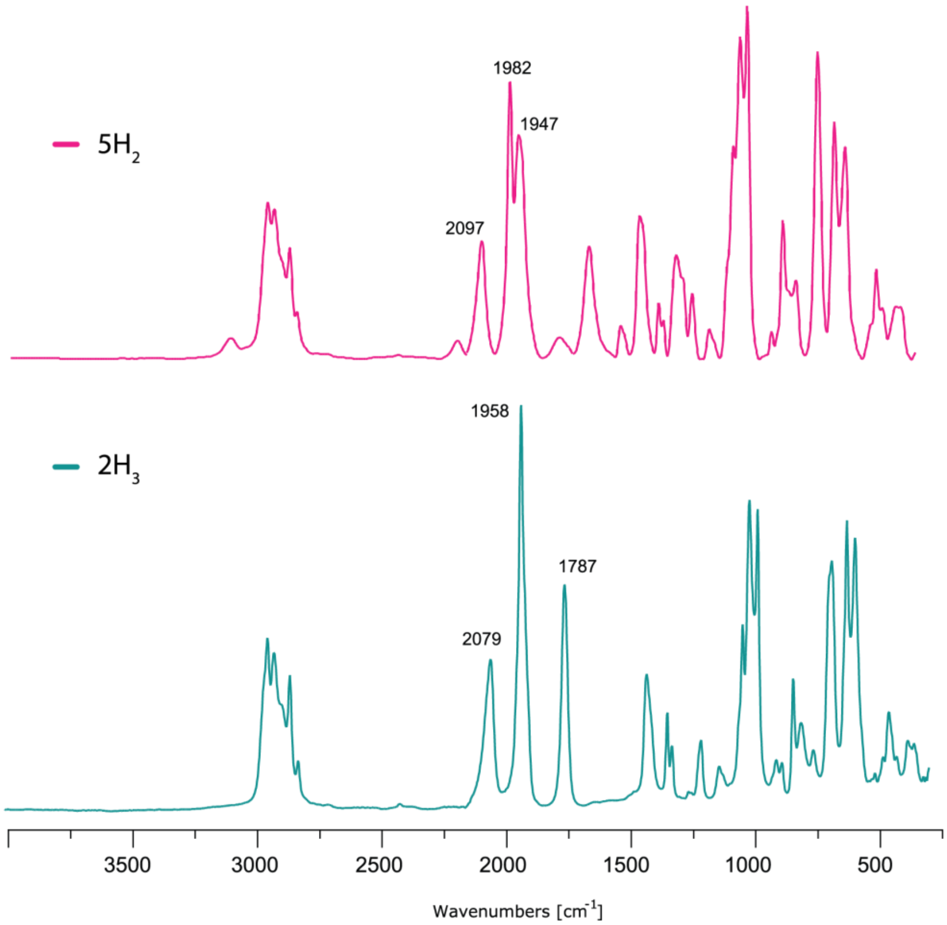

Figure S35. IR spectra comparison for **5H2** and **2H3** (methanol solution)

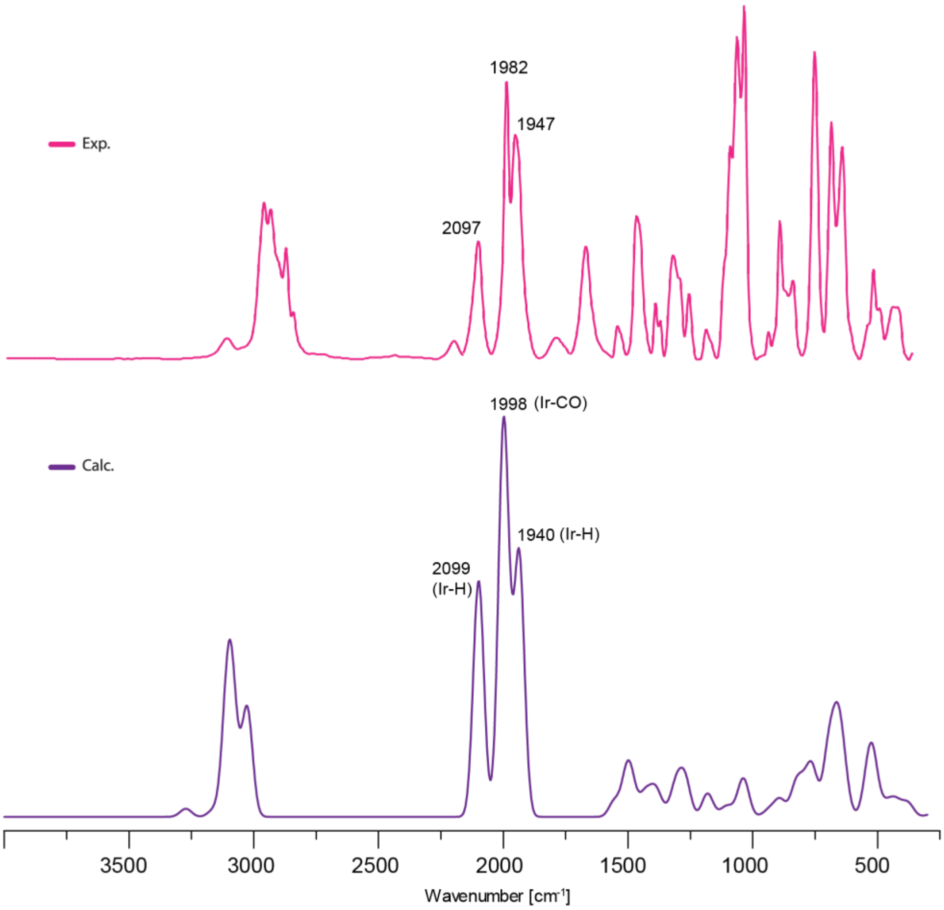

Figure S36. IR Spectra comparison for **5H$_2$**.

1.14 ^{13}CO labeling experiments

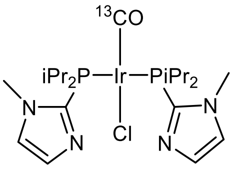

A suspension of [{IrCl(COD)}$_2$] (1000 mg, 1.489 mmol) in toluene (10 mL) was treated with P*i*Pr$_2$Im^{Me} (847 mg, 5.956 mmol) and stirred for 15 min. The reaction mixture was degassed two times, filled with ^{13}CO (1 bar) and stirred for 20 h. All volatiles were then removed *in vacuo,* and the residue was washed with Et$_2$O (3x10 mL). After recrystallisation in toluene, **^{13}CO-2** (1.195 mg, 1.830 mmol, 61%) was obtained as pale-yellow solid.

^{1}H NMR (300 MHz, CD$_3$OD) δ = 7.35 (m, 2H, Im-H), 7.23 (m, 2H, Im-H), 4.29 (s, 6H, N-CH$_3$), 3.04 (m, 4H, CH), 1.35-1.24 (m, 12H, *i*Pr-CH$_3$), 1.22-1.13 (m, 12H, *i*Pr-CH$_3$); **^{31}P NMR** (121 MHz, CD$_3$OD) δ = 26.7 (d, $^2J_{P,C}$ = 12.1 Hz); **^{13}C NMR** (75.5 MHz, CD$_3$OD) δ = 170.1 (t, $^2J_{C,P}$ = 12.1 Hz) ppm; **IR (ATR)**: $\tilde{\nu}$ = 1895 (s, Ir-^{13}CO) cm^{-1}.

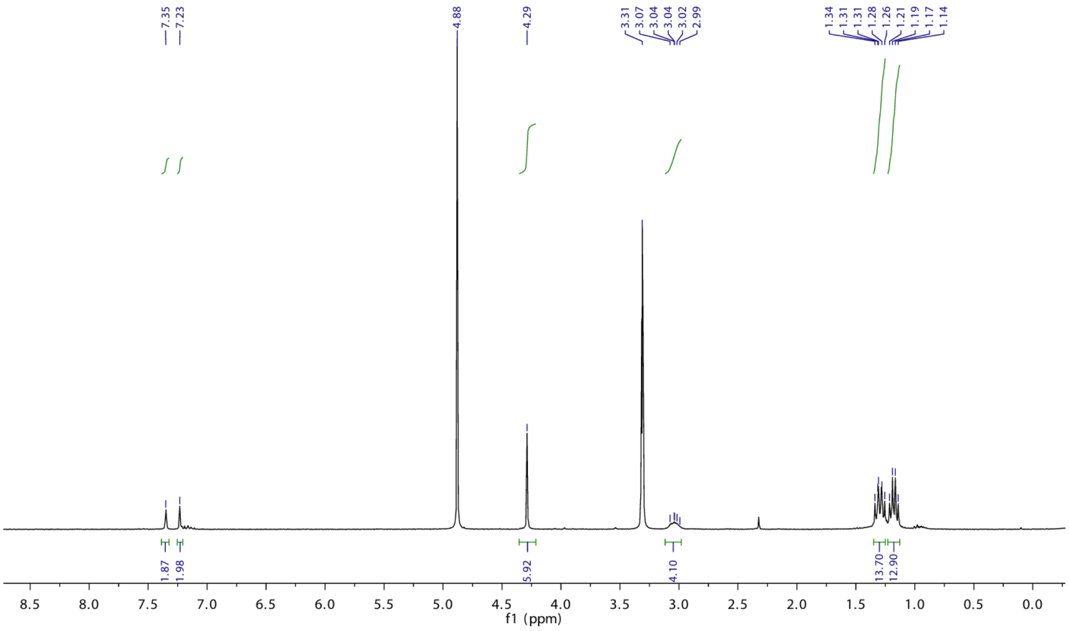

Figure S37. ^{1}H NMR spectrum of **^{13}CO-2** in CD_3OD

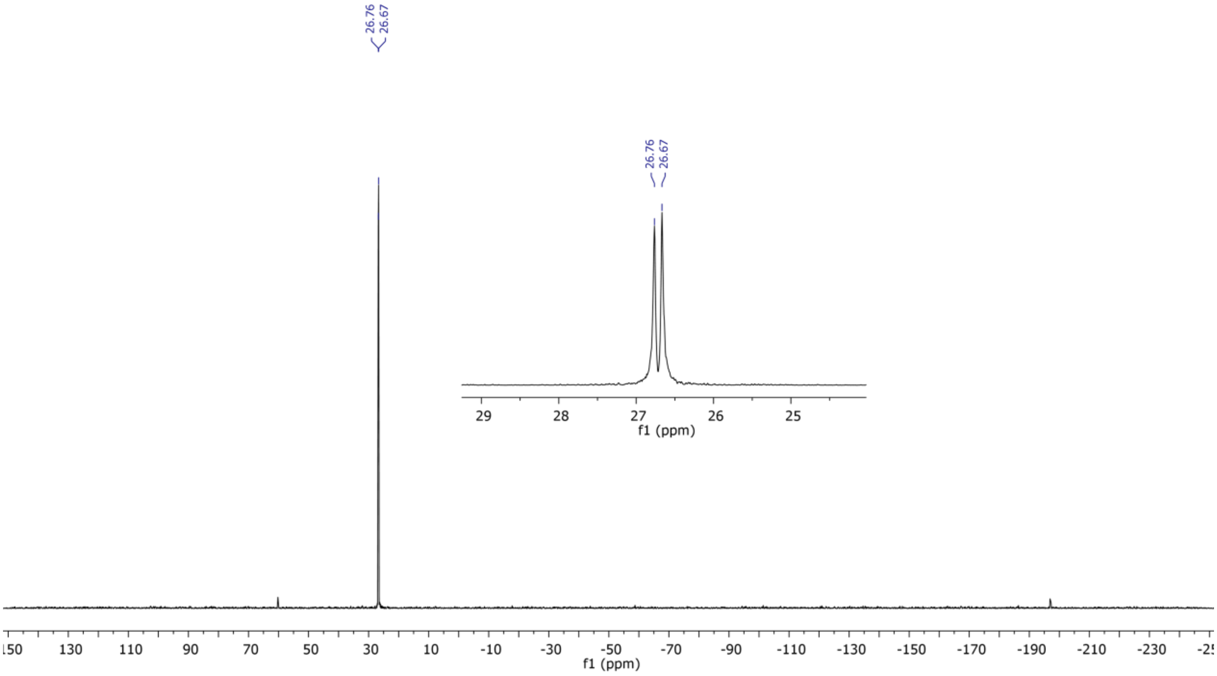

Figure S38. $^{31}P\{^{1}H\}$ NMR spectrum of **^{13}CO-2** in CD_3OD

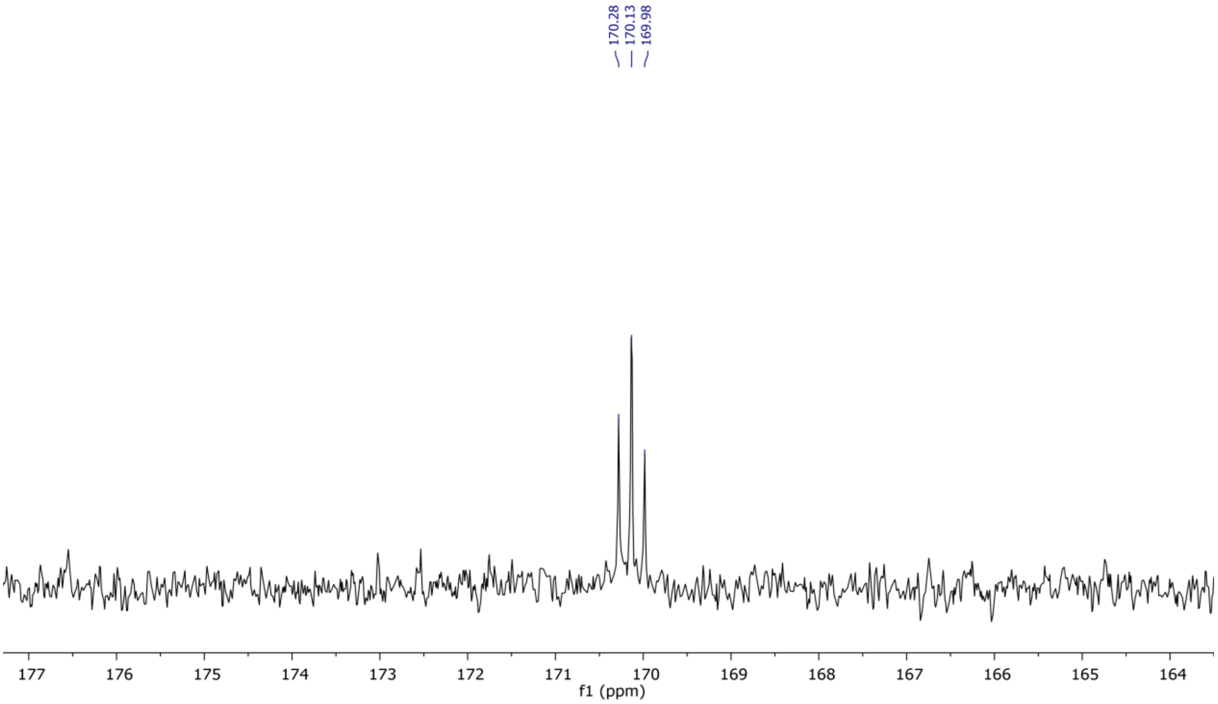

Figure S39. $^{13}C\{^1H\}$ NMR spectrum of **^{13}CO-2** in CD_3OD

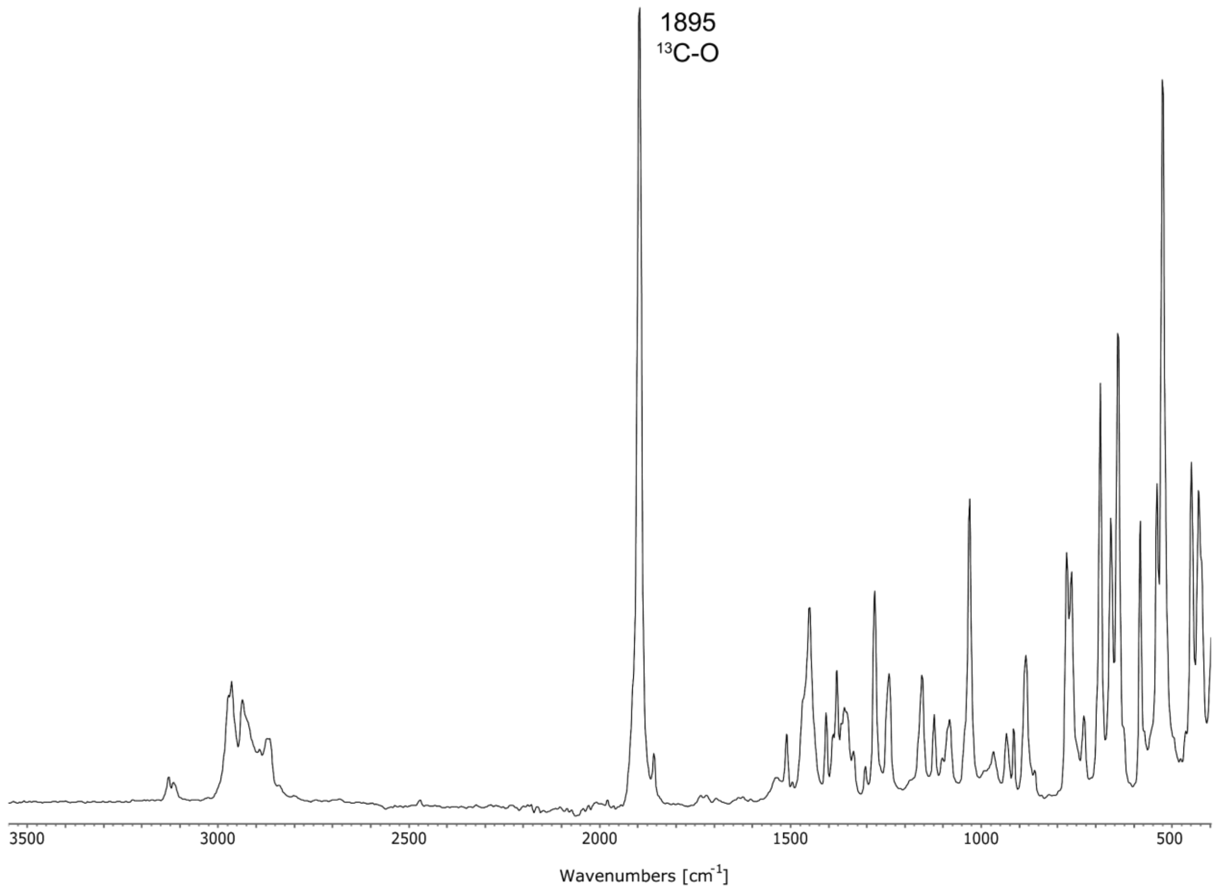

Figure S40. IR spectrum of **^{13}CO-2**

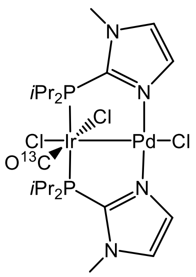

[Ir(CO)(P*i*Pr$_2$Im)$_2$]Cl (**^{13}CO-2**) (200 mg, 0.307 mmol) and *cis*-[PdCl$_2$(COD)] (88 mg, 0.157 mmol) were stirred in THF (10 mL) for 2 d. The solvent was removed *in vacuo* and the residue was washed first with EtOH (2x3 mL) and then with Et$_2$O (2x3 mL). After drying under high vacuum, **^{13}CO-5** (121 mg, 0.145 mmol, 47%) was obtained as an orange solid.

Analytical data for **^{13}CO-5**: IR (ATR**)**: $\tilde{\nu}$ = 1903 (s, ^{13}CO) cm^{-1}. ^{1}H NMR (300 MHz, DMSO-d$_6$): δ = 7.54 (m, 2H, Im-H), 7.34 (m, 2H, Im-H), 3.89 (s, 6H, N-CH$_3$), 3.41 (m, 2H, CH), 3.20 (m, 2H, CH), 1.76-1.63 (m, 12H, CH3), 1.47-1.32 (m, 12H, CH$_3$); **^{31}P NMR** (121 MHz, DMSO-d$_6$): δ = 10.5 (d, $^{2}J_{P,C}$ = 9.2 Hz); **^{13}C NMR:** (75.5 MHz, DMSO-d$_6$) δ = 165.8 (t, $^{2}J_{C,P}$ = 9.2 Hz) ppm; **IR (ATR)**: $\tilde{\nu}$ = 1903 (s, Ir-^{13}CO) cm^{-1}.

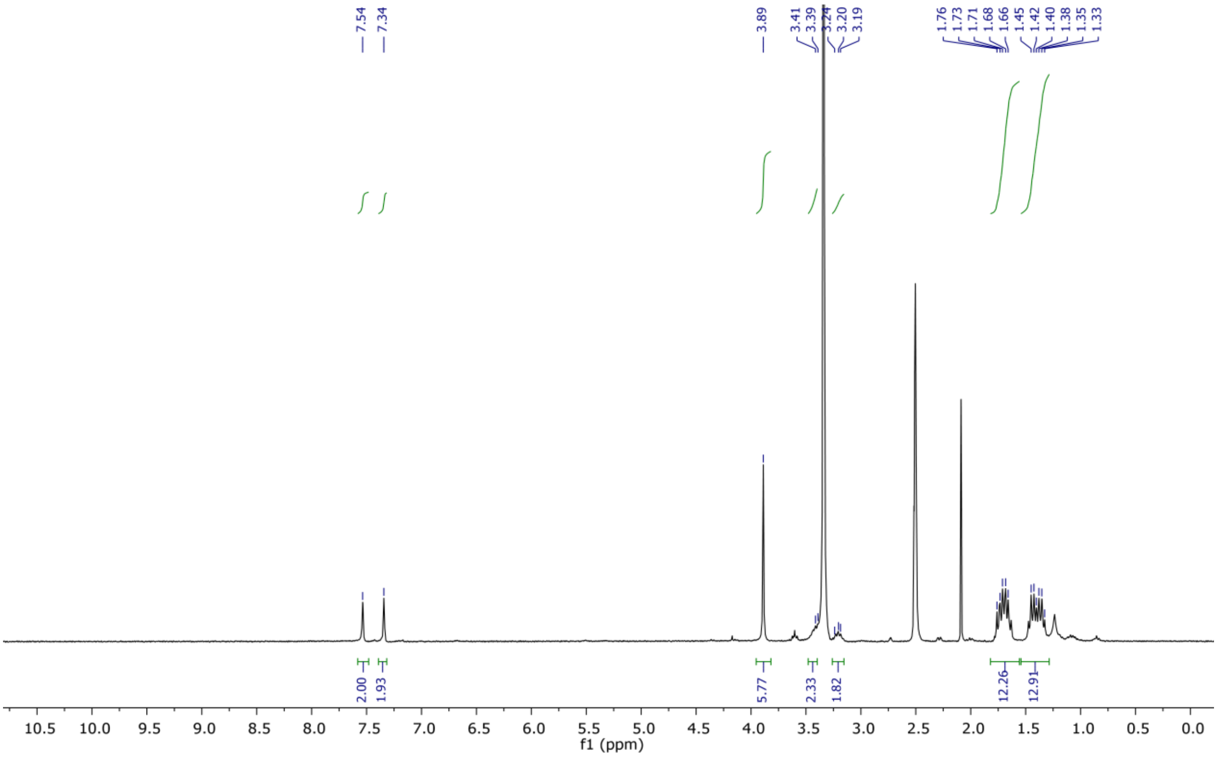

Figure S41. ^{1}H NMR spectrum of **^{13}CO-5** in DMSO-d$_6$

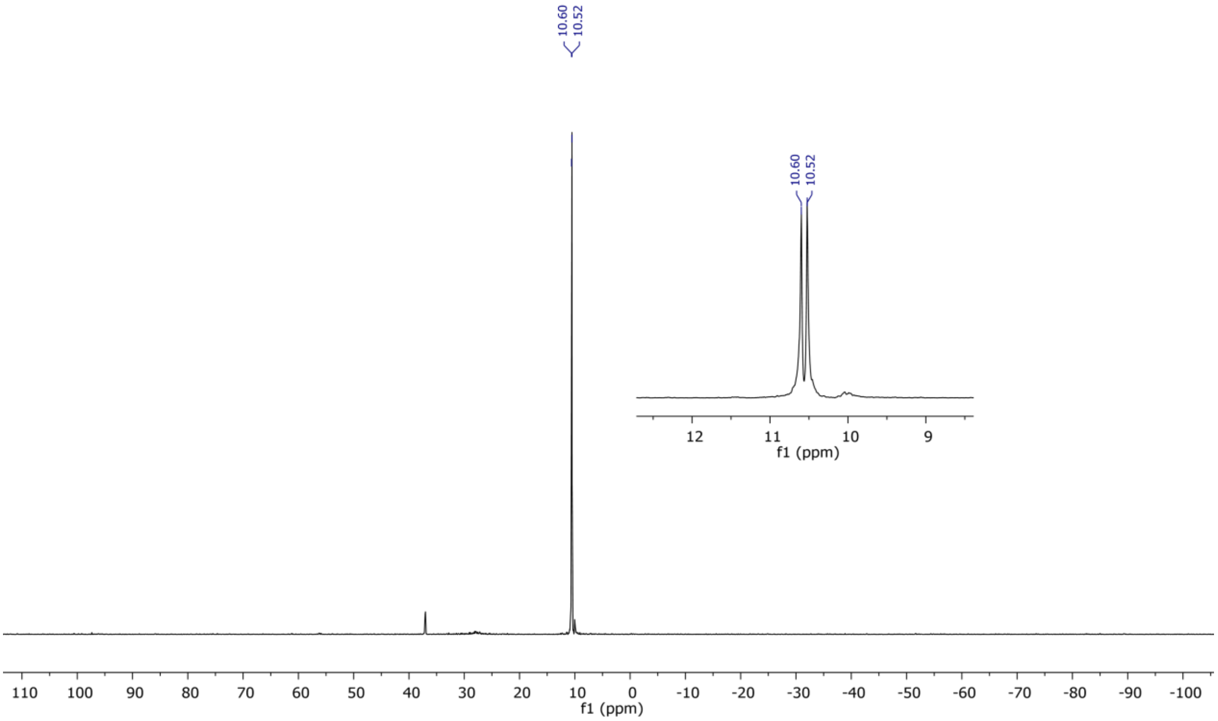

Figure S42. $^{31}P\{^{1}H\}$ NMR spectrum of **^{13}CO-5** in DMSO-d$_6$

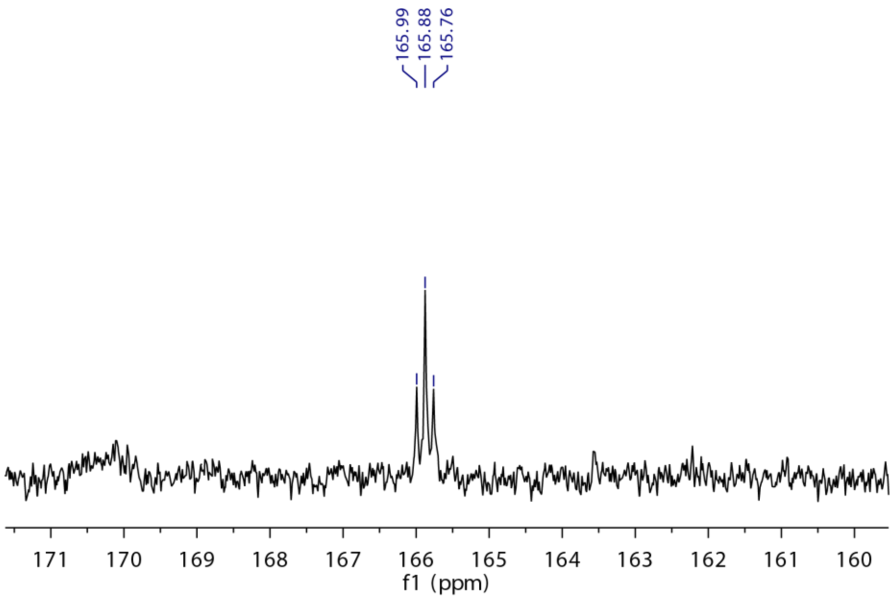

Figure S43. $^{13}C\{^{1}H\}$ NMR spectrum of **^{13}CO-5** in DMSO-d$_6$

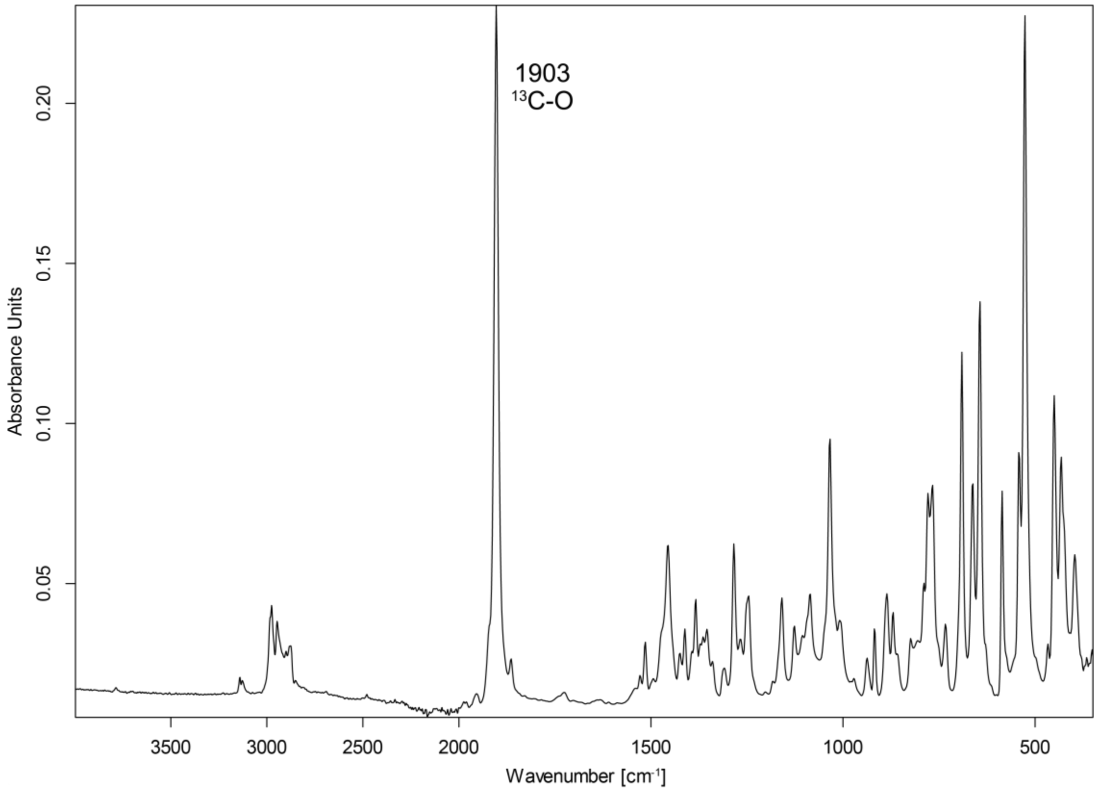

Figure S44. IR spectrum of **^{13}CO-5**

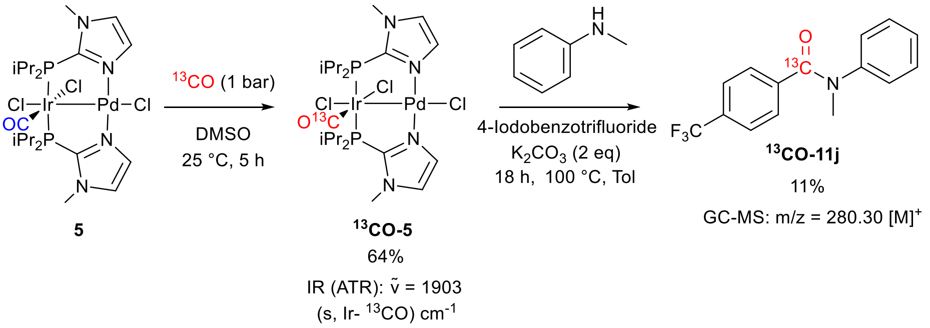

Scheme S4. Formation of **^{13}CO-5** from **5** and it's reactivity in the aminocarbonylation of *N*-methylaniline

A 10ml young-flask containing **5** (50 mg, 0.060 mmol) was dissolved in DMSO (1.5 ml) and after degassing, the resulting orange solution was put under 1 bar of ^{13}CO. After 1 hour at room temperature a brown solution was formed which was further stirred for an additional 4 hours at room temperature. The solvent was removed under vacuum to afford **^{13}CO-5** as a brown solid (32.4 mg, 0.039 mmol, 64%), which was analysed by IR and NMR without further purification. The analytical data matched well with those of **^{13}CO-5** obtained via the synthesis from **^{12}CO-2** (see above).

For the aminocarbonylation using **^{13}CO-5**, isolated **^{13}CO-5** (41 mg, 5 mol%) was added to a 5 ml toluene solution of *N*-methylaniline (1 mmol), K_2CO_3 (2 eq) and 4-iodobenzotrifluoride (1.2 mmol). The mixture was then stirred overnight. The reaction mixture was passed through a short column eluting with diethyl ether to remove the catalyst and salts. GC-MS analysis using mesitylene as the internal standard revealed the formation of **^{13}CO-11j** in 11% of yield.

1.15 Methanol dehydrogenation with ^{13}CO-5

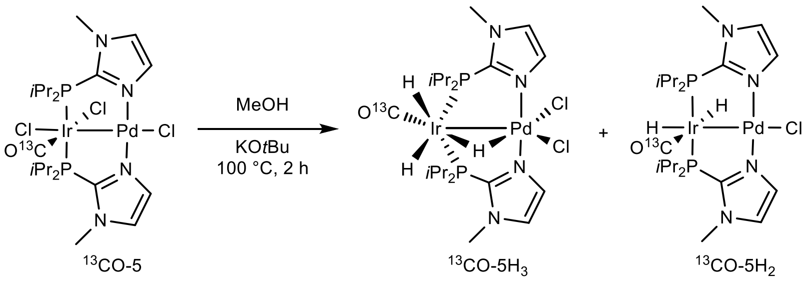

This reaction was carried out in methanol similarly to the **5H$_3$** and **5H$_2$** formation (section 1.11), but with **^{13}CO-5** instead of **5**. **^{1}H NMR** for **^{13}CO-5H$_3$** (300 MHz, CD$_3$OD) δ = -12.03 (tdd, $^2J_{H,P}$ = 14.7, $^2J_{H,H}$ = 4.9, $^2J_{H,C}$ = 3.9 Hz, 2H), -13.12 (dtt, $^2J_{H,C}$ = 33.7, $^2J_{H,P}$ 19.7, $^2J_{H,H}$ = 4.9 Hz, 1H); **^{1}H NMR** for **^{13}CO-5H$_2$** (300 MHz, CD$_3$OD) δ = -8.96 (dtd, $^2J_{H,C}$ = 42.0, $^2J_{H,P}$ = 18.5, $^2J_{H,H}$ = 5.3 Hz, 1H), -20.48 – -20.58 (m, 1H); **^{1}H {^{31}P} NMR** for **^{13}CO-5H$_3$** (300 MHz, CD$_3$OD) δ = -12.03 (dd, $^2J_{H,H}$ = 4.9, $^2J_{H,C}$ = 3.9, 1H), -13.12 (dt, $^2J_{H,C}$ = 33.5, $^2J_{H,H}$ = 4.9, 1H); **^{1}H {^{31}P} NMR** for **^{13}CO-5H$_2$** (300 MHz, CD$_3$OD) δ = -8.96 (dd, $^2J_{H,C}$ = 41.6, $^2J_{H,H}$ = 5.1, 1H) -20.48 – -20.58 (m, 1H); **^{31}P NMR** for **^{13}CO-5H$_3$** (121 MHz, CD$_3$OD) δ = 150.65 (d, J = 7.0 Hz); **^{31}P NMR** for **^{13}CO-5H$_2$** (121 MHz, CD$_3$OD) δ = 138.60 (d, J = 6.6 Hz); **^{13}C NMR** for **^{13}CO-5H$_3$** (75 MHz, CD$_3$OD) δ = 178.21 (t, J = 7.0 Hz); **^{13}C NMR** for **^{13}CO-5H$_2$** (75 MHz, CD$_3$OD) δ = 178.11 (t, J = 6.6 Hz); **^{1}H,^{13}C HMBC NMR** (300 MHz/75 MHz, CD$_3$OD): δ = -8.96/178.11 (dtd/t), -12.03/178.21 (tdd,t), -13.12/178.21 (dtt,t), -20.48 – -20.58/178.11 (m/t) ppm.

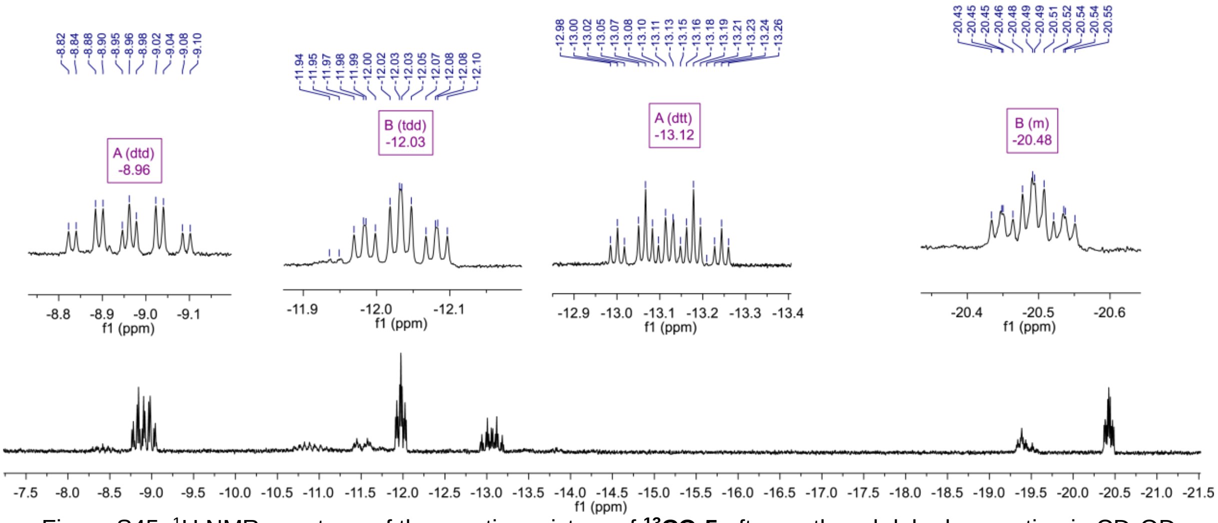

Figure S45. ^{1}H NMR spectrum of the reaction mixture of **^{13}CO-5** after methanol dehydrogenation in CD$_3$OD

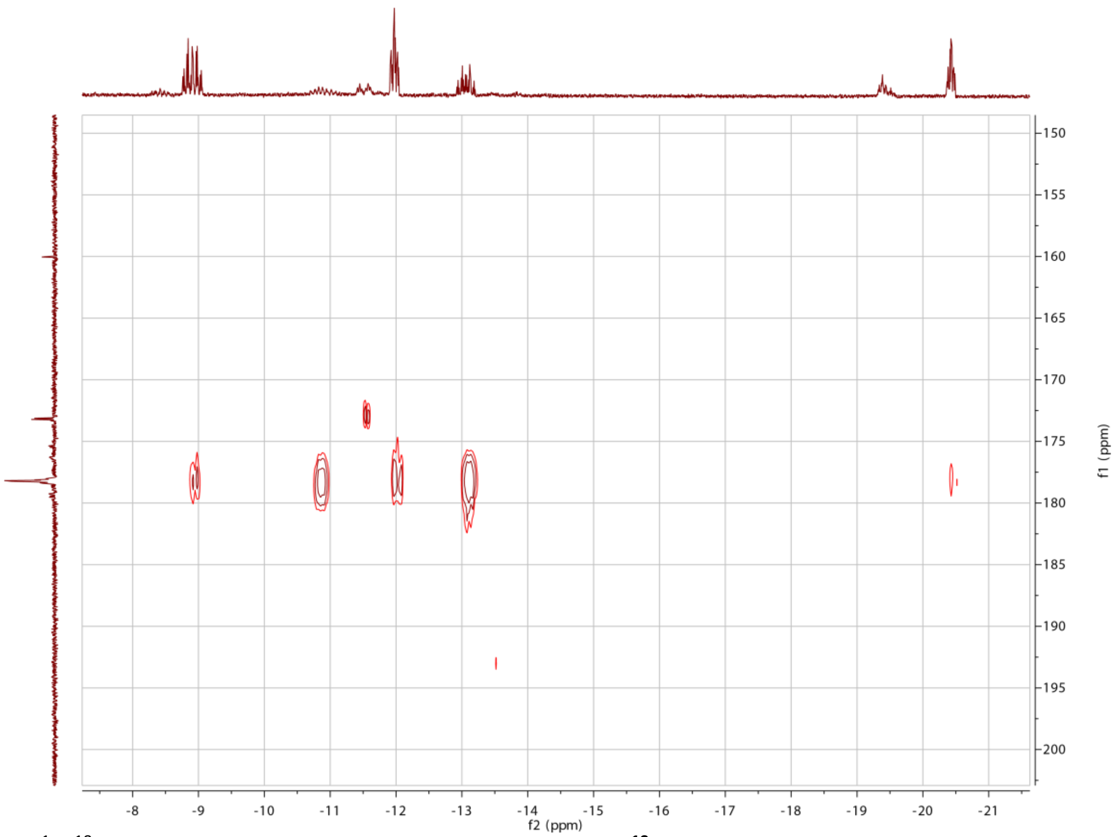

Figure S46. $^{1}H,^{13}C$ HMBC NMR spectrum of the reaction mixture of **^{13}CO-5** toward methanol dehydrogenation in CD_3OD

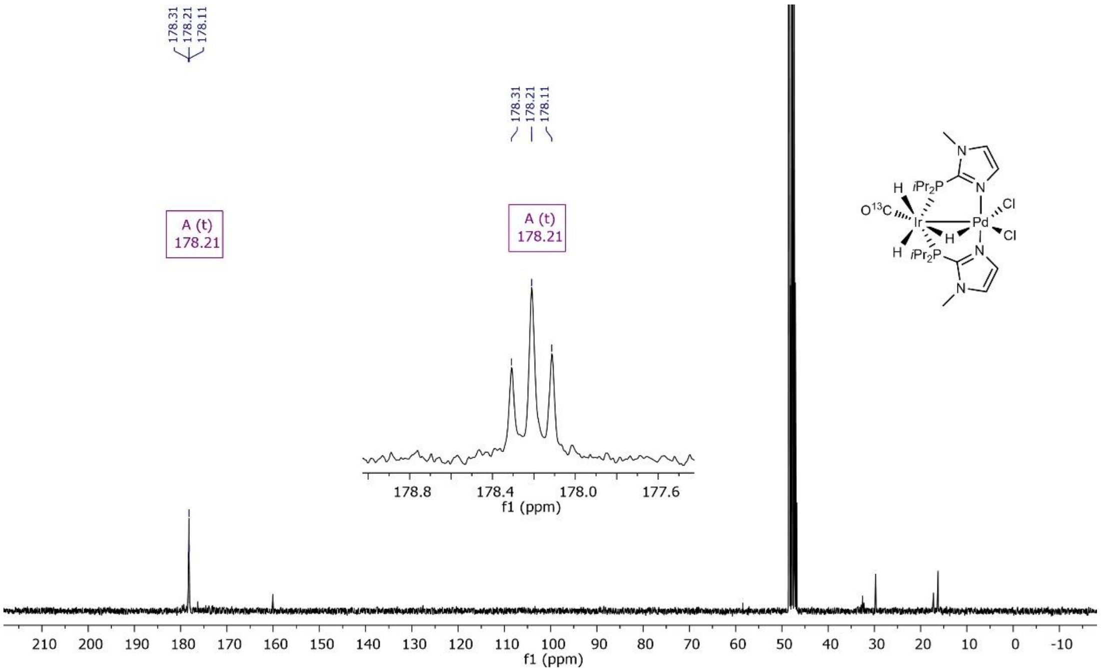

Figure S47. ^{13}C {^{1}H} NMR spectrum of **^{13}CO-5H$_3$** in CD_3OD

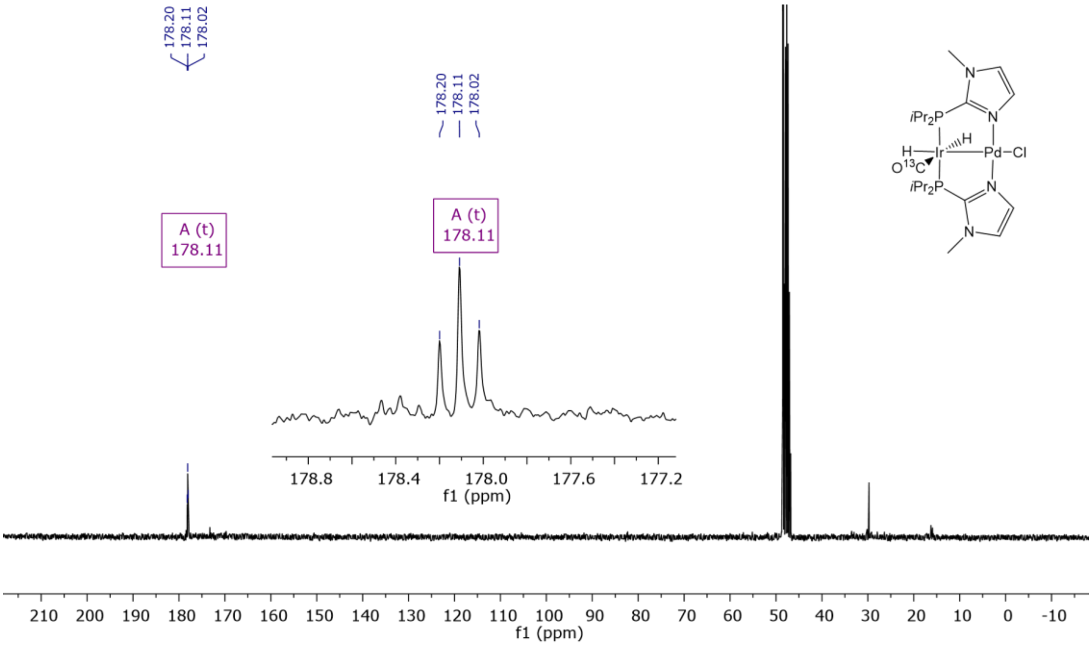

Figure S48. ^{13}C {^{1}H} NMR spectrum of **^{13}CO-5H$_2$** in CD_3OD

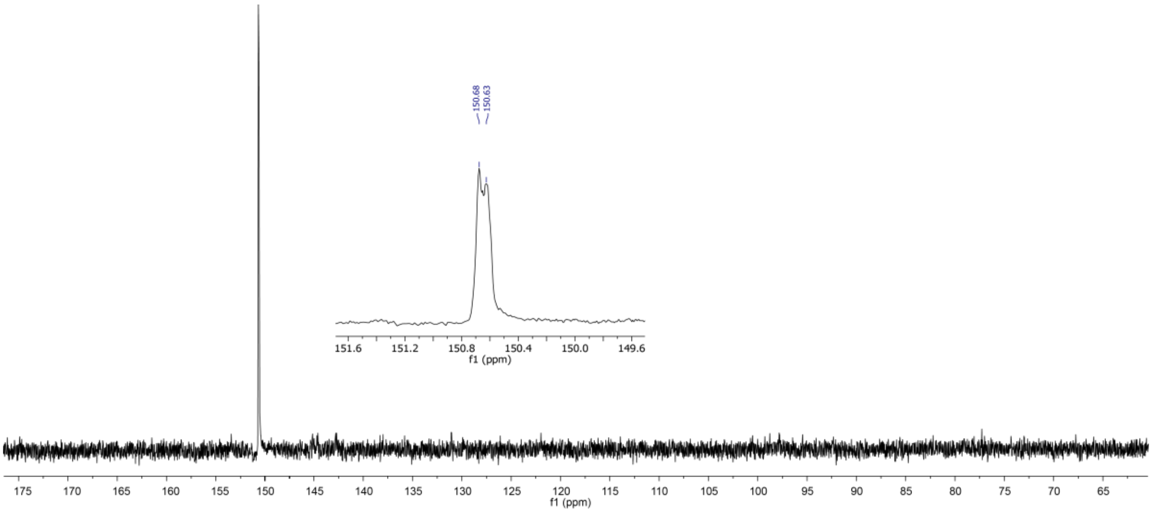

Figure S49. ^{31}P {^{1}H} NMR spectrum of **^{13}CO-5H$_3$** in CD_3OD

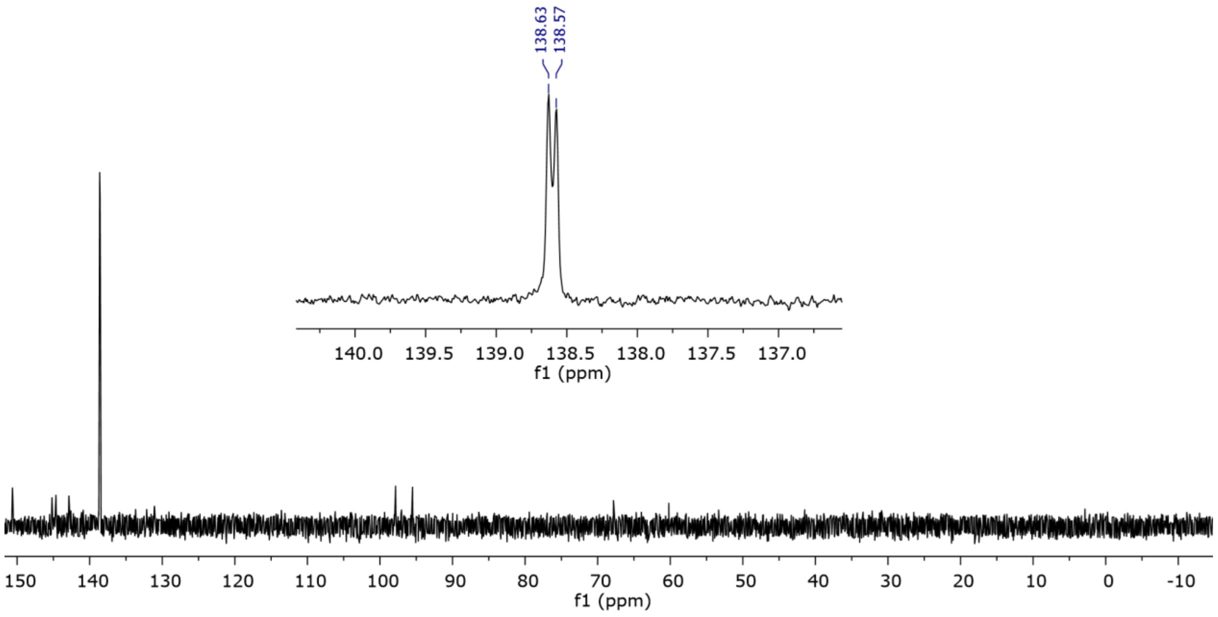

Figure S50. ^{31}P {^{1}H} NMR spectrum of **^{13}CO-5H$_2$** in CD_3OD

1.16 Formation of the species **5H**

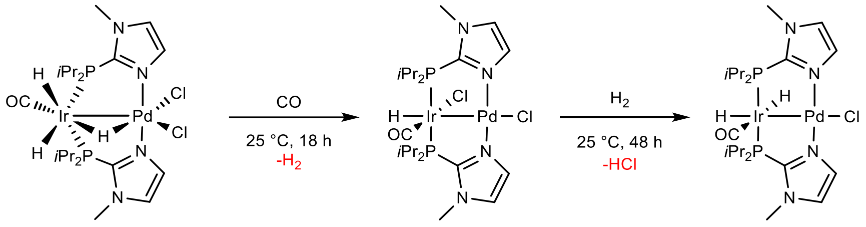

5H$_3$ 5H 5H$_2$

A young NMR tube containing **5H$_3$** in a DCM solution was placed under a CO atmosphere (1 bar) and left for 18 hours at room temperature. Monitoring the tube via ^{1}H NMR confirmed the formation of **5H** overnight, and this monitoring continued for up to one month. **^{1}H NMR** (300 MHz, CD_2Cl_2) δ = -12.44 (t, J=14.8, 1H); **IR (ATR)**: $\tilde{\nu}$ = 1949 (s, Ir-H), 1978 (s, Ir-CO) cm^{-1}.

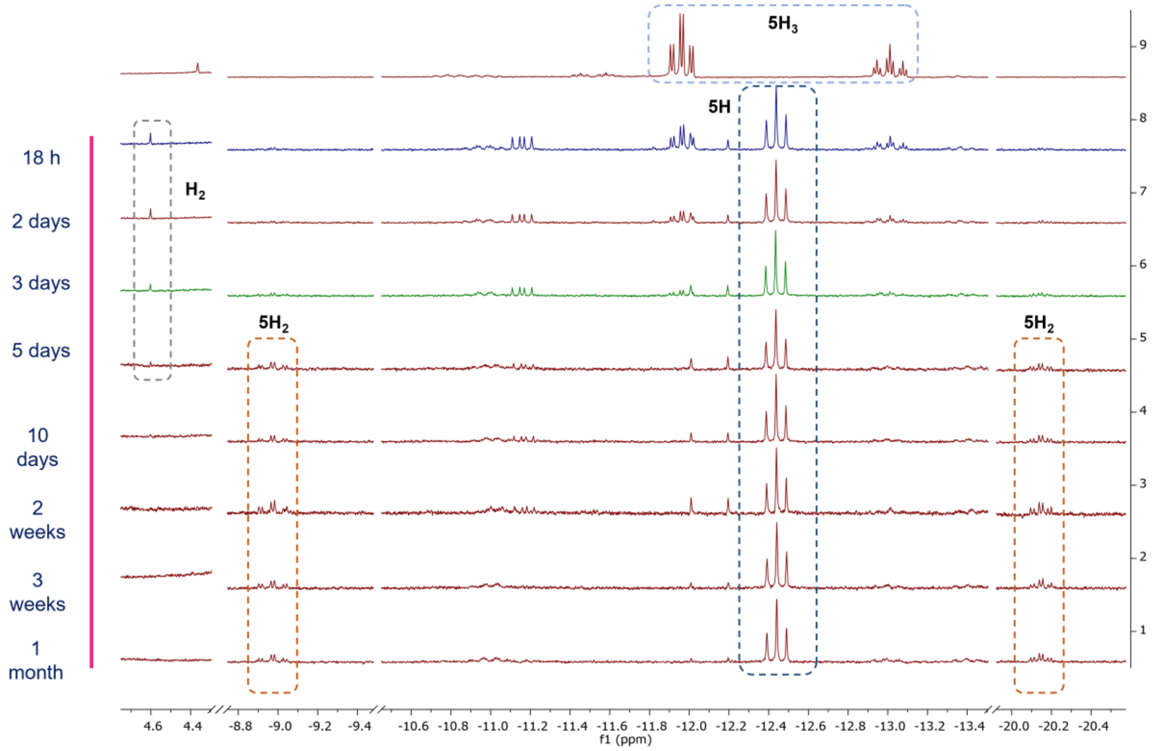

Figure S51. ^{1}H NMR monitoring of the reactivity of **5H3** towards CO in CD_2Cl_2.

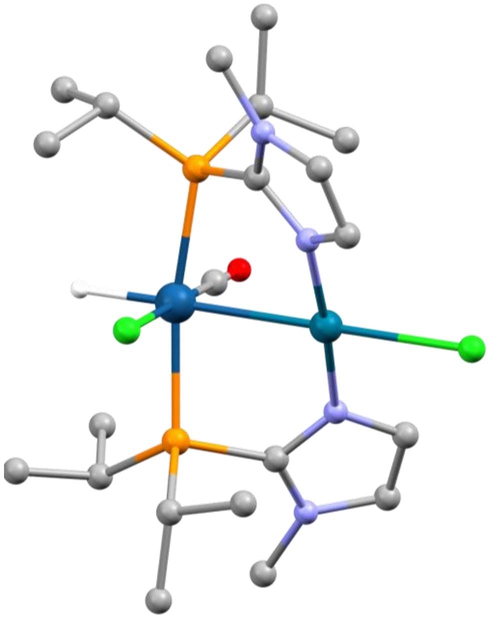

Figure S52. DFT calculated structure of **5H**

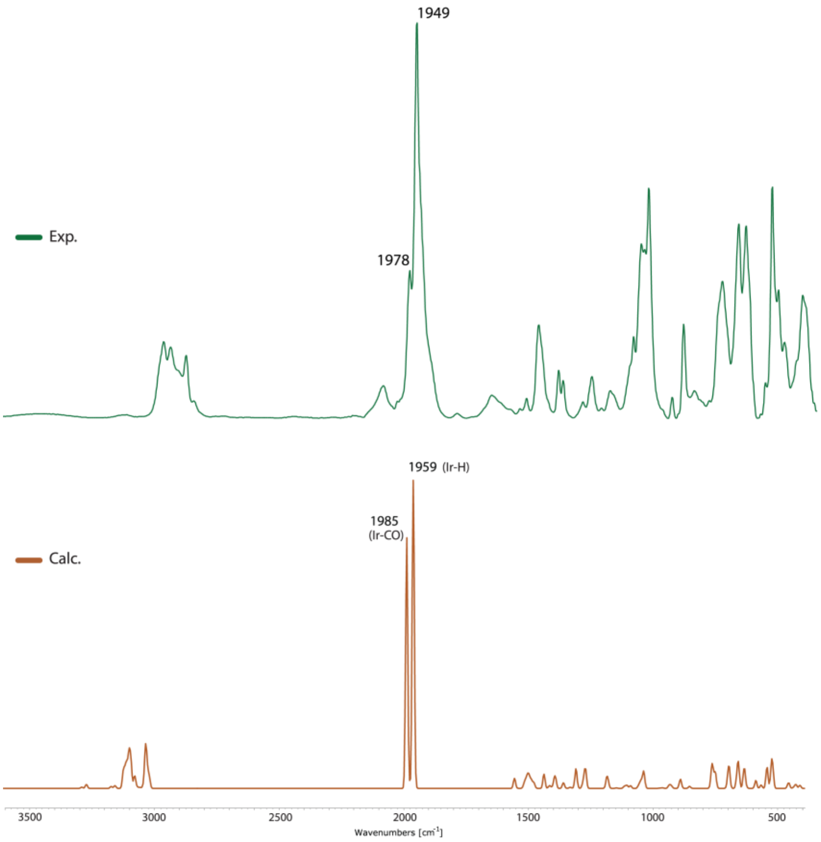

Figure S53. IR Spectra comparison for **5H**

1.17 Synthesis of **$3H_2$** [5]

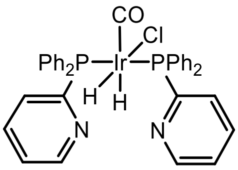

This compound was synthesized from [Ir(CO)($Ph_2PPy)_2$Cl] (**3**), and H_2 gas in DCM according to the described procedure.[5] **^{1}H NMR** (300 MHz, DCM-d_2) δ = 8.72 (d, $^2J_{H,H}$ = 4.6 Hz, 2H), 7.89 (d, $^2J_{H,H}$ = 7.7 Hz, 1H), 7.80-7.61 (m, 10H), 7.49-7.35 (m, 12H), 7.30 (ddd, $J_{H,H}$ = 7.7, 4.7, 1.1 Hz, 2H), -7.58 (td, IrH, 1H, $^2J_{H,P}$ = 17.7 Hz, $^2J_{H,H}$ = 4.8 Hz), -18.59 (td, IrH, 1H, $^2J_{H,P}$ = 14.3 Hz, $^2J_{H,H}$ = 4.8 Hz); **^{31}P NMR** (121 MHz, DCM-d_2): δ = 11.68 (s) ppm. **IR (ATR)**: $\tilde{\nu}$ = 1986 (s, CO), 2075 (s, Ir-H), 2195 (s, Ir-H) cm^{-1}. **ESI-MS** (+) (m/z) [M-H_2-Cl]$^+$ calculated for $C_{35}H_{28}IrN_2OP_2$, 747.1; found, 747.1.

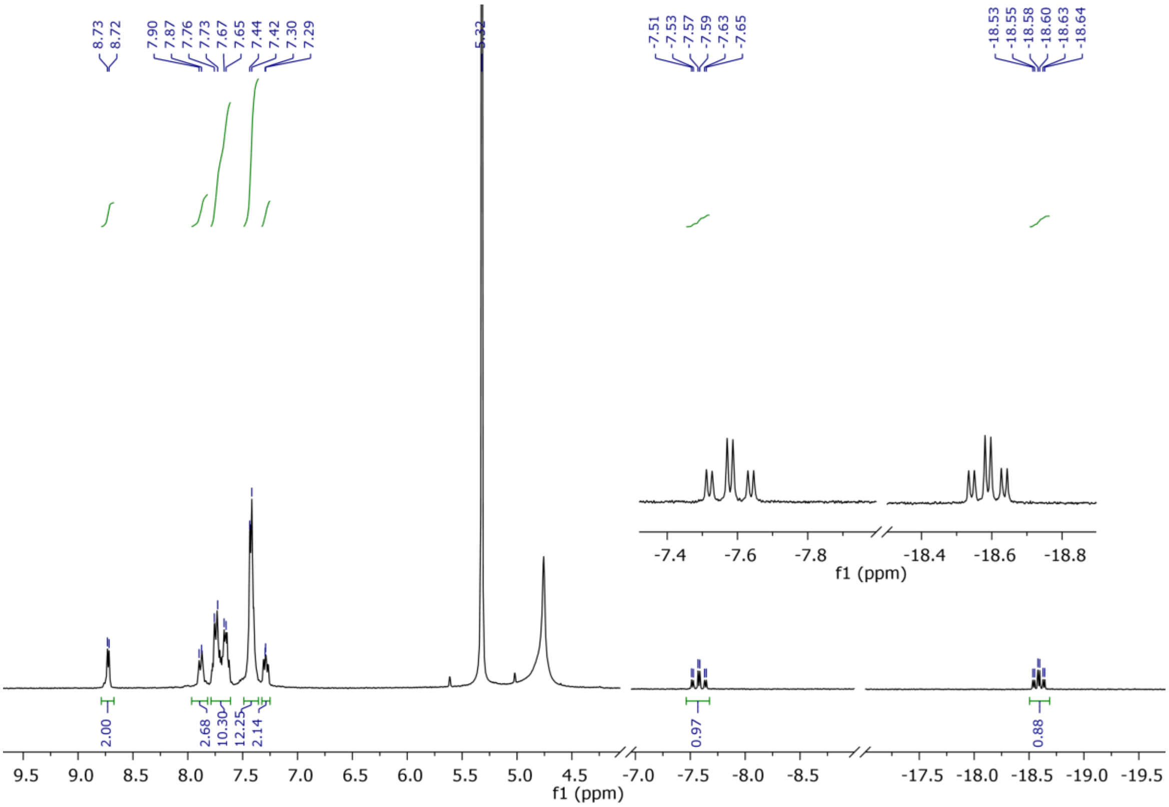

Figure S54. ^{1}H NMR spectrum for **3H₂** in DCM-d₂

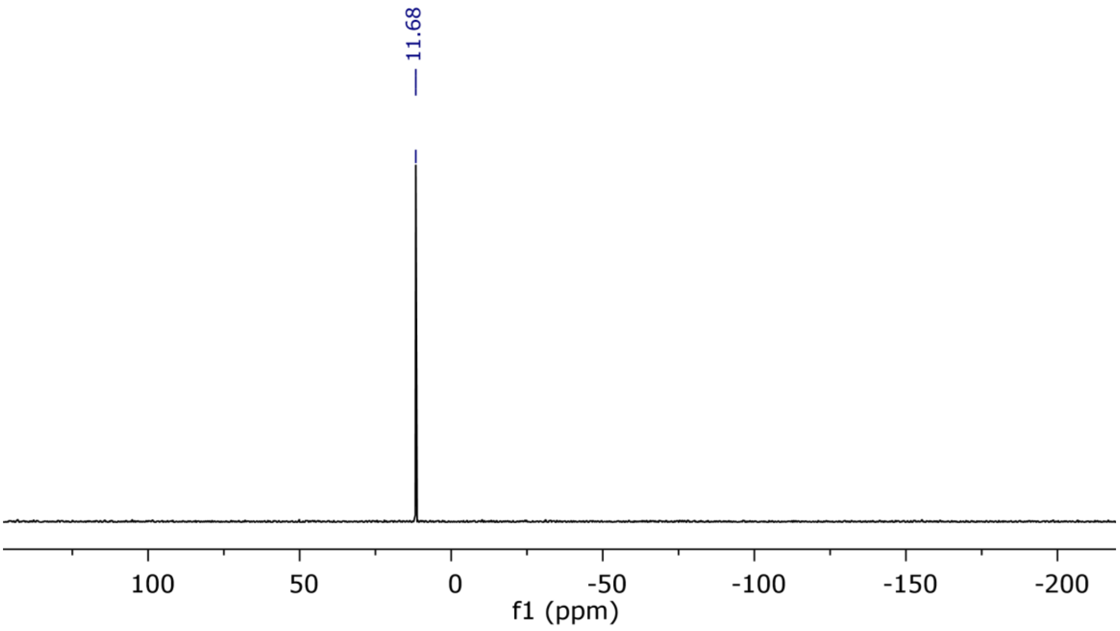

Figure S55. ^{31}P{^{1}H} NMR spectrum for **3H₂** in DCM-d₂

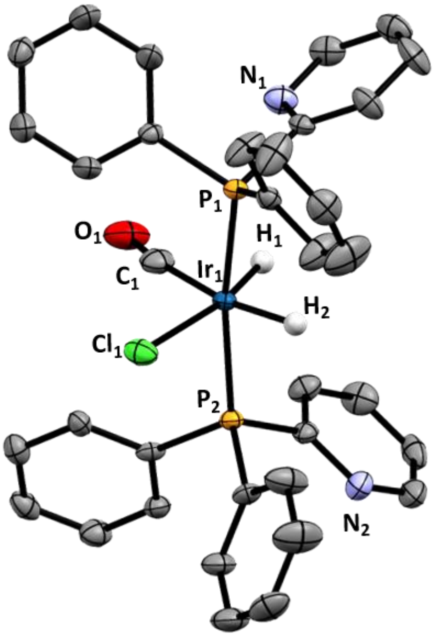

Figure S56. Molecular structure in the solid state of [IrClH$_2$(CO)(PPh$_2$Py)$_2$] (**3H$_2$**). Electron density which was assigned to the hydrido ligands was found in the difference Fourier map and allowed for a free refinement.

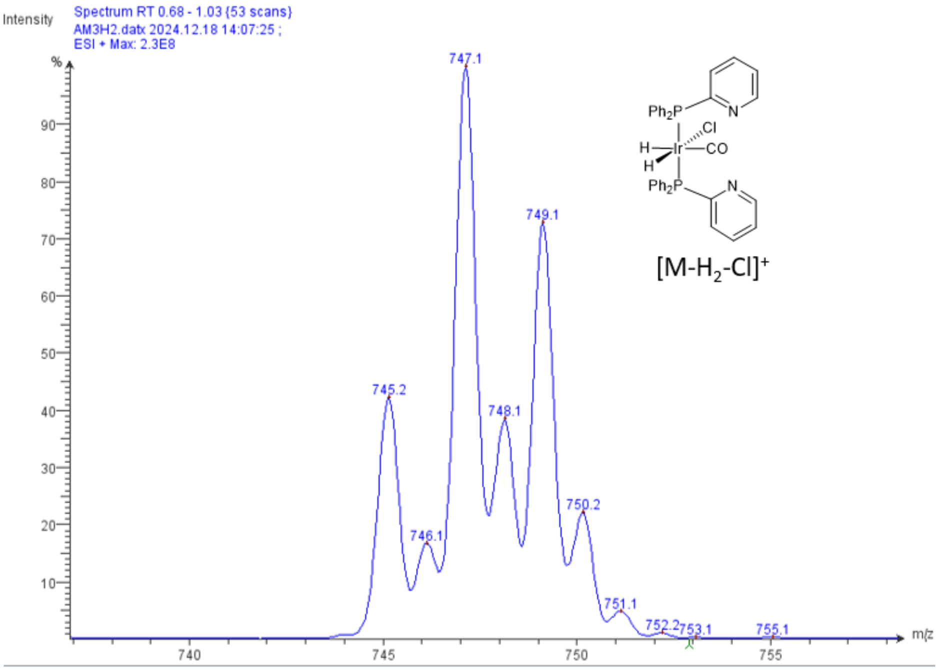

Figure S57. ESI-MS Spectrum for **3H$_2$**

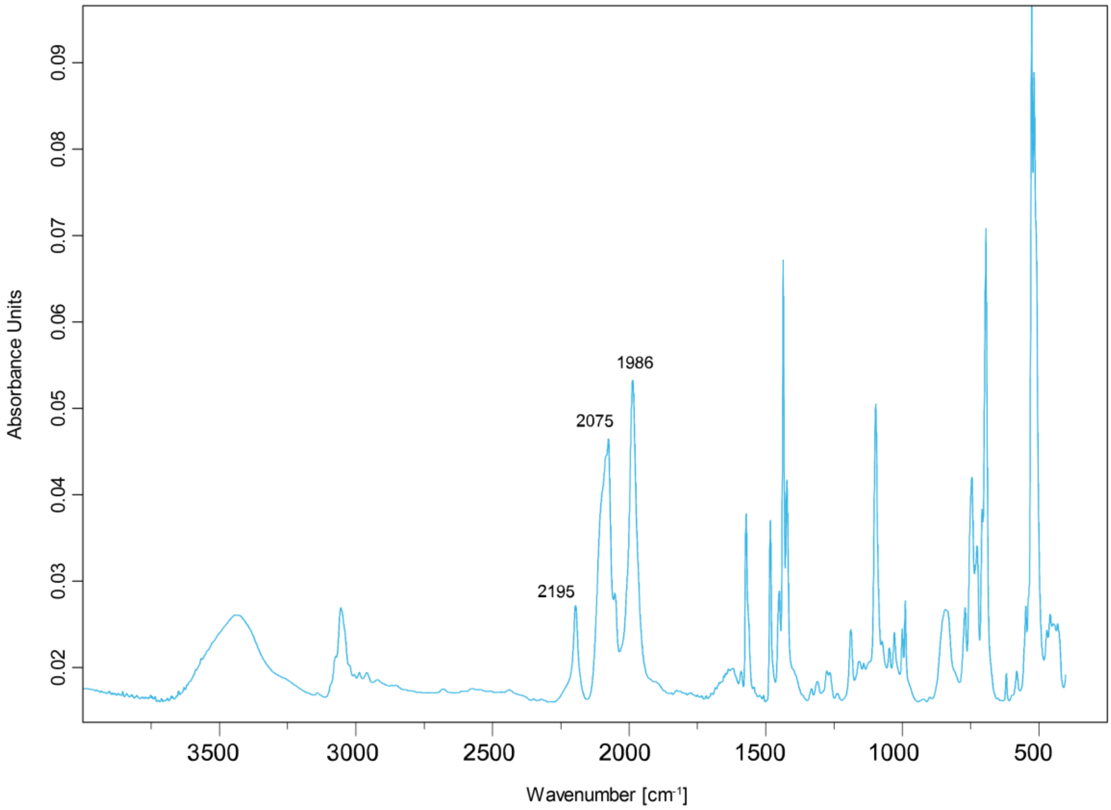

Figure S58. IR Spectrum of **$3H_2$**

1.18 Methanol dehydrogenation with **6**

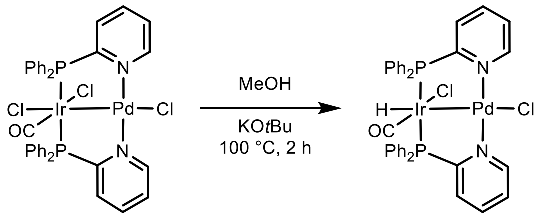

6 $6H_a/6H_b$

To a methanol solution of **6** (30 mg, 0.031 mmol) was added KO*t*Bu (13 mg, 0.117 mmol) and stirred at 100 °C for 2 hours. The brown solution was evaporated and the residue was washed with Et_2O and immediately redissolved in DCM-d_2 for further NMR analysis. Based on ^{1}H NMR data, 9% of **6** was converted into **$6H_a/6H_b$**. **^{1}H NMR** (300 MHz, DCM-d_2) δ = 9.32 (d, *J*=5.3 Hz, 10H), -16.58 (t, *J*=15.0 Hz, 1H).

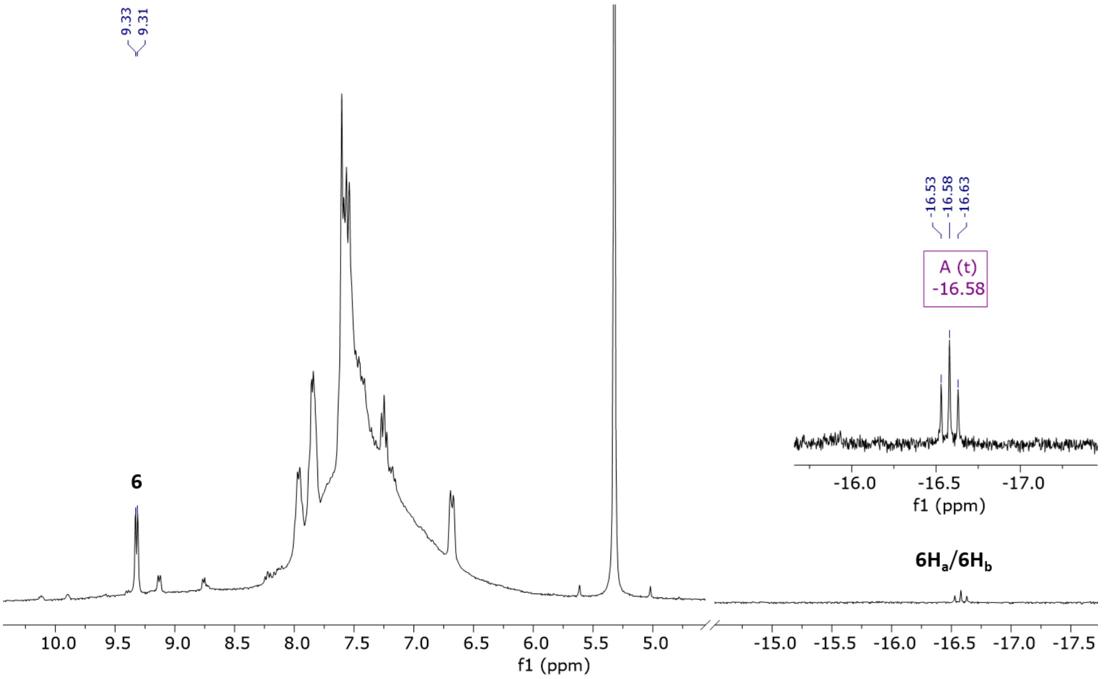

Figure S59. ^{1}H NMR of the reaction mixture of **6** through methanol dehydrogenation in DCM-d$_2$. The signal at δ = 9.32 ppm is related to the complex **6**

1.19 Independent synthesis of **6H$_a$/6H$_b$**

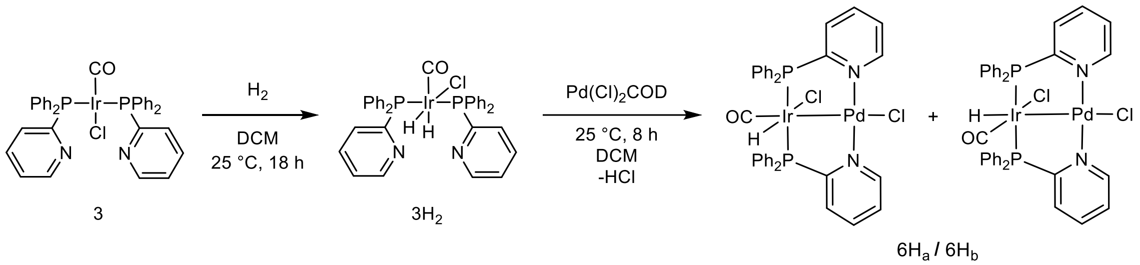

In a Schlenk flask added **3H$_2$** (80 mg, 0.10 mmol) and dissolved in DCM (10 ml) and [PdCl$_2$(COD)] (14 mg, 0.05 mmol) added to the bright yellow solution. The solution turned red after 1 hour stirring at room temperature, and after 8 hours a deep orange suspension was obtained. The solution was reduced to ca. 2 ml in vacuo and *n*-pentane was added slowly to obtain the isomers **6H$_a$** and **6H$_b$** as an orange powder (45 mg, 0.048 mmol, 48%). ^{1}H NMR and ^{31}P NMR confirmed the formation of **6H$_a$** or **6H$_b$**. **^{1}H NMR** (300 MHz, DCM-d$_2$) δ = 9.13 (d, $^{2}J_{H,H}$ = 5.6 Hz, 2H), 8.75 (d, $^{2}J_{H,H}$ = 4.5 Hz, 2H), -15.39 (t, $^{2}J_{H,P}$ = 11.5 Hz, 1H), -16.58 (t, $^{2}J_{H,P}$ = 15.0 Hz, 1H); **^{31}P NMR** (121 MHz, DCM-d$_2$) δ = 1.08 (s), -15.76 (s) ppm. **IR (ATR)**: $\tilde{\nu}$ = 1994 (s, CO), 2047 (s, CO), 2180 (s, Ir-H), 2192 (s, Ir-H) cm^{-1}. **ESI-MS** (+) (m/z) [M-H]$^+$ calculated for $C_{35}H_{28}Cl_2IrN_2OP_2Pd$, 922.9; found, 922.6; [M-Cl]$^+$ calculated for $C_{35}H_{29}ClIrN_2OP_2Pd$, 889.0; found, 888.6.

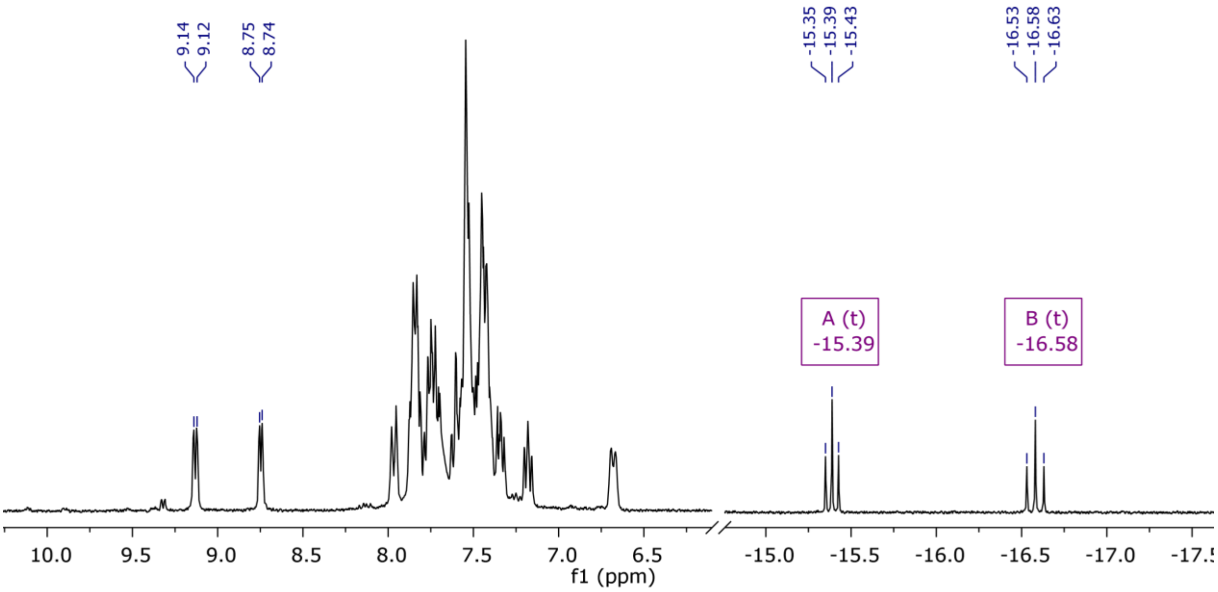

Figure S60. ^{1}H NMR of the mixture of **6Ha** and **6Hb**

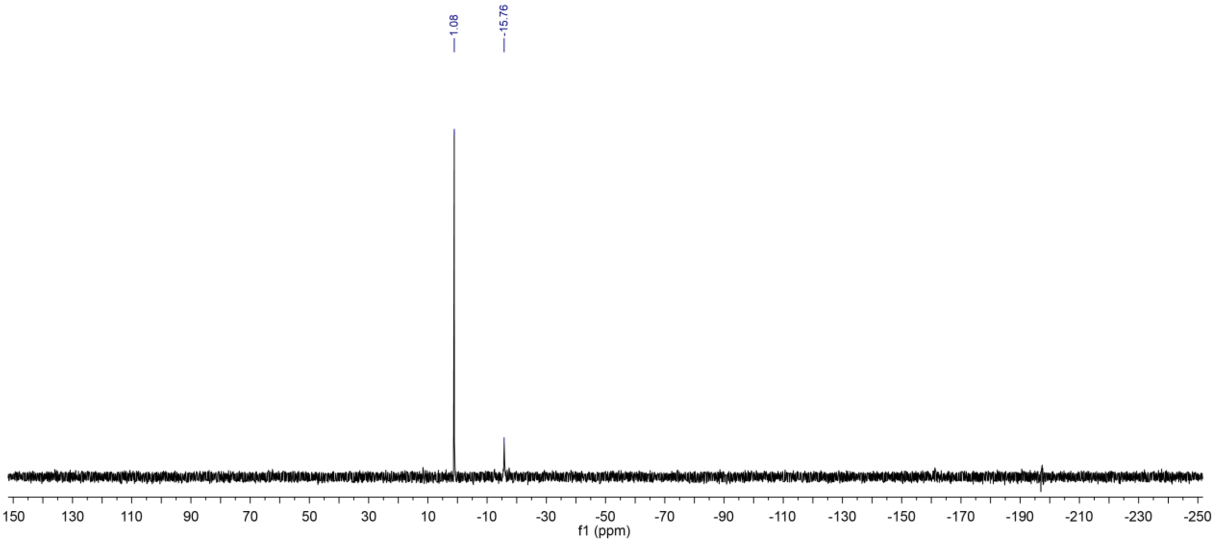

Figure S61. $^{31}P\{^{1}H\}$ NMR of the mixture of **6Ha** and **6Hb**

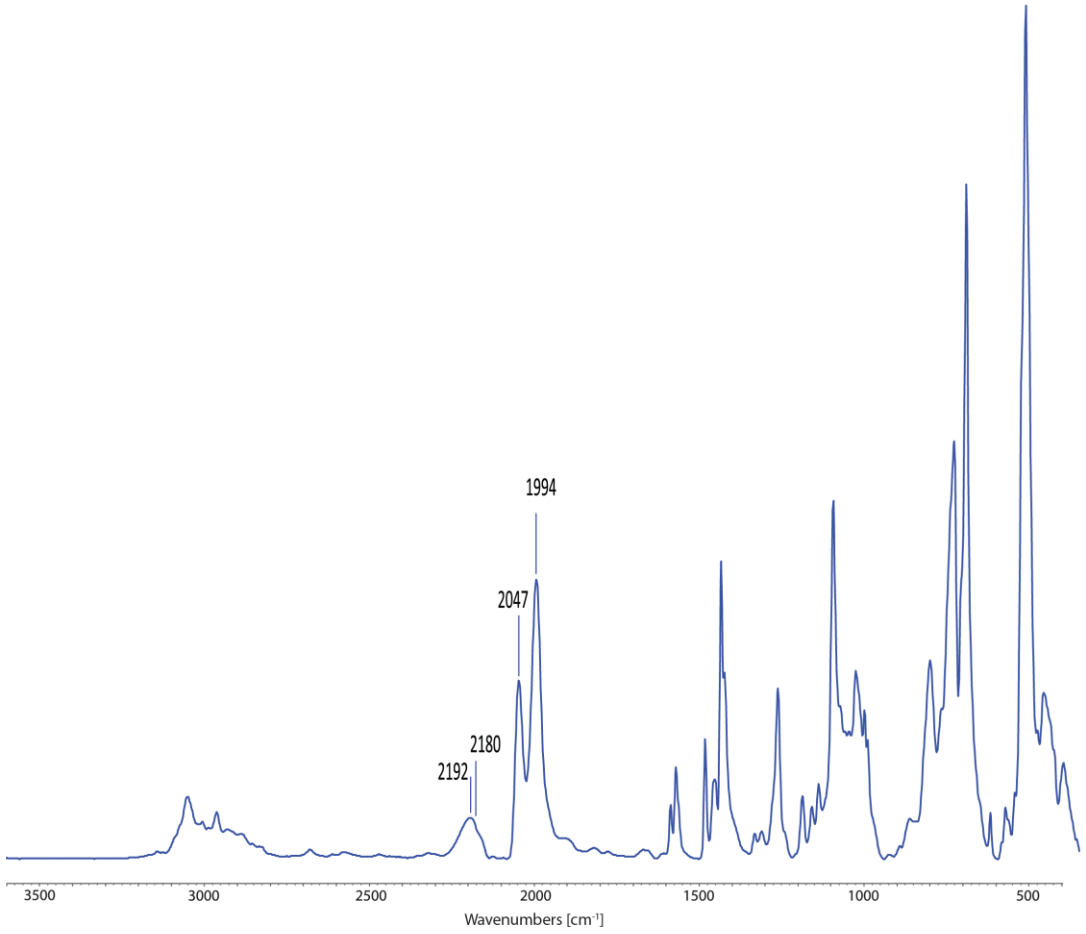

Figure S62. IR Spectrum from the mixture of **6Ha** and **6Hb**

For a better understanding of the isomers and the hydride complex formed in the reaction of **6** with methanol and KO*t*Bu, we synthesized the ^{13}CO labeled complexes of **3H₂**, **6**, **6Ha**, **6Hb**, and **3H** (note that **3H** has been reported in the literature but without ^{13}CO labeling) [5]. All of these compounds were synthesized following the same manner as their CO counterparts (Figure S61).

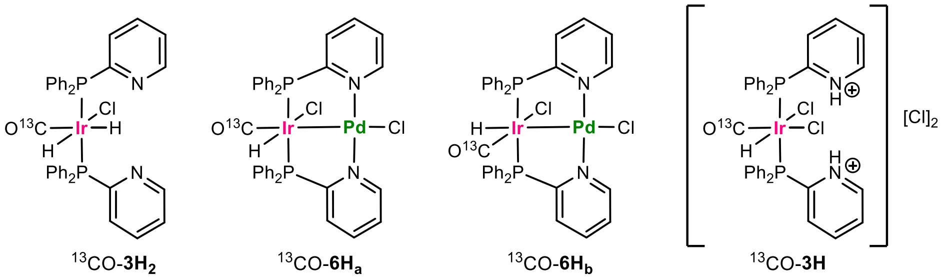

Figure S63. Structures of the ^{13}CO-labeled compounds.

In the ^{13}CO-**3H₂** NMR spectra in DCM-d₂ the $^2J_{H,C}$ values for the hydride ligands are 43.9 Hz and 3.8 Hz, corresponding to the signals at chemical shifts of -7.66 ppm and -18.65 ppm, respectively. The former value relates to the hydride ligand positioned *trans* to ^{13}CO.

For ^{13}CO-**6Ha**/^{13}CO-**6Hb**, the $^2J_{H,C}$ values for the hydride ligands are 5.2 Hz and 3.1 Hz, with corresponding $^2J_{H,P}$ values of 11.5 Hz and 15.1 Hz (chemical shifts of -15.38 ppm and -16.58 ppm in DCM-d₂). Comparing of these values with those of ^{13}CO-**3H₂** indicate that for both isomers the hydride ligand adopts a *cis* position relative to ^{13}CO.

Upon reacting ^{13}CO-**6** with methanol and KO*t*Bu, the resulting ^{13}CO-**6H$_a$**/^{13}CO-**6H$_b$** complex showed a triplet of doublet signal at -16.58 ppm, with a $^2J_{H,C}$ value of 3.1 Hz, suggesting that the product corresponds to either ^{13}CO-**6H$_a$** or ^{13}CO-**6H$_b$**.

The complex ^{13}CO-**3H**, in which the hydride ligand appears at -15.58 ppm in DCM-d$_2$, is characterized by one hydride ligand positioned *trans* to a chlorido ligand and *cis* to another chlorido ligand, with a $J_{H,C}$ value of 5.4 Hz and $J_{H,P}$ of 11.88 Hz.

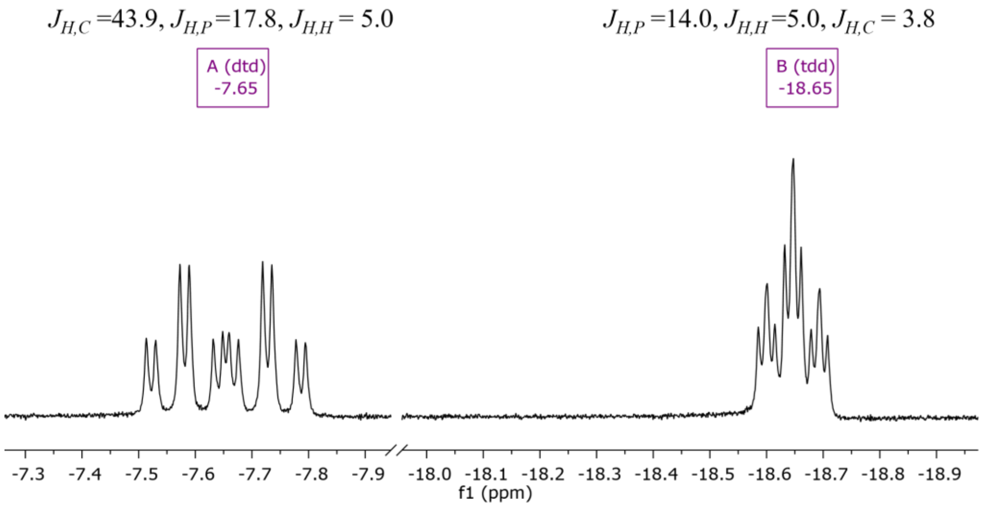

Figure S64. Hydride region of the ^{1}H NMR spectrum of ^{13}CO-**3H$_2$** in DCM-d$_2$

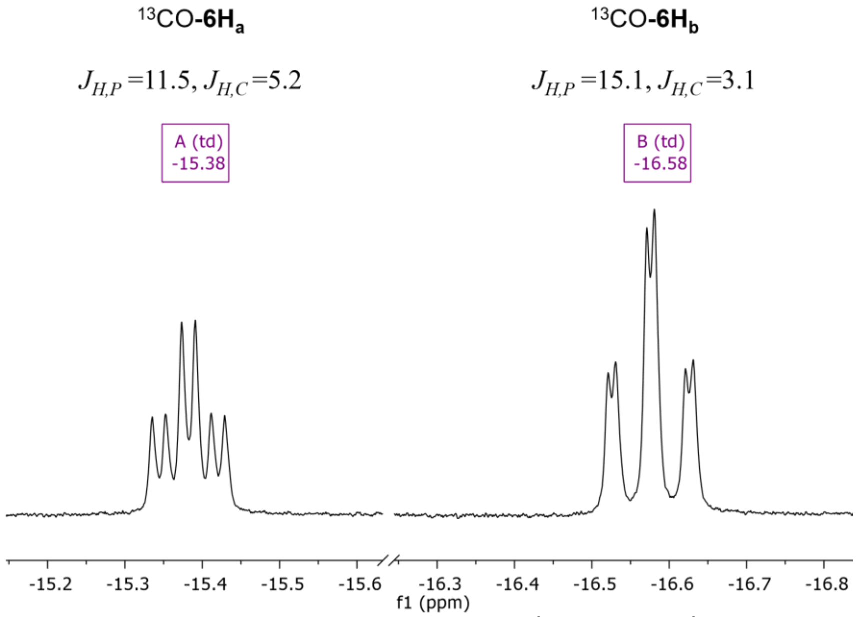

Figure S65. Hydride region of the ^{1}H NMR spectrum of ^{13}CO-**6H$_a$** and ^{13}CO-**6H$_b$** in DCM-d$_2$

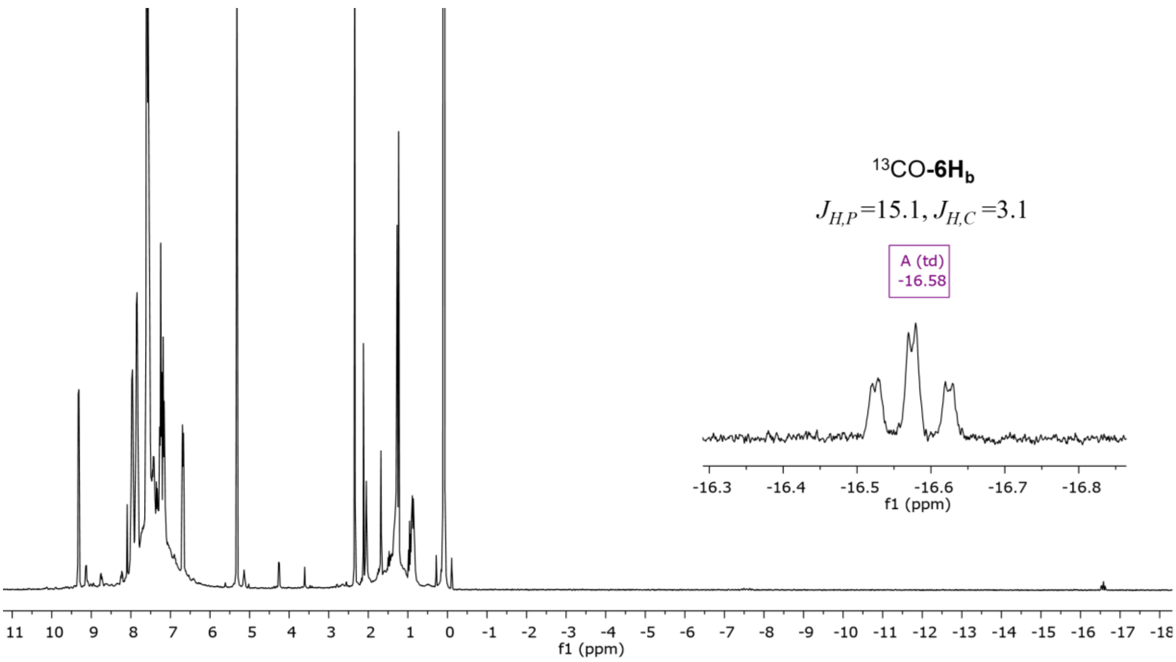

Figure S66. Hydride region of the ^{1}H NMR spectrum of ^{13}CO-**6H$_b$** formed from the reaction of ^{13}CO-**6** with MeOH and KO*t*Bu in DCM-d$_2$

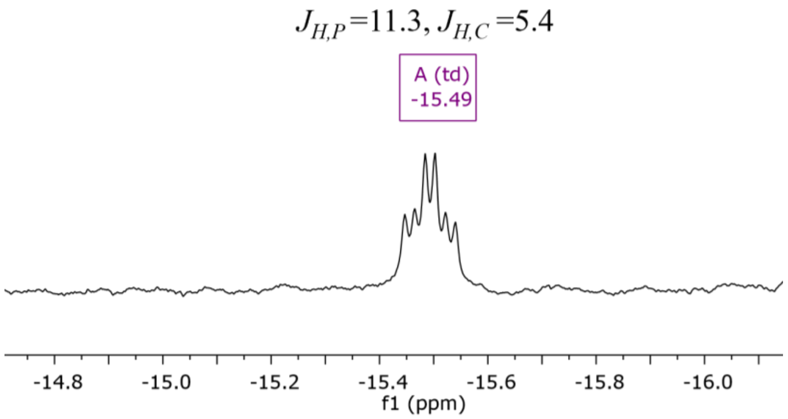

Figure S67. Hydride region of the ^{1}H NMR spectrum of ^{13}CO-**3H** formed from the reaction of ^{13}CO-**3** with HCl at room temperature within 60 min in DCM-d$_2$ and measured at -20°C

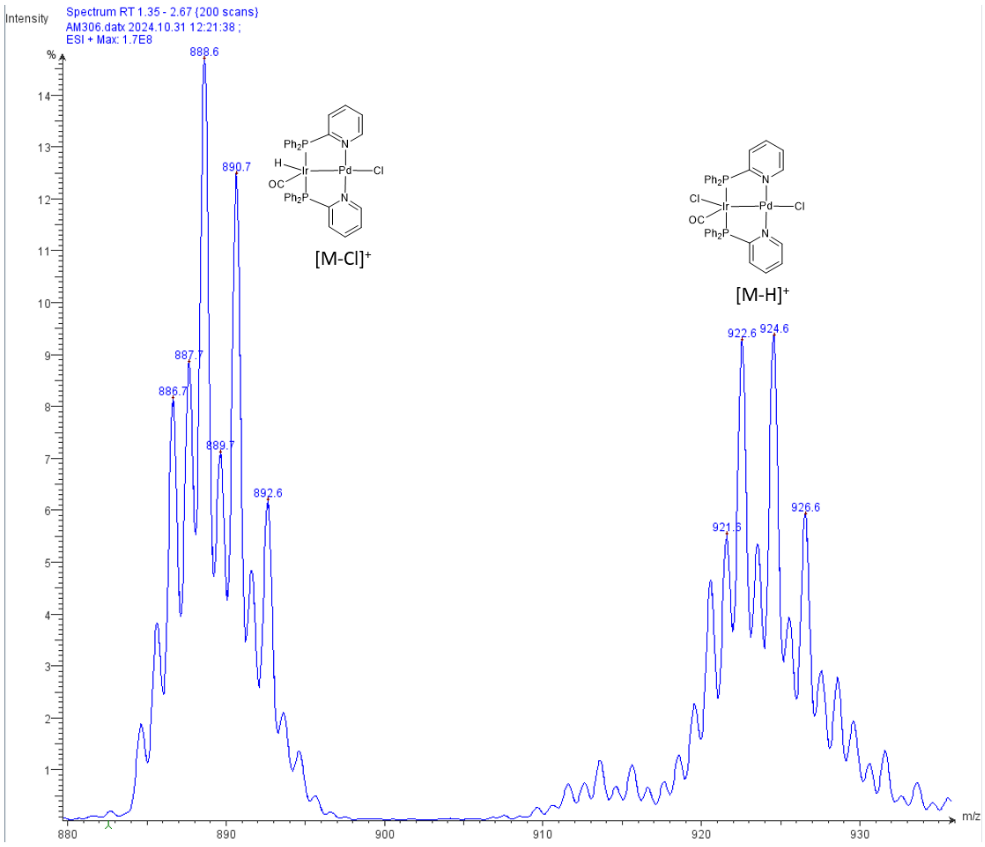

Figure S68. ESI-MS spectrum for **6H**

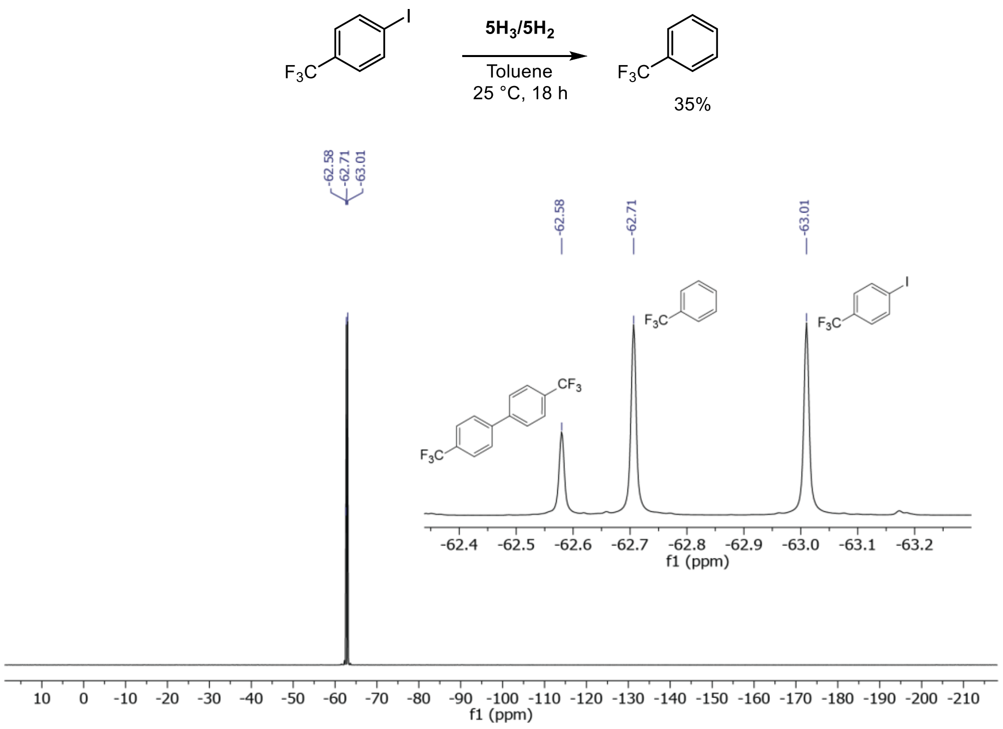

Figure S69. ^{19}F NMR spectrum of the reaction of 4-Iodobenzotrifluoride with **5H$_2$** in DMSO-d$_6$. The identity of the products was confirmed by GC-MS [9]

2. Quantum chemical calculations

Methodology. The calculations performed for this work encompass global minima searches, geometry optimizations, thermodynamical calculations and eigenmode calculations, as well as modeling properties. The foremost were conducted with the program CREST in version 2.11.1,[10, 11] with the xTB version 6.4.0. All other calculations were carried out with the ORCA program package in its version 5.0.3.[12, 13]

Where suitable, X-ray crystallographic data was used as initial input for geometry optimizations. Those were carried out using the TPSSh functional.[14] Due to the presence of heavy elements relativistic effects were modeled using the ZORA Hamiltonian and the according ZORA-def2-TZVP(-f) basis set.[15] For the chemically central elements Ir, Pd and Cl new basis sets were assigned, namely the SARC-ZORA-TZVPP, SARC-ZORA-TZVPP, and ZORA-def2-TZVPP respectively.[16,17] Dispersive effects were modeled using the Grimme's D4 correction.[18,19] For all ORCA calculations implicit solvation effects were incorporated using the CPCM approximation using the default settings for the solvent DMSO. Convergence criteria were controlled by the keywords TightOpt and TightSCF to ensure close convergence. In single cases marked by an asterisk also a slightly larger basis set ZORA-def2-TZVPP was assigned to the centrally bond CO.

The local minimum character was confirmed by the analytical calculation of the Hesse-matrix and the corresponding eigenvalues and eigenvectors. Alongside, Gibbs free energies were obtained by employing the particle in a box, rigid rotor, and harmonic approximations for contributions to the enthalpy and entropy.

Results. For the molecules **4**, **4-H**, **5**, **5-H**, **6** and **6-H** several functionals (B3-LYP, M06, M06-2X, SCAN, TPSS, TPSS0, TPSSh, wB97x-D4) were tested. The agreement of the nature of the minima was found satisfactory, such that further calculations were carried out using TPSSh. To achieve the best comparability with experimental data it was decided to rely on IR spectra over calculated Gibbs free energies, further underpinned by the often-varying number of atoms.

NMR. Calculations aiming for the NMR shielding were conducted, but the results were inconclusive. Reasons for this lie in the naive treatment of relativistic effects and complete neglect of time propagation and conformational dynamics. Readers seeking a detailed discussion are directed to the referenced source.[20]

Scaling factor. Employing the harmonic oscillator approximation to the vibrational degrees of freedom results in a systematic over estimation of absorption energies. To correct for this fact a linear scaling factor can be employed to counteract said effect. We tried to deduce a meaningful factor by comparing the intense modes between experimental and computational spectra (i. e. the CO and Ir-H stretching modes between 1750 and 2100 cm^{-1}.

Table S1. Experimental vs computational carbonyl and hydride stretching frequencies in compounds with known structure, alongside derived scaling factor.

compound	computational (un-scaled) Ir-H/Ir-CO/Ir-H	Experimental Ir-H/Ir-CO/Ir-H	scaling factor averaged
**$2H_3$**	1787/ 1961/ 2114	1787/ 1958/ 2097	1.00
**5H**	-/1959/1985	-/ 1949/ 1978	1.00
**$5H_2$**	1940/ 1998/ 2099	1947/ 1982/ 2097	1.00
**$5H_3$**	2133/ 2003/ 2169	2115/ 1997/ 2158	1.00

**6**	-/2018/-	-/ 2016/-	1.00

As can be seen from Table S1 we benefit from error compensation in such an extent that no scaling factor needs to be applied to bring the desired modes into satisfactory agreement.

Table S2. DFT calculated Ir-Pd and CO bond length.

compound	**Ir–Pd** [Å]	**C–O** [Å]
**$2H_3$**	-	1.155
**5**	2.601	1.156
**5H**	2.628	1.159
**$5H_2$**	2.644	1.159
**$5H_3$**	2.655	1.154
**4**	2.621	1.156
**6**	2.601	1.153
**$6H_a$**	2.626	1.155
**$6H_b$**	2.644	1.155

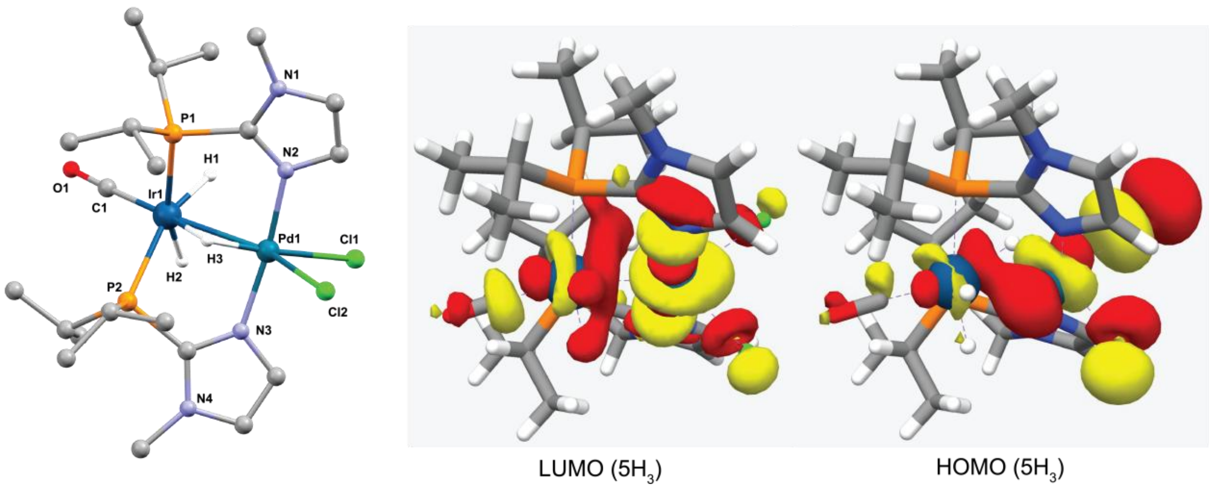

Figure S70. DFT calculated structure of **$5H_3$** and the corresponding orbital distribution of HOMO and LUMO. 0.03 was chosen as threshold for the iso-surface

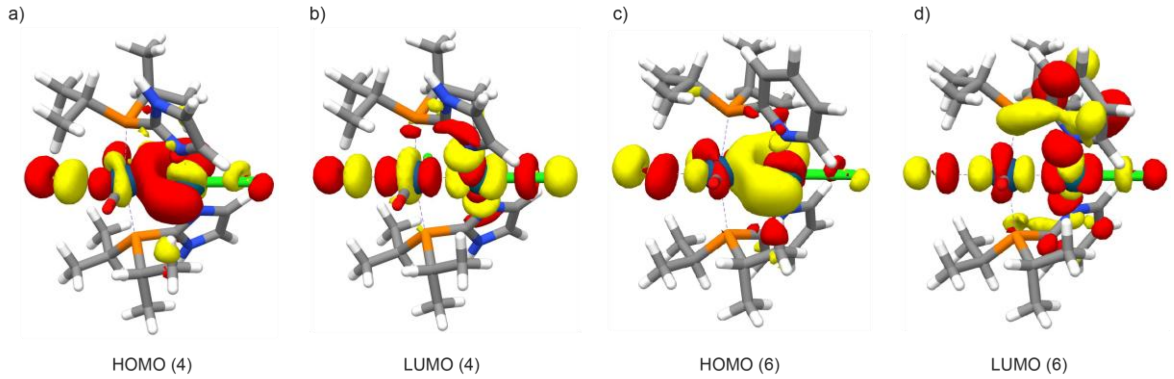

Figure S71. DFT calculated orbital distribution of HOMO and LUMO for **4** (a and b) and for **6** (c and d). 0.03 was chosen as threshold for the iso-surface

6H

The experimental IR spectrum of compounds **6H$_a$**/**6H$_b$** exhibited four peaks in the region where Ir-Hydride and CO modes are anticipated (Figure S60). The presence of only one hydride and one CO moiety in **6H** suggests the existence of multiple conformer states. In an effort to clarify, three conformers **6H$_a$**, **6H$_b$** (Figure S70, here was assumed that in **6H$_a$**, the hydride ligand is *trans* to the chlorido, while in **6H$_b$** it is *cis*) and **6H$_c$** (in which hydride ligand is in a *trans* position to CO moiety, but is ruled out by the NMR data) modeled computationally to obtain their IR-spectra and relative energies. Implicitly, it was assumed that the molecule possesses a mirror-plane, in which the metal atoms and the Phosphor atoms lie, and the positions above and below that mirror-plane are identical, reducing the number of conformers from six to three.

The corresponding theoretical IR spectra for **6H$_a$** and **6H$_b$** are shown in Figure S71. Notably, both **6H$_a$** and **6H$_b$** conformers show one peak close to 1982 cm^{-1}. Their relative Gibbs free energies (Table S3) underpin the existence of **6H$_a$**; however, they do not differ to an extend that would allow for the conclusion that **6H$_b$** is thermodynamically inaccessible.

Table S3. Relative energies of the conformers of **6H**. Energies are given in kcal/mol and in reference to the thermodynamically most stable.

	**6H$_b$**	**6H$_a$**	**6H$_c$**
ΔG [kcal/mol]	3.15	0	2.33

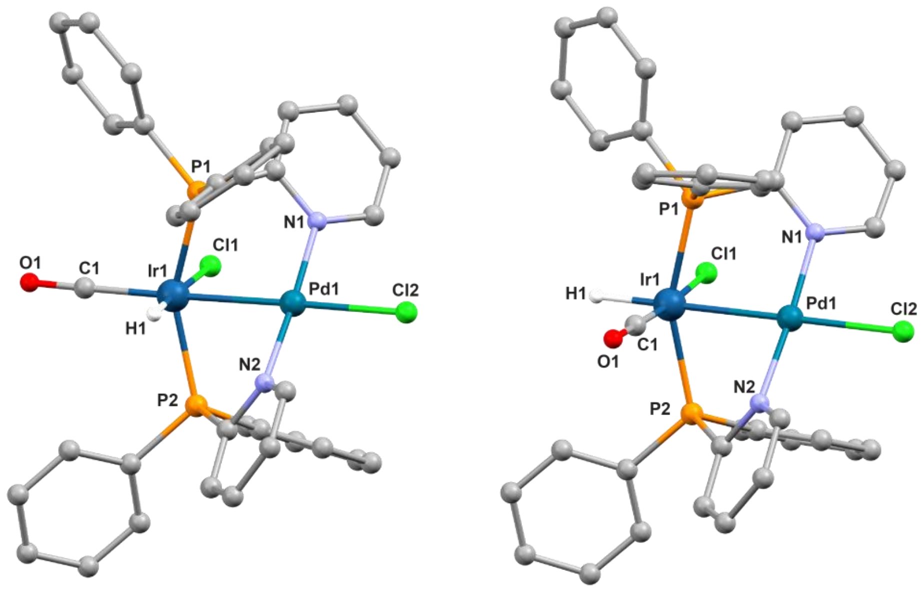

Figure S72. DFT calculated structures of **6H$_a$** (left) and **6H$_b$** (right)

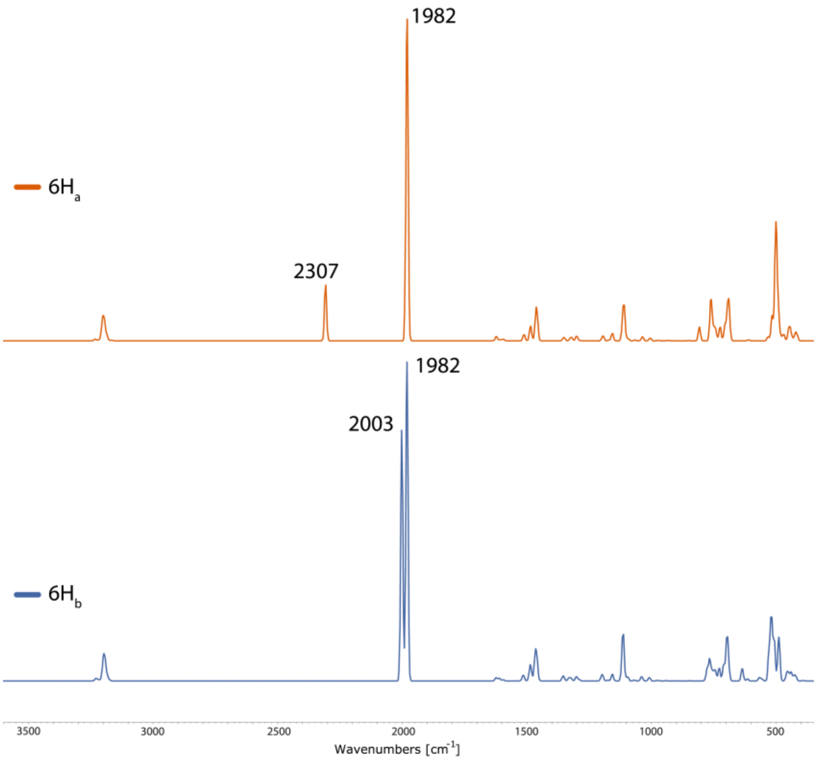

Figure S73. DFT calculated IR spectra of **6H$_a$** and **6H$_b$**

Cartesian Coordinates

4

65

G=-26632.27336811 lowest_mode=32.57

Ir	4.816431	1.571179	14.140330
C	6.076097	0.269052	13.934748
O	6.872986	-0.555707	13.796491
Cl	3.208261	3.329841	14.483084

Cl	3.484008	0.852729	12.111108
P	3.669179	0.281452	15.718575
P	6.125223	3.141954	13.008614
Pd	6.180249	2.352697	16.252641
Cl	7.434898	3.049837	18.177716
N	6.256770	4.222258	15.491132
N	6.051916	0.479703	17.002586
N	6.251321	5.839707	14.021454
N	5.129737	-1.427509	17.538459
H	6.263991	4.743043	11.252352
C	5.579317	3.902238	11.413900
C	5.763798	2.885835	10.279211
C	4.142916	4.424023	11.448932
H	5.112446	2.025620	10.439903
H	5.488091	3.359903	9.333603
H	6.794108	2.536634	10.192434
H	3.988404	5.168297	12.230833
H	3.920493	4.884719	10.481634
H	3.444511	3.605916	11.619703
H	2.625414	-1.839099	16.098082
C	2.840644	-1.278049	15.181269
C	3.772283	-2.101706	14.289768
C	1.522089	-0.957164	14.468933
H	3.284754	-3.047071	14.036402
H	3.979206	-1.563294	13.363394
H	4.719861	-2.331317	14.782405
H	0.831221	-0.403487	15.107216
H	1.714422	-0.369880	13.570079
H	1.037249	-1.892899	14.177838
H	1.759583	1.584035	16.214046
C	2.484464	1.108756	16.881185
C	1.782583	0.108038	17.802910
C	3.175235	2.194189	17.708468
H	1.107579	0.658175	18.463953
H	1.190028	-0.630365	17.262674
H	2.503750	-0.415786	18.436771
H	3.889525	1.758204	18.410548
H	3.699926	2.907900	17.073580
H	2.415872	2.730904	18.284395
C	6.217925	4.497942	14.189595
C	7.915606	2.724439	12.711280
C	6.313504	5.413375	16.167899
C	4.985769	-0.295443	16.809853
H	6.213411	6.324157	13.137964
C	6.302250	6.432355	15.257502
H	6.369843	5.441978	17.241531
C	6.892818	-0.170943	17.868630
H	4.475448	-2.194310	17.568811
C	6.328057	-1.369577	18.203425
H	7.815195	0.276598	18.193368
H	6.668295	-2.169258	18.838151
H	7.872930	1.818629	12.096966

C	8.664657	2.413165	14.008136
C	8.655820	3.827481	11.948590
H	6.324470	7.501464	15.377858
H	8.158345	1.671127	14.625581
H	9.659081	2.034781	13.756313
H	8.785221	3.315309	14.611065
H	8.632984	4.767200	12.507595
H	9.703841	3.536701	11.838559
H	8.253803	4.003624	10.951180

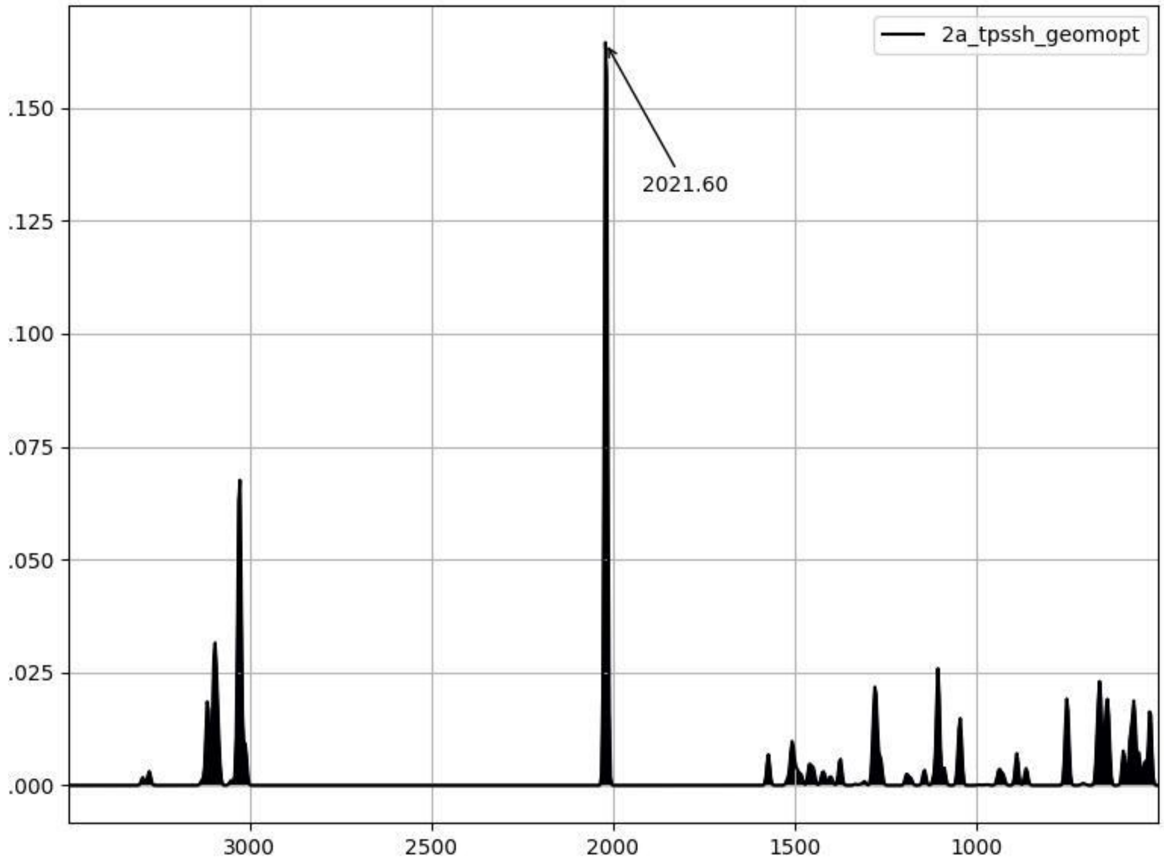

5

71

G=-26710.90184987 lowest_mode=36.33

Ir	4.846258	1.568455	14.146846
C	6.102148	0.260949	13.936152
O	6.895137	-0.577055	13.794605
Cl	3.219587	3.319101	14.472544
Cl	3.539486	0.822034	12.094930
P	6.158357	3.160887	13.027041
P	3.702170	0.277394	15.746004
Pd	6.192422	2.345856	16.252425
Cl	7.448210	3.046540	18.191031
N	6.241460	4.223258	15.505579
N	6.093932	0.461151	16.979663
N	6.273867	5.901516	14.074939
N	5.226535	-1.485602	17.548195
H	6.260627	4.684863	11.176124
C	5.614268	3.824849	11.381612

C	5.864984	2.755981	10.302711
C	4.148460	4.273464	11.372941
H	5.276420	1.859340	10.514112
H	5.547964	3.158507	9.334032
H	6.920854	2.482200	10.223407
H	3.930740	5.015979	12.144599
H	3.924353	4.709099	10.391637
H	3.490875	3.418554	11.541442
H	2.518230	-1.802950	16.034194
C	2.778374	-1.211872	15.150002
C	3.664109	-2.046671	14.216122
C	1.478702	-0.766914	14.459972
H	3.136028	-2.967155	13.942112
H	3.879521	-1.484371	13.303428
H	4.614342	-2.324628	14.684496
H	0.809071	-0.244930	15.149768
H	1.698385	-0.112282	13.612767
H	0.954949	-1.654442	14.086765
H	1.828490	1.629447	16.240027
C	2.543260	1.145400	16.915964
C	1.814378	0.172089	17.851669
C	3.256381	2.228596	17.731618
H	1.103868	0.740173	18.462726
H	1.252570	-0.600448	17.320733
H	2.519717	-0.309513	18.538120
H	3.980321	1.787924	18.424373
H	3.778691	2.936033	17.083019
H	2.508649	2.775152	18.318702
C	6.228473	4.541380	14.201013
C	7.959427	2.738856	12.763048
C	6.288359	5.389500	16.224478
C	5.031671	-0.346243	16.816070
C	6.295172	6.712283	12.855817
C	6.302482	6.436949	15.343540
H	6.326669	5.380042	17.301494
C	6.980915	-0.166578	17.815582
C	4.355357	-2.655663	17.681953
C	6.452440	-1.379620	18.166868
H	7.900974	0.308426	18.114476
H	6.831405	-2.172356	18.793189
H	7.922951	1.829609	12.149207
C	8.689567	2.428018	14.073872
C	8.714739	3.841031	12.006500
H	6.331871	7.504342	15.497940
H	8.175023	1.676607	14.678290
H	9.694486	2.059080	13.838181
H	8.789527	3.329780	14.685361
H	8.730422	4.767095	12.592143
H	9.754595	3.526055	11.864828
H	8.293606	4.052867	11.020973
H	3.363022	-2.350173	18.014335
H	4.798366	-3.309524	18.432661

H	4.285373	-3.189468	16.732883
H	7.165310	6.459141	12.248340
H	6.361383	7.756888	13.158512
H	5.380047	6.559201	12.283617

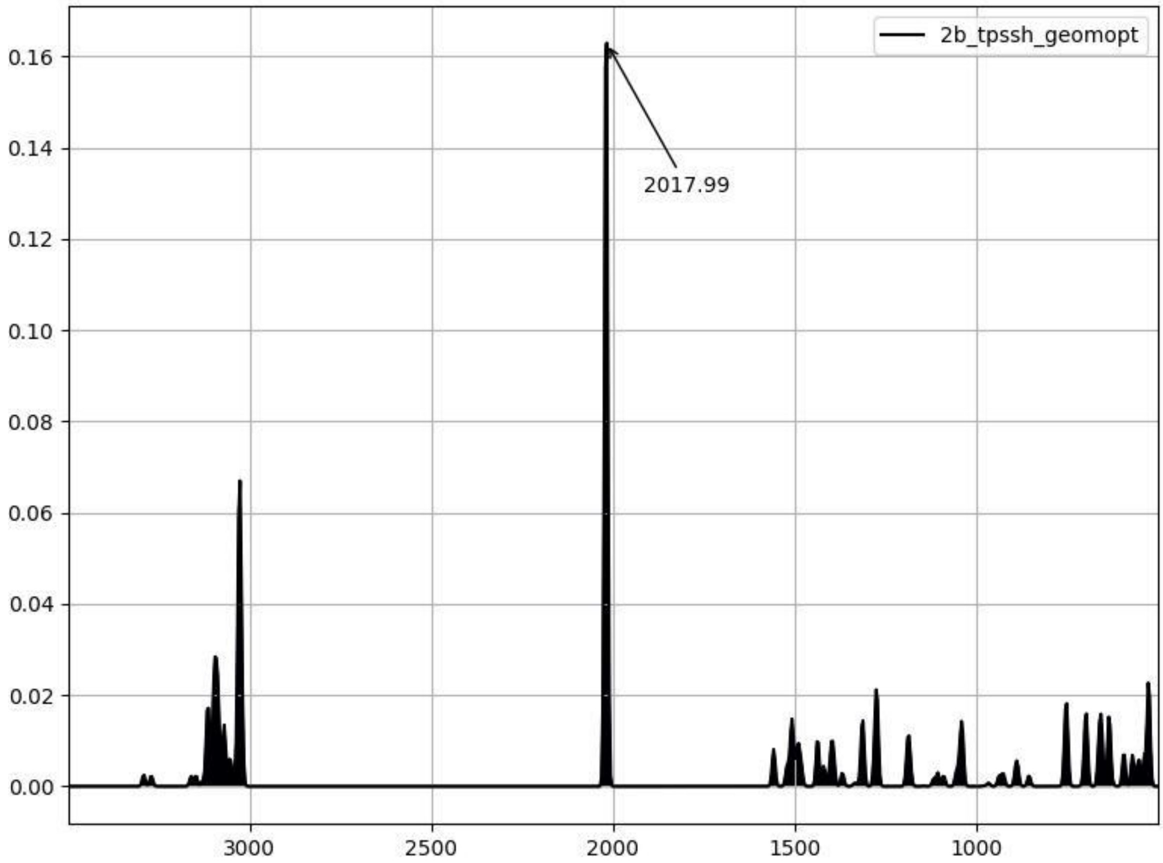

5H:
71
G=-26248.95004945 lowest_mode=29.99

Ir	4.86654017700595	1.60089861371843	14.19349558045870
C	6.11282483799576	0.29727027792746	13.93751757456833
O	6.90584305381388	-0.53157349874835	13.77519844242437
Cl	3.24096747412637	3.36805003632099	14.56552114620140
H	3.92516010460610	1.17668632360052	12.90997954829982
P	6.14174084752340	3.17073541537243	13.06338345234275
P	3.73421980727385	0.29311189584065	15.74268767297350
Pd	6.24432277283224	2.37680868697098	16.31705218145201
Cl	7.55204321948816	3.08211935694991	18.30774407358002
N	6.32232572888055	4.24196465438946	15.51617418421986
N	6.08275201032532	0.48280300034491	17.03197907522915
N	6.32706419093081	5.90945355855502	14.07752497459020
N	5.19853030631360	-1.45675096638315	17.58680073713343
H	6.14993862159167	4.59559946915955	11.10988183881042
C	5.52110933689828	3.75844461622252	11.42122268251344
C	5.69391867754821	2.60749521477695	10.42106429620481
C	4.05961533890173	4.20040949948480	11.45985770281615
H	5.16462504233847	1.71889211802084	10.77470065329758
H	5.26378604638124	2.90194227824861	9.46051532523423
H	6.74100640124568	2.34813969871793	10.25675940719400

H	3.87521773160761	4.98838367688653	12.18981658224388
H	3.77646454663902	4.56464287610449	10.46777656299039
H	3.41789613975825	3.35940088337644	11.72404280378491
H	2.48846345274943	-1.77824650780718	15.89091486931404
C	2.84236181624464	-1.17131971458262	15.05488771553242
C	3.77098859408588	-2.00443766083139	14.17048766656947
C	1.63417635882368	-0.67311385095854	14.25427507826686
H	3.25303488428069	-2.91248607105446	13.85053553335849
H	4.04989946328461	-1.43708090779903	13.28068613238660
H	4.68949861553812	-2.29706922543968	14.68411417737110
H	0.90674137521335	-0.15627738188401	14.88290203553940
H	1.95976394907596	0.01314837737068	13.46867606308245
H	1.13450988717729	-1.52471445912546	13.78520643673857
H	1.86386406309842	1.65433785999157	16.19629446549772
C	2.52806991255471	1.12098330728672	16.88214747728307
C	1.72400395681419	0.13282442481550	17.72960020430686
C	3.22478216712525	2.14462563042047	17.77883493659590
H	0.99878524717365	0.69217055065279	18.32687233472653
H	1.17182266339547	-0.59485790083171	17.13358804926369
H	2.37381513089656	-0.40115163259196	18.42791343684792
H	3.87019177380511	1.64881690034457	18.50756990091111
H	3.83244097376116	2.83804852725965	17.19594305727874
H	2.46470860899833	2.71173137473358	18.32402526555995
C	6.25680809588550	4.55737566963004	14.21796530799028
C	7.92334307614781	2.74306646452679	12.73135834968093
C	6.43132049039635	5.40932820739593	16.21941544336095
C	5.03601599592976	-0.32951958468107	16.83753366025392
C	6.28988031796028	6.69934687792698	12.85278209724662
C	6.42734366973456	6.45004961241036	15.33538718734987
H	6.52258927893813	5.40331245800196	17.29158314492502
C	6.93120320586375	-0.13504587829467	17.90825036654288
C	4.33518652987868	-2.62937651060100	17.66789474621253
C	6.39422584146307	-1.34297628249452	18.25081560353171
H	7.83298300938390	0.34760121829279	18.24193395027368
H	6.74928070679960	-2.12613380362168	18.89904530390789
H	7.86191260907390	1.81622277739327	12.15118670969374
C	8.70594267977898	2.47000452192090	14.01638679793832
C	8.64388688686468	3.81108532079885	11.90493741945127
H	6.48636279913437	7.51510341418811	15.48208668871725
H	8.20760472867422	1.74925291307413	14.66552535909790
H	9.69445558728397	2.08378964819329	13.75212283877142
H	8.83945143391055	3.38838719994816	14.59143494208092
H	8.68459912609286	4.75796693752372	12.44990309856667
H	9.67471824364831	3.49089787208995	11.73135537224960
H	8.18169584740636	3.98432279768857	10.93252702026457
H	3.30932426435157	-2.32870113459993	17.86649912391812
H	4.68892425013728	-3.24573059973375	18.49098029512158
H	4.38173447938083	-3.20245949661452	16.74261626490458
H	7.10146228337269	6.40727614336211	12.18859927588350
H	6.41539859265217	7.74343989931325	13.12898719754248
H	5.33329066173163	6.57473401113402	12.34903509952295

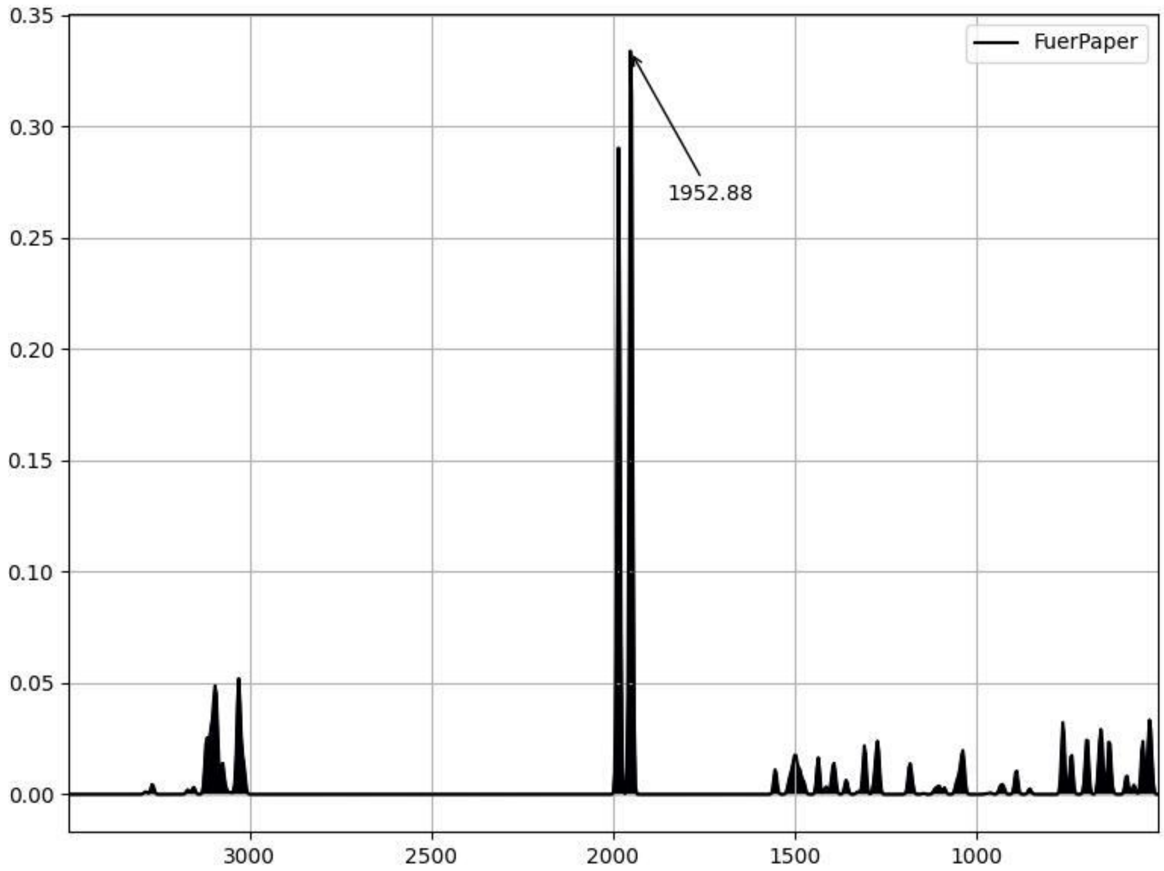

6:

73

G=-27129.34684213 lowest_mode=-81.04

Cl	8.719234	5.168201	8.387564
Ir	9.543695	7.434318	8.345584
Pd	7.152727	8.412224	8.042459
P	8.889110	7.573972	10.567960
P	9.382986	7.471686	6.021768
Cl	11.860274	6.482056	8.634209
Cl	4.955070	9.366032	7.781482
N	6.473635	7.153781	9.483671
N	7.883989	9.626157	6.583495
C	7.202597	6.885649	10.590502
C	9.762533	6.672041	11.874578
C	8.743726	9.273047	11.174335
C	5.275005	6.569158	9.342951
C	4.757916	5.708776	10.299812
H	4.732852	6.811615	8.441190
C	5.486600	5.460192	11.452614
H	3.792585	5.250210	10.130729
C	6.731885	6.060489	11.597244
H	5.103823	4.800729	12.221629
H	7.345900	5.875933	12.467920
C	9.800175	7.145219	13.189103
C	10.383410	5.463217	11.549971
C	10.457711	6.412123	14.170334
H	10.487855	6.783066	15.188128

C	11.075548	5.207251	13.845236
H	9.320185	8.080488	13.447995
C	7.512305	9.922332	11.249641
C	9.918502	9.962303	11.487000
C	7.457622	11.257314	11.636500
H	6.597985	9.396219	11.006564
C	8.627334	11.943008	11.950027
H	6.499087	11.759297	11.693214
C	9.857383	11.293124	11.878328
H	8.581974	12.983058	12.251271
H	10.770250	11.823333	12.123003
H	10.878375	9.461789	11.424132
C	8.740098	9.140650	5.653284
C	8.202188	6.318437	5.288716
C	10.872008	7.365482	4.990859
C	7.476856	10.901184	6.489734
C	7.896720	11.733041	5.462995
H	6.797720	11.238418	7.258319
C	8.745049	11.230734	4.487890
H	7.552417	12.758540	5.440040
C	9.173993	9.912355	4.588465
H	9.081816	11.854735	3.669392
H	9.855629	9.489950	3.863033
C	6.907616	6.720185	4.962049
C	8.586714	4.986561	5.113708
C	6.003330	5.793859	4.453715
H	6.599452	7.748882	5.099829
C	6.389376	4.469359	4.270272
H	4.999361	6.110611	4.197285
C	7.682405	4.067919	4.597586
H	5.684268	3.749563	3.871030
H	7.985929	3.037092	4.457454
H	9.588145	4.669296	5.376599
H	9.910590	6.488803	3.270757
C	10.836598	6.864297	3.686748
H	10.358338	5.109129	10.528063
C	11.034623	4.732805	12.536647
H	11.590996	4.639664	14.611594
C	12.071003	7.856607	5.513011
H	11.518338	3.797023	12.281525
C	11.994531	6.851517	2.917309
H	12.106033	8.223534	6.530051
C	13.224020	7.848923	4.737331
H	11.963779	6.458948	1.907670
C	13.187718	7.342878	3.440879
H	14.151810	8.228383	5.149265
H	14.089307	7.328896	2.839262
C	10.176418	9.150764	8.346604
O	10.597986	10.223579	8.339317

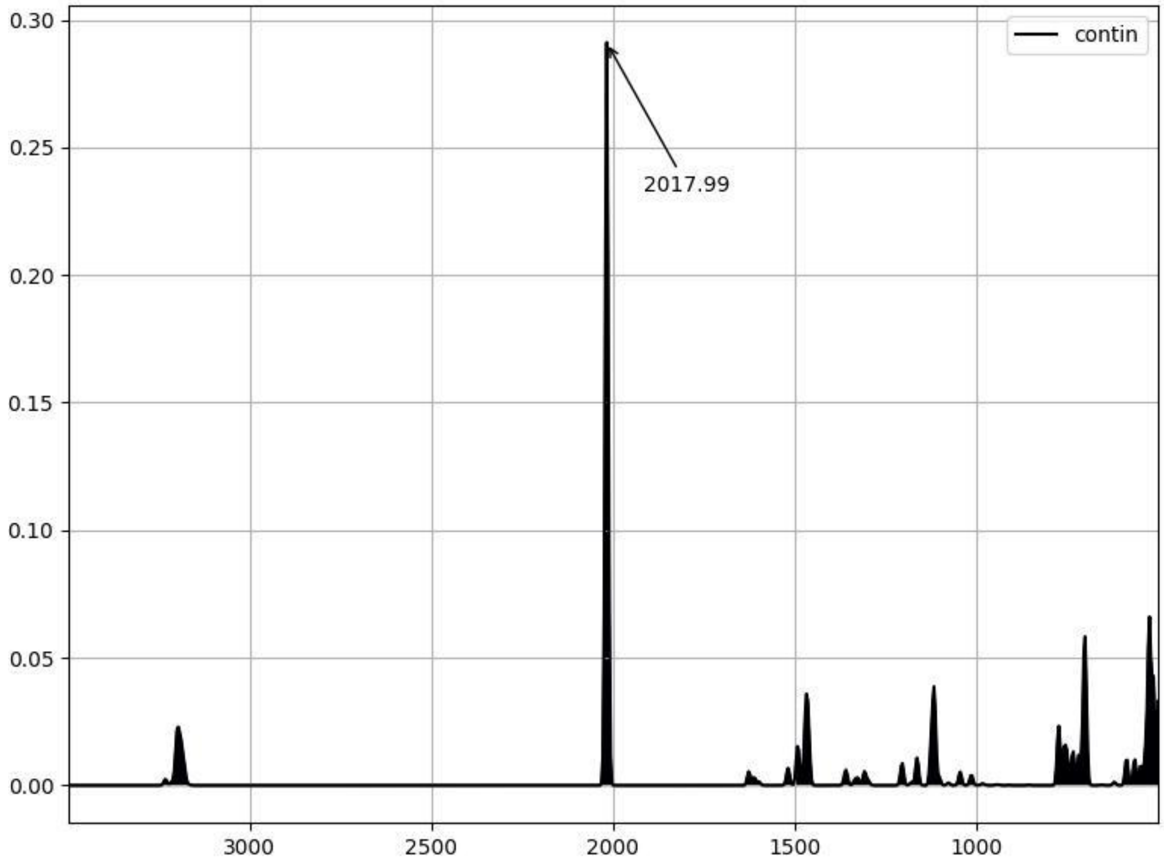

5H$_3$:

72
G=-25787.44221897 lowest_mode=26.97

Ir	6.289521	1.052272	14.627448
C	6.535478	0.009885	13.110955
O	6.710995	-0.613873	12.158882
H	7.853122	1.461908	14.596643
H	6.828547	-0.161939	15.548947
H	5.521262	2.191386	15.763492
P	6.259569	3.109111	13.456877
P	4.258173	0.170526	15.510908
Pd	6.595408	2.319758	16.970758
Cl	7.788943	2.908218	18.941240
N	7.129797	4.006727	15.970659
N	5.704297	0.709655	17.829053
N	7.062414	5.653010	14.520796
N	4.304584	-0.942018	18.194096
H	5.050888	4.582118	11.979246
C	4.734900	3.804891	12.679176
C	4.047113	2.676167	11.902513
C	3.800527	4.398705	13.730918
H	3.696421	1.903877	12.589918
H	3.186598	3.082020	11.365392
H	4.710144	2.202266	11.176147
H	4.246882	5.242140	14.259769
H	2.885107	4.744377	13.244471
H	3.526791	3.644242	14.467617

H	2.946778	-1.841022	15.463570
C	3.782075	-1.486381	14.857929
C	4.934823	-2.486038	14.955156
C	3.290202	-1.320324	13.415054
H	4.566984	-3.476232	14.675253
H	5.745013	-2.221453	14.276109
H	5.350457	-2.546113	15.962836
H	2.431020	-0.651040	13.347213
H	4.077135	-0.929161	12.769142
H	2.990216	-2.298082	13.030859
H	2.617058	1.607467	14.609773
C	2.697801	1.183761	15.614856
C	1.422942	0.393971	15.919095
C	2.855319	2.317954	16.631808
H	0.570488	1.071353	15.821129
H	1.262750	-0.442711	15.239978
H	1.426125	0.024160	16.945936
H	2.876867	1.917713	17.647854
H	3.762510	2.904252	16.480159
H	1.998867	2.991329	16.547183
C	6.814113	4.329049	14.698693
C	7.601984	3.062442	12.143629
C	7.578942	5.142746	16.589067
C	4.750156	-0.059046	17.261960
C	6.804793	6.463820	13.332913
C	7.538380	6.169369	15.693040
H	7.887982	5.132955	17.618913
C	5.860186	0.299350	19.124645
C	3.308236	-2.006833	18.060813
C	4.998214	-0.730361	19.355651
H	6.565090	0.775983	19.781898
H	4.815643	-1.328119	20.232609
H	7.670860	1.992272	11.939932
C	8.948345	3.505783	12.721390
C	7.286010	3.754430	10.817020
H	7.800718	7.209854	15.783625
H	9.164652	3.008282	13.669050
H	9.738415	3.247199	12.012367
H	8.980878	4.584801	12.882582
H	7.204146	4.837822	10.908274
H	8.106093	3.545738	10.124824
H	6.367302	3.379478	10.366101
H	2.409107	-1.625209	17.588166
H	3.063758	-2.347078	19.063525
H	3.717699	-2.836167	17.487234
H	7.182982	5.959523	12.450022
H	7.329958	7.407224	13.455598
H	5.736567	6.649598	13.232161

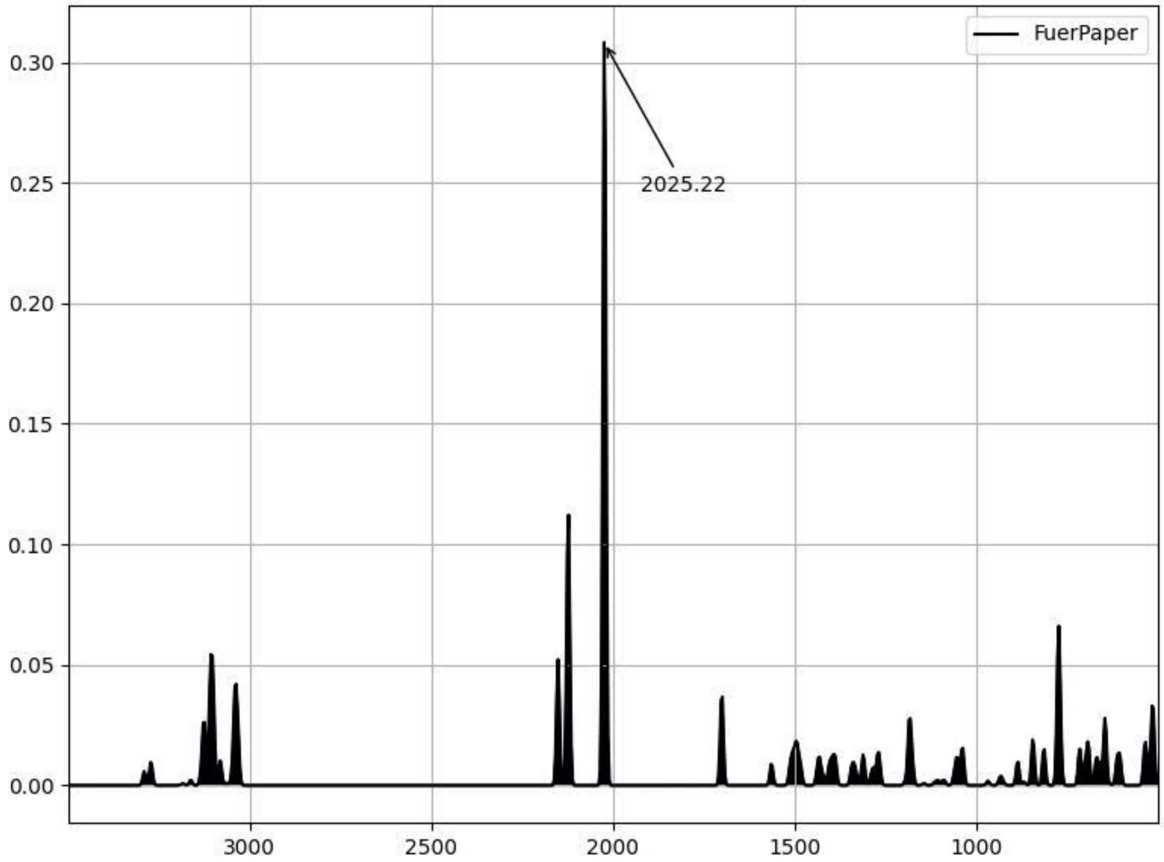

2H3

Ir	4.969587	1.674388	14.351113
C	5.927715	2.069400	15.830297
O	6.545375	2.323847	16.785011
P	6.025265	3.407713	13.207957
P	3.879901	-0.028876	15.509835
N	8.460312	2.593874	14.112090
N	3.407705	1.652209	17.601186
N	8.503209	4.798395	13.843052
N	3.587202	-0.420622	18.378682
H	5.673226	5.800289	12.977654
C	5.117688	4.998160	13.464843
C	3.735359	4.898586	12.812142
C	4.993665	5.289026	14.963112
H	3.169616	4.066848	13.238668
H	3.184749	5.825120	12.995859
H	3.802510	4.751963	11.732611
H	5.960961	5.280688	15.471198
H	4.538994	6.272537	15.109279
H	4.360291	4.535854	15.437442
H	4.335455	-2.358110	16.040167
C	4.840173	-1.606088	15.433070
C	6.256262	-1.375356	15.968105
C	4.877261	-2.095674	13.982053
H	6.797036	-2.325167	15.992818
H	6.793501	-0.683485	15.315356
H	6.257278	-0.954031	16.976269
H	3.881486	-2.339798	13.607154
H	5.312414	-1.332961	13.331815
H	5.493444	-2.996827	13.923037
H	2.293303	-0.664590	13.883040
C	2.163311	-0.430466	14.943121
C	1.537892	-1.623363	15.662791
C	1.281234	0.814620	15.057557
H	0.557954	-1.833442	15.224558

H	2.142526	-2.527882	15.573737
H	1.383647	-1.404255	16.722477
H	1.131922	1.089917	16.103898
H	1.727345	1.663216	14.536250
H	0.303315	0.607027	14.614151
C	7.738368	3.659392	13.770528
C	6.205615	3.203763	11.376454
C	9.709796	3.056484	14.411974
C	3.648689	0.385549	17.267771
C	8.148775	6.192177	13.582713
C	9.751407	4.416792	14.257496
H	10.502451	2.394598	14.725240
C	3.191762	1.660407	18.949562
C	3.800489	-1.860379	18.511238
C	3.305285	0.389097	19.446552
H	2.973280	2.570926	19.486016
H	3.223513	-0.014341	20.442716
H	5.178177	3.050888	11.034846
C	7.006385	1.934812	11.077750
C	6.807453	4.421900	10.679411
H	10.532800	5.143841	14.408076
H	6.566036	1.067485	11.572576
H	7.013769	1.755900	9.998937
H	8.040020	2.038443	11.415220
H	7.837179	4.589296	11.005428
H	6.827488	4.245957	9.600049
H	6.229783	5.331018	10.857392
H	3.128532	-2.407252	17.854202
H	3.584536	-2.131087	19.542016
H	4.834145	-2.116050	18.284796
H	7.772231	6.307520	12.569010
H	9.053053	6.785595	13.693653
H	7.405501	6.537261	14.298840
Cl	3.712185	1.148462	12.395052

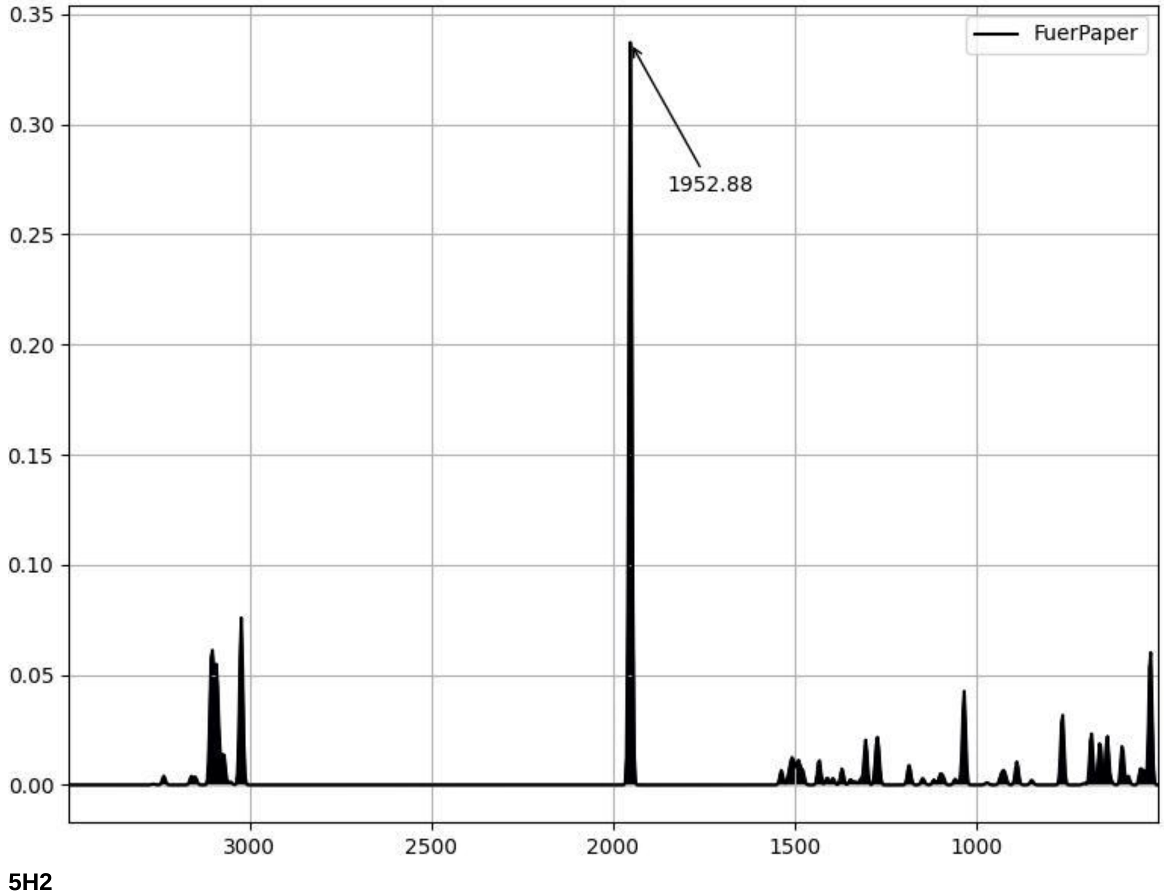

5H2

Ir	4.632605	1.848085	14.348184
C	3.378781	3.219405	14.706990
O	2.621783	4.063814	14.943959
P	6.031509	3.198753	13.124045
P	3.706816	0.281650	15.746774
Pd	6.111233	2.611568	16.402478
Cl	7.461932	3.408973	18.432171
N	6.265449	4.430993	15.511195
N	5.969637	0.725166	17.158178
N	6.592989	5.957067	13.955114
N	5.275309	-1.313393	17.628406
H	6.086816	4.594727	11.147360
C	5.382595	3.849096	11.519565
C	5.299138	2.705472	10.503898
C	4.012887	4.498761	11.720932
H	4.680859	1.892908	10.893672
H	4.840042	3.076571	9.584020
H	6.282723	2.304164	10.253105
H	4.020168	5.253152	12.510926
H	3.699385	4.980044	10.791008
H	3.272200	3.741587	11.984507
H	2.808538	-1.955734	15.767261
C	3.104972	-1.277991	14.964711
C	4.208938	-1.930651	14.132199
C	1.872592	-0.971731	14.108163
H	3.851960	-2.889220	13.746287
H	4.476416	-1.290670	13.290013
H	5.115039	-2.111599	14.715380
H	1.053074	-0.558143	14.699233
H	2.124076	-0.260408	13.318344
H	1.521384	-1.895898	13.642100
H	1.632043	1.331321	16.138821
C	2.327377	0.866814	16.846397
C	1.603102	-0.240923	17.611016
C	2.836296	1.935774	17.816447
H	0.766076	0.197470	18.161747
H	1.201527	-1.016761	16.957838
H	2.266895	-0.704320	18.344898
H	3.443465	1.476307	18.600353
H	3.451986	2.685237	17.315541
H	1.984456	2.431470	18.289271
C	6.327766	4.640940	14.187185
C	7.716627	2.534274	12.723801
C	6.485214	5.632769	16.128837
C	5.021290	-0.187426	16.904009
C	6.729919	6.655610	12.678604
C	6.682246	6.585136	15.170223
H	6.507235	5.716939	17.200900
C	6.845739	0.166546	18.051539
C	4.544071	-2.578830	17.652841
C	6.424970	-1.098887	18.342768
H	7.689293	0.719305	18.425312
H	6.833084	-1.861419	18.984753
H	7.488747	1.637969	12.136602
C	8.475946	2.114921	13.981033
C	8.564078	3.492529	11.885651
H	6.874573	7.642714	15.239245
H	7.883901	1.438671	14.598580
H	9.404401	1.616243	13.687736
H	8.734411	2.988866	14.584561
H	8.818169	4.387138	12.460117
H	9.502013	2.997656	11.619648
H	8.073808	3.795542	10.959690
H	3.485716	-2.400041	17.821615
H	4.940560	-3.169187	18.474918
H	4.689146	-3.118419	16.718190
H	7.446218	6.140795	12.043697
H	7.097173	7.656568	12.891028

H	5.765236	6.724842	12.178838
H	5.748426	0.669743	14.086359
H	3.889356	1.232010	13.028201

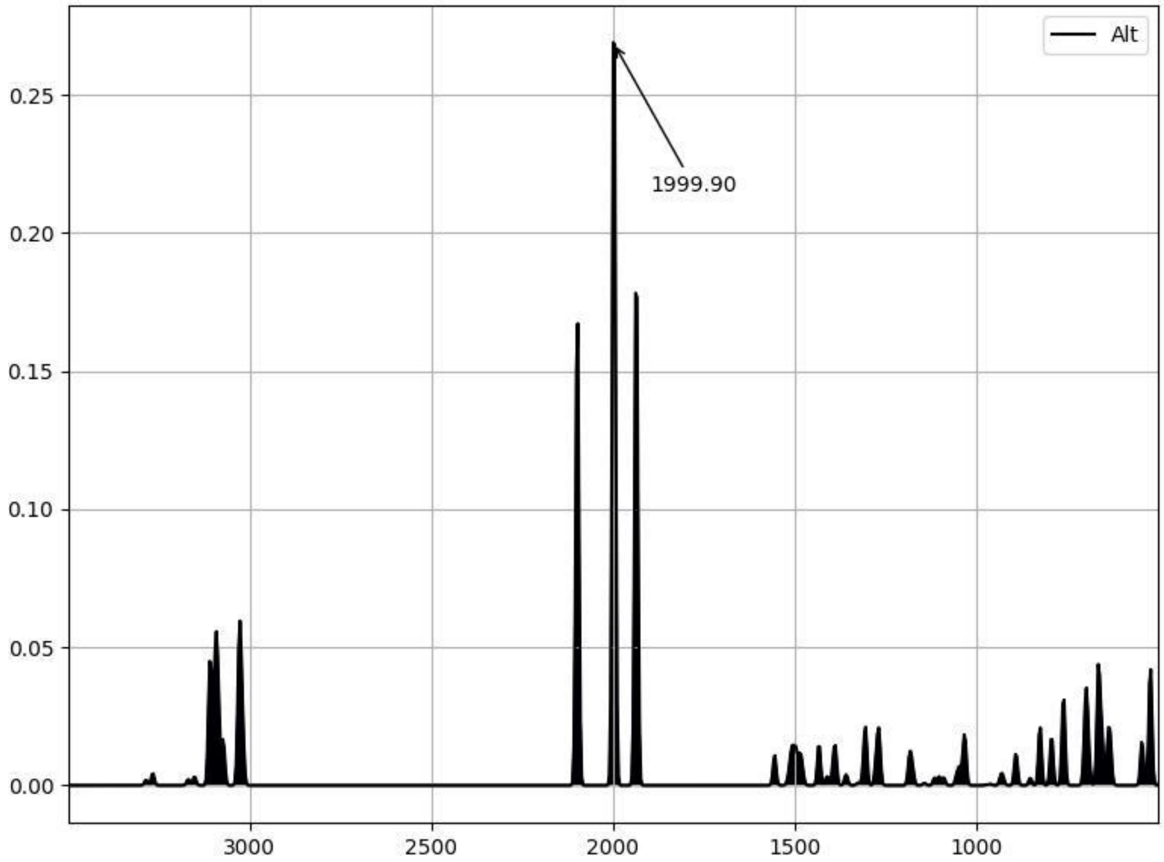

6Ha

Cl	-0.11440967127687	1.29936095359028	2.53435449671514
Ir	0.03363483640621	0.71384731963808	0.09144755195645
Pd	-0.11242854965850	-1.87204049023165	0.52305639195957
P	-2.20196425440181	0.20303738006297	0.04440434946489
P	2.25137725372045	0.08707096554481	0.16029430101262
C	0.06036990838576	2.52353251316141	-0.40987546648838
Cl	-0.20949478307378	-4.31055716299382	0.95083398326770
N	-1.55119733273735	-1.53522707460235	1.91545362481417
N	1.28461482478606	-2.13747313985887	-0.93007682203119
C	-2.53413748467391	-0.65711955457262	1.61508314903436
C	-3.42077199493260	1.54925161271078	0.01734360631326
C	-2.73161515182140	-0.97249776464375	-1.23091259150157
C	-1.58734517906379	-2.17576368259402	3.09030544518845
C	-2.60828060972559	-1.96524578683396	4.00660107262470
H	-0.78079437898033	-2.86925029972446	3.27978916288007
C	-3.63050544384569	-1.08108903945718	3.69327701406059
H	-2.59215710875019	-2.49719134700378	4.94883247802183
C	-3.59183930393287	-0.41446845875375	2.47266375289664
H	-4.44185246471440	-0.90451924462938	4.38868259894182
H	-4.35919541176727	0.29446076913997	2.19275741094701
C	-4.60718888156632	1.47389213890437	-0.71308886401373
C	-3.13537951088005	2.69377777803777	0.76801030100155
C	-5.50383574112389	2.53819584705834	-0.68909405265700
H	-6.42220522728696	2.48010845777697	-1.26175020720191
C	-5.22216503123344	3.67252598690116	0.06665690911373
H	-4.83026281581487	0.59188889561950	-1.30082153130272
C	-3.60011677149445	-2.02336440165328	-0.92955445898127
C	-2.25577112747125	-0.81800811410552	-2.53480134900656
C	-3.97365429590701	-2.92228732162163	-1.92261739506394
H	-3.97676315743422	-2.14987500458884	0.07809496560150
C	-3.49410022209724	-2.76910095643930	-3.21994720393863

H	-4.63861557278560	-3.74297307056331	-1.68011849313313
C	-2.63967130445070	-1.71174134265287	-3.52572459357108
H	-3.78401155114707	-3.47252480518310	-3.99174651495713
H	-2.26487978099007	-1.58859883443108	-4.53508324327377
H	-1.57460219659589	-0.00847090422685	-2.76764392869266
C	2.37037158121539	-1.33231523145291	-0.98122605294091
C	2.90257060981771	-0.52173174902635	1.73632914655492
C	3.53093354743573	1.21325672783021	-0.47660490360406
C	1.18127598234773	-3.14905075353554	-1.80457549545392
C	2.16013301410217	-3.39905274808742	-2.75493658912180
H	0.29575030172672	-3.76184702239108	-1.71844467392408
C	3.29148660344083	-2.59676837333546	-2.78968751565964
H	2.02673975938393	-4.21858466796404	-3.44888713559767
C	3.39679588842846	-1.54488864880834	-1.88602530504974
H	4.07589832920013	-2.77728913201096	-3.51433578416211
H	4.25386009056864	-0.88531418610653	-1.89293223402908
C	3.12126685175842	-1.88148882110728	1.96029256107596
C	3.12737107737602	0.39795177671254	2.76377705715465
C	3.56633949440198	-2.31773934852469	3.20348090478678
H	2.94545106704686	-2.60329252365911	1.17294520180365
C	3.79537209974445	-1.39982358909868	4.22465047822090
H	3.73543030177286	-3.37496924606373	3.37144019112541
C	3.57912484670687	-0.04203237764551	4.00187883885392
H	4.14317925529298	-1.74160173841135	5.19270123197630
H	3.75648256737585	0.67533963399516	4.79463990903313
H	2.95114863001105	1.45281092518811	2.59581427794983
H	5.09856155523166	0.58762912373122	0.86254497998011
C	4.83022414329345	1.23083082532800	0.03418029945023
H	-2.20994421662161	2.74691320092678	1.33183139228522
C	-4.03826858158584	3.74839793931774	0.79825266558518
H	-5.92272233189749	4.49956012700334	0.08321824251199
C	3.20095623090886	2.03812896995960	-1.55462394832393
H	-3.81633845754502	4.63219058012744	1.38516532204237
C	5.78487268524467	2.07589333167157	-0.52286264165115
H	2.19675665508235	2.01793990216797	-1.96083884036956
C	4.15908139305915	2.87291542593769	-2.11573567026606
H	6.79023524086249	2.08958149012779	-0.11826170010253
C	5.45126557776767	2.89683670741451	-1.59620471136699
H	3.89647498534713	3.50795458927120	-2.95374349190068
H	6.19702212605425	3.55433792036978	-2.02792289025628
H	0.12139856655614	0.26655657619504	-1.41721100100449
O	0.05046325732432	3.62331073287230	-0.76148531010723

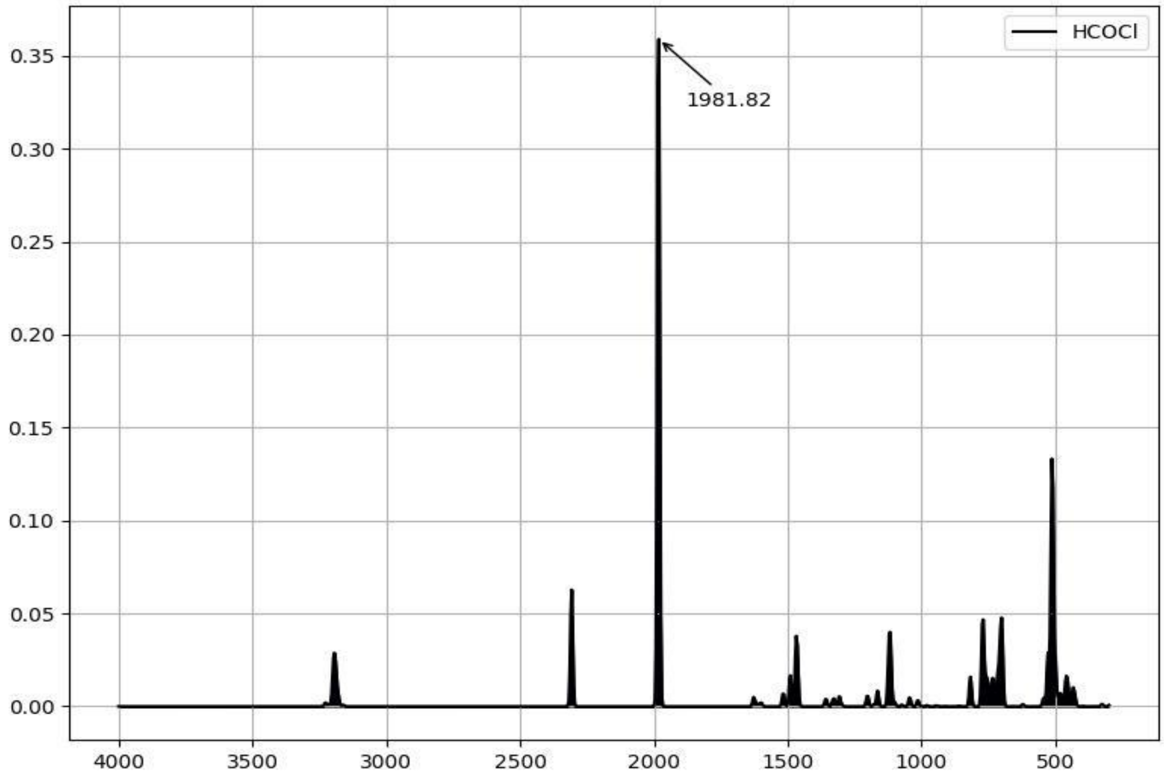
HCOCl
1981.82
0.35
0.30
0.25
0.20
0.15
0.10
0.05
0.00
4000
3500
3000
2500
2000
1500
1000
500

6Hb

Cl	-0.09899878834858	1.25058637986882	2.12514525140452
Ir	0.03687896141275	0.50523576688620	-0.18356965442062
Pd	-0.02625307678157	-2.05610293524315	0.46772253015947
P	-2.24165950469077	0.15315316071983	-0.05262454562876
P	2.28637969868060	0.00797072891321	0.08490762778544
H	0.08657349050637	2.10352596223145	-0.52386089769608
Cl	-0.09511139245462	-4.50529007103316	1.01360991750037
N	-1.51775823813540	-1.64183322785135	1.79015830080279
N	1.47962279065393	-2.34283628081393	-0.87886464880655
C	-2.49690122529688	-0.74875372323057	1.51425344877174
C	-3.39778191106189	1.54781569325232	0.02113237653416
C	-2.91635540605892	-0.92713443185053	-1.34336474827123
C	-1.54900028608777	-2.28781823095915	2.96611900862611
C	-2.55034345544563	-2.07517082880474	3.90126155243841
H	-0.74954763719746	-2.99462981551603	3.13513173701716
C	-3.56875186213991	-1.17992398146391	3.60983698750449
H	-2.52206274282178	-2.61379366212692	4.83944496257475
C	-3.53825307853284	-0.50622517225182	2.39439049769864
H	-4.36814368127086	-0.99747451015299	4.31741965917545
H	-4.30112036058733	0.21570546609197	2.13815785621717
C	-4.76537560783218	1.37177845895958	-0.21005549697134
C	-2.90060800642810	2.81285117786196	0.33477426407473
C	-5.62749416970656	2.45754568449338	-0.11916207477635
H	-6.68699606420886	2.32049885215197	-0.30109701177899
C	-5.12937308624804	3.71915150540998	0.20026583498063
H	-5.15401701419478	0.39271231984771	-0.46408471330502
C	-3.11316169812632	-2.29219104398831	-1.13428264262974
C	-3.16075363046532	-0.37240617459679	-2.60182397216230
C	-3.55111007607096	-3.09684675749694	-2.18055621095190
H	-2.92453719268721	-2.73191434528019	-0.16342229505299
C	-3.79108170183369	-2.54399160628752	-3.43550601065175
H	-3.70470731207426	-4.15637953914987	-2.01276033317326
C	-3.59819754505958	-1.18055595571936	-3.64405699745740
H	-4.13052392993573	-3.17386087255536	-4.24957223822365
H	-3.78640895130164	-0.74571939257895	-4.61865038505079
H	-3.00854303054868	0.68798952320148	-2.76802237248434
C	2.52970307161240	-1.48780994585655	-0.93664895909503
C	2.82907046301958	-0.46252256927802	1.74930744904026
C	3.56995138075614	1.15826657037132	-0.48638371801004
C	1.48167117516218	-3.42468121691424	-1.67338950660930
C	2.52316331735037	-3.69519573484122	-2.54762305146846
H	0.62566404855550	-4.07731280059614	-1.58213354233497
C	3.60966685242264	-2.83416567087411	-2.59383572031152
H	2.47126404795028	-4.57412524734755	-3.17677604169947
C	3.61095105108395	-1.71214209537205	-1.77250731253278
H	4.43924418749052	-3.02300672546485	-3.26392977820929
H	4.43133732040785	-1.00829674511239	-1.79250997998292
C	2.86555272271038	-1.79726519282162	2.15329407113050
C	3.16001870660857	0.54791866692097	2.65437122385612
C	3.23370994525638	-2.11864914194509	3.45499804579801
H	2.60780243286662	-2.58866586007827	1.46135257380113
C	3.56298593724174	-1.11063767034673	4.35703078356711
H	3.26456953039008	-3.15774043771476	3.76138071283132
C	3.52880665720312	0.22194069762385	3.95400907286099
H	3.84892415594769	-1.36296636034775	5.37167809376093
H	3.78498492231271	1.00976741092557	4.65273283292144
H	3.13180393202382	1.58539866229745	2.34532908043404
H	5.19640007469813	0.19718017431893	0.55523409872161

C	4.90896471881162	0.99915139129309	-0.11412175561365
H	-1.83702398632475	2.93777744305018	0.50067123066597
C	-3.76731907747071	3.89636851882774	0.42693929831786
H	-5.80395753320348	4.56501118470427	0.26675117244618
C	3.20653803415474	2.19982474984523	-1.33964907048722
H	-3.37869691786366	4.87858800298880	0.66940461564090
C	5.87218808007959	1.87380584550439	-0.60048271620929
H	2.16424976504518	2.32913254636879	-1.60796946044791
C	4.17386635181610	3.07288492834030	-1.82659061562748
H	6.90851149586474	1.74920476812059	-0.30891230889849
C	5.50573187629207	2.91004470873515	-1.45745106393291
H	3.88624839865095	3.88223296269409	-2.48741496436600
H	6.25936094514420	3.59324229677683	-1.83188160362332
C	0.09992023777961	0.08405295987921	-1.96106913536064
O	0.14796840053366	-0.16923919561366	-3.08736861474667

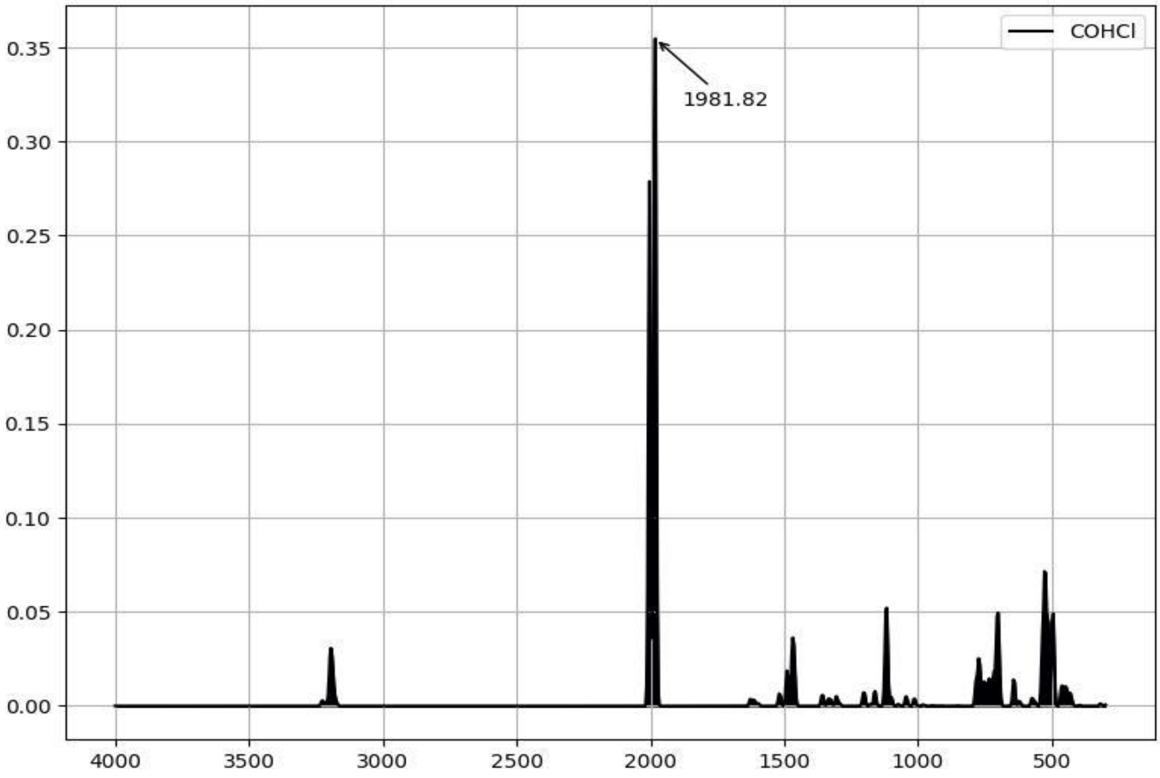

6Hc

C	-0.00323348783562	0.04715455949103	2.40001863644801
Ir	0.05847233844593	-0.20037574613387	0.50831182076436
Pd	-0.15959690598893	-2.79797808924335	0.80766953242912
P	-2.20119117836007	-0.57941045482035	0.28960626535425
P	2.27714136370165	-0.74539658593198	0.39016929214577
Cl	0.16164904249319	2.23464664374827	-0.02045683815692
Cl	-0.35090371831671	-5.21281321536105	1.17730621256953
N	-1.68540789535266	-2.44076890603855	2.12100227187574
N	1.30466612554300	-3.05243568982350	-0.59528793142225
C	-2.62422556352959	-1.51804102162506	1.80123032636949
C	-3.41132862124465	0.76902772562053	0.26094884270588
C	-2.68906777369861	-1.71493733288597	-1.04306762706225
C	-1.80928752833601	-3.12583436140257	3.26616695911094
C	-2.87169264985037	-2.91891528837332	4.13437000521254
H	-1.03696457452568	-3.85332189450685	3.46931785918235
C	-3.84535009443919	-1.98847690384818	3.80338940678028
H	-2.92300776315196	-3.48853319961941	5.05291183381681

C	-3.71770666011500	-1.27721698692772	2.61435360900203
H	-4.68849521336046	-1.81153632719022	4.45974824353281
H	-4.44833919515618	-0.53511972638984	2.32288927108306
C	-4.55515043341029	0.73063664614703	-0.53831417333879
C	-3.17115616508614	1.87467179764716	1.08176607927190
C	-5.45876400196190	1.78800304241551	-0.50512961155527
H	-6.34564387202179	1.75647045933999	-1.12725911941791
C	-5.22497981818645	2.88087135977775	0.32479818842655
H	-4.74156734793606	-0.11972658784236	-1.18250367179800
C	-3.61101000982769	-2.73741890885895	-0.80872490340900
C	-2.13675481023819	-1.56079095097362	-2.31579441154664
C	-3.95925880088324	-3.60942480283986	-1.83447252512463
H	-4.04949420968564	-2.86530262770734	0.17333533447222
C	-3.40193790021879	-3.45666935726706	-3.10033135662109
H	-4.66455093337116	-4.40932421292332	-1.64161105020407
C	-2.49591733028351	-2.42607404493517	-3.34096056869575
H	-3.67077646234556	-4.14011852885473	-3.89743941452458
H	-2.05983750188229	-2.30282846593991	-4.32547072211745
H	-1.41018105515556	-0.77774881954058	-2.49367764402542
C	2.36542647752287	-2.21724731580094	-0.69009460989247
C	3.02685687643053	-1.27944636713735	1.95188377447849
C	3.48122803187307	0.37494294209588	-0.37322691001804
C	1.21961979141136	-4.08924472621038	-1.44462628090895
C	2.18977505565164	-4.34220895592107	-2.40236937242450
H	0.35282693413098	-4.72298811651605	-1.32853090645485
C	3.29795099147989	-3.51238162114096	-2.47701141807129
H	2.06643251436492	-5.18547313331246	-3.06917315729460
C	3.38219168990549	-2.43202702282050	-1.60660038550749
H	4.07888865497850	-3.69226795601777	-3.20554775024512
H	4.21896524096890	-1.74840417602672	-1.64765466244205
C	3.34590576309718	-2.61350648589846	2.20249047688379
C	3.21520985023447	-0.31937849455191	2.94994608478771
C	3.84952986441293	-2.98443066429362	3.44558963990908
H	3.20006302415986	-3.36644113933903	1.43816990848011
C	4.04199344180889	-2.02646145333251	4.43615076688599
H	4.09187142413865	-4.02319421443453	3.63717707874658
C	3.72791875447535	-0.69211775853238	4.18535813545894
H	4.43541839150377	-2.31777352521833	5.40311737993864
H	3.87673660898116	0.05697988056973	4.95419813161473
H	2.96101451052728	0.71772335282681	2.76071338346648
H	5.11727065615600	-0.06635736365032	0.95851898468388
C	4.79227225244294	0.49418668773760	0.09105507306375
H	-2.26876328436368	1.91427564395230	1.67940837335544
C	-4.08139245704270	2.92288008181374	1.11981385465872
H	-5.93114687573595	3.70290088655130	0.34853009297672
C	3.07076636133432	1.09811605844889	-1.49669350147405
H	-3.89442517310144	3.77673177310761	1.76053987704472
C	5.68672785321142	1.33223082074687	-0.56683672329367
H	2.04780162804918	1.01271114780311	-1.84161711310990
C	3.97051629732306	1.92571542749347	-2.15621084499368
H	6.70360344355025	1.42375791576699	-0.20324698627649
C	5.27806407914999	2.04589836414078	-1.68999715287065
H	3.65017205559198	2.48319467307982	-3.02869800519245
H	5.97738735932850	2.69746222832532	-2.20126801649261
H	0.13385132395708	-0.46631505316198	-1.10588983857890
O	-0.04851880633644	0.17338446247462	3.54380419757430

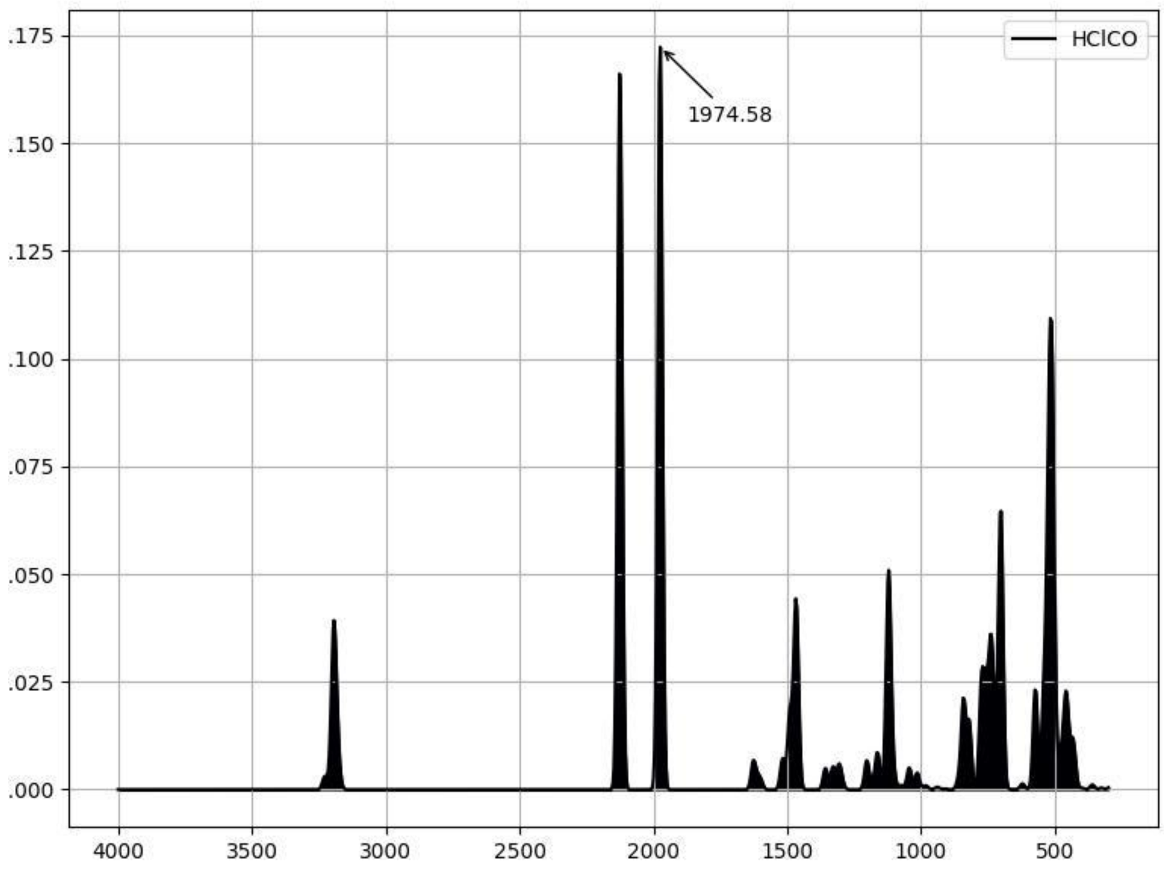

3. Characterization data of organic products

3.1 Analytical data of organic products

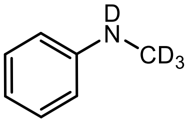

***N*-methylaniline-d$_4$.** obtained in 99% yield. ^{1}H NMR (300 MHz, $CDCl_3$) δ = 7.31 – 7.24 (m, 2H), 6.82 – 6.75 (m, 1H), 6.00 – 6.65 (m, 2H). ^{13}C NMR (75 MHz, $CDCl_3$) δ = 149.4, 129.3, 117.3, 112.5, 30.2 ppm; GC-MS: m/z = 111.20 [M]$^+$.

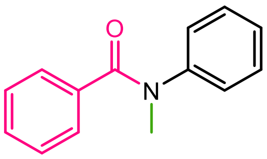

***N*-methyl-*N*-phenyl-benzamide** (**11a**) obtained in 90% yield as a yellow oil. ^{1}H NMR (300 MHz, $CDCl_3$) δ = 7.33 – 7.27 (m, 2H), 7.26 – 7.19 (m, 3H), 7.19 – 7.11 (m, 3H), 7.07 – 7.00 (m, 2H), 3.50 (s, 3H) ppm.

^{13}C NMR (75 MHz, $CDCl_3$) δ = 170.7, 144.9, 136.0, 129.6, 129.2, 128.7, 127.8, 126.9, 126.5, 38.4 ppm; GC-MS: m/z = 211.30 $[M]^+$.

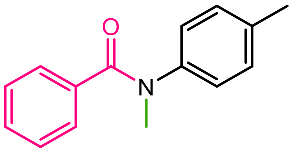

***N*-Methyl-*N*-(4-methylphenyl)benzamide** (**11b**) obtained in 92% yield as an orange oil. ^{1}H NMR (300 MHz, $CDCl_3$) δ = 7.34 – 7.26 (m, 2H), 7.26 – 7.19 (m, 1H), 7.19 – 7.11 (m, 2H), 7.01 (d, J = 8.2, 2H), 6.91 (d, J = 8.2, 2H), 3.46 (s, 3H), 2.26 (s, 3H) ppm. ^{13}C NMR (75 MHz, $CDCl_3$) δ = 170.7, 142.3, 136.3, 136.1, 129.8, 129.5, 128.7, 127.7, 126.7, 38.5, 21.0 ppm; GC-MS: m/z = 225.40 $[M]^+$.

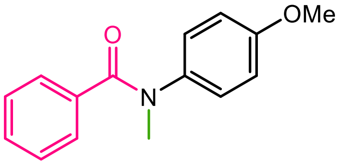

***N*-(4-methoxyphenyl)-*N*-methylbenzamide** (**11c**) obtained in 95% yield as a red oil. ^{1}H NMR (300 MHz, $CDCl_3$) δ = 7.28 (d, J = 7.1, 2H), 7.24 – 7.09 (m, 3H), 6.95 (d, J = 8.7, 2H), 6.72 (d, J = 8.7, 2H), 3.72 (s, 3H), 3.44 (s, 3H) ppm. ^{13}C NMR (75 MHz, $CDCl_3$) δ = 170.7, 157.9, 137.8, 136.1, 129.4, 128.6, 128.1, 127.7, 114.3, 55.4, 38.6 ppm; GC-MS: m/z = 241.20 $[M]^+$.

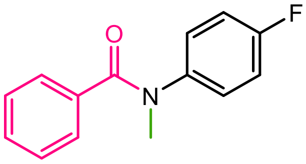

***N*-(4-fluorophenyl)-*N*-methylbenzamide** (**11d**) obtained in 99% yield as an orange oil. ^{1}H NMR (300 MHz, $CDCl_3$) δ = 7.30 – 7.23 (m, 3H), 7.22 – 7.12 (m, 2H), 7.05 – 6.95 (m, 2H), 6.89 (t, J = 8.5, 2H), 3.45 (s, 3H) ppm. ^{13}C NMR (75 MHz, $CDCl_3$) δ = 170.7, 160.8 (d, J = 247.1), 140.9 (d, J = 3.2), 135.8, 129.7, 128.6, 128.6 (d, J = 6.6), 127.9, 116.1 (d, J = 22.7), 38.5 ppm. ^{19}F NMR (282 MHz, $CDCl_3$) δ = -114.9 ppm; GC-MS: m/z = 229.30 $[M]^+$.

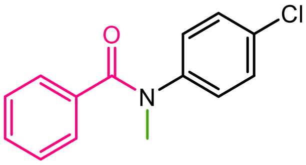

***N*-(4-chlorophenyl)-*N*-methylbenzamide** (**11e**) obtained in 99% yield as an orange oil. ^{1}H NMR (300 MHz, $CDCl_3$) δ = 7.34 – 7.27 (m, 3H), 7.26– 7.17 (m, 4H), 6.99 (d, J = 8.6, 2H), 3.49 (s, 3H) ppm. ^{13}C NMR (75 MHz, $CDCl_3$) δ = 169.6, 142.5, 134.5, 131.0, 128.8, 128.3, 127.6, 127.0, 126.9, 37.3 ppm; GC-MS: m/z = 245.20 $[M]^+$.

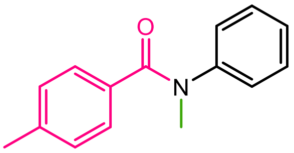

***N*,4-Dimethyl-*N*-phenylbenzamide** (**11f**). obtained in 96% yield as a yellow oil. ^{1}H NMR (300 MHz, $CDCl_3$) δ = 7.26 – 7.16 (m, 4H), 7.13 (t, J = 7.3 Hz, 1H), 7.02 (d, J = 7.9 Hz, 2H), 6.94 (d, J = 7.9 Hz, 2H), 3.47 (s, 3H), 2.23 (s, 3H) ppm. ^{13}C NMR (75 MHz, $CDCl_3$) δ = 170.8, 145.3, 139.9, 133.1, 129.3, 129.0, 128.5, 127.0, 126.5, 38.6, 21.5 ppm; GC-MS: m/z = 225.30 $[M]^+$.

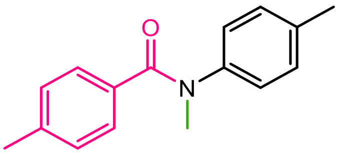

***N*,4-Dimethyl-*N*-(4-methylphenyl)benzamide** (**11g**). obtained in 99% yield as a brown oil. ^{1}H NMR (300 MHz, $CDCl_3$) δ = 7.22 – 7.15 (m, 2H), 7.00 (d, J = 8.2, 2H), 6.92 (dd, J = 11.6, 8.2, 4H), 3.44 (s, 3H), 2.25 (s, 3H), 2.23 (s, 3H) ppm. ^{13}C NMR (75 MHz, $CDCl_3$) δ = 170.8, 142.8, 139.8, 136.3, 133.2, 129.9, 129.0, 128.5, 126.8, 38.7, 21.5, 21.1 ppm; GC-MS: m/z = 239.40 $[M]^+$.

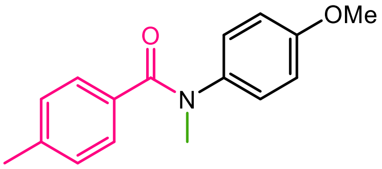

***N*-(4-methoxyphenyl)-*N*,4-dimethylbenzamide (11h)**. obtained in 90% yield as a yellow oil. ^{1}H NMR (300 MHz, $CDCl_3$) δ = 7.18 (d, J = 8.0, 2H), 6.94 (d, J = 8.0, 4H), 6.73 (d, J = 8.8, 2H), 3.74 (s, 3H), 3.44 (s, 3H), 2.25 (s, 3H) ppm. ^{13}C NMR (75 MHz, $CDCl_3$) δ = 170.9, 158.0, 139.7, 138.2, 133.3, 129.0, 128.5, 128.2, 114.5, 55.5, 38.9, 21.5 ppm; GC-MS: m/z = 255.30 $[M]^+$.

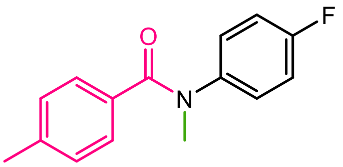

***N*-(4-fluorophenyl)-*N*,4-dimethylbenzamide (11i)**. obtained in 61% yield as a yellow oil. ^{1}H NMR (300 MHz, $CDCl_3$) δ = 7.17 (d, J = 8.1, 2H), 7.06 – 6.94 (m, 4H), 6.90 (t, J = 8.8, 2H), 3.44 (s, 3H), 2.25 (s, 3H) ppm. ^{13}C NMR (75 MHz, $CDCl_3$) δ = 170.7, 160.7 (d, J = 246.8), 141.3 (d, J = 3.2 Hz), 139.9, 132.8, 128.8, 128.6 (d, J = 8.4 Hz), 128.5, 116.1 (d, J = 22.6 Hz), 38.6, 21.3 ppm. ^{19}F NMR (282 MHz, CDCl3) δ = -115.1 ppm; GC-MS: m/z = 243.30 $[M]^+$.

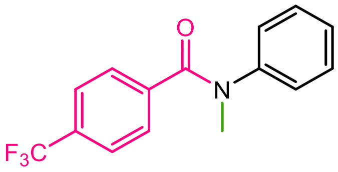

***N*-Methyl-*N*-phenyl-4-(trifluoromethyl)benzamide (11j).** obtained in 99% yield as a yellow oil. ^{1}H NMR (300 MHz, $CDCl_3$) *δ* = 7.46 – 7.36 (m, 4H), 7.25 – 7.21 (m, 2H), 7.20 – 7.14 (m, 1H), 7.03 (d, *J* = 7.5, 2H), 3.51 (s, 3H) ppm. ^{13}C NMR (75 MHz, $CDCl_3$) *δ* = 169.2, 144.2, 139.4, 131.3 (q, *J* = 32.9) 129.4, 129.0, 127.1, 126.6, 124.8 (q, *J* = 8.9), 123.7 (q, *J* = 272.3), 38.4 ppm. ^{19}F NMR (282 MHz, $CDCl_3$) *δ* = -63.0 ppm; GC-MS: m/z = 279.15 $[M]^+$.

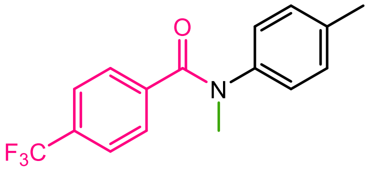

***N*-methyl-*N*-(p-tolyl)-4-(trifluoromethyl)benzamide (11k).** in obtained in 91% yield as an orange oil. ^{1}H NMR (300 MHz, $CDCl_3$) *δ* = 7.46 – 7.39 (m, 4H), 7.04 (d, *J* = 8.1, 2H), 6.92 (d, *J* = 8.1, 2H), 3.49 (s, 3H), 2.28 (s, 3H) ppm. ^{13}C NMR (75 MHz, $CDCl_3$) *δ* = 169.2, 141.6, 136.9, 130.7 (q, *J* = 31.9), 130.0, 129.0, 126.7, 124.8 (q, *J* = 8.8 Hz), 123.7 (q, *J* = 272.1), 118.3, 38.5, 21.0 ppm. ^{19}F NMR (282 MHz, $CDCl_3$) *δ* = -63.0 ppm; GC-MS: m/z = 293.15 $[M]^+$.

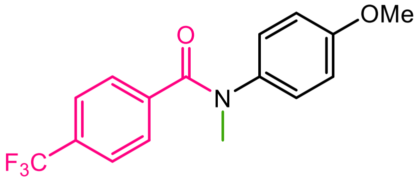

***N*-(4-methoxyphenyl)-*N*-methyl-4-(trifluoromethyl)benzamide (11l).** obtained in 97% yield as a brown oil. ^{1}H NMR (300 MHz, $CDCl_3$) *δ* = 7.55 – 7.40 (m, 4H), 7.02 (d, *J* = 8.4, 2H), 6.85 – 6.77 (m, 2H), 3.82 (s, 3H), 3.53 (s, 3H) ppm. ^{13}C NMR (75 MHz, $CDCl_3$) *δ* = 169.4, 158.4, 139.8, 137.2, 131.3 (q, *J* = 33.1), 129.1, 128.3, 124.9 (q, *J* = 3.8), 123.9 (q, *J* = 272.4) 114.7, 55.5, 38.7 ppm. ^{19}F NMR (282 MHz, $CDCl_3$) *δ* = -62.9 ppm; IR (ATR): $\tilde{v}$ = 3380 (w), 3067 (w), 3002 (w), 2940 (m), 2838 (w), 1644 (s, CO), 1580 (w), 1513 (s), 1466 (m), 1439 (w), 1407 (w), 1373 (m), 1323 (s), 1248 (s), 1168 (s), 1124 (s), 1108 (s), 1066 (s), 1034 (m), 1016 (m), 854 (m), 834 (m), 769 (w), 727 (w), 690 (w), 615 (w) cm^{-1};GC-MS: m/z = 309.30 $[M]^+$; HR-MS: m/z 310.1054 (calcd, $[M+H]^+$); 310.1148 (found, $[M+H]^+$).

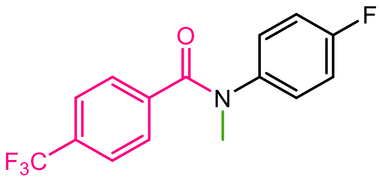

***N*-(4-fluorophenyl)-*N*-methyl-4-(trifluoromethyl)benzamide (11m).** obtained in 97% yield as a brown oil. ^{1}H NMR (300 MHz, $CDCl_3$) *δ* = 7.49 – 7.35 (m, 4H), 7.05 – 6.97 (m, 2H), 6.93 (t, *J* = 8.5, 2H) 3.47

(s, 3H). ^{13}C NMR (75 MHz, $CDCl_3$) δ = 169.4, 161.2 (d, J = 248.2), 139.4, 131.6 (q, J = 32.7), 130.4, 129.1, 128.8 (d, J = 8.6), 125.1 (q, J = 3.9), 123.8 (q, J = 272.8), 116.6 (d, J = 22.8), 38.7 ppm. ^{19}F NMR (282 MHz, $CDCl_3$) δ = -63.0, -113.9 ppm; IR (ATR): $\tilde{\nu}$ = 3342 (w), 3066 (w), 2963 (w), 2935 (m), 2876 (w), 1645 (s, CO), 1608 (w), 1578 (w), 1507 (s), 1429 (w), 1408 (m), 1370 (m), 1321 (s), 1278 (w), 1224 (m), 1164 (s), 1122 (s), 1106 (s), 1064 (s), 1017 (m), 841 (s), 815 (m), 768 (m), 723 (m), 691 (w), 610 (m) cm^{-1}; GC-MS: m/z = 297.20 [M]$^+$; HR-MS: m/z 298.0850 (calcd, [M+H]$^+$); 298.0854 (found, [M+H]$^+$).

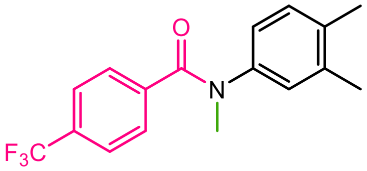

***N*-(3,4-dimethylphenyl)-*N*-methyl-4-(trifluoromethyl)benzamide (11n)** obtained in 93% yield as a yellow oil. ^{1}H NMR (300 MHz, $CDCl_3$) δ = 7.48 – 7.38 (m, 4H), 6.96 (d, J = 7.9, 1H), 6.84 (s, 1H), 6.71 (d, J = 7.9, 1H), 3.46 (s, 3H), 2.18 (s, 3H), 2.16 (s, 3H) ppm. ^{13}C NMR (75 MHz, $CDCl_3$) δ = 169.3, 142.0, 138.1, 135.8, 131.3 (q, J = 32.5), 130.6, 129.1, 128.4, 127.8, 124.9 (q, J = 3.8), 123.9 (q, J = 272.7), 124.5, 38.7, 19.9, 19.4 ppm. ^{19}F NMR (282 MHz, $CDCl_3$) δ = -62.9 ppm; IR (ATR): $\tilde{\nu}$ = 3348 (w), 3055 (w), 2971 (w), 2927 (m), 2868 (w), 1645 (s, CO), 1606 (m), 1578 (m), 1502 (s), 1425 (w), 1407 (m), 1368 (m), 1320 (s), 1274 (w), 1163 (s), 1120 (s), 1105 (s), 1064 (s), 1030 (m), 1016 (s), 850 (s), 821 (m), 768 (s), 717 (s), 691 (s), 615 (m) cm^{-1}; HR-MS: m/z 308.1257 (calcd, [M+H]+); 308.1265 (found, [M+H]$^+$).

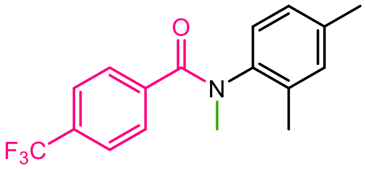

***N*-(2,4-dimethylphenyl)-*N*-methyl-4-(trifluoromethyl)benzamide (11o)** obtained in 88% yield as a brown oil. ^{1}H NMR (300 MHz, $CDCl_3$) δ = 7.42 – 7.37 (m, 4H), 6.94 (s, 1H), 6.91 – 6.89 (m, 2H), 3.36 (s, 3H), 2.25 (s, 3H), 2.15 (s, 3H) ppm. ^{13}C NMR (75 MHz, $CDCl_3$) δ = 169.4, 140.2, 138.0, 134.3, 132.2, 131.2 (q, J = 32.8), 129.2, 128.6, 128.2, 127.9, 124.6 (q, J = 3.7), 123.9 (q, J = 272.6), 37.6, 20.9, 17.6 ppm. ^{19}F NMR (282 MHz, $CDCl_3$) δ = -62.9 ppm; IR (ATR): $\tilde{\nu}$ = 3350 (w), 3058 (w), 2969 (w), 2924 (m), 2865 (w), 1649 (s, CO), 1609 (m), 1582 (m), 1505 (s), 1428 (w), 1406 (m), 1370 (m), 1322 (s), 1273 (w), 1160 (s), 1122 (s), 1107 (s), 1063 (s), 1032 (m), 1017 (s), 848 (s), 820 (m), 769 (s), 717 (s), 691 (s), 616 (m) cm^{-1};GC-MS: m/z = 307.35; HR-MS: m/z 308.1257 (calcd, [M+H]$^+$); 308.1356 (found, [M+H]$^+$).

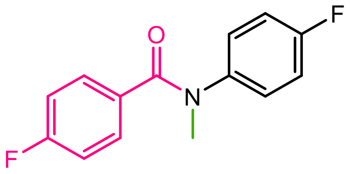

4-Fluoro-*N*-(4-fluorophenyl)-*N*-methylbenzamide (11p). obtained in 70% yield as a reddish oil. ^{1}H NMR (300 MHz, $CDCl_3$) δ = 7.31 – 7.24 (m, 2H), 7.00 (ddd, J = 9.1, 4.8, 2.1, 2H), 6.93 (dd, J = 8.4, 2.1,

2H), 6.88 – 6.80 (m, 2H), 3.44 (s, 3H) ppm. ^{13}C NMR (75 MHz, $CDCl_3$) δ = 169.6, 163.3 (d, J = 250.7 Hz), 160.9 (d, J = 247.4 Hz), 140.9 (d, J = 3.3 Hz), 131.8 (d, J = 3.6 Hz), 131.1 (d, J = 8.6 Hz), 128.6 (d, J = 8.8 Hz), 116.3 (d, J = 22.6 Hz), 115.0 (d, J = 21.7 Hz), 38.7 ppm. ^{19}F NMR (282 MHz, $CDCl_3$) δ = -109.8, -114.5 ppm; GC-MS: m/z = 247.35 $[M]^+$.

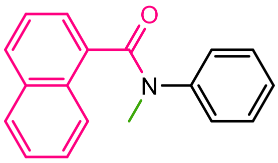

N-(4-fluorophenyl)-N-methyl-1-naphthamide (11q). obtained in 94% yield as an orange oil. Spectral data match those previously reported.[21] ^{1}H NMR (300 MHz, $CDCl_3$) δ = 8.08 (d, J = 8.4, 2H), 7.76 – 7.57 (m, 1H), 7.56 – 7.44 (m, 1H), 7.41 (t, J = 7.6, 1H), 7.23 – 6.84 (m, 7H), 3.56 (s, 3H) ppm. ^{13}C NMR (75 MHz, $CDCl_3$) δ = 170.6, 144.1, 137.9, 134.4, 133.4, 129.1, 128.9, 128.3, 128.3, 126.9, 126.7, 126.3, 126.2, 125.4, 124.5, 37.5 ppm; GC-MS: m/z = 261.10 $[M]^+$.

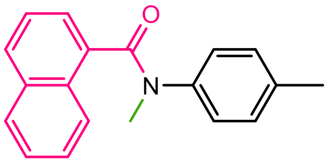

N-methyl-N-(p-tolyl)-1-naphthamide (11r) obtained in 96% yield as an orange oil. Spectral data match those previously reported.[21] ^{1}H NMR (300 MHz, $CDCl_3$) δ = 8.17 (d, J = 8.0 Hz, 1H), 7.71 (d, J = 7.2 Hz, 1H), 7.62 (d, J = 5.9 Hz, 1H), 7.53 (t, J = 7.3 Hz, 1H), 7.42 (t, J = 7.3 Hz, 1H), 7.25 – 7.11 (m, 2H), 6.93 – 6.66 (m, 4H), 3.60 (s, 3H), 2.06 (s, 3H) ppm. ^{13}C NMR (75 MHz, $CDCl_3$) δ = 170.6, 141.5, 136.5, 134.6, 133.3, 130.4, 129.9, 129.6, 128.9, 128.3, 126.8, 126.1, 125.6, 125.5, 124.5, 37.6, 20.9 ppm; GC-MS: m/z = 275.15 $[M]^+$.

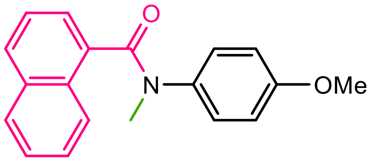

N-(4-methoxyphenyl)-N-methyl-1-naphthamide (11s). obtained in 94% yield as an orange oil. ^{1}H NMR (300 MHz, $CDCl_3$) δ = 8.07 (d, J = 8.0 Hz, 1H), 7.75 (d, J = 8.1 Hz, 1H), 7.66 (d, J = 8.0 Hz, 1H), 7.53 (t, J = 7.3 Hz, 1H), 7.45 (m, 1H), 7.23 – 7.12 (m, 2H), 6.90 (d, J = 8.9 Hz, 2H), 6.53 (d, J = 8.9 Hz, 2H), 3.61 (s, 3H), 3.58 (s, 3H) ppm. ^{13}C NMR (75 MHz, $CDCl_3$) δ = 170.8, 157.9, 137.0, 134.6, 133.3, 130.3, 129.0, 128.2, 127.6, 126.7, 126.0, 125.5, 125.4, 124.4, 114.0, 55.2, 37.7 ppm; IR (ATR): $\tilde{\nu}$ = 3411 (w), 3334 (w), 3051 (m), 3006 (w), 2957 (w), 2935 (m), 2837 (w), 1675 (w), 1643 (s, CO), 1592 (w), 1510 (s), 1466 (m), 1441 (m), 1397 (w), 1368 (s), 1296 (m), 1247 (s), 1174 (m), 1115 (s), 1032 (s), 835 (s), 803 (s), 781 (s) cm^{-1};HR-MS: m/z 292.1332 (calcd, $[M+H]^+$); 292.1339 (found, $[M+H]^+$).

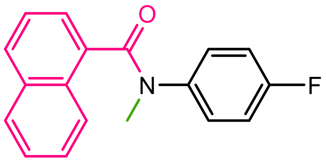

***N*-(4-fluorophenyl)-*N*-methyl-1-naphthamide (11t)** obtained in 96% yield as a yellow oil. Spectral data match those previously reported.[21] ^{1}H NMR (300 MHz, $CDCl_3$) δ = 8.05 (d, *J* = 8.3 Hz, 1H), 7.76 (d, *J* = 8.3, 1H), 7.72 – 7.65 (m, 1H), 7.54 (t, *J* = 7.2 Hz, 1H), 7.50 – 7.44 (m, 1H), 7.24 – 7.10 (m, 2H), 7.01 – 6.86 (m, 2H), 6.82 – 6.64 (m, 2H), 3.59 (s, 3H) ppm. ^{13}C NMR (75 MHz, $CDCl_3$) δ = 170.6, 160.8 (d, *J* = 247.1), 140.1, 134.2, 133.4, 130.2, 129.2, 128.4, 128.1 (d, *J* = 8.4), 126.9, 126.2, 125.6, 125.2, 124.5, 115.8 (d, *J* = 22.8), 37.6 ppm. ^{19}F NMR (282 MHz, $CDCl_3$) δ = -114.6; GC-MS: m/z = 279.10 $[M]^{+}$.

3.2 Copies of ^{1}H, ^{13}C NMR and ^{19}F NMR spectra of the *N*-methylated amines and amides

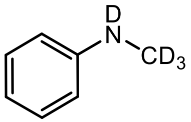

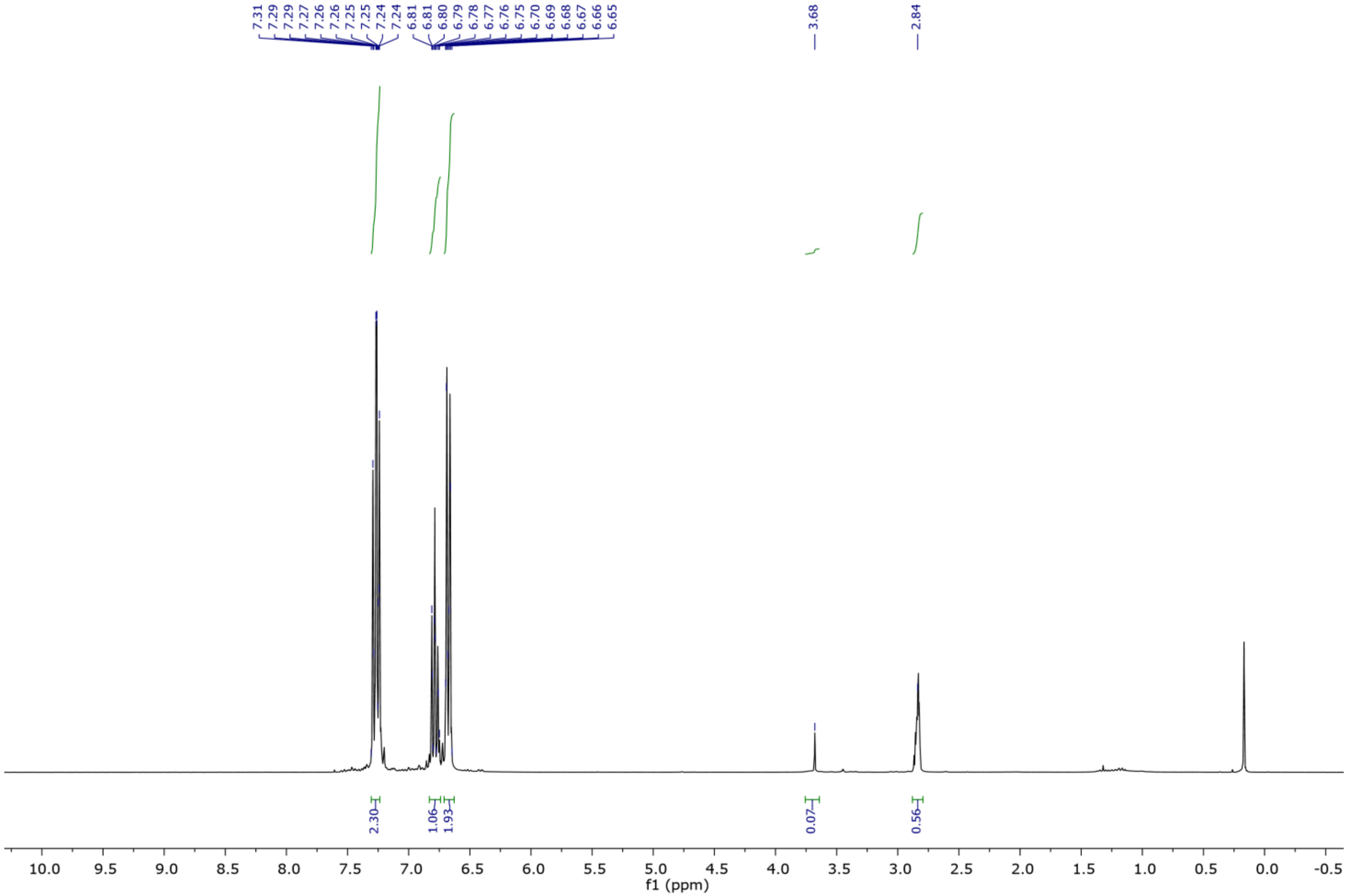

^{1}H NMR spectrum of ***N*-methylaniline-d$_4$** (300 MHz, $CDCl_3$)

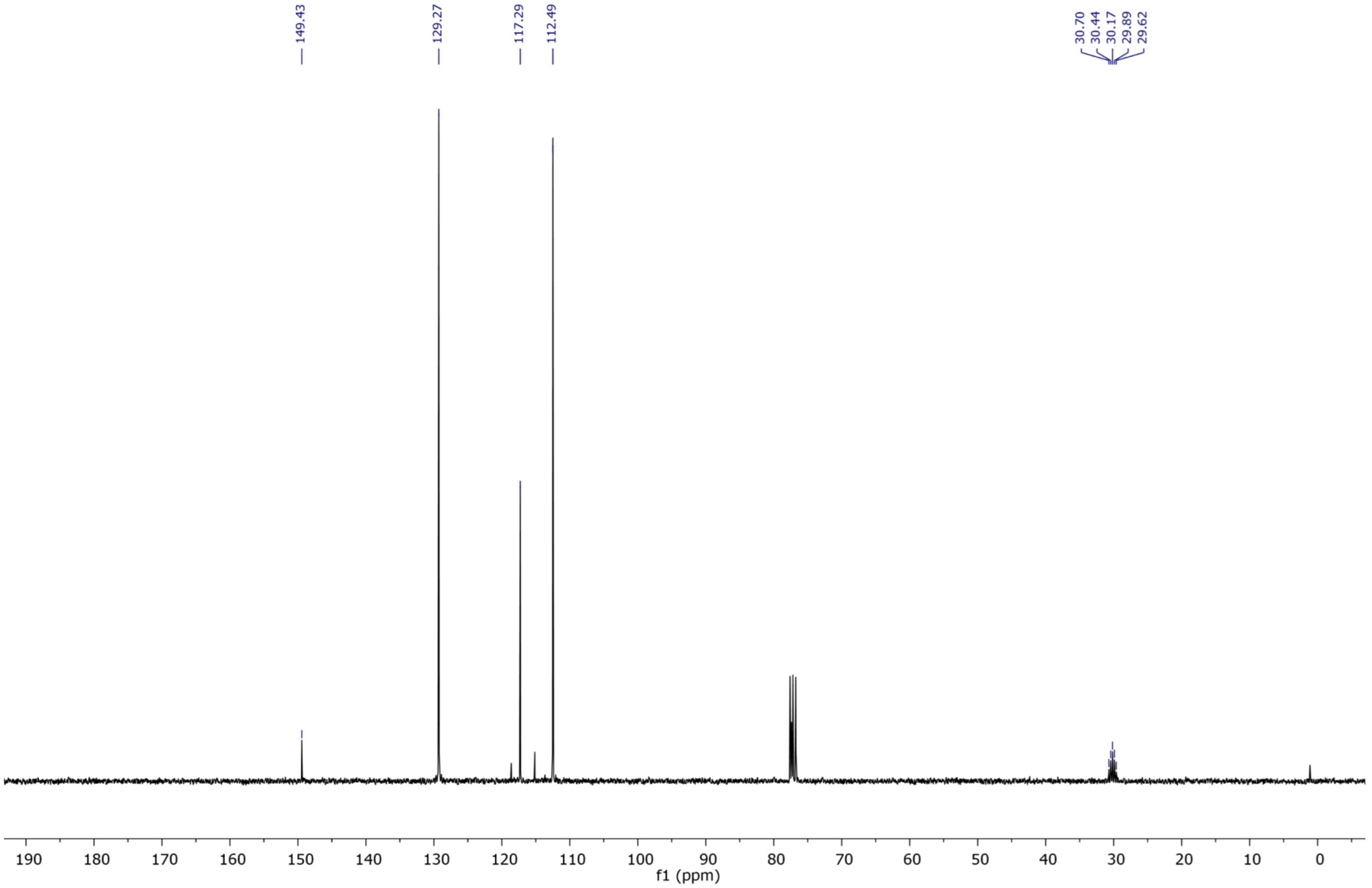

^{13}C NMR spectrum of ***N*-methylaniline-d$_4$** (75 MHz, $CDCl_3$)

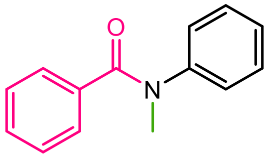

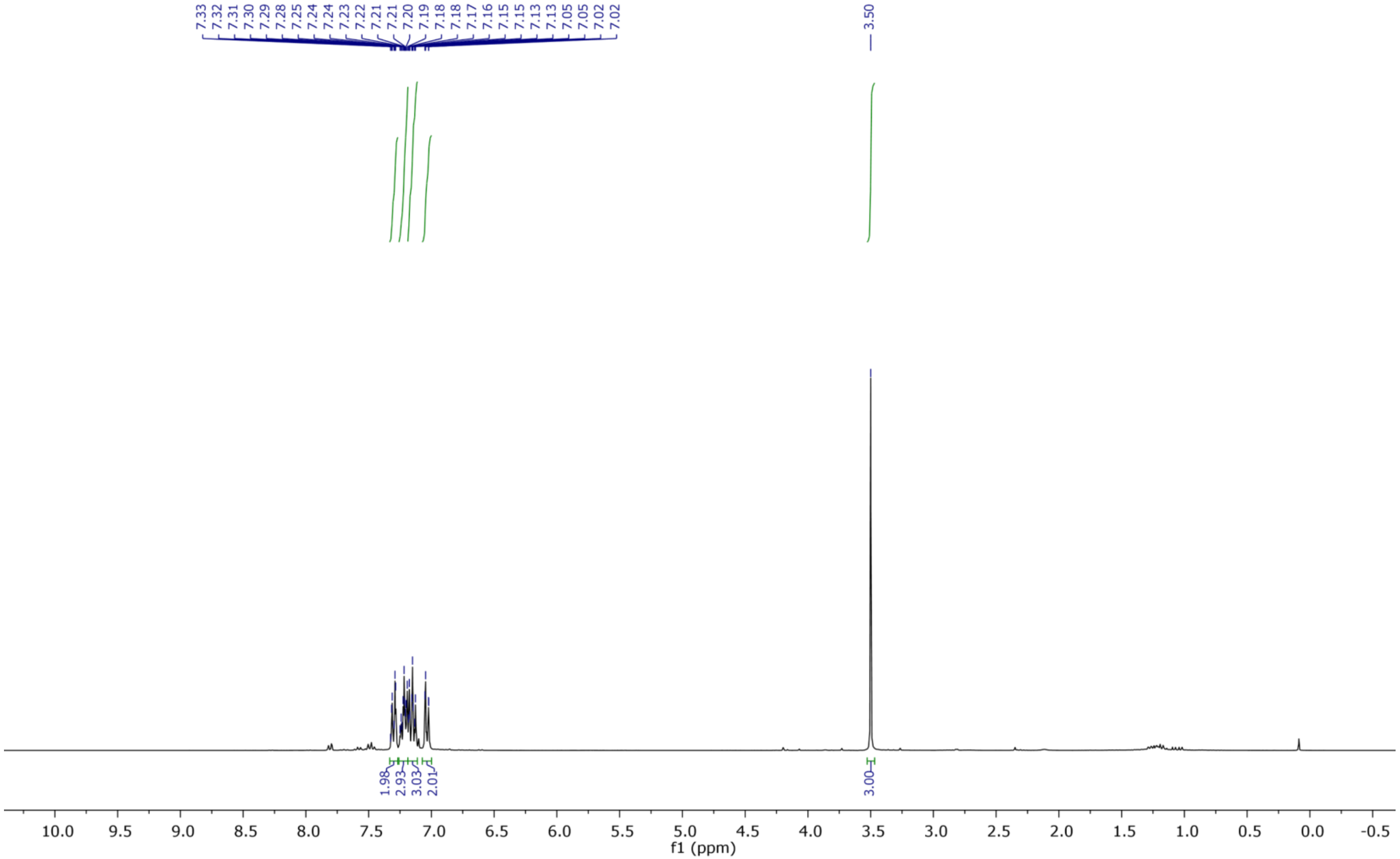

^{1}H NMR spectrum of **11a** (300 MHz, $CDCl_3$)

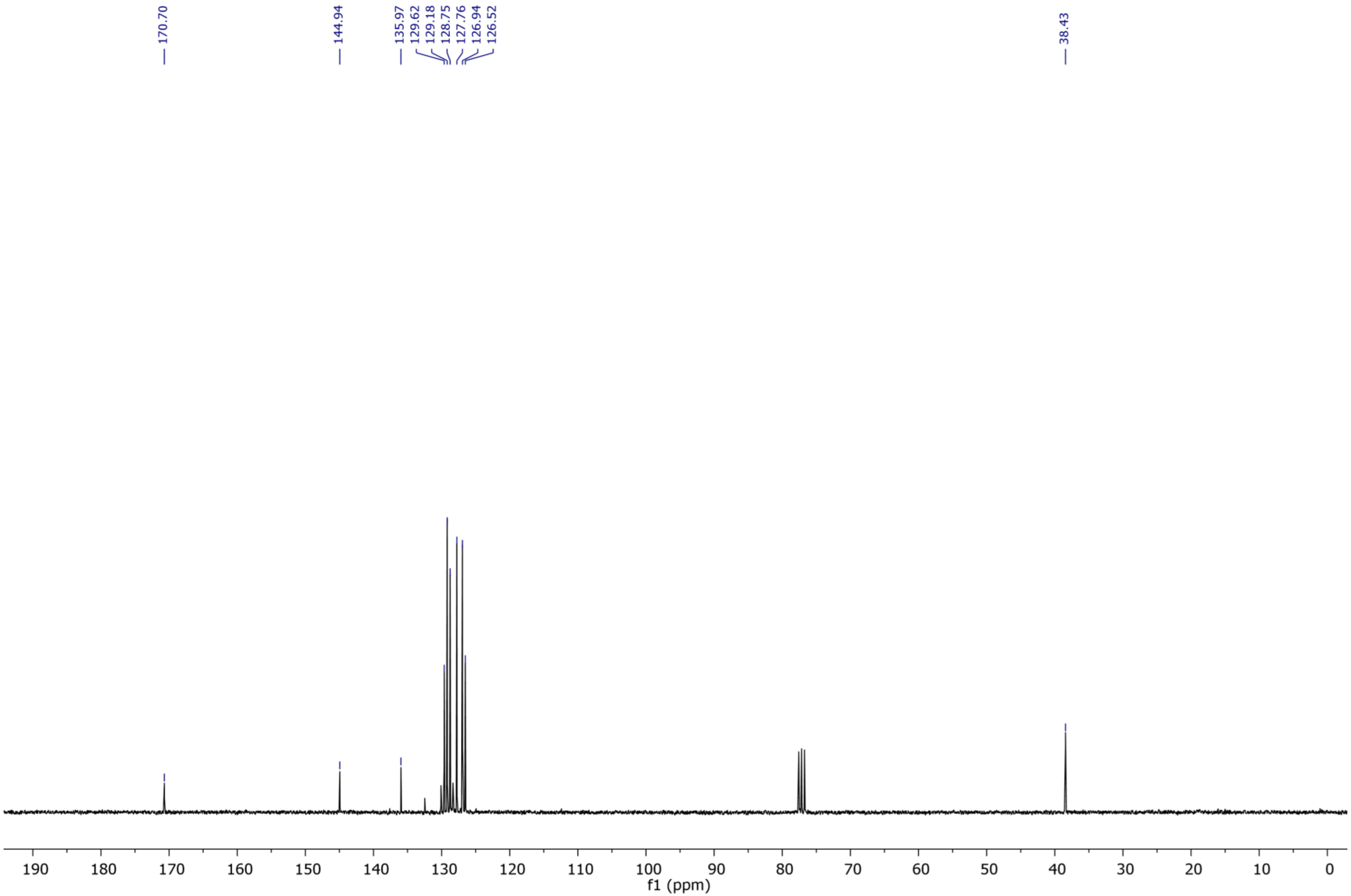

^{13}C NMR spectrum of **11a** (75 MHz, $CDCl_3$)

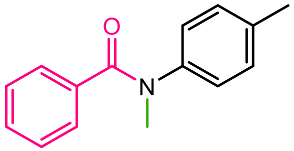

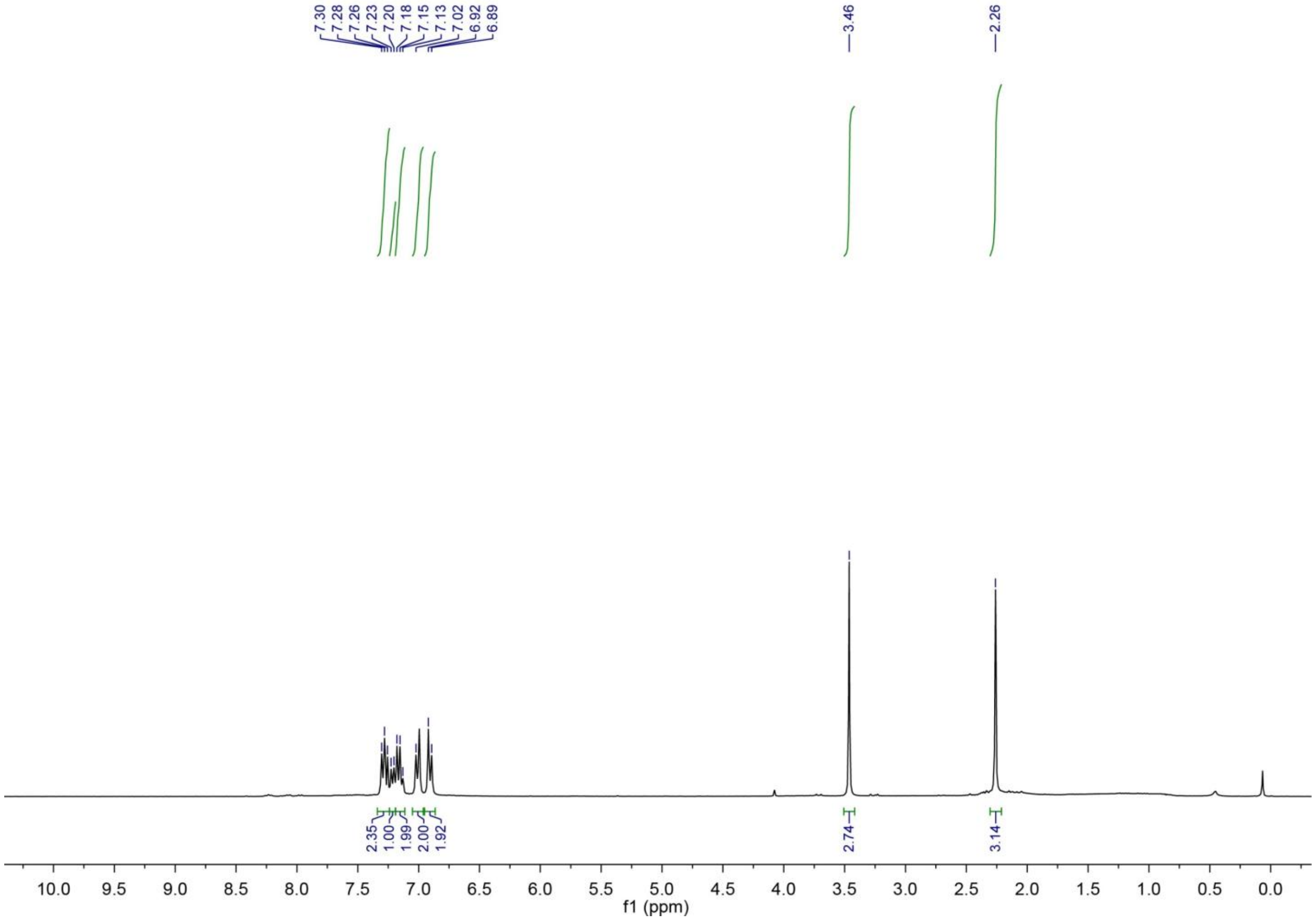

^{1}H NMR spectrum of **11b** (300 MHz, $CDCl_3$)

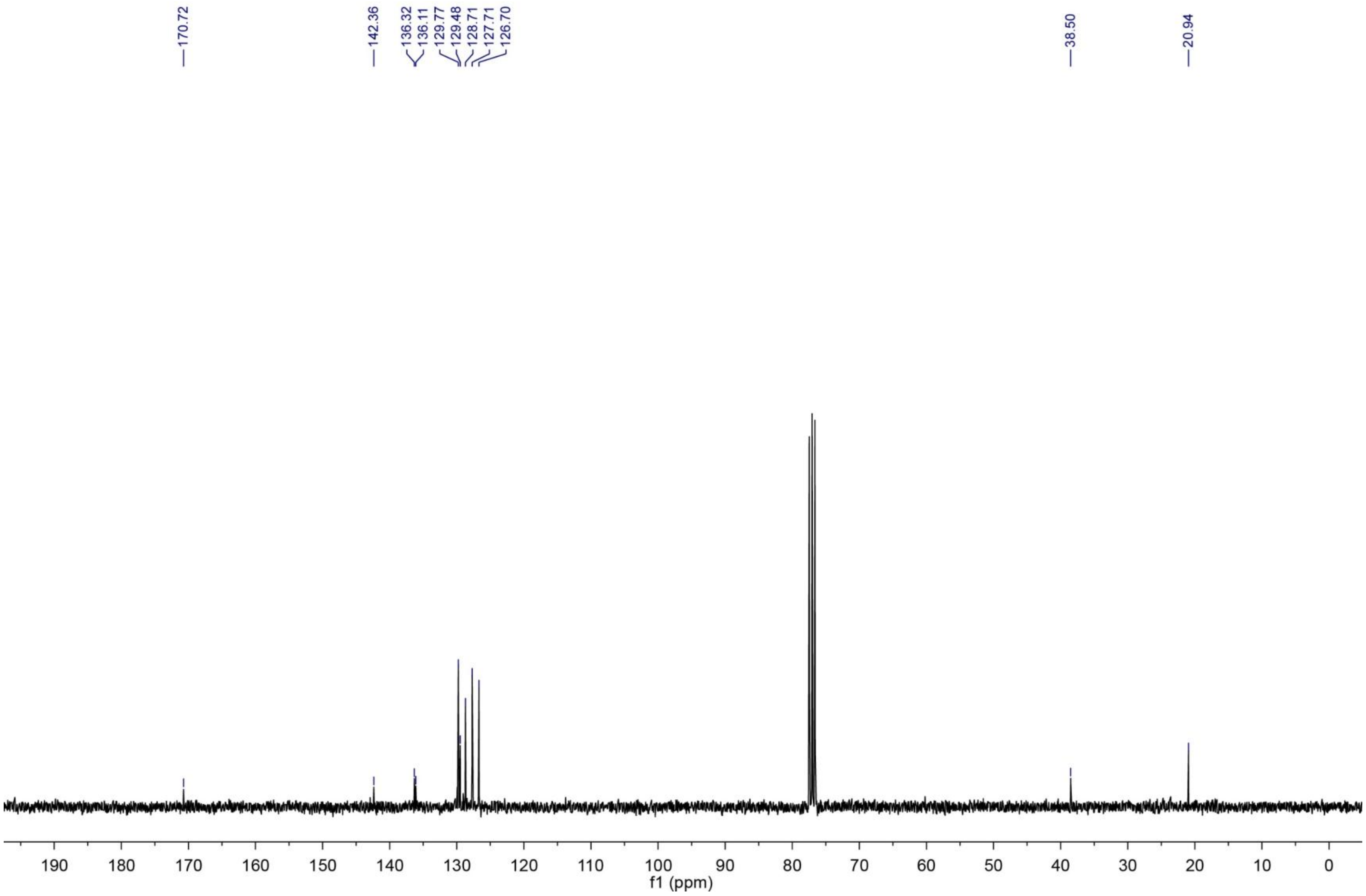

^{13}C NMR spectrum of **11b** (75 MHz, $CDCl_3$)

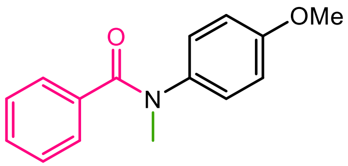

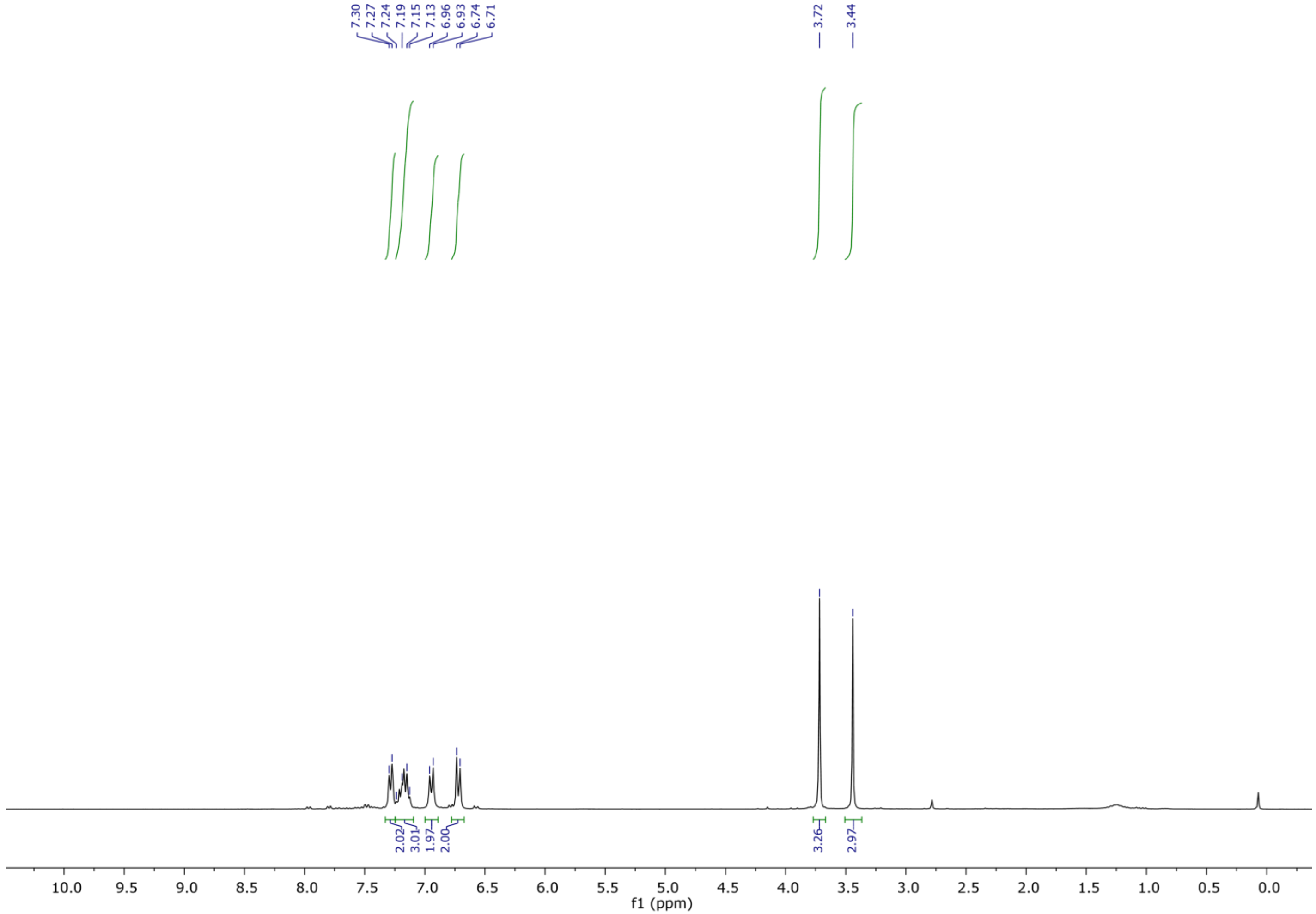

^{1}H NMR spectrum of **11c** (300 MHz, $CDCl_3$)

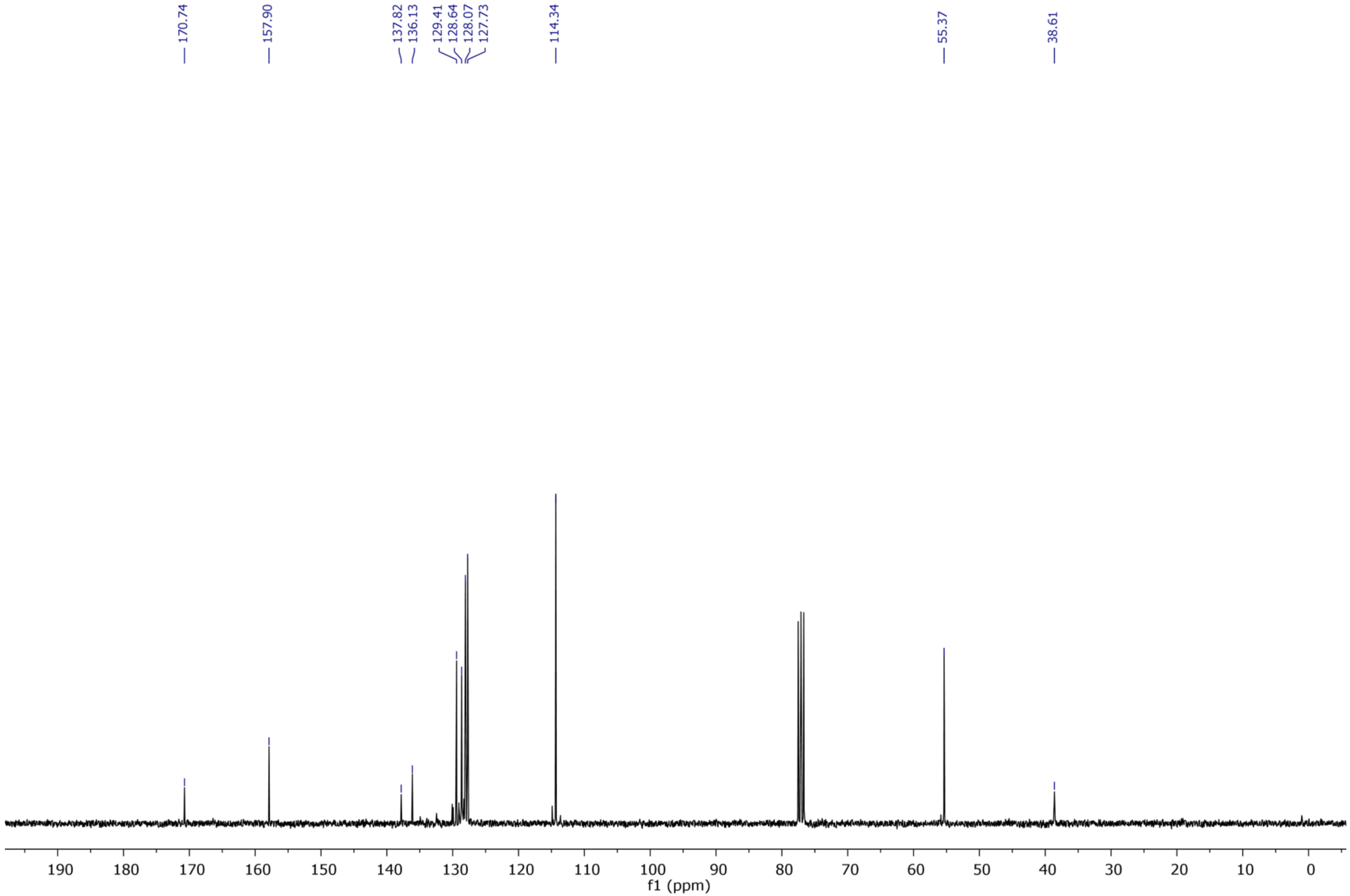

^{13}C NMR spectrum of **11c** (75 MHz, $CDCl_3$)

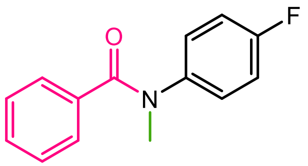

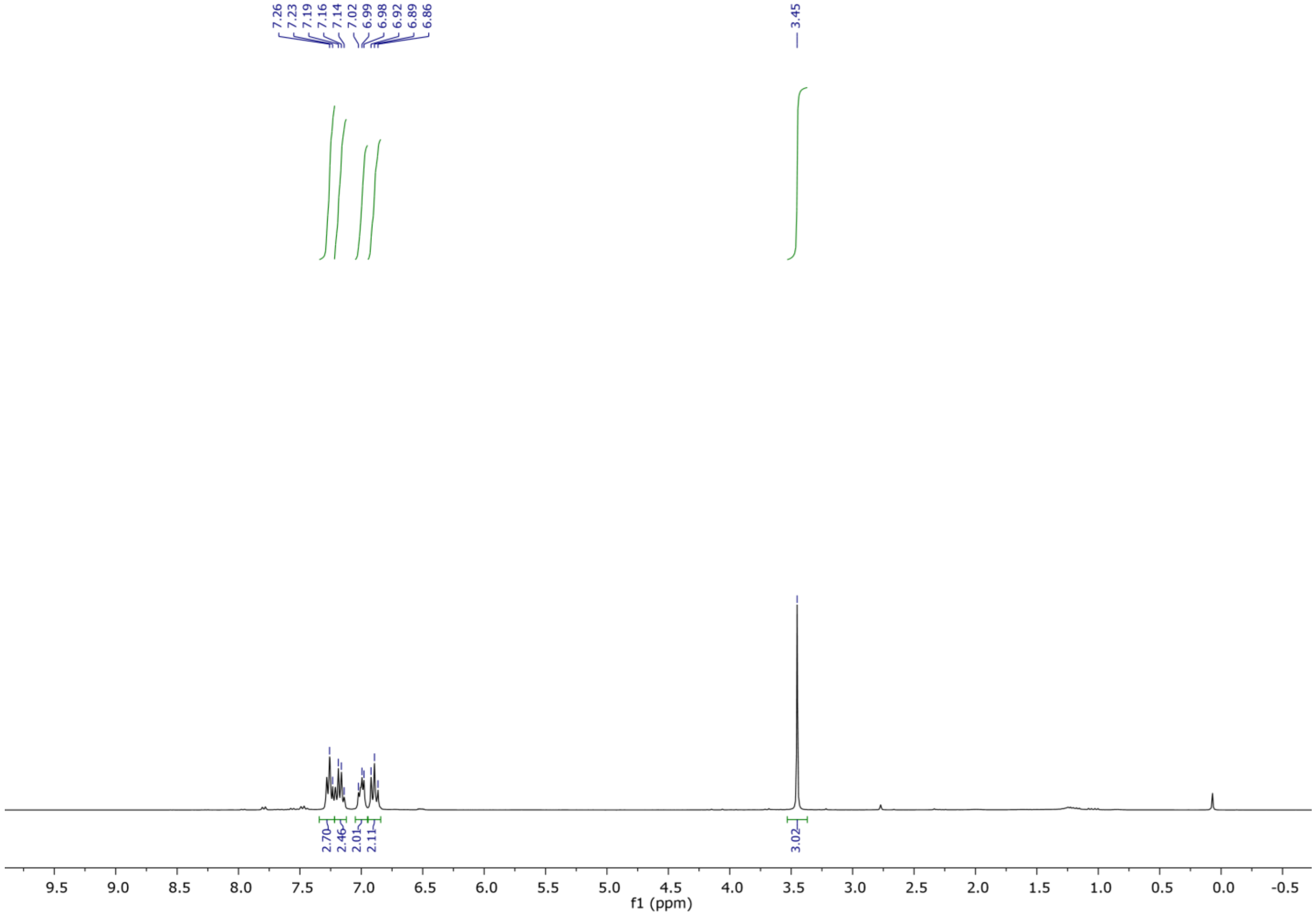

^{1}H NMR spectrum of **11d** (300 MHz, $CDCl_3$)

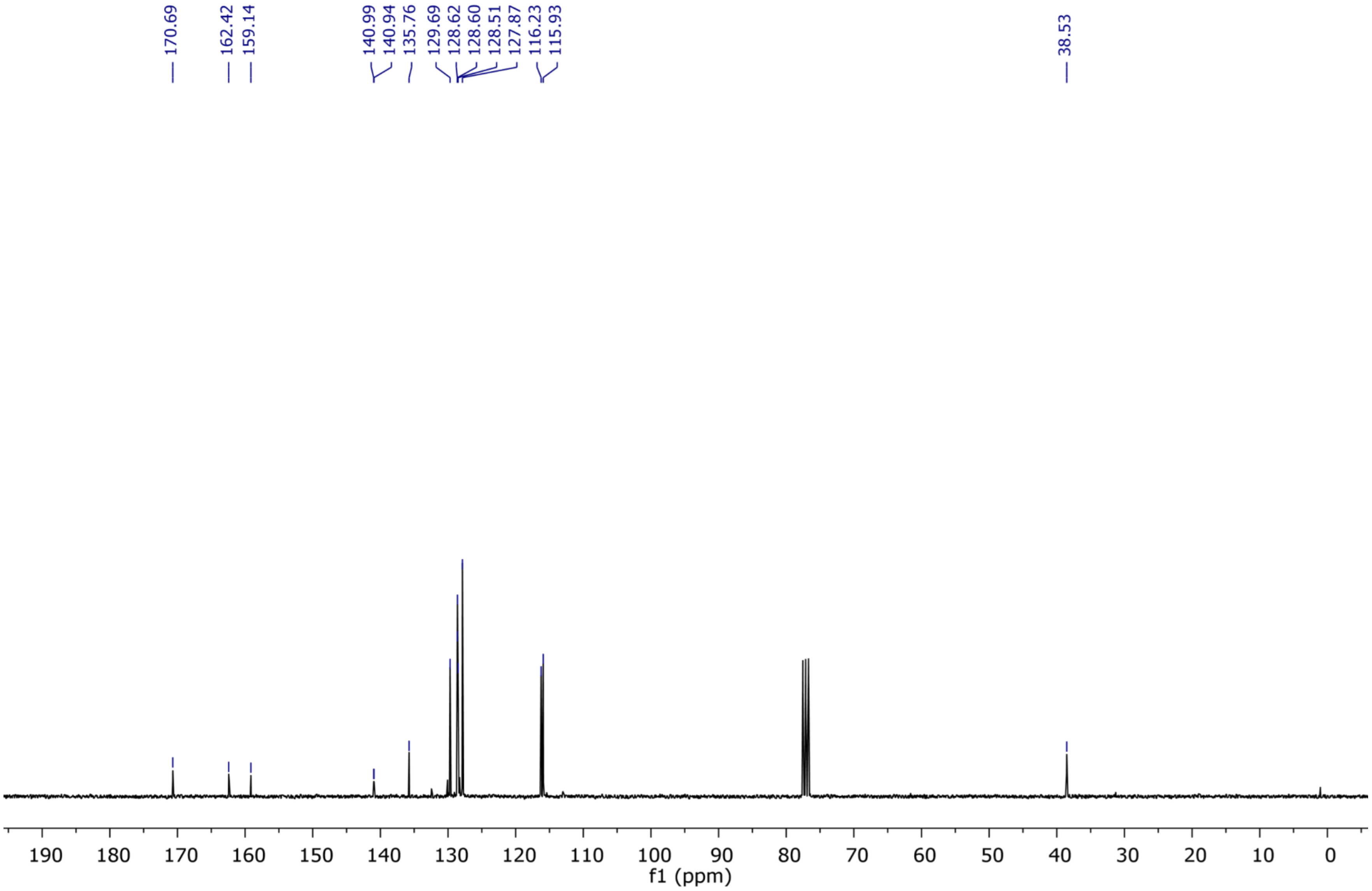

^{13}C NMR spectrum of **11d** (75 MHz, $CDCl_3$)

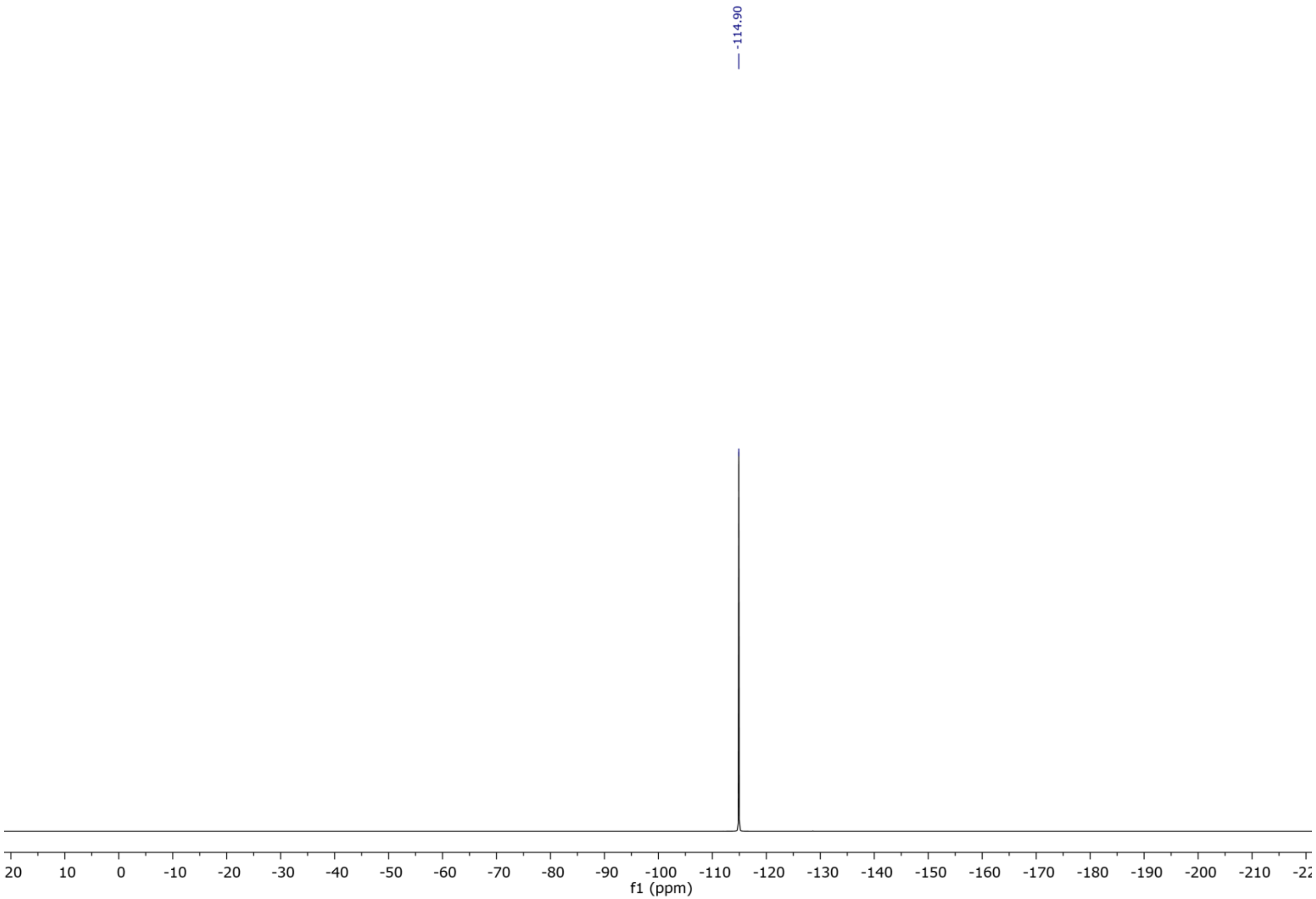

^{19}F NMR spectrum of **11d** (282 MHz, $CDCl_3$)

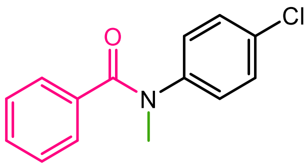

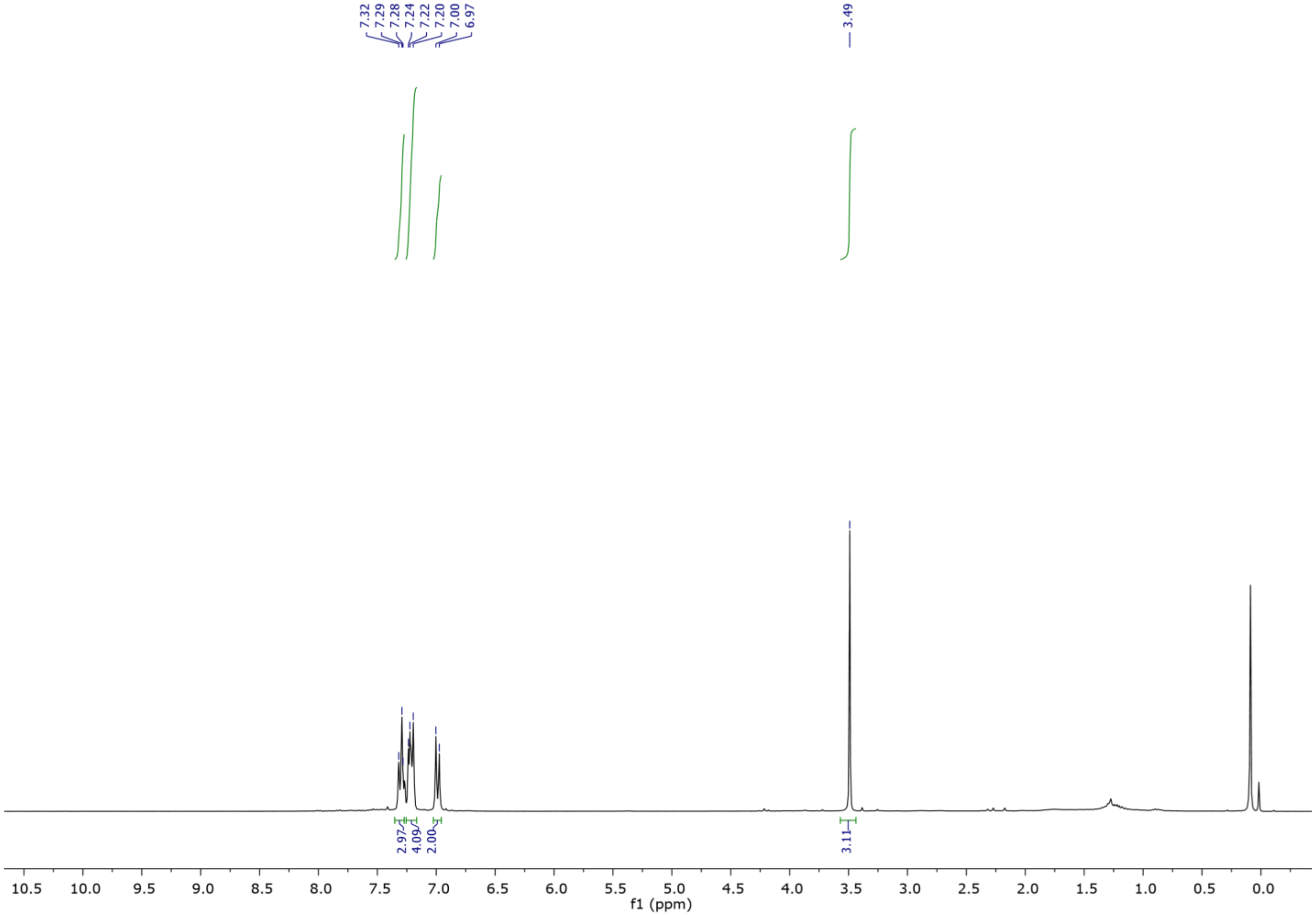

^{1}H NMR spectrum of **11e** (300 MHz, $CDCl_3$)

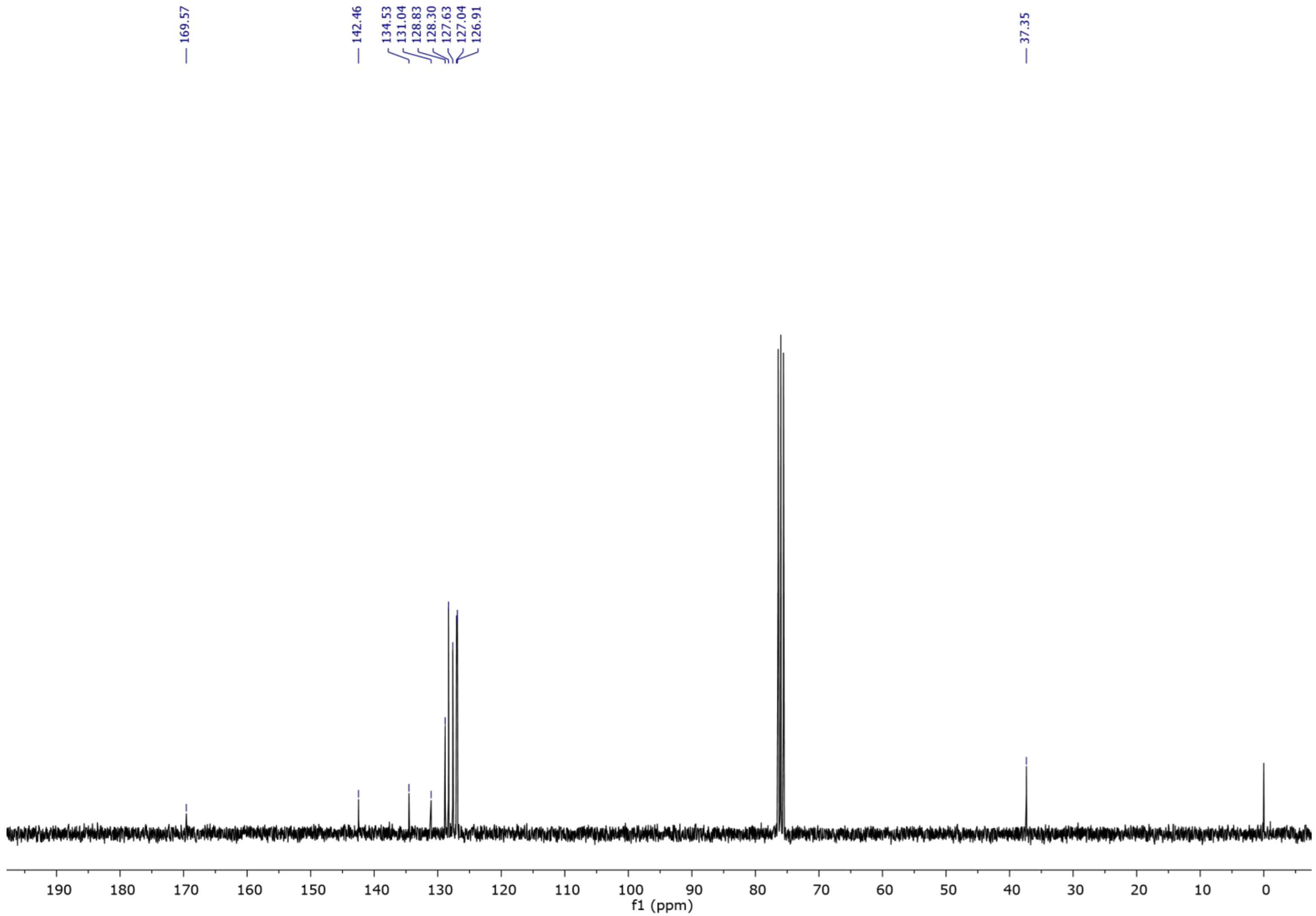

^{31}C NMR spectrum of **11e** (75 MHz, $CDCl_3$)

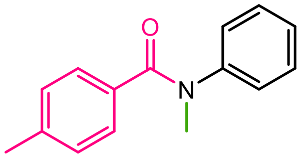

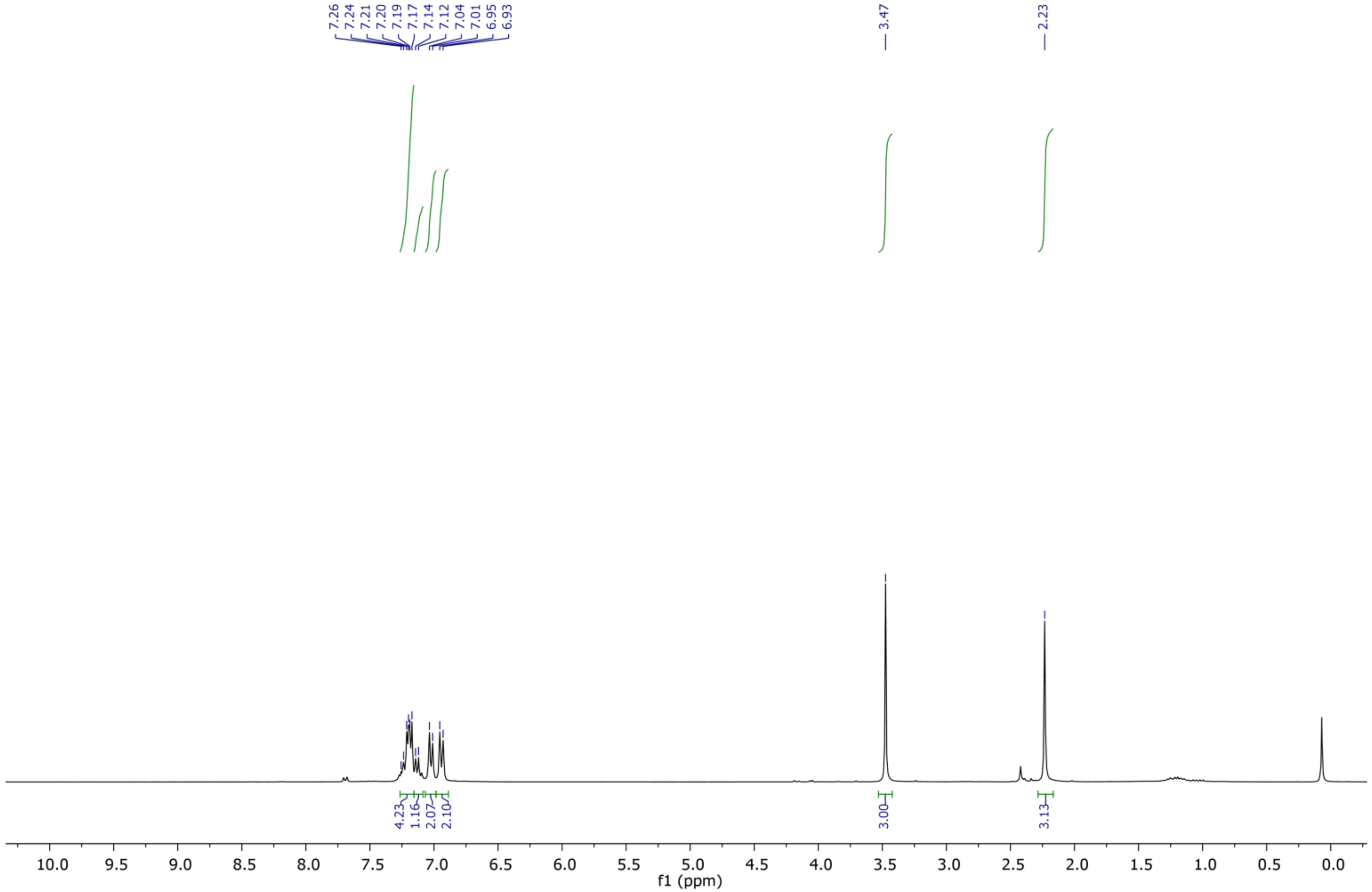

^{1}H NMR spectrum of **11f** (300 MHz, $CDCl_3$)

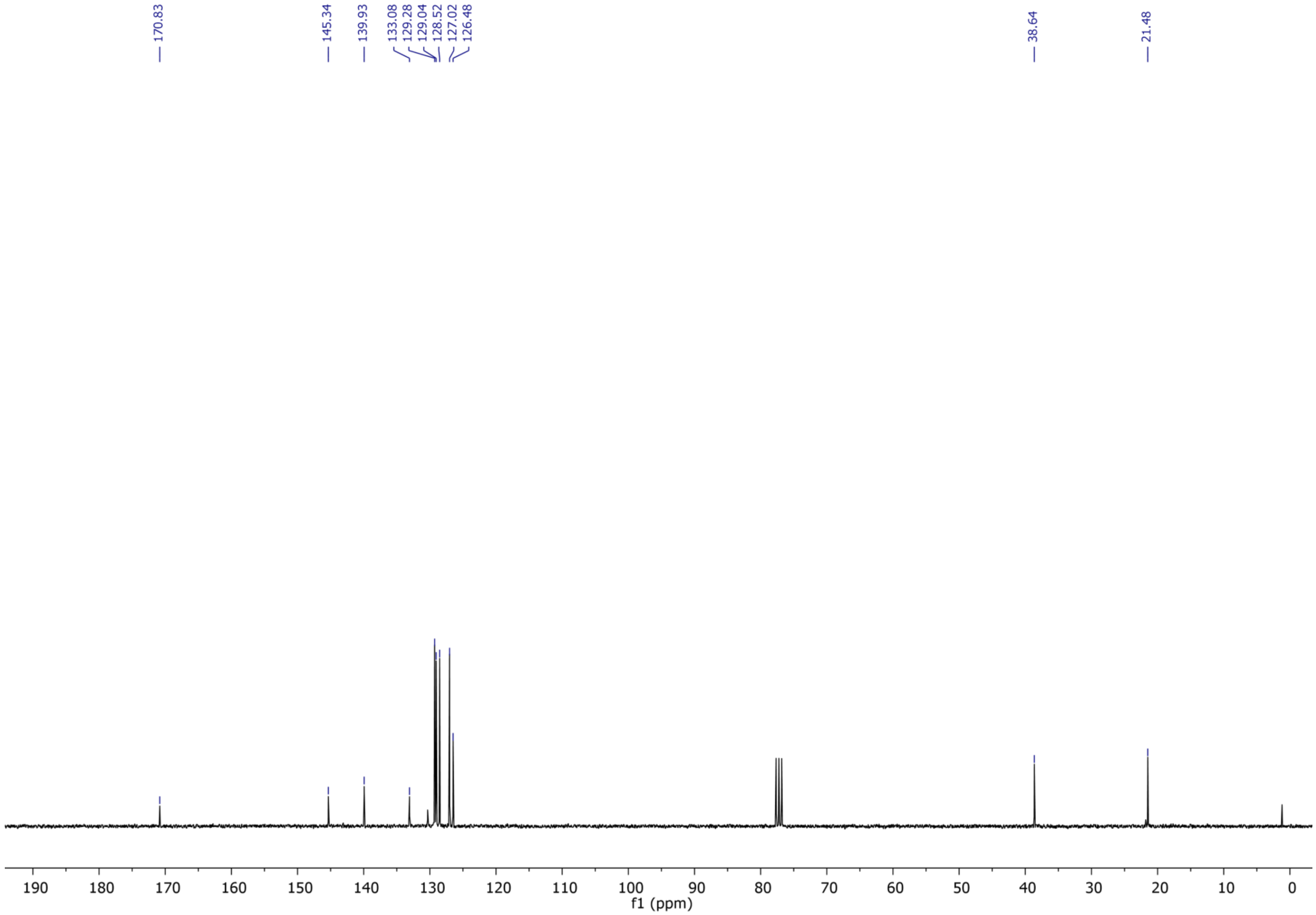

^{13}C NMR spectrum of **11f** (75 MHz, $CDCl_3$)

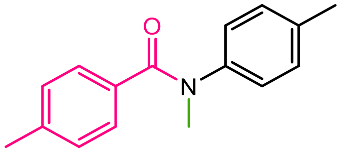

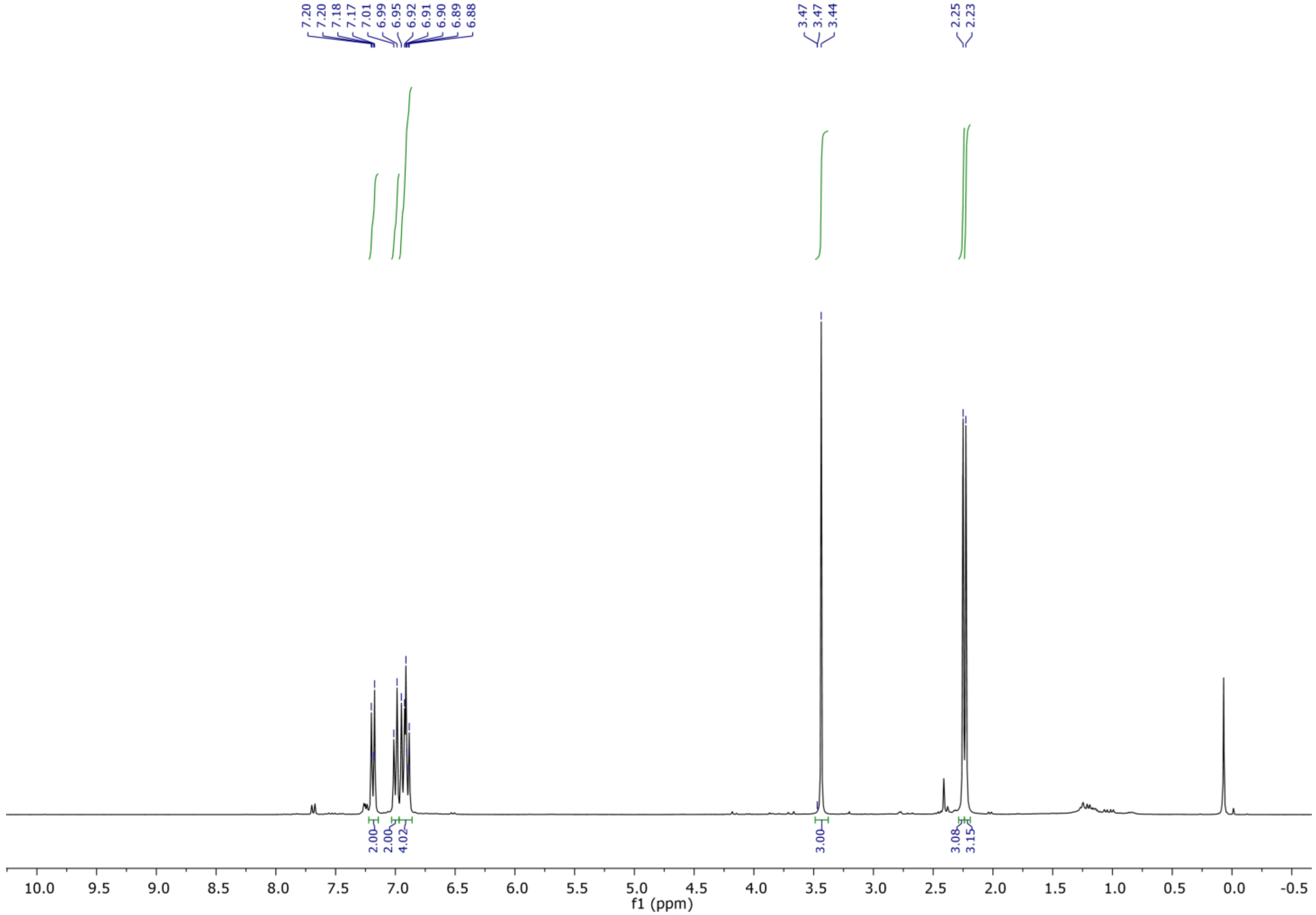

^{1}H NMR spectrum of **11g** (300 MHz, $CDCl_3$)

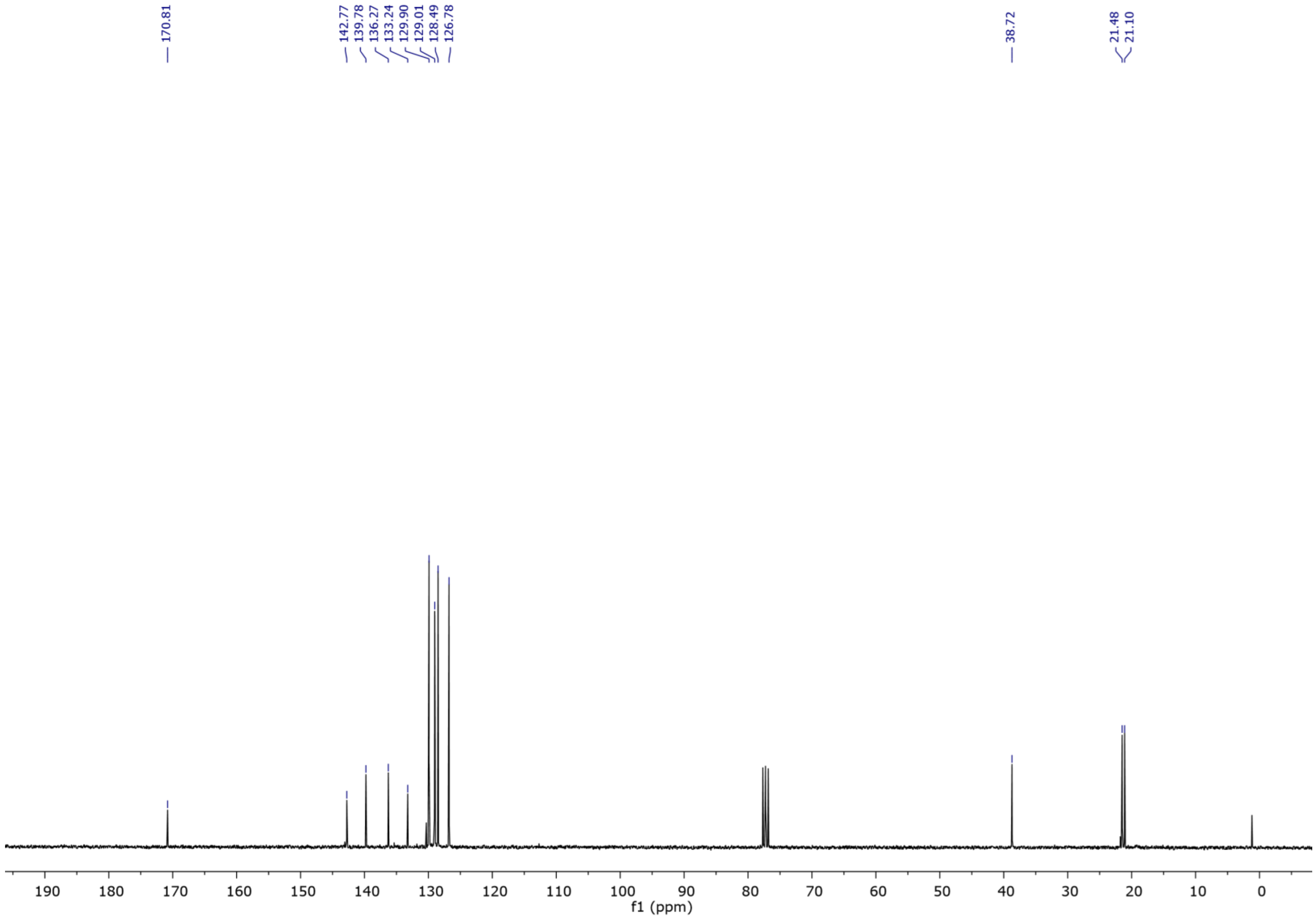

^{13}C NMR spectrum of **11g** (75 MHz, $CDCl_3$)

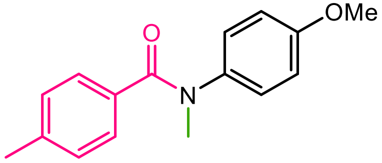

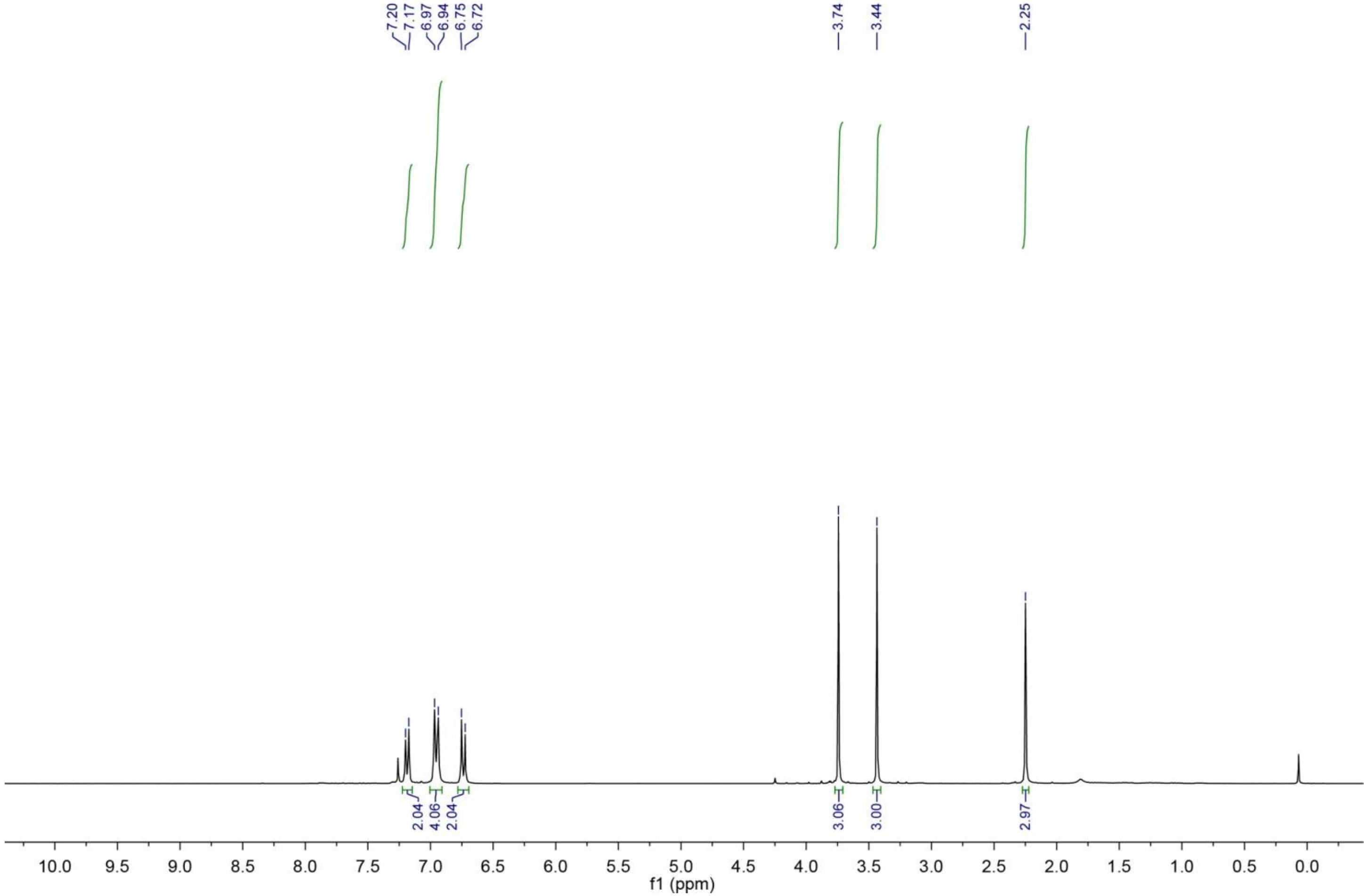

^{1}H NMR spectrum of **11h** (300 MHz, $CDCl_3$)

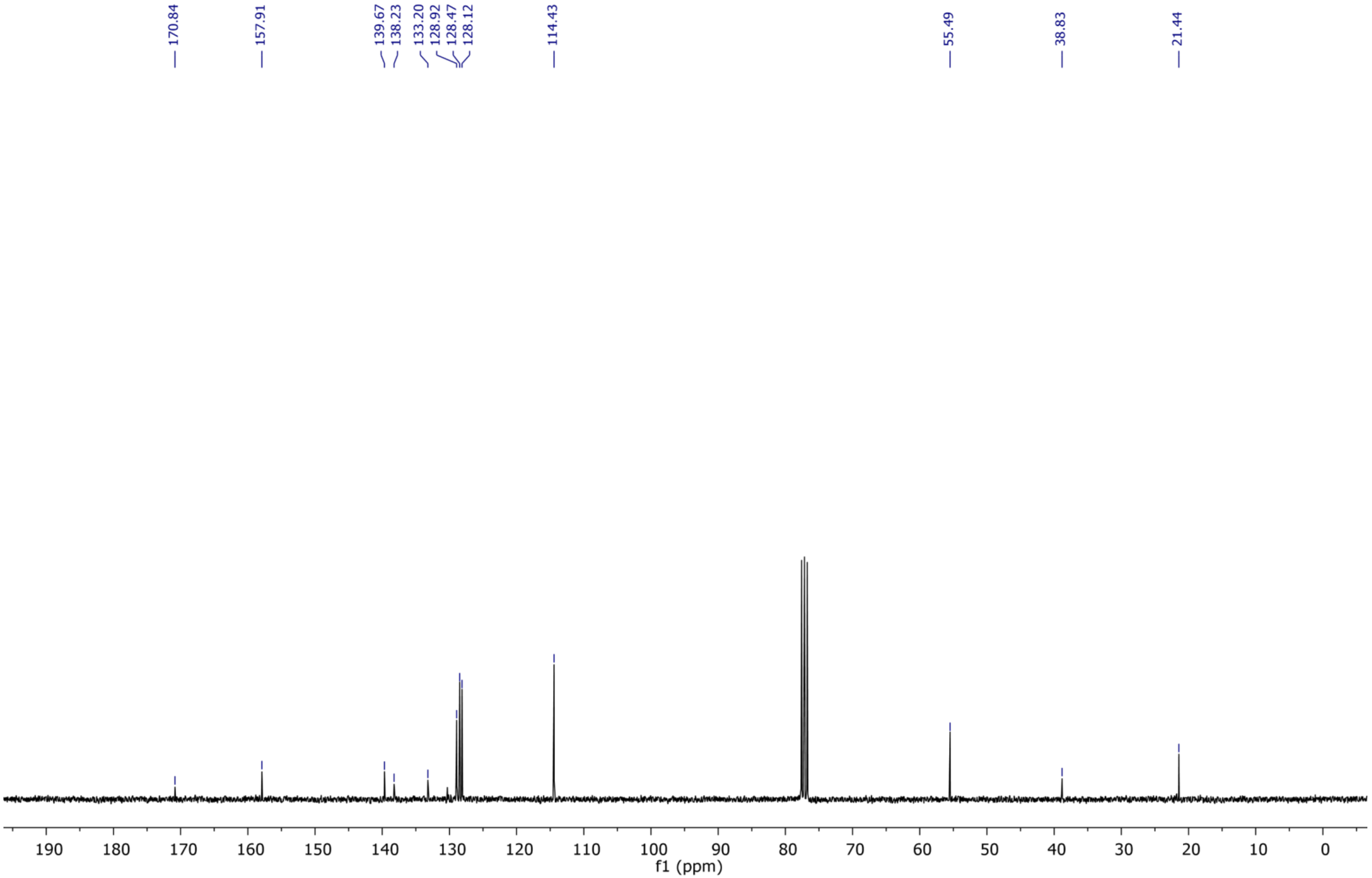

^{13}C NMR spectrum of **11h** (75 MHz, $CDCl_3$)

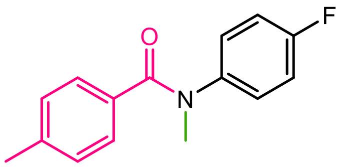

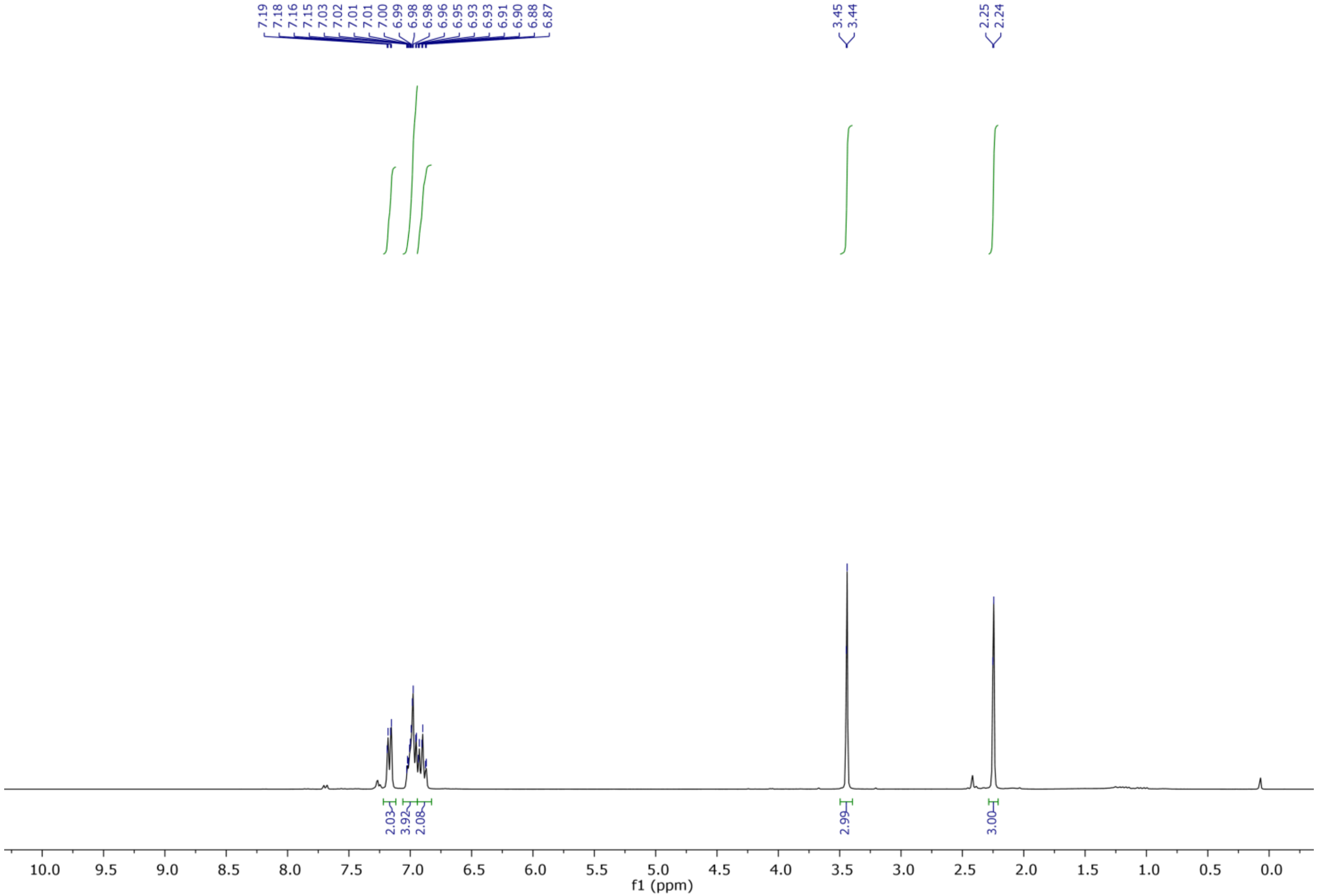

^{1}H NMR spectrum of **11i** (300 MHz, $CDCl_3$)

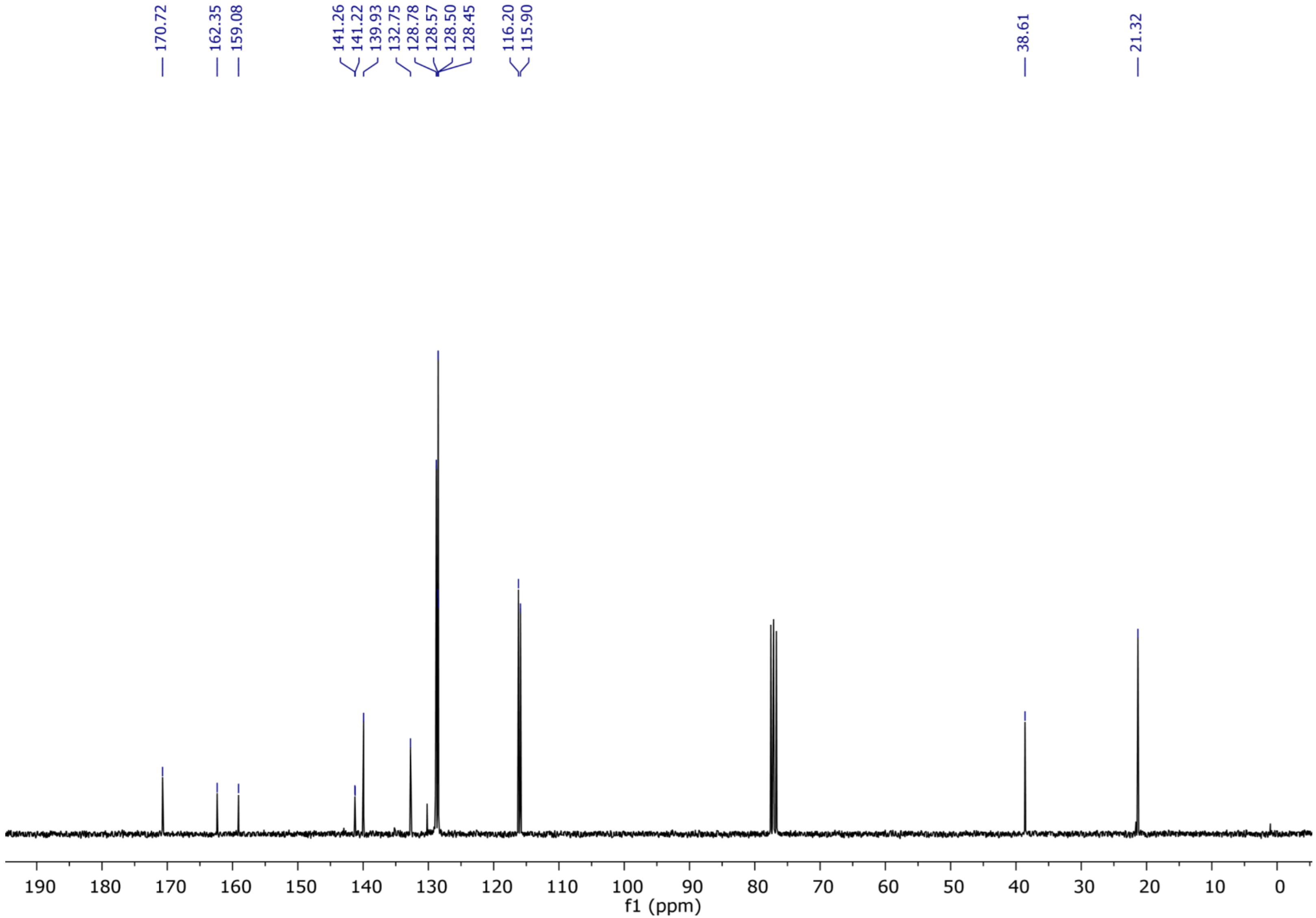

^{13}C NMR spectrum of **11i** (75 MHz, $CDCl_3$)

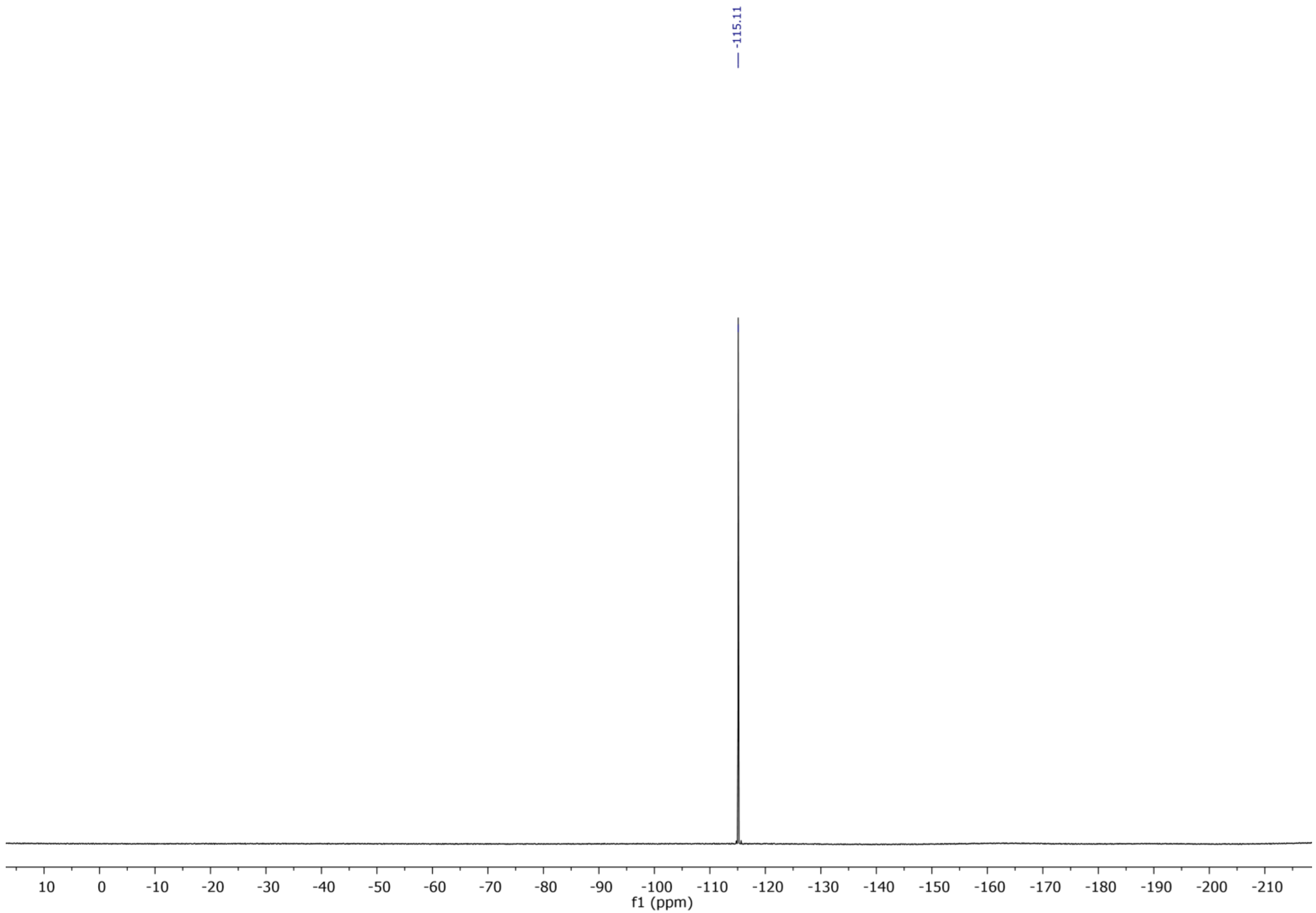

^{19}F NMR spectrum of **11i** (282 MHz, $CDCl_3$)

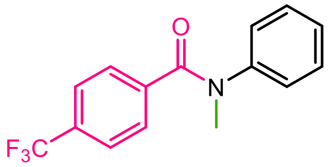

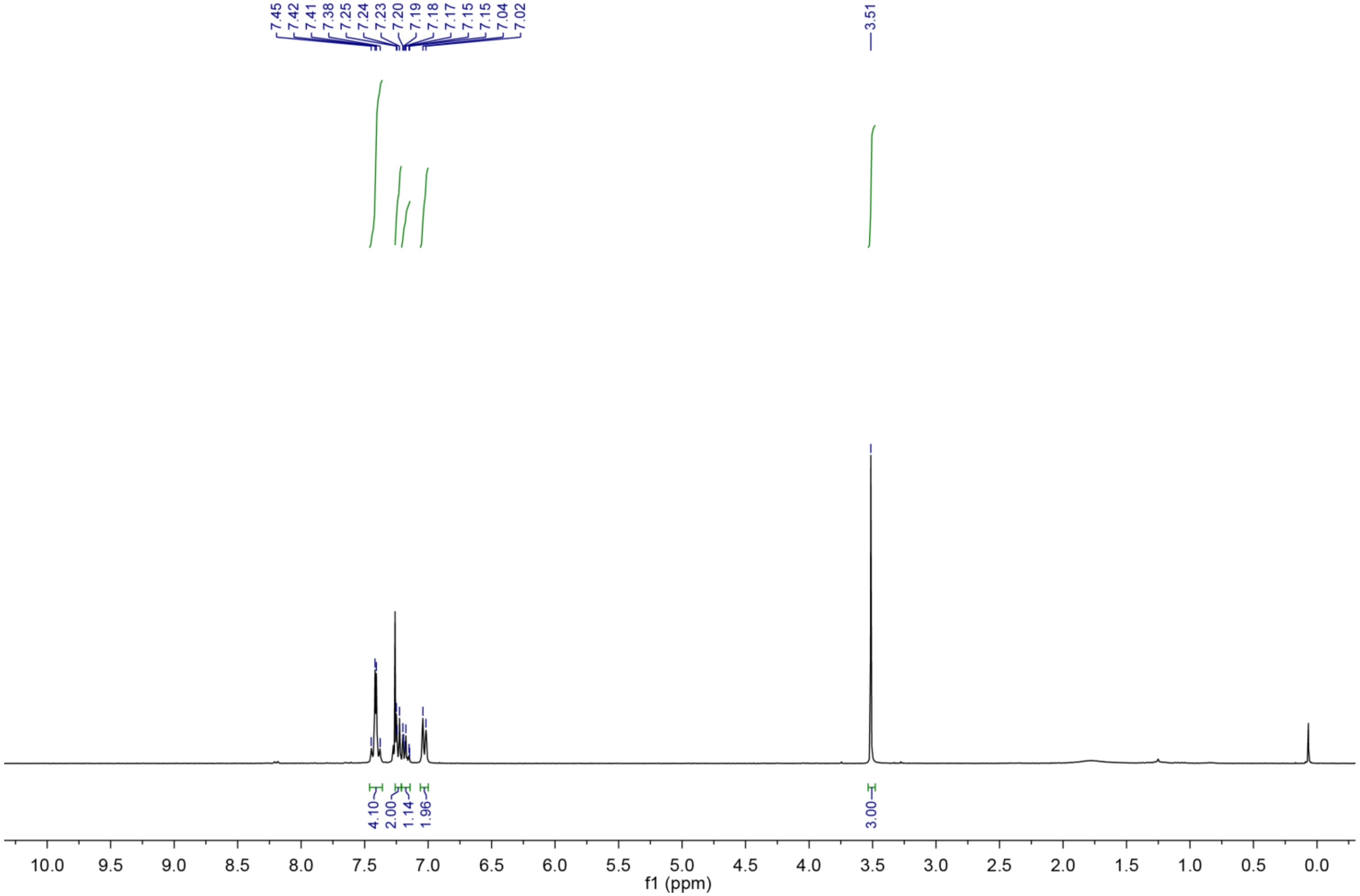

^{1}H NMR spectrum of **11j** (300 MHz, $CDCl_3$)

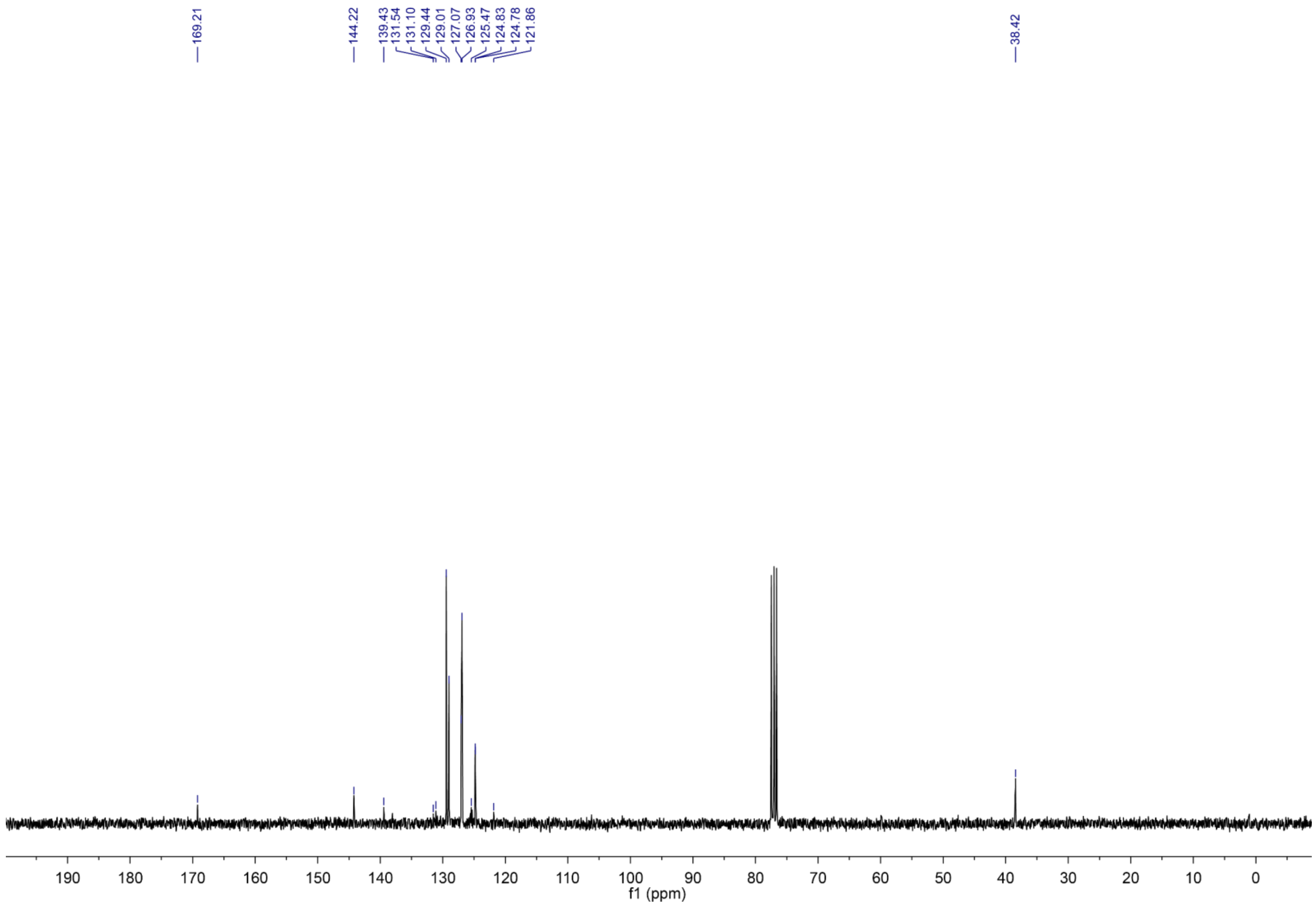

^{13}C NMR spectrum of **11j** (75 MHz, $CDCl_3$)

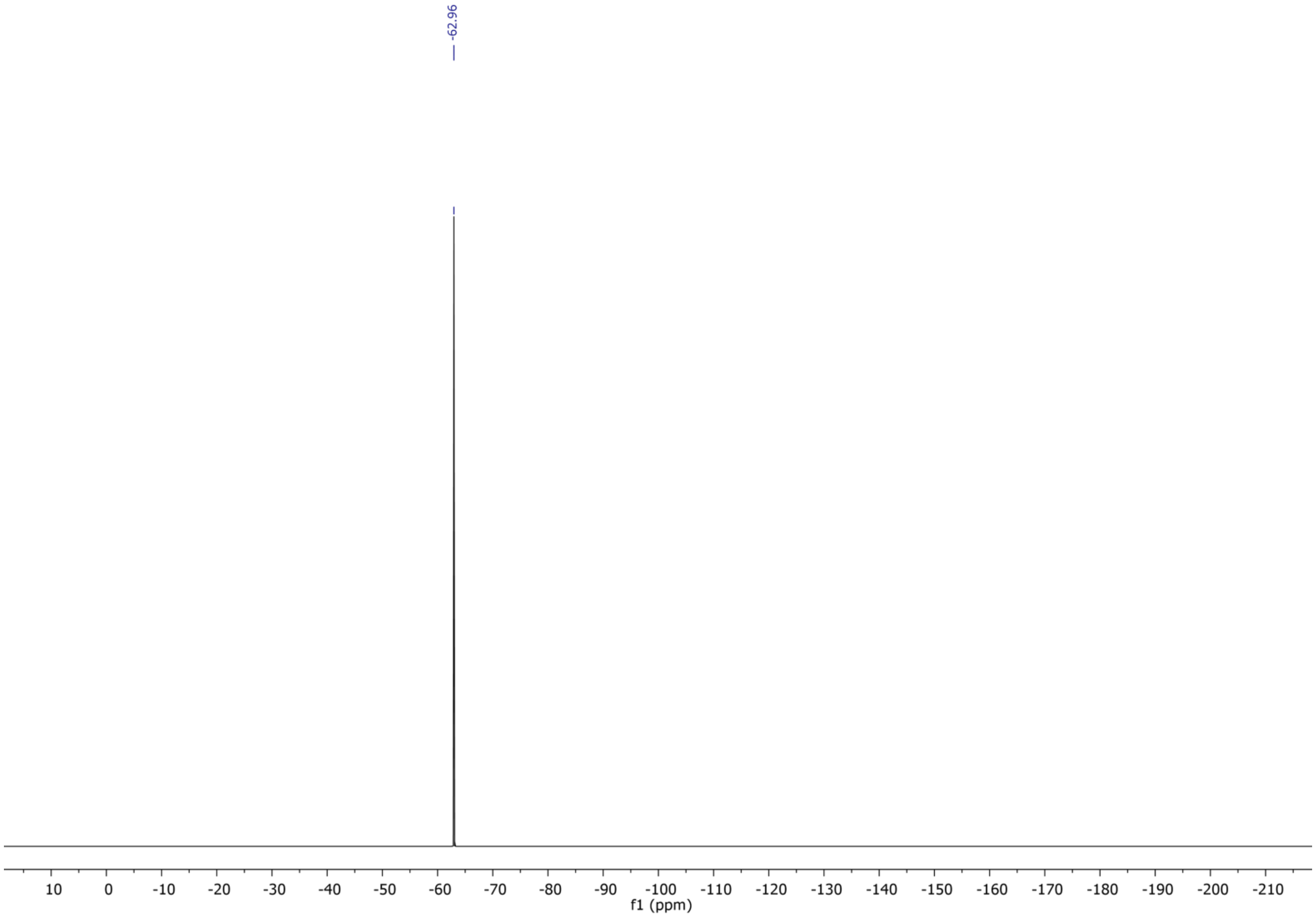

^{19}F NMR spectrum of **11j** (282 MHz, $CDCl_3$)

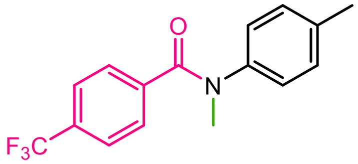

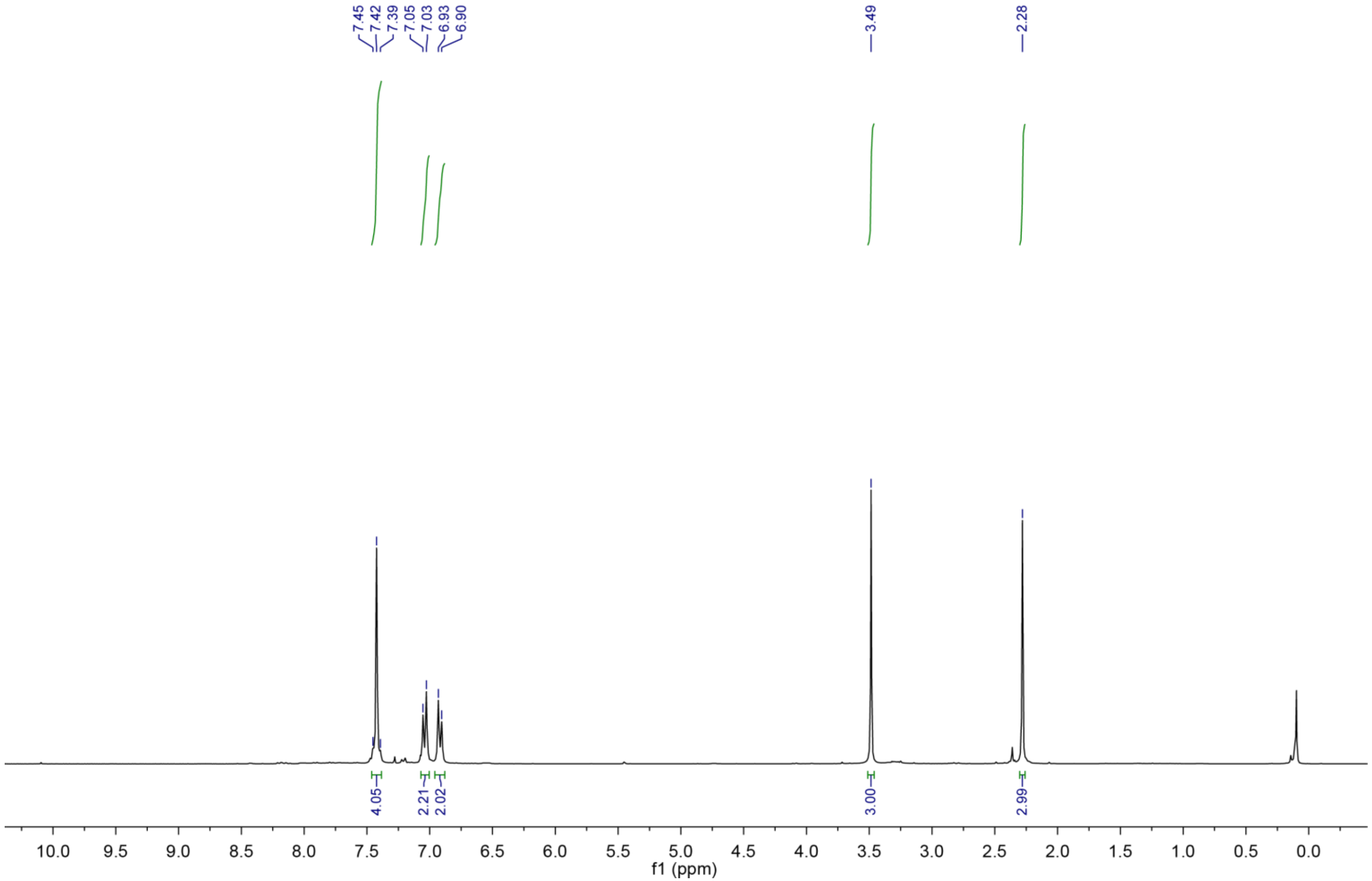

^{1}H NMR spectrum of **11k** (300 MHz, $CDCl_3$)

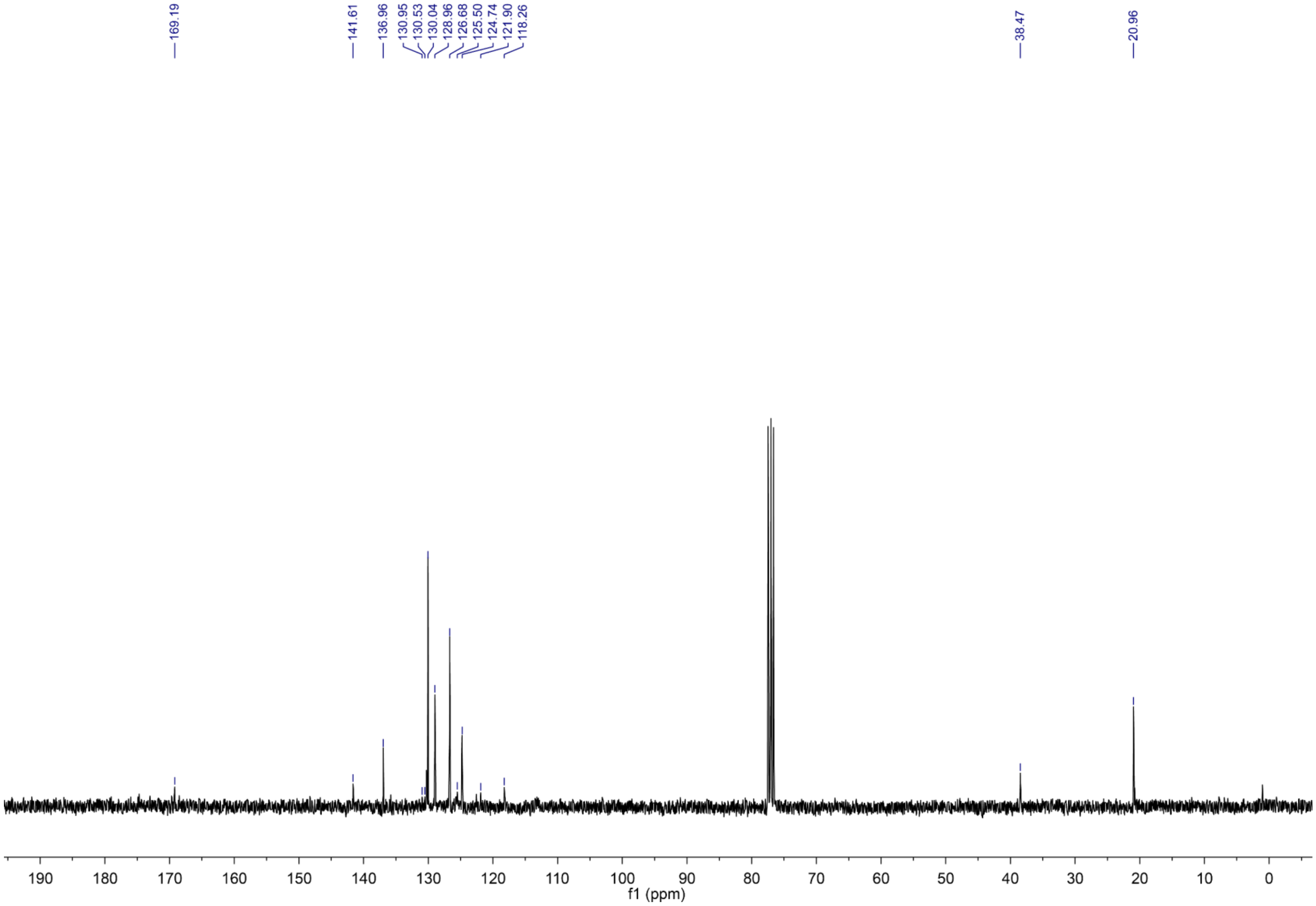

^{13}C NMR spectrum of **11k** (75 MHz, $CDCl_3$)

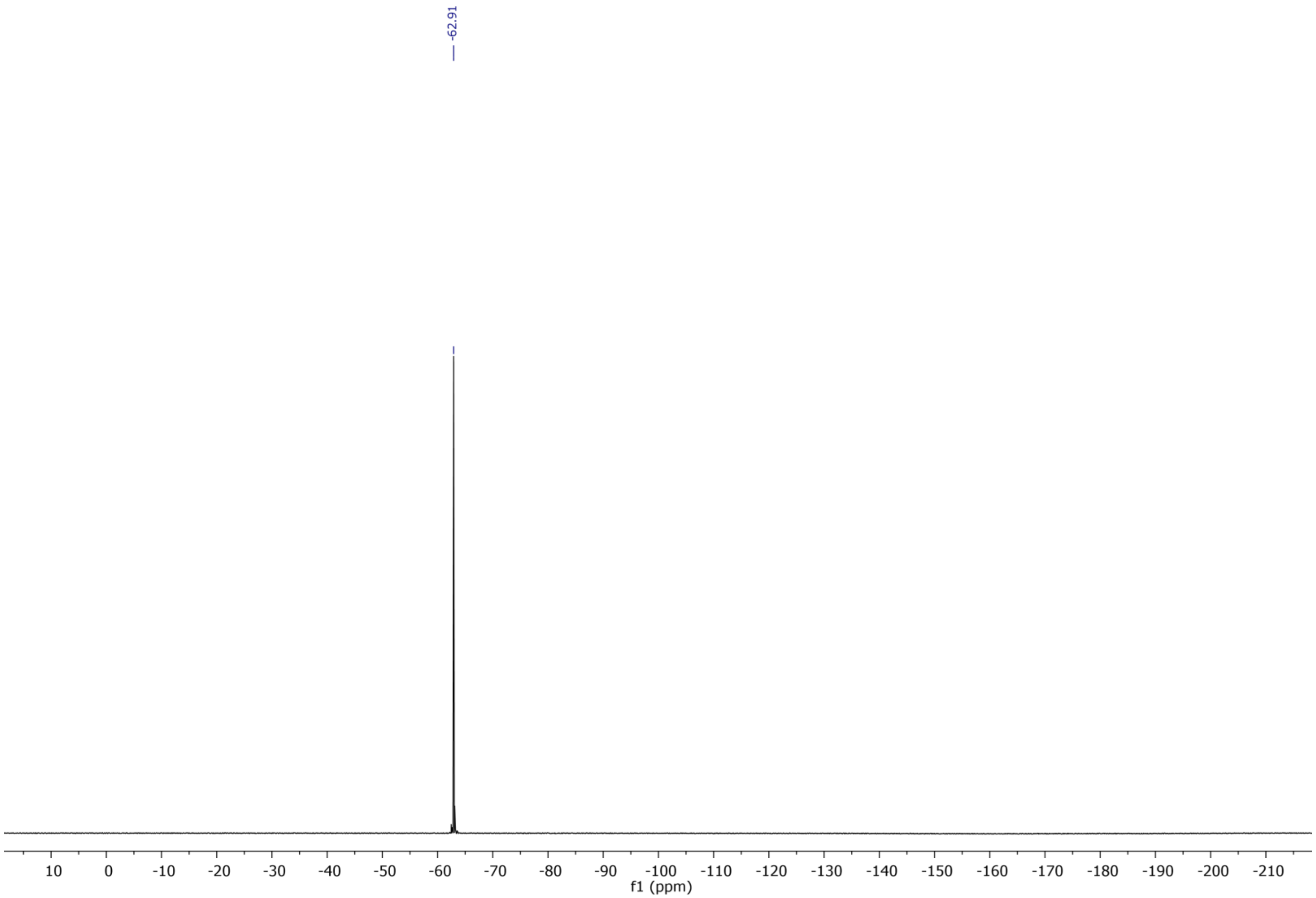

^{19}F NMR spectrum of **11k** (282 MHz, $CDCl_3$)

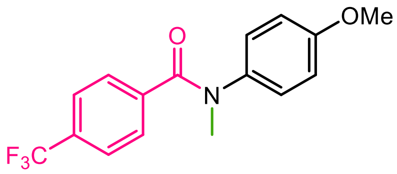

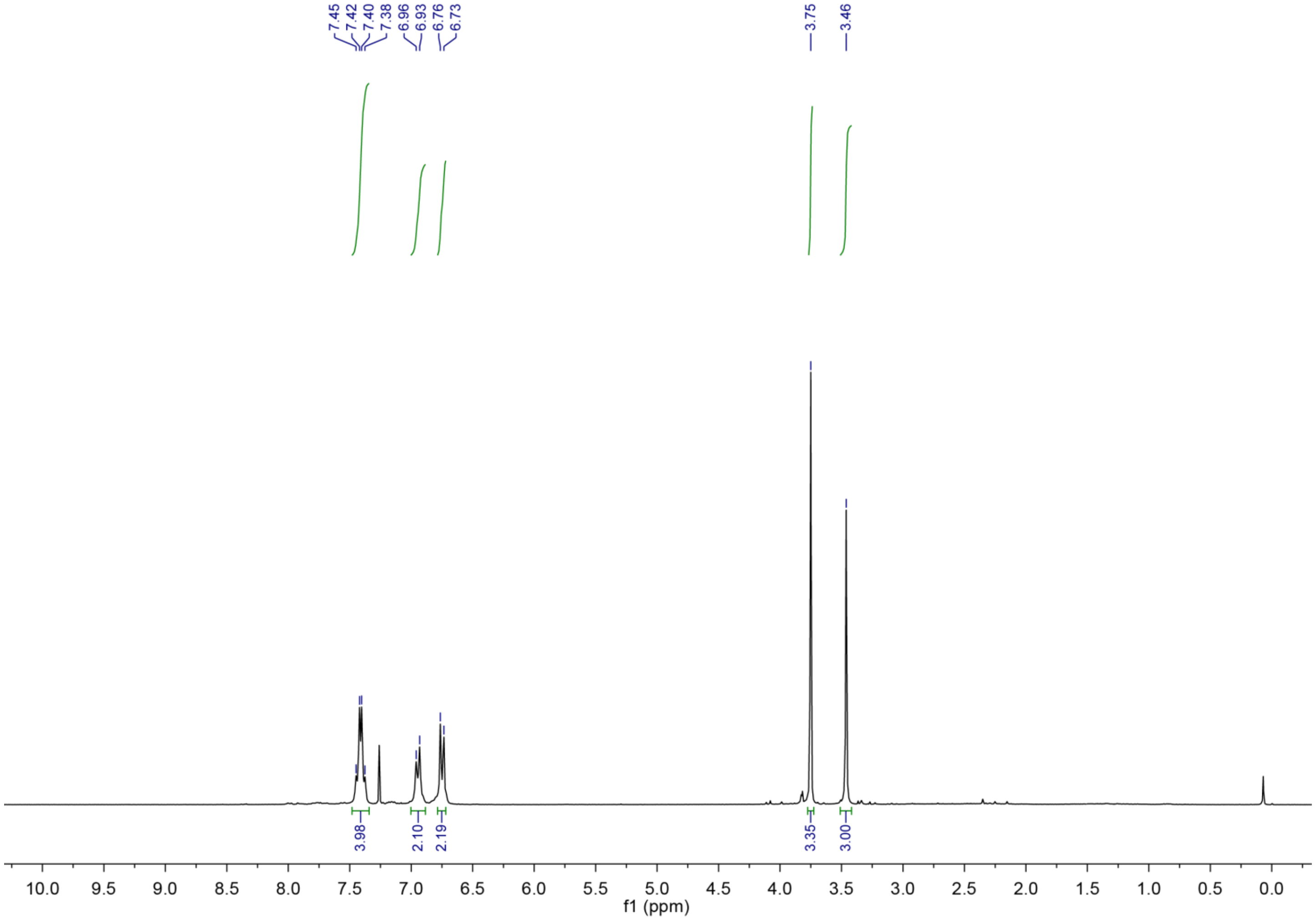

^{1}H NMR spectrum of **11l** (300 MHz, $CDCl_3$)

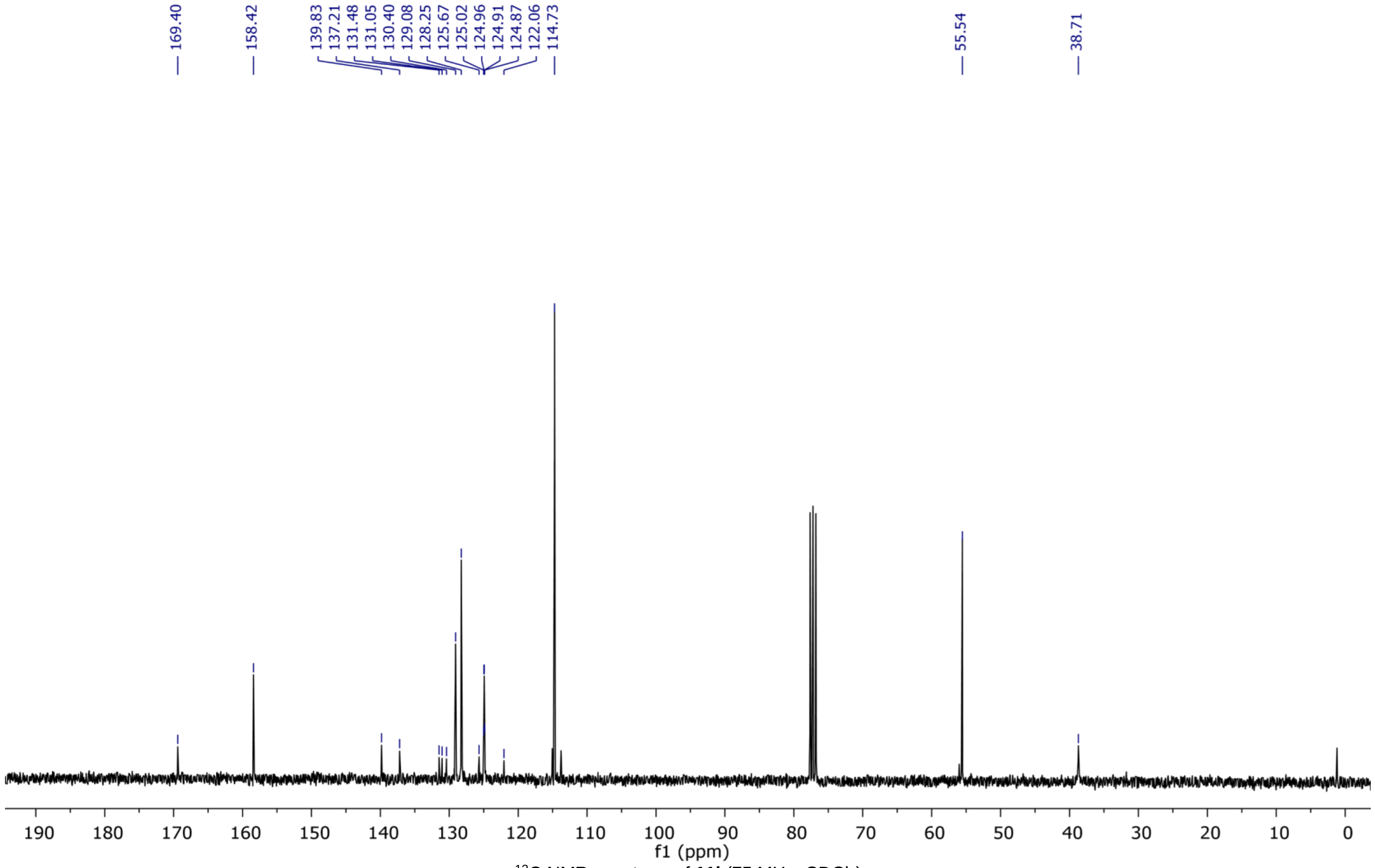

^{13}C NMR spectrum of **11l** (75 MHz, $CDCl_3$)

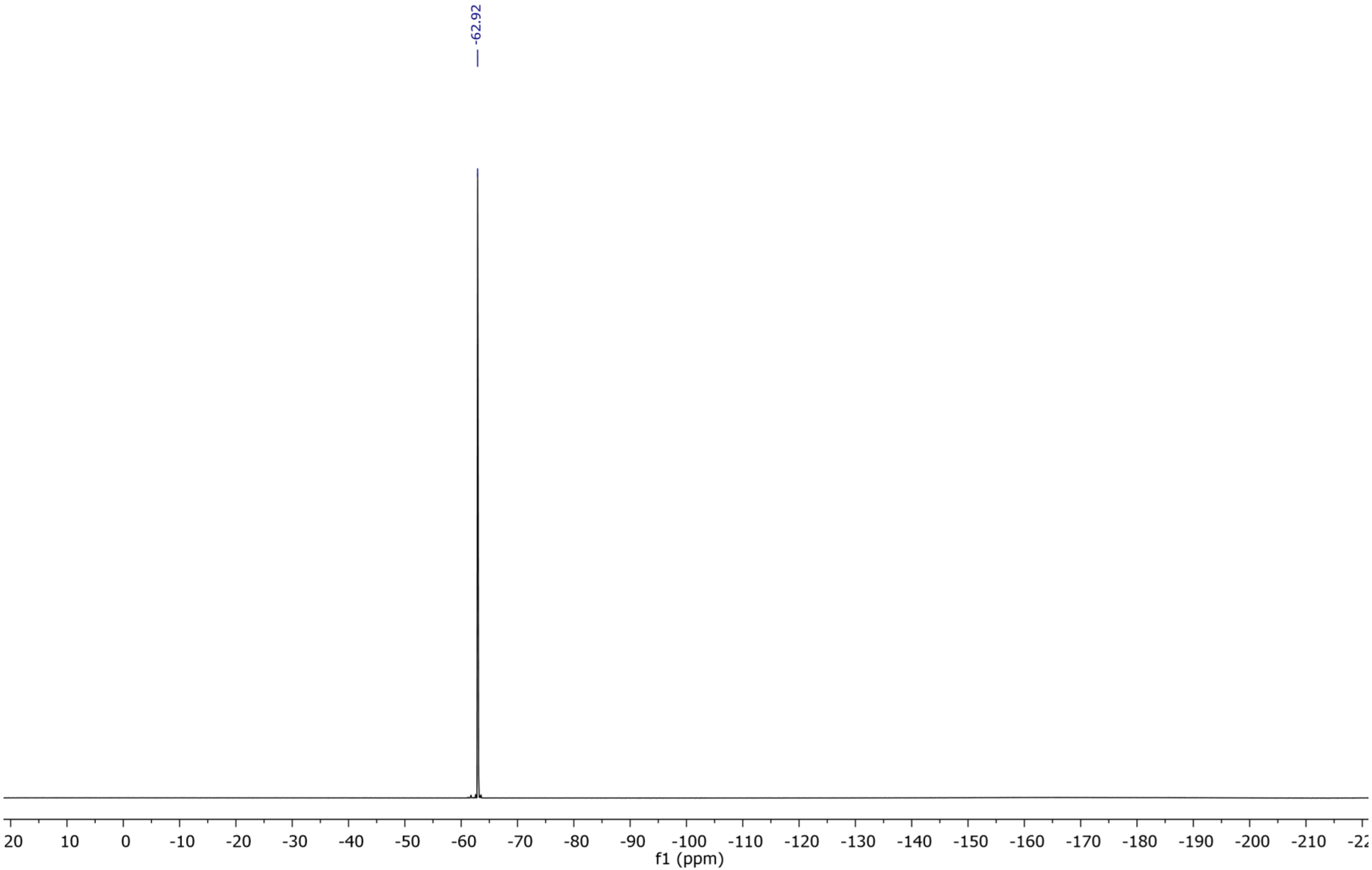

^{19}F NMR spectrum of **11l** (282 MHz, $CDCl_3$)

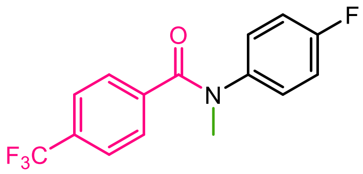

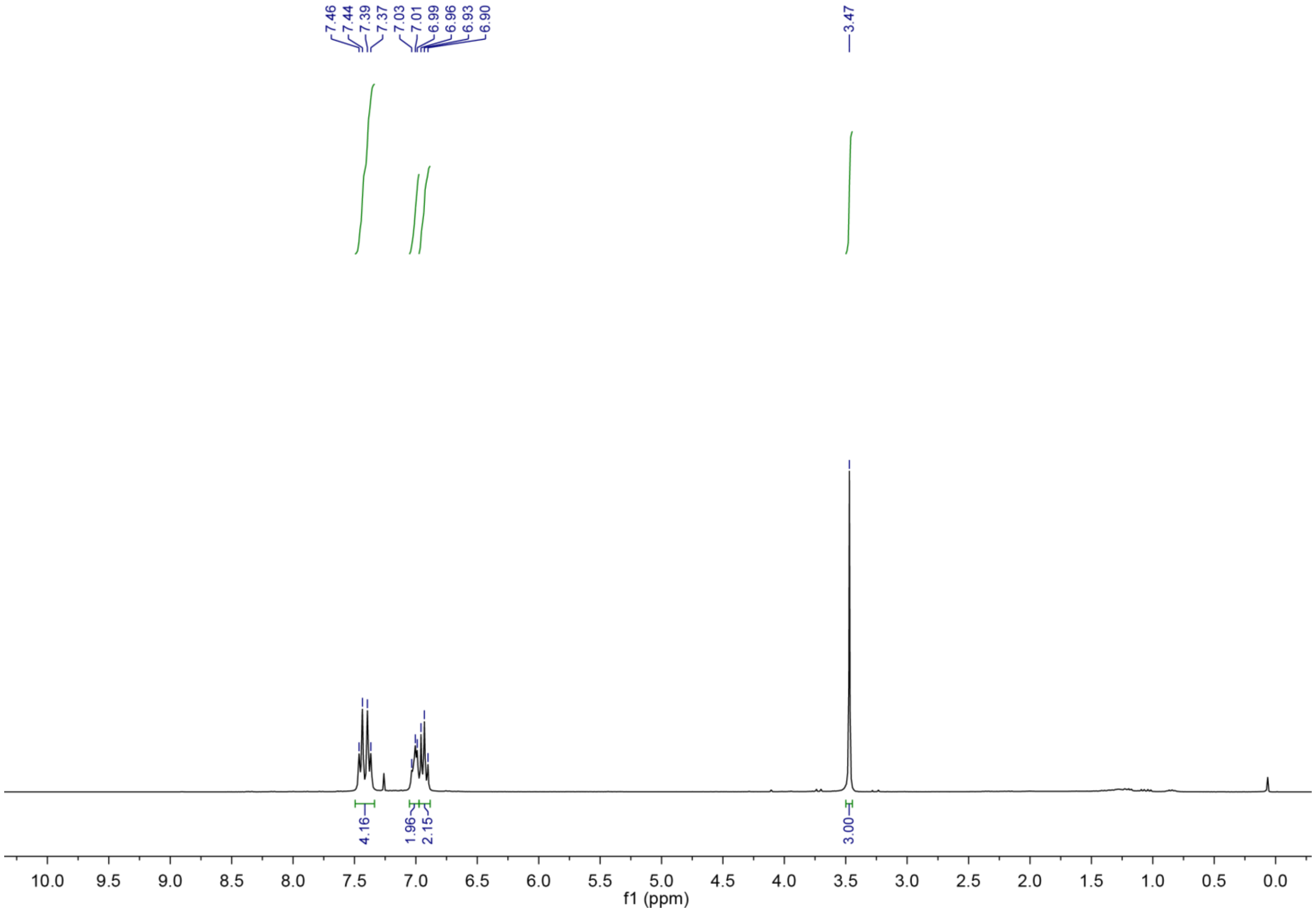

^{1}H NMR spectrum of **11m** (300 MHz, $CDCl_3$)

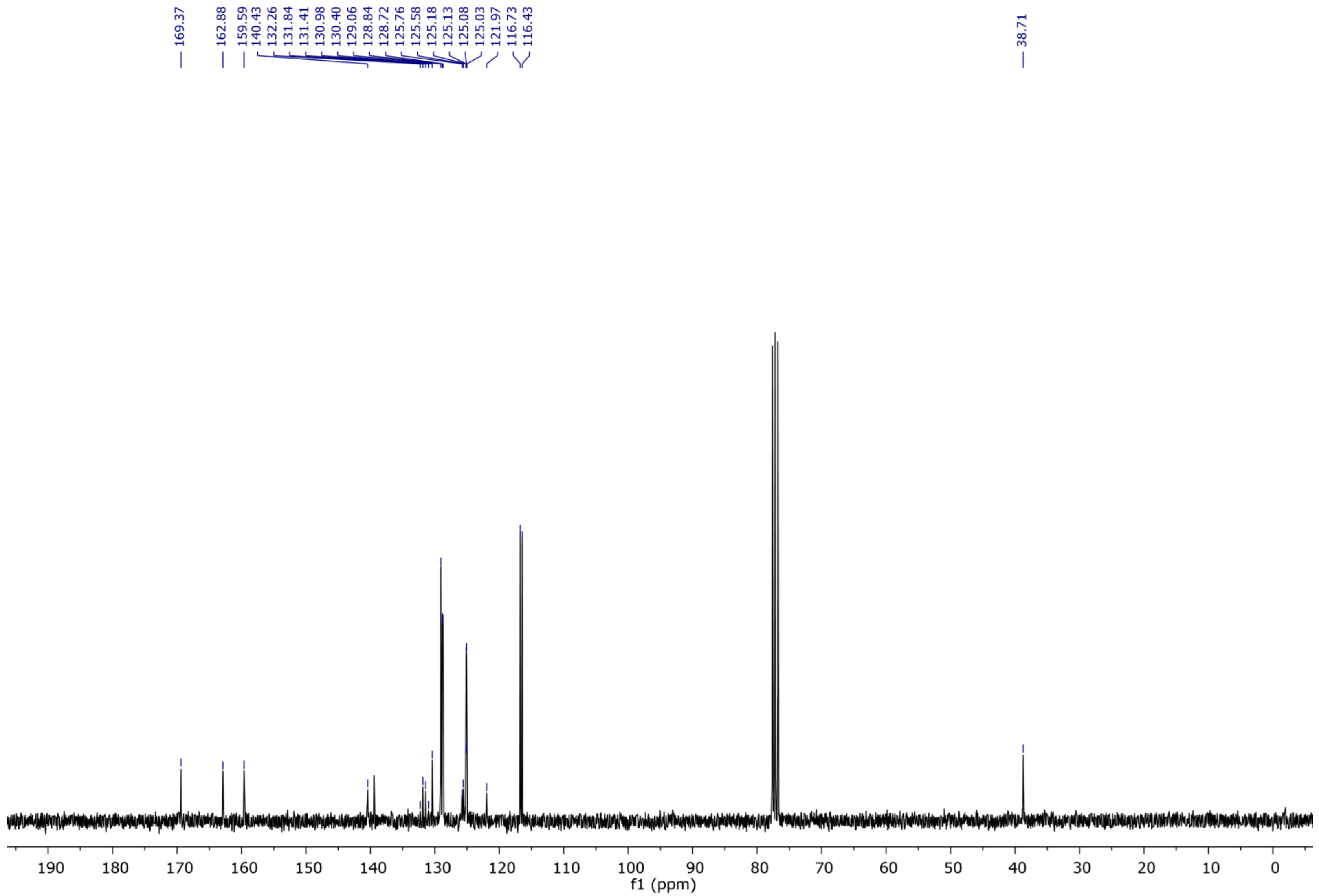

^{13}C NMR spectrum of **11m** (75 MHz, $CDCl_3$)

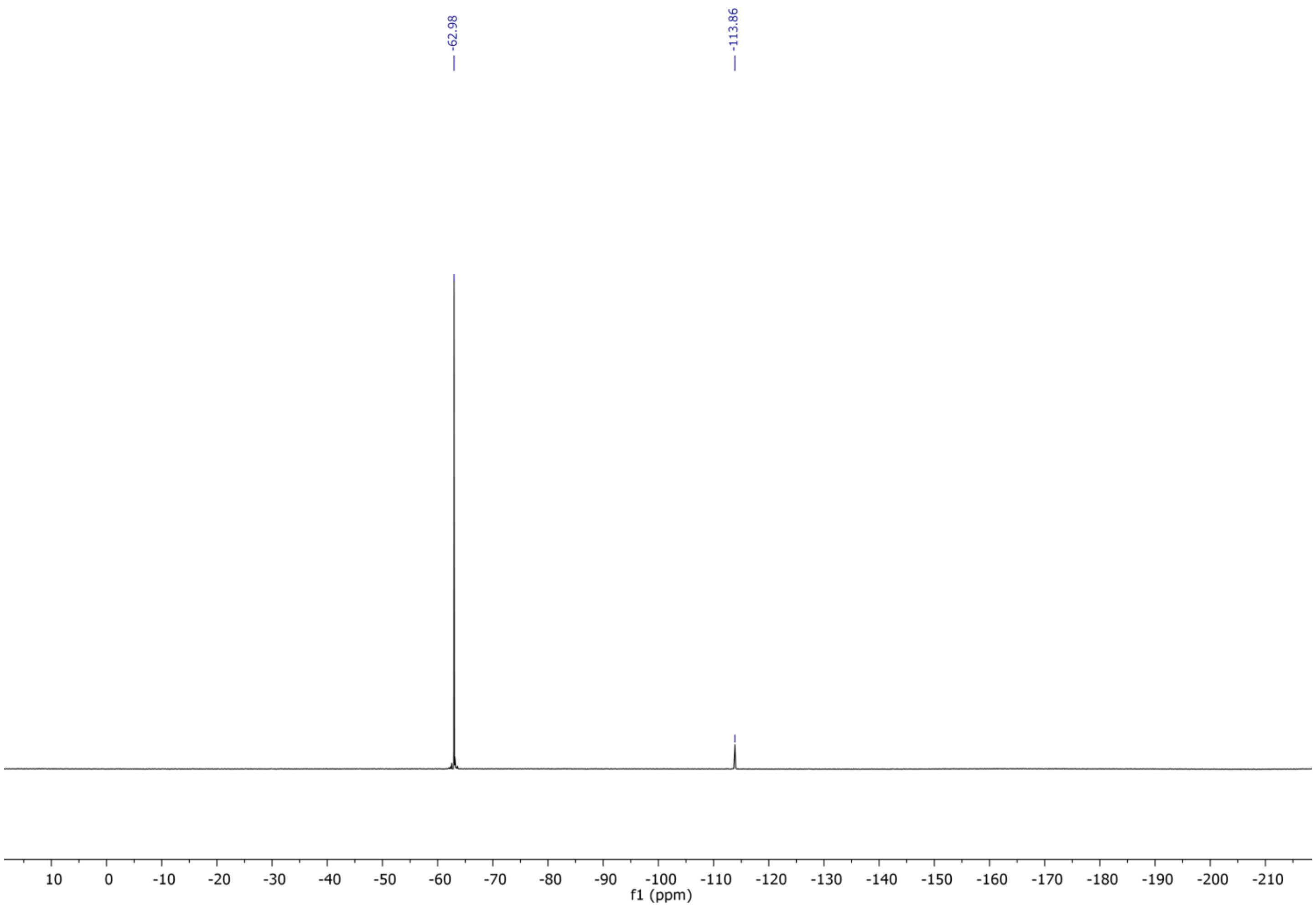

^{19}F NMR spectrum of **11m** (282 MHz, $CDCl_3$)

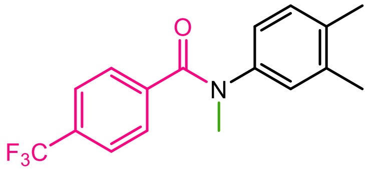

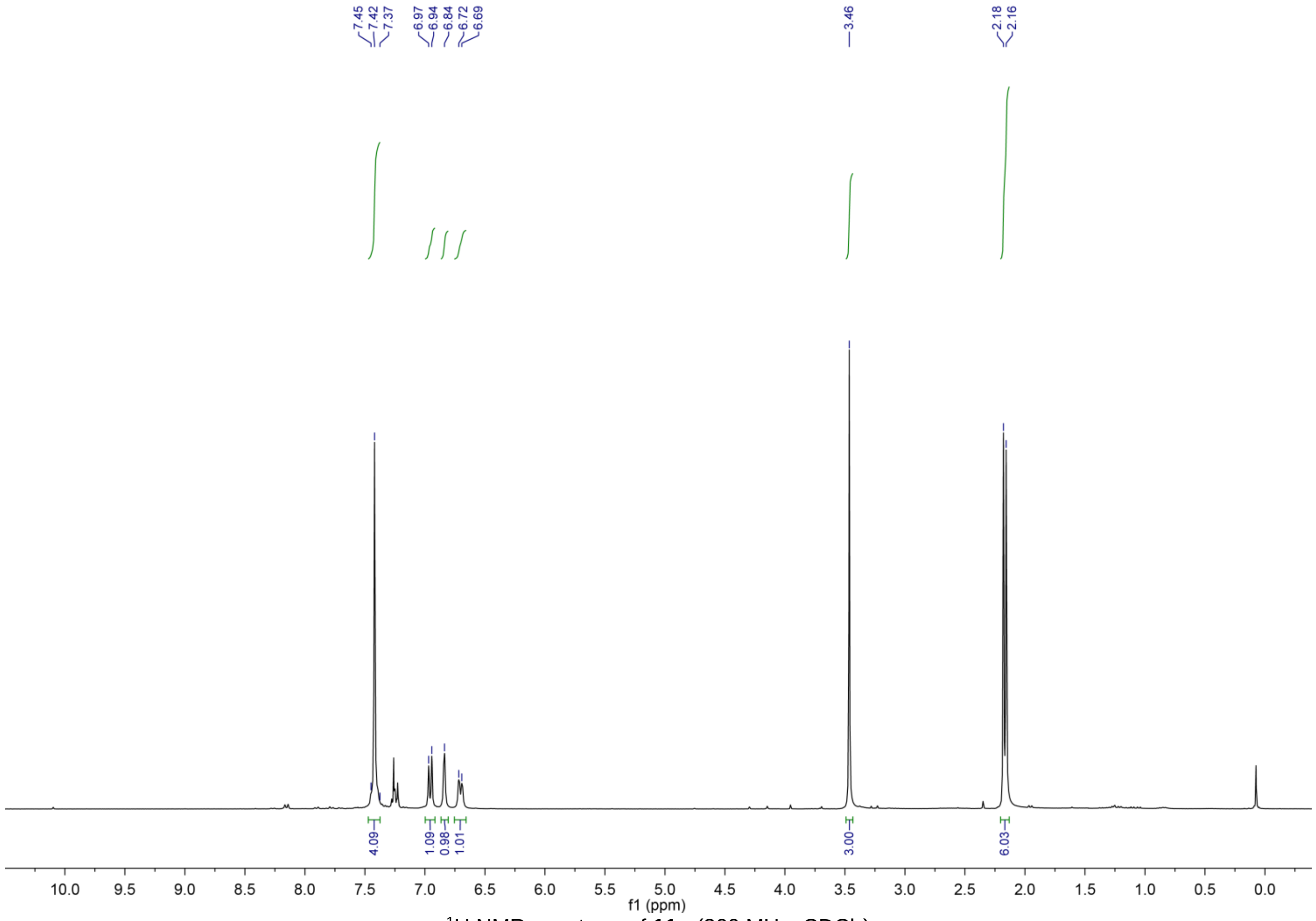

^{1}H NMR spectrum of **11n** (300 MHz, $CDCl_3$)

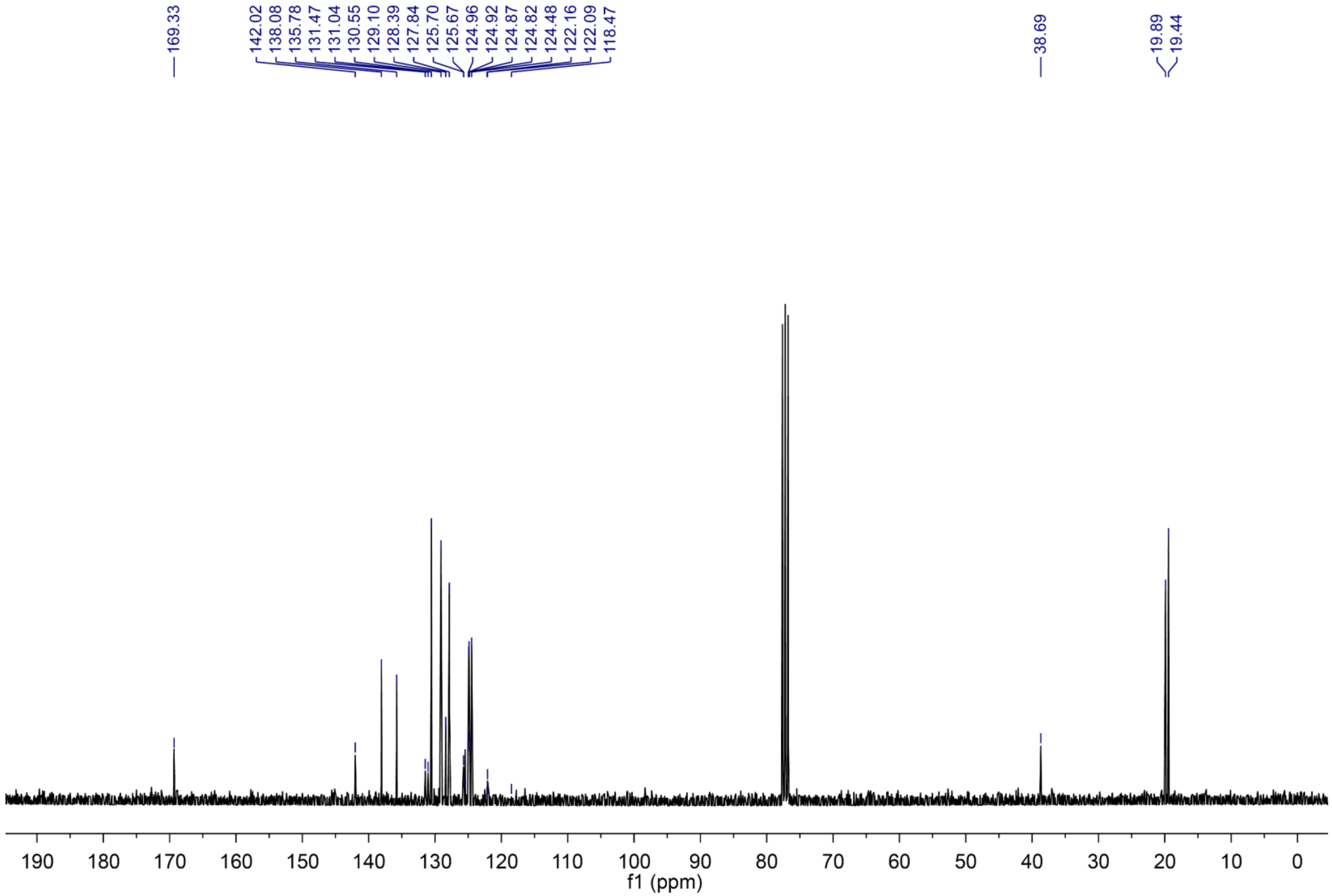

^{13}C NMR spectrum of **11n** (75 MHz, $CDCl_3$)

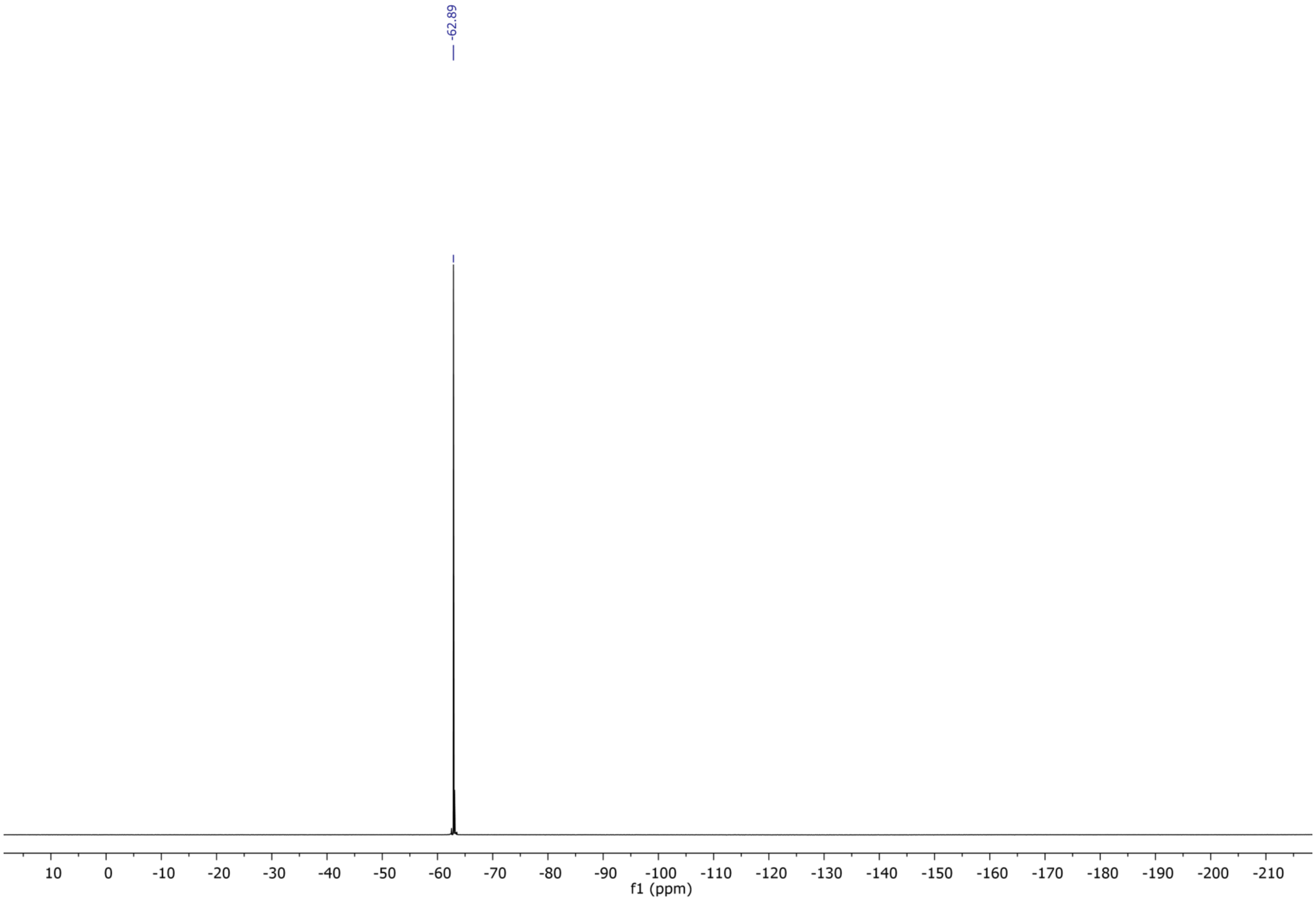

^{19}F NMR spectrum of **11n** (282 MHz, $CDCl_3$)

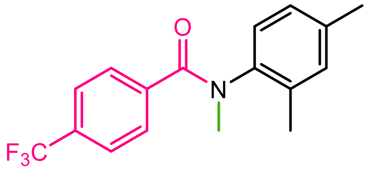

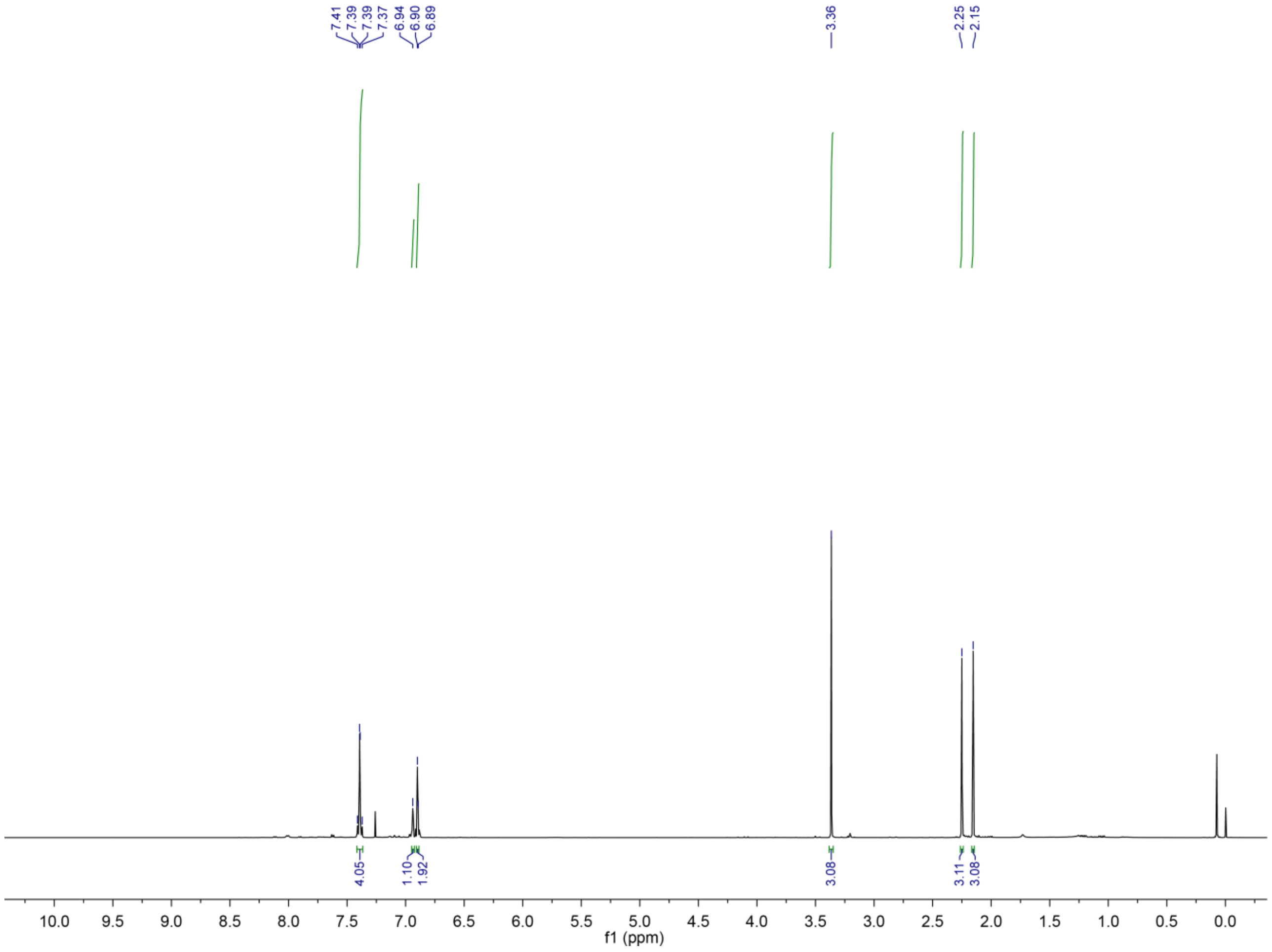

^{1}H NMR spectrum of **11o** (300 MHz, $CDCl_3$)

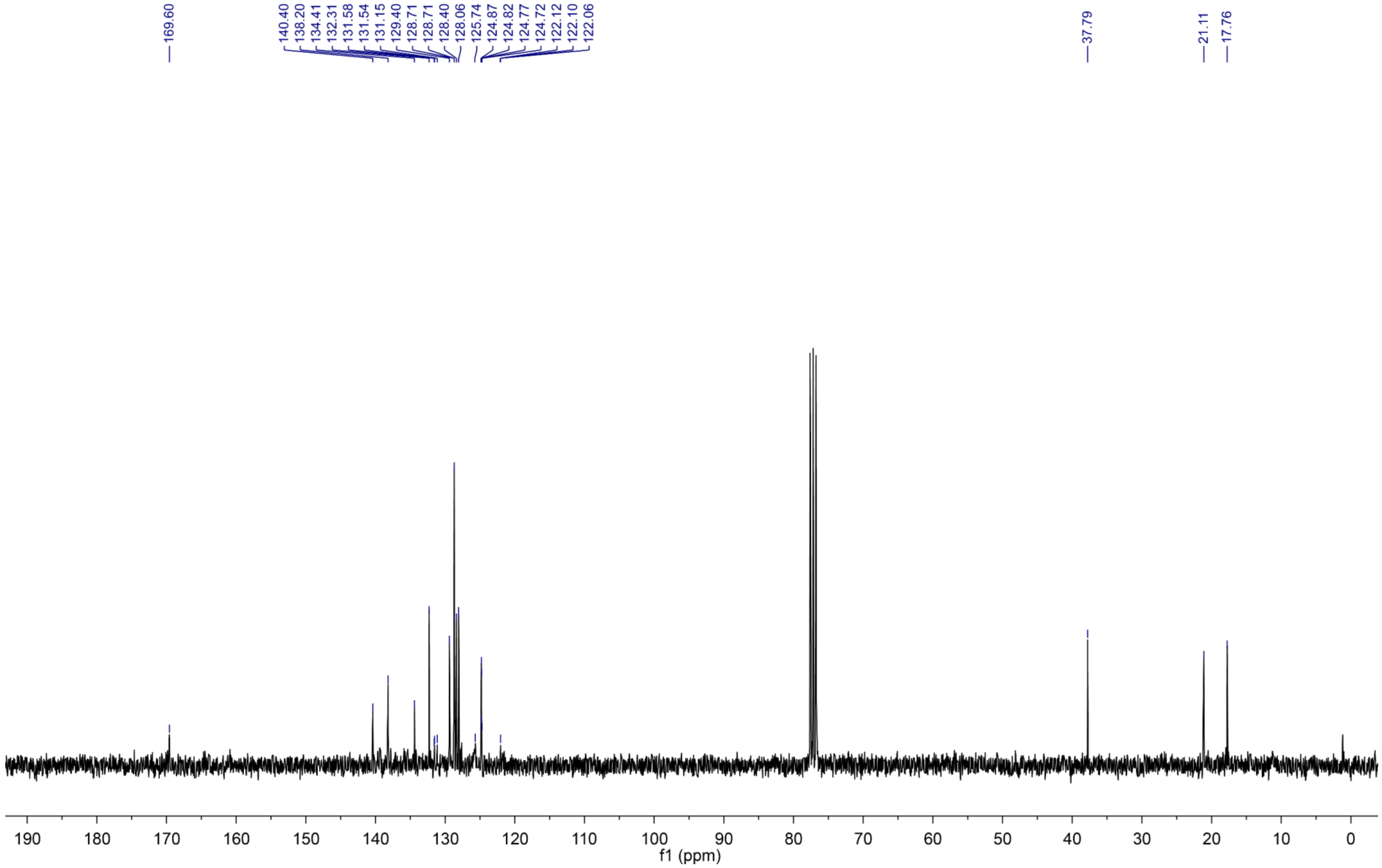

^{13}C NMR spectrum of **11o** (75 MHz, $CDCl_3$)

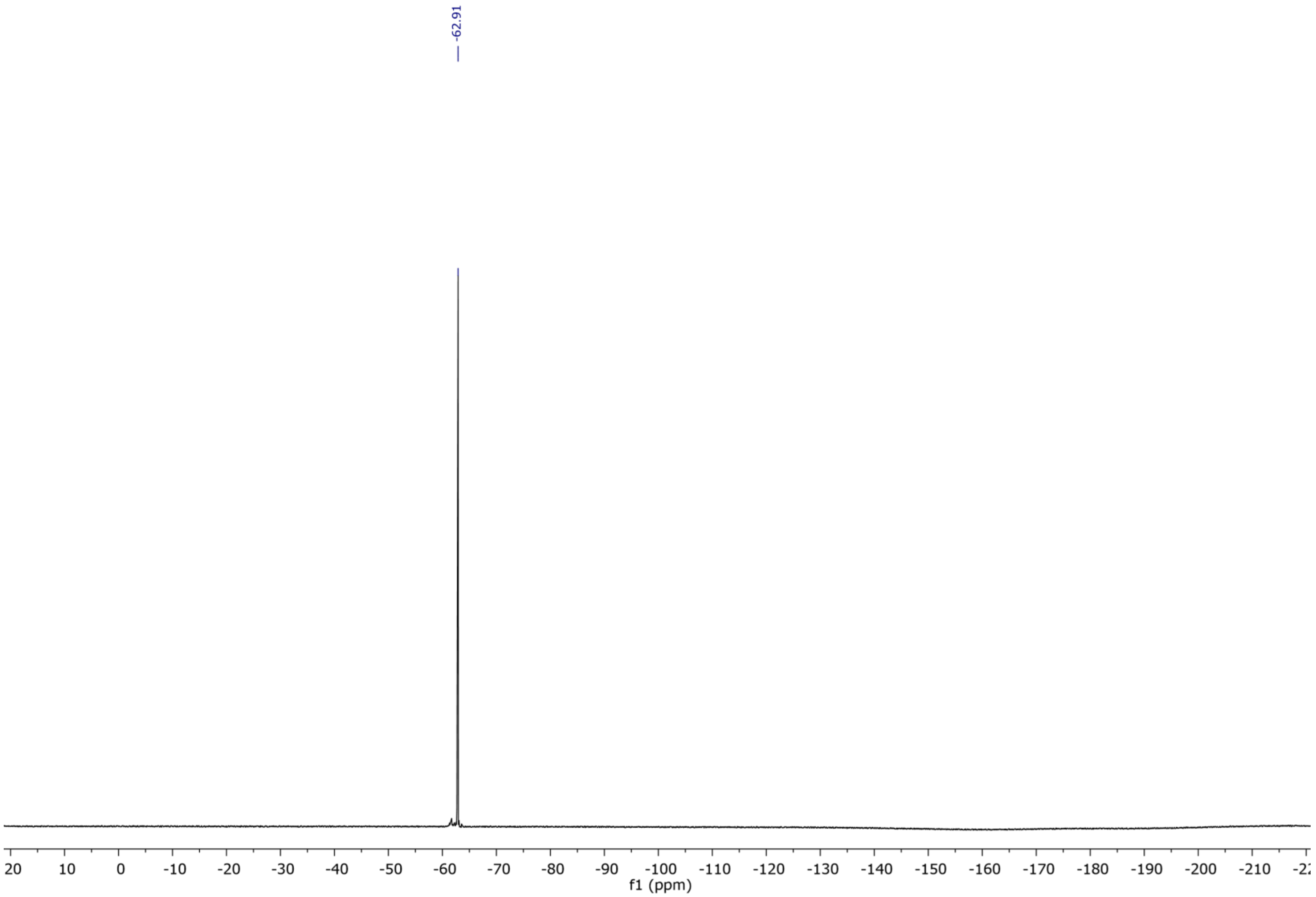

^{19}F NMR spectrum of **11o** in CDCl3 (300 MHz, CDCl3)

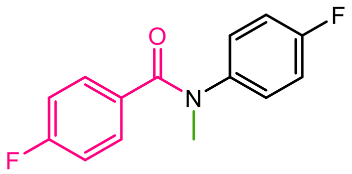

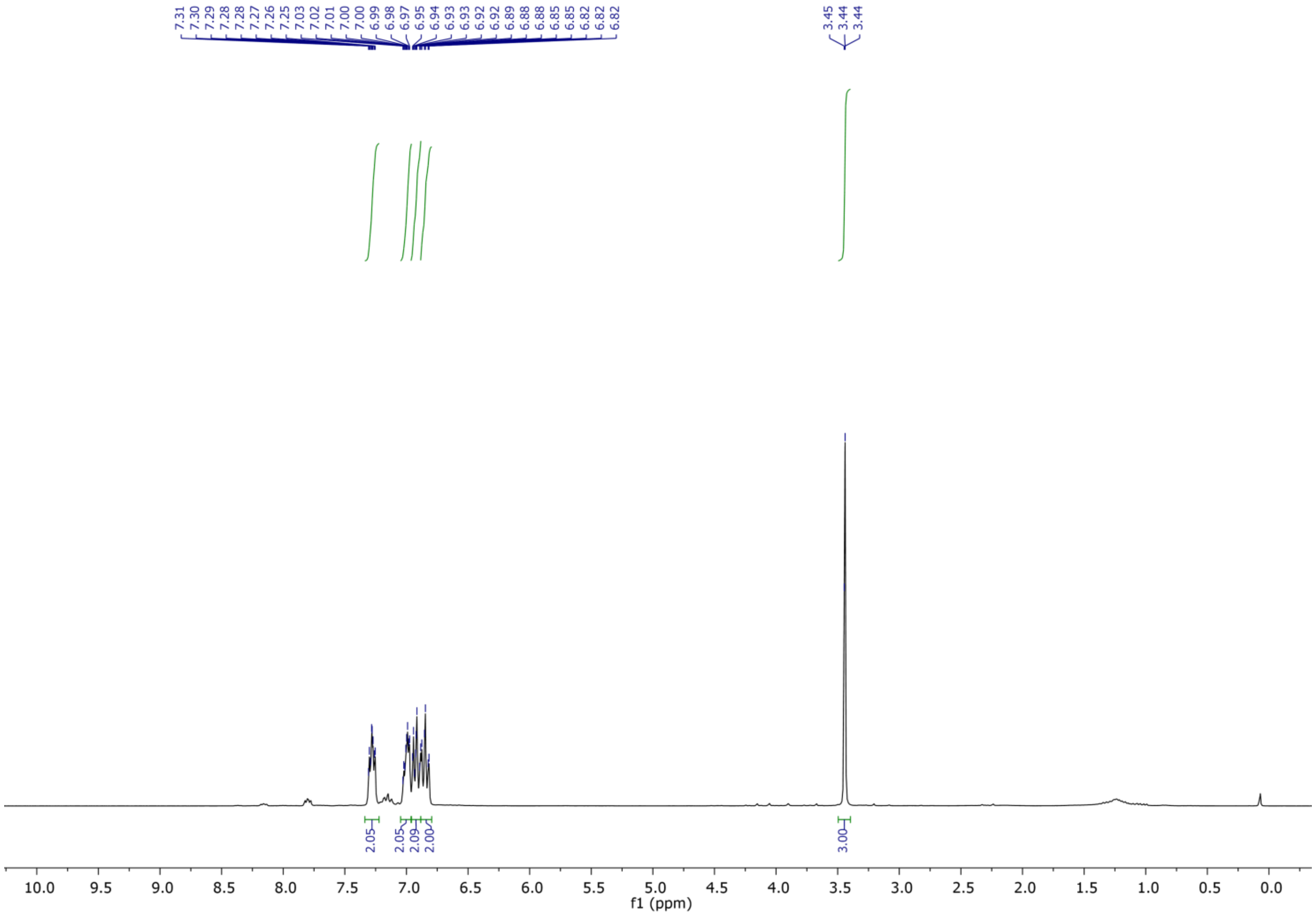

^{1}H NMR spectrum of **11p** (300 MHz, $CDCl_3$)

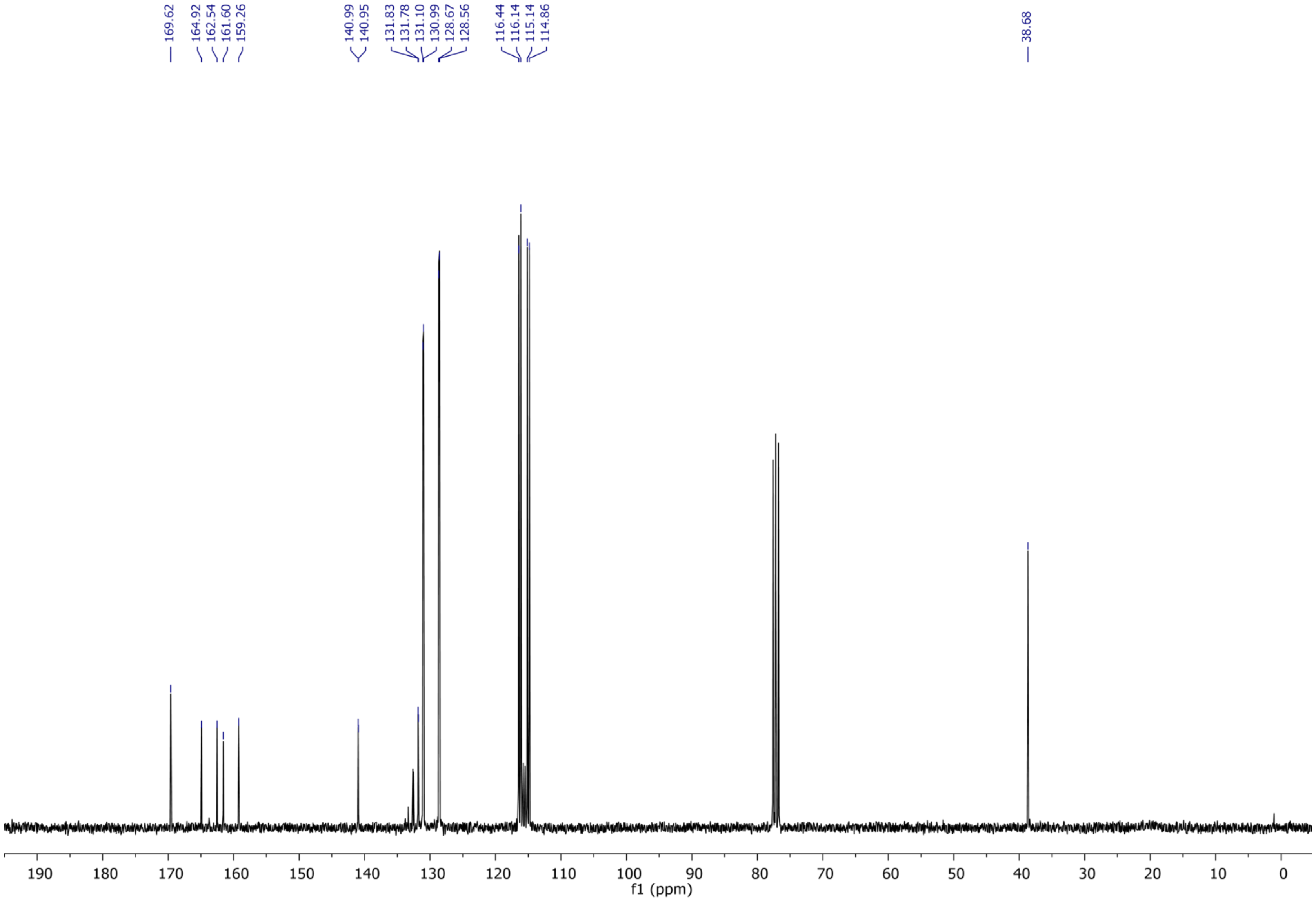

^{13}C NMR spectrum of **11p** (75 MHz, $CDCl_3$)

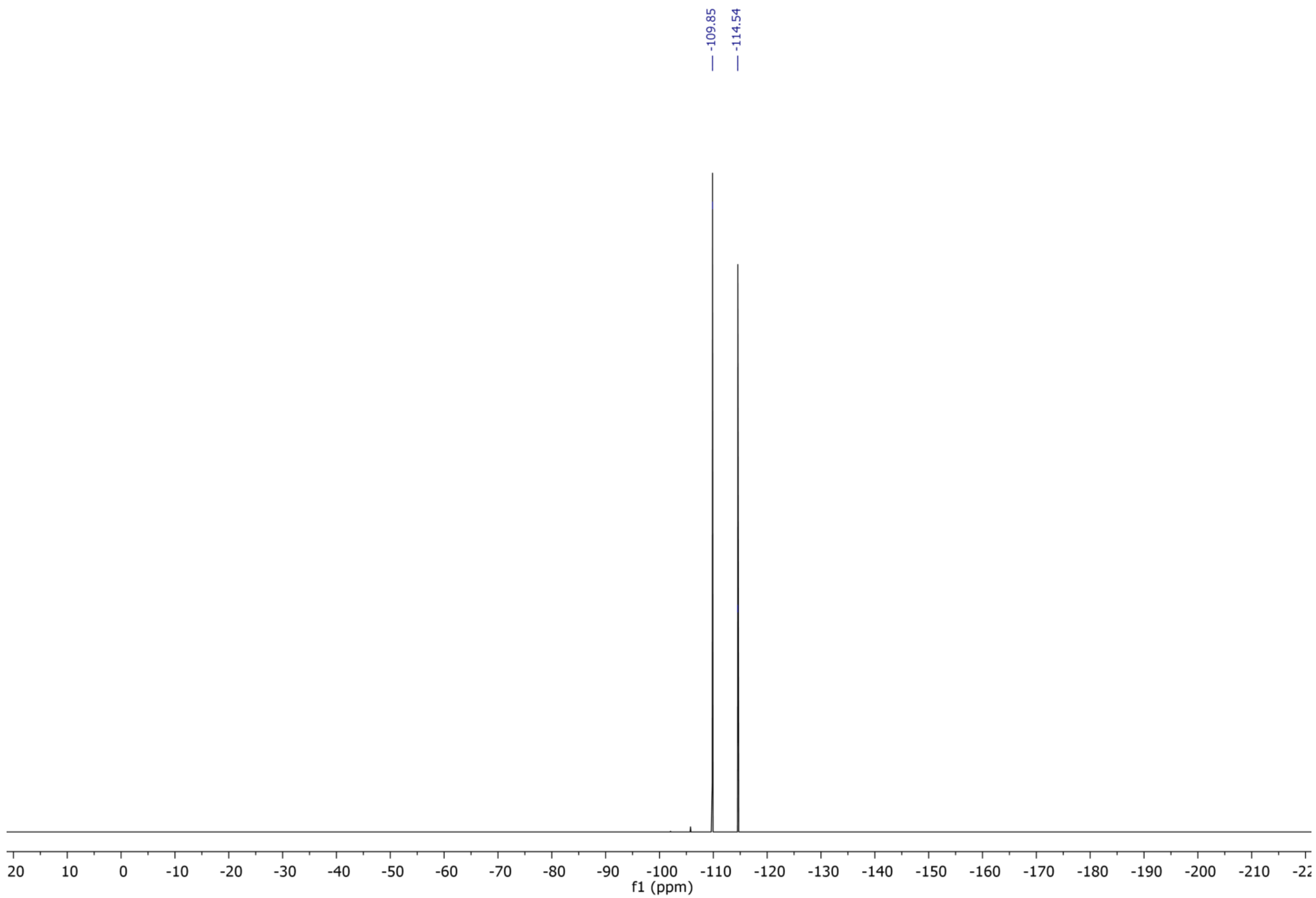

^{19}F NMR spectrum of **11p** (282 MHz, $CDCl_3$)

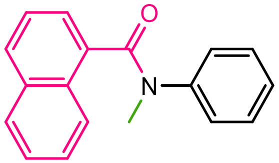

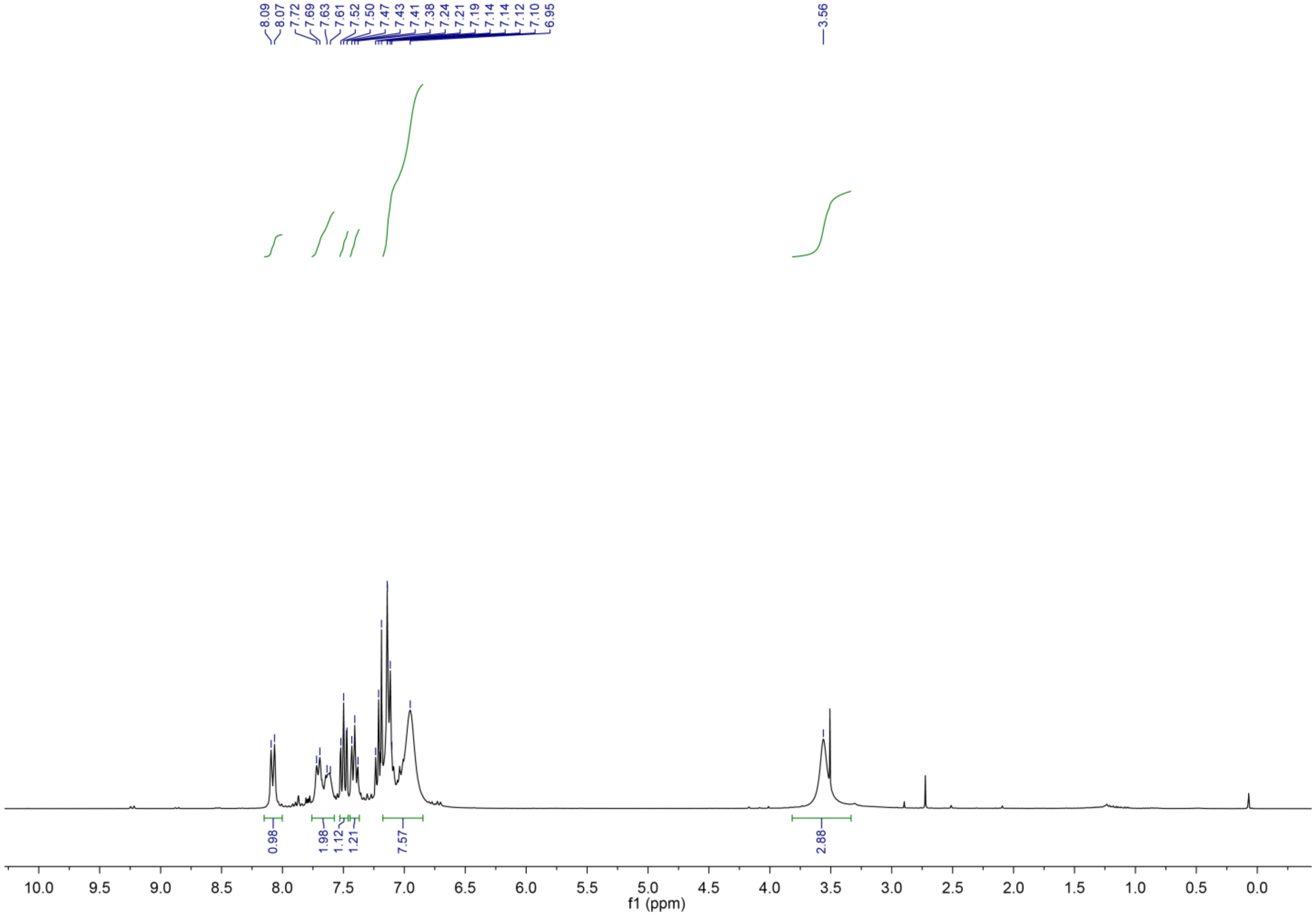

^{1}H NMR spectrum of **11q** (300 MHz, $CDCl_3$)

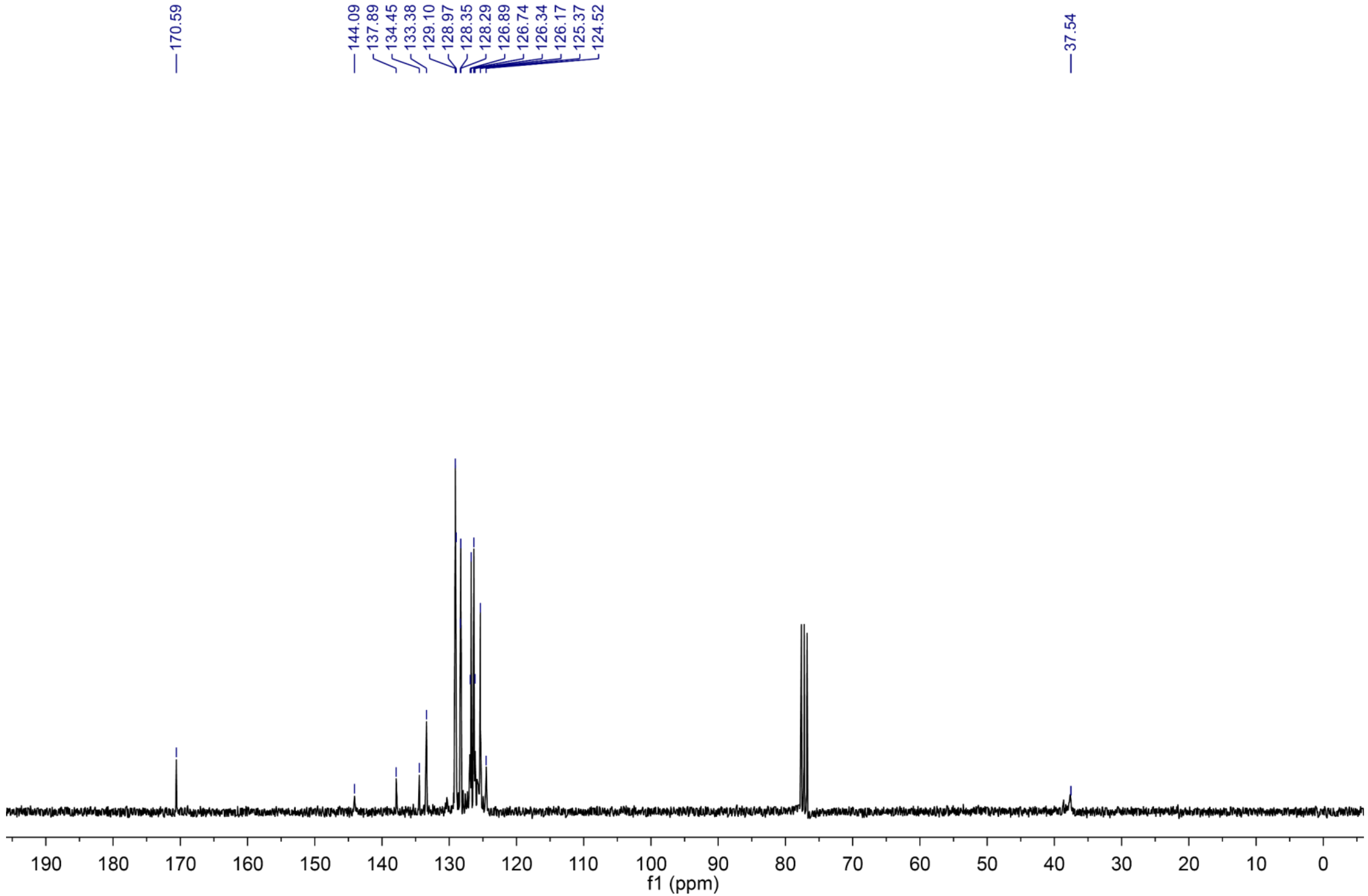

^{13}C NMR spectrum of **11q** (75 MHz, $CDCl_3$)

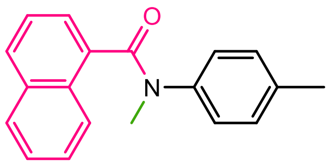

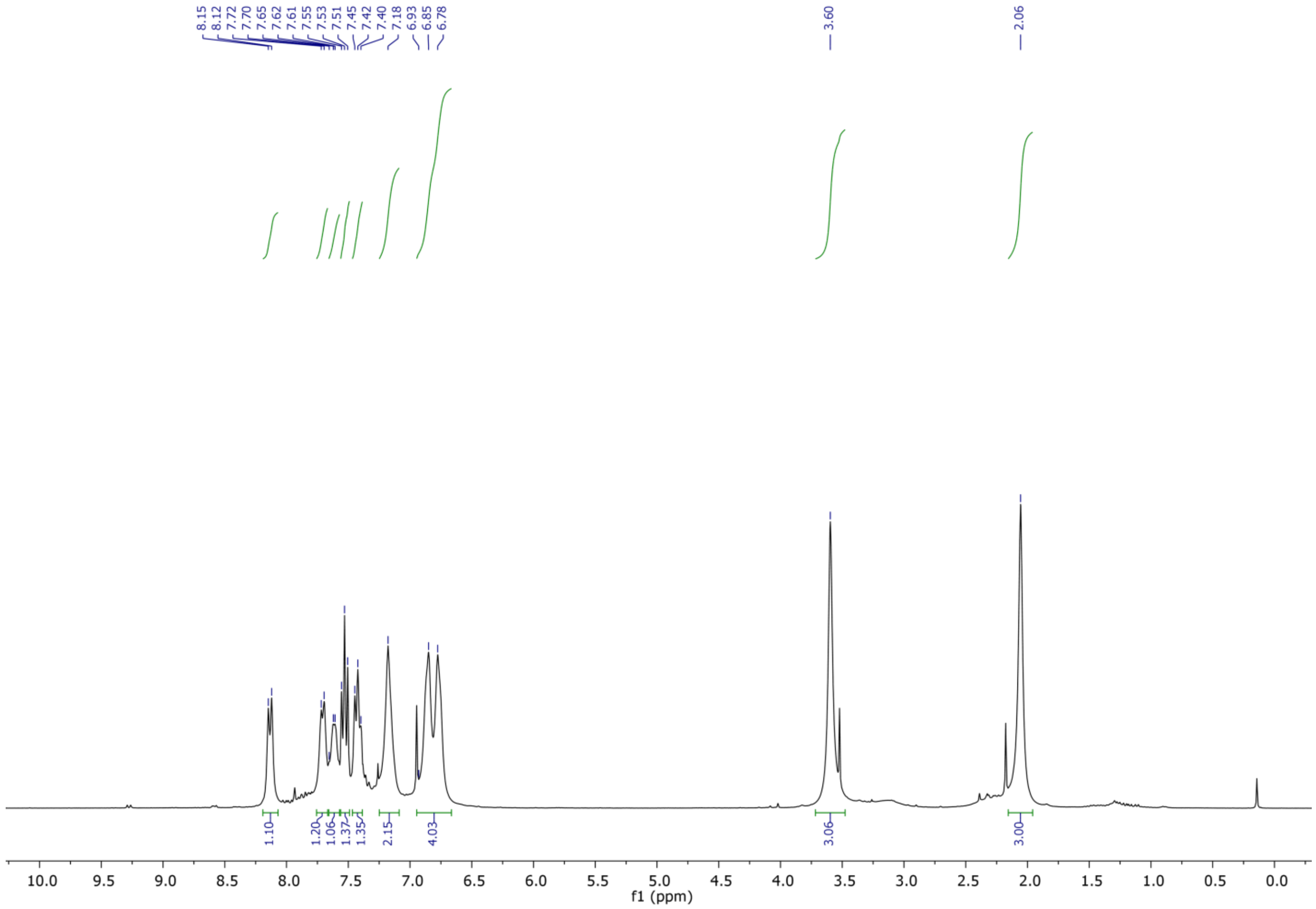

^{1}H NMR spectrum of **11r** (300 MHz, $CDCl_3$)

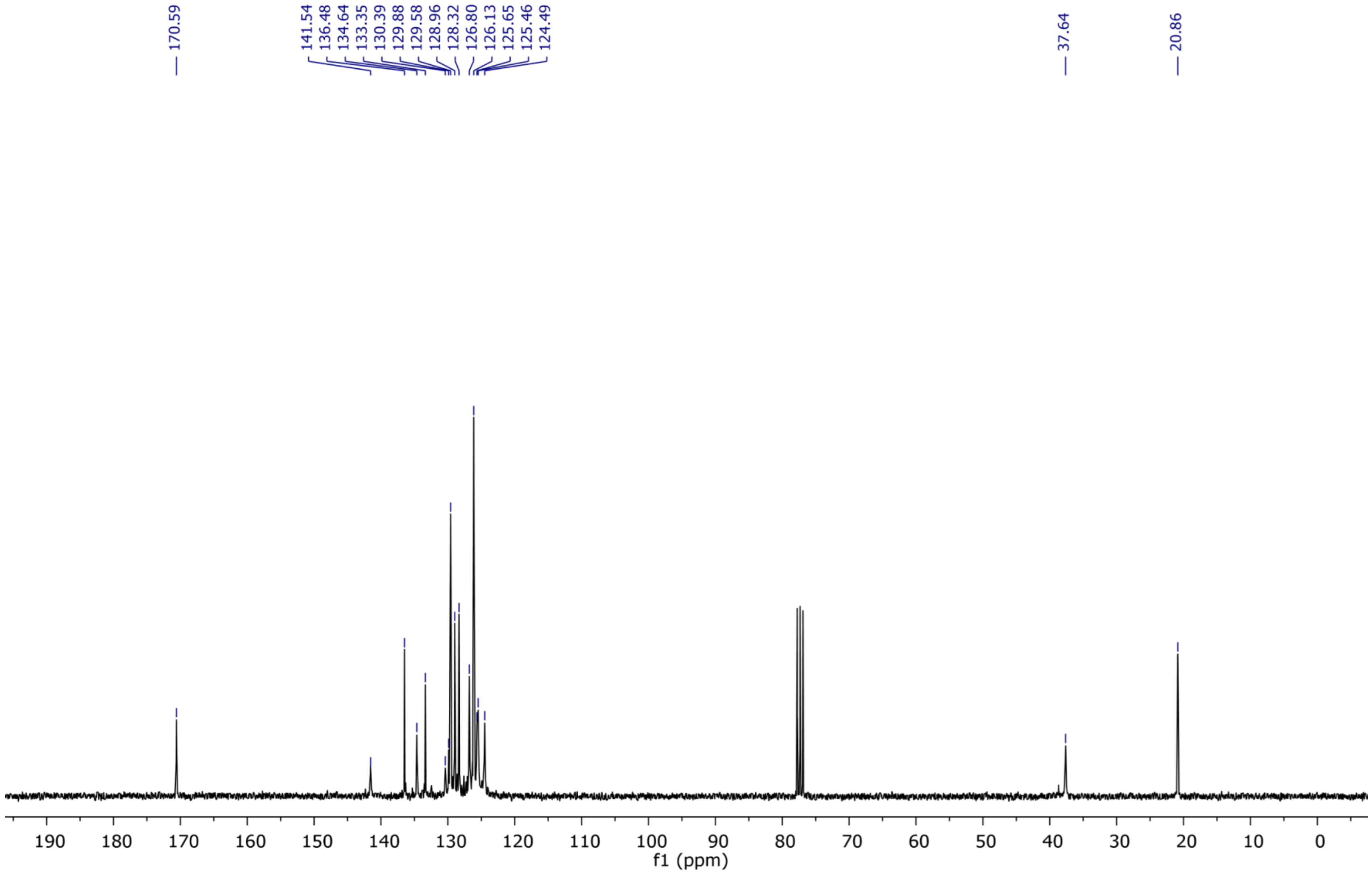

^{13}C NMR spectrum of **11r** (75 MHz, $CDCl_3$)

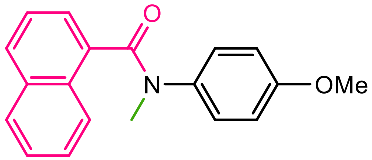

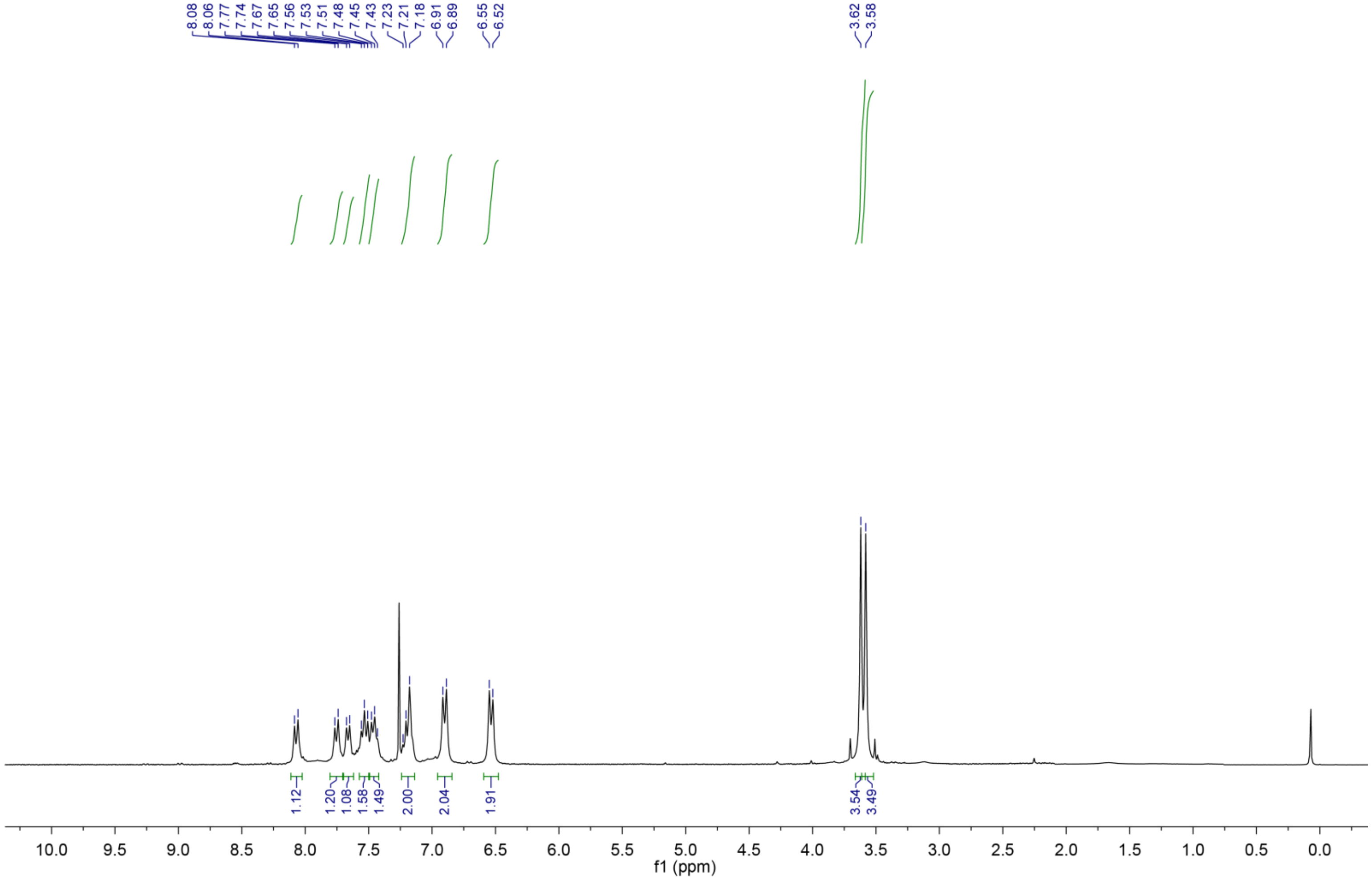

^{1}H NMR spectrum of **11s** (300 MHz, $CDCl_3$)

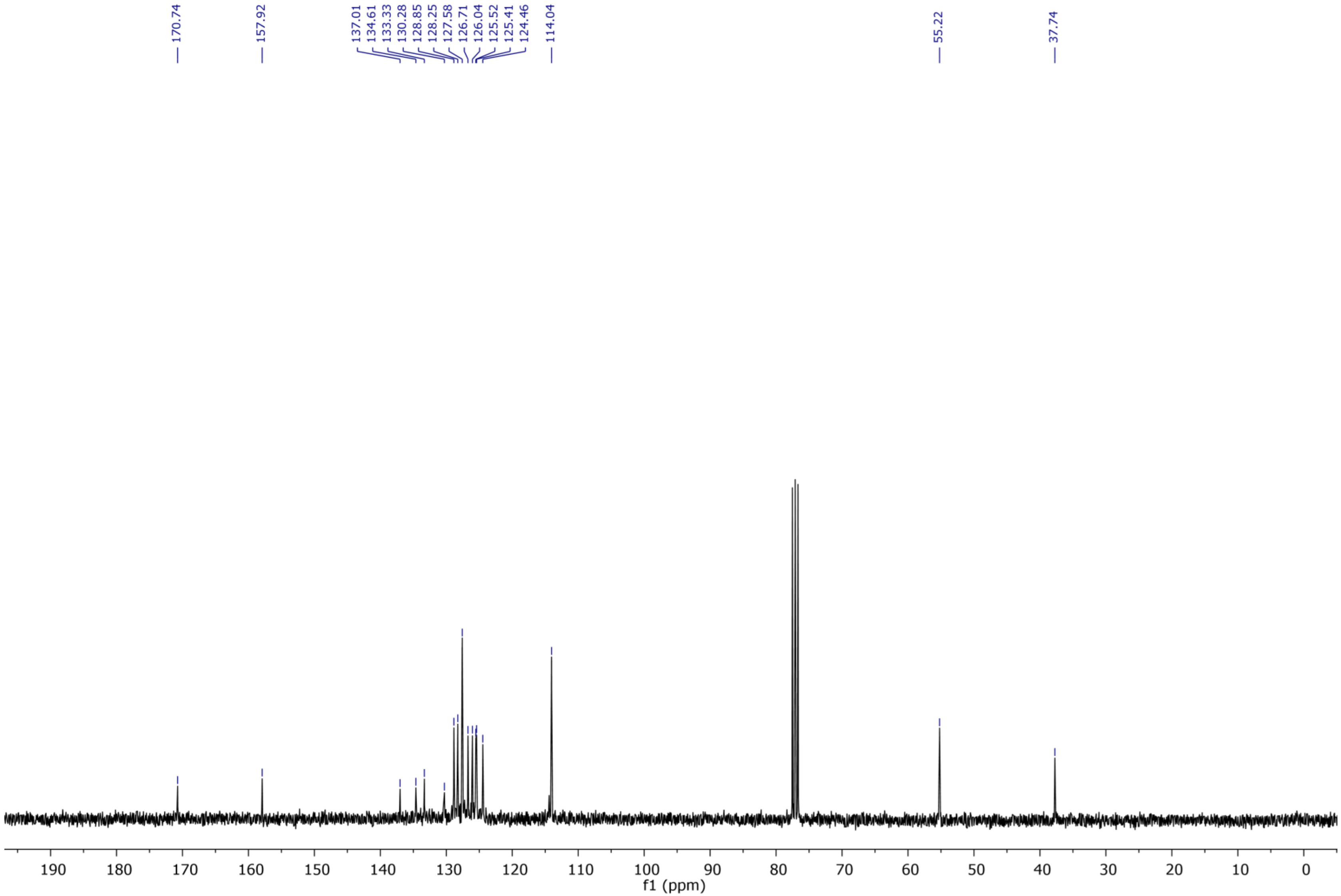

^{13}C NMR spectrum of **11s** (75 MHz, $CDCl_3$)

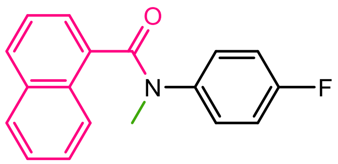

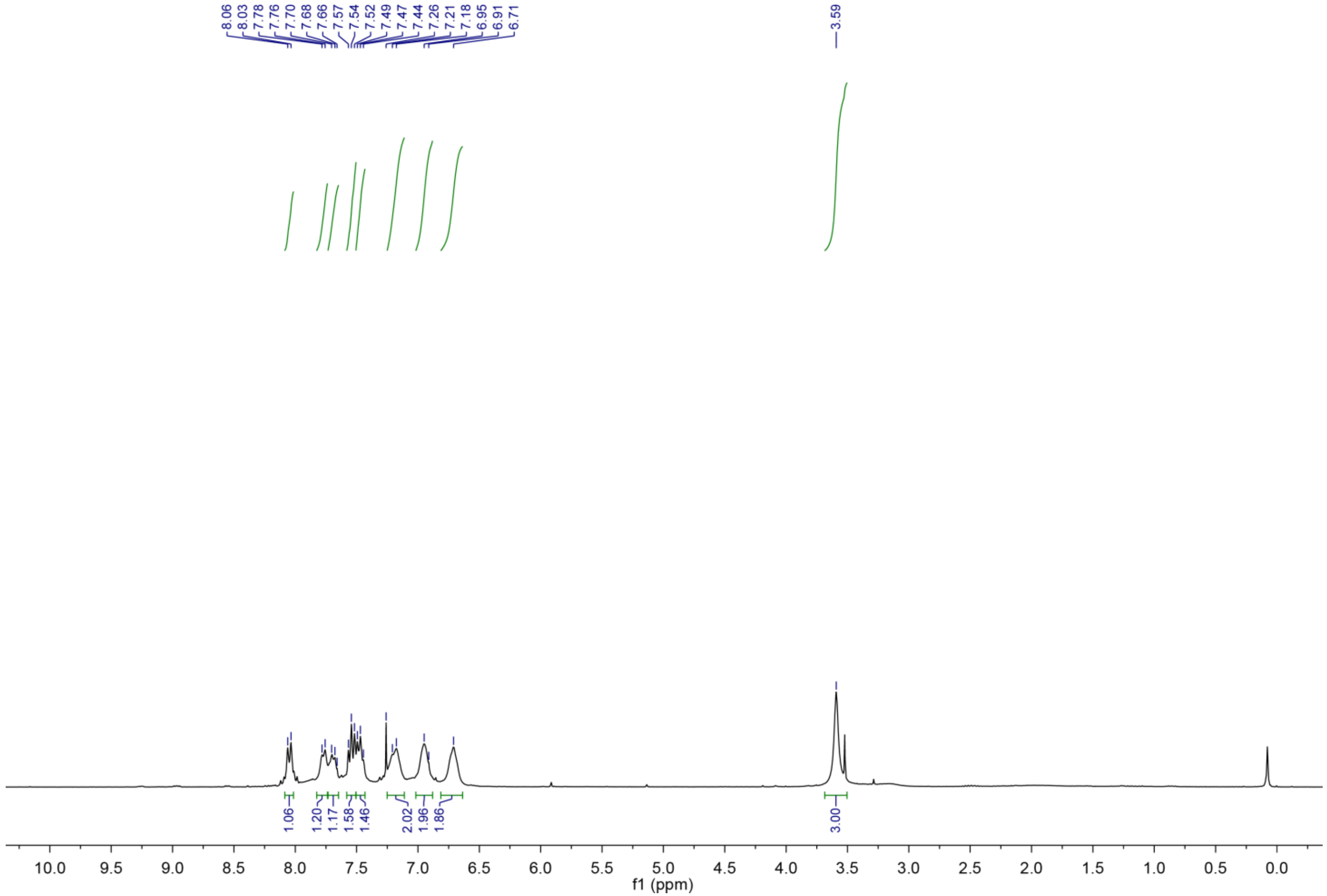

^{1}H NMR spectrum of **11t** (300 MHz, $CDCl_3$)

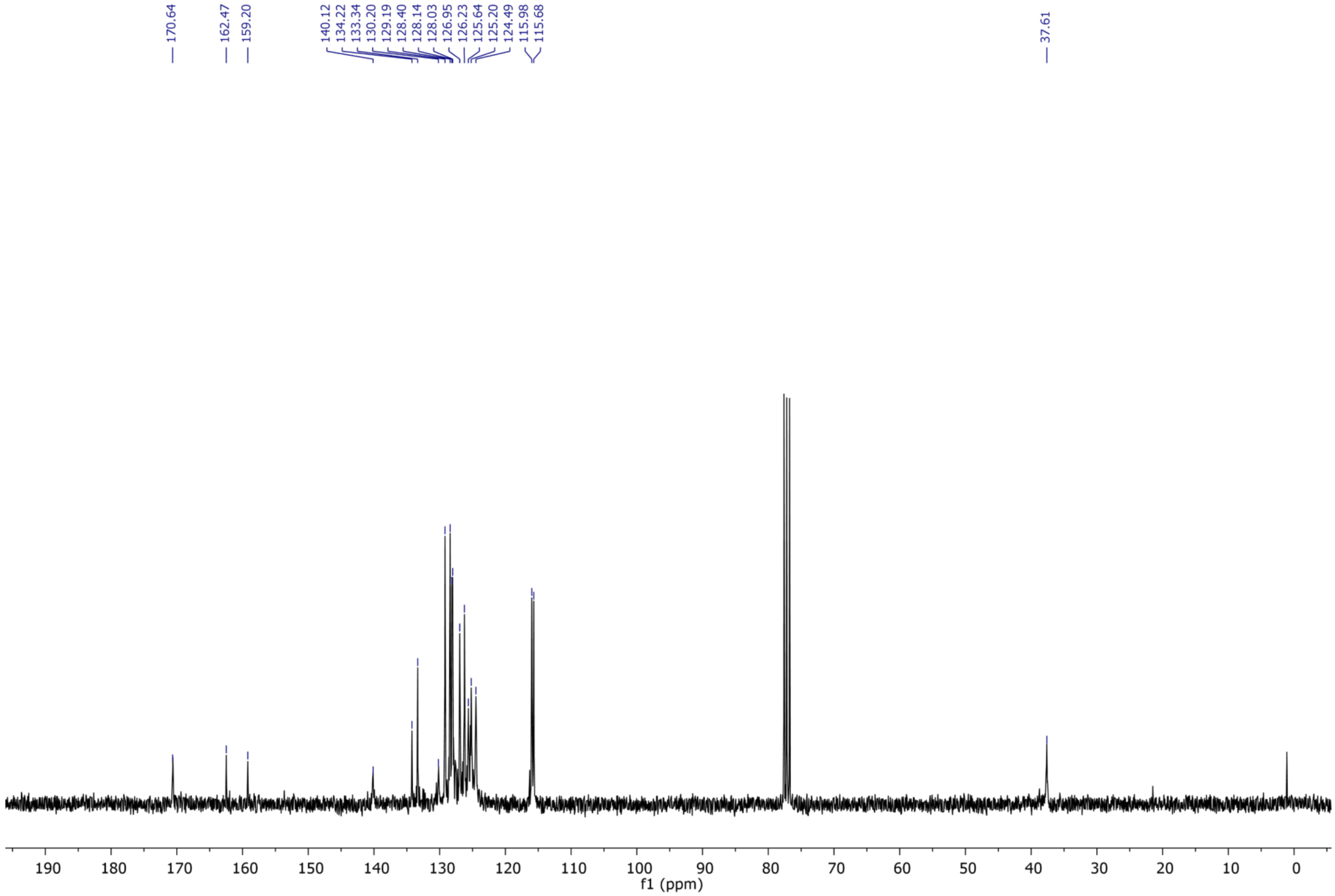

^{13}C NMR spectrum of **11t** (75 MHz, $CDCl_3$)

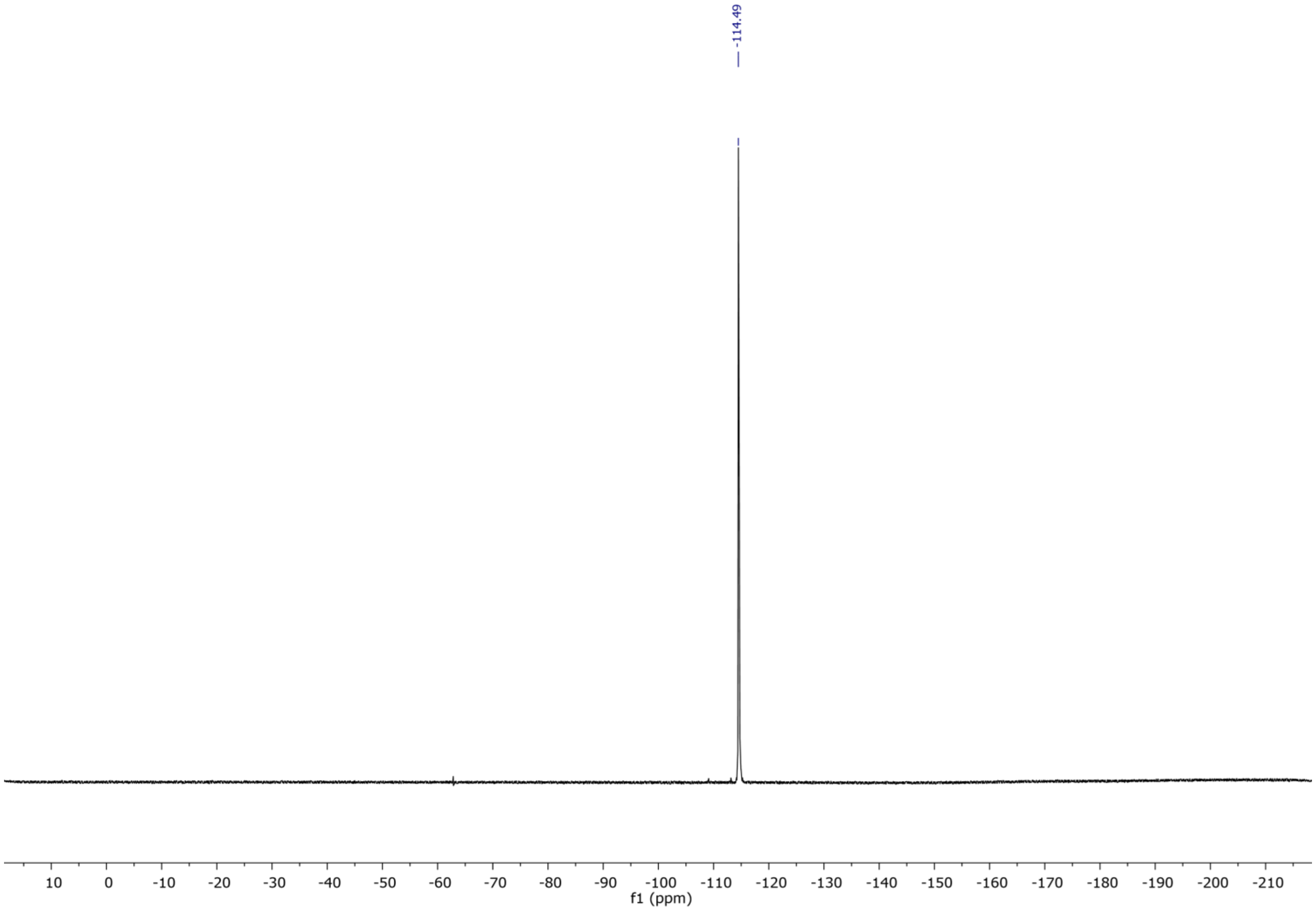

^{19}F NMR spectrum of **11t** (282 MHz, $CDCl_3$)

3.3 Copies of HRMS and IR spectra of the newly synthesized amides

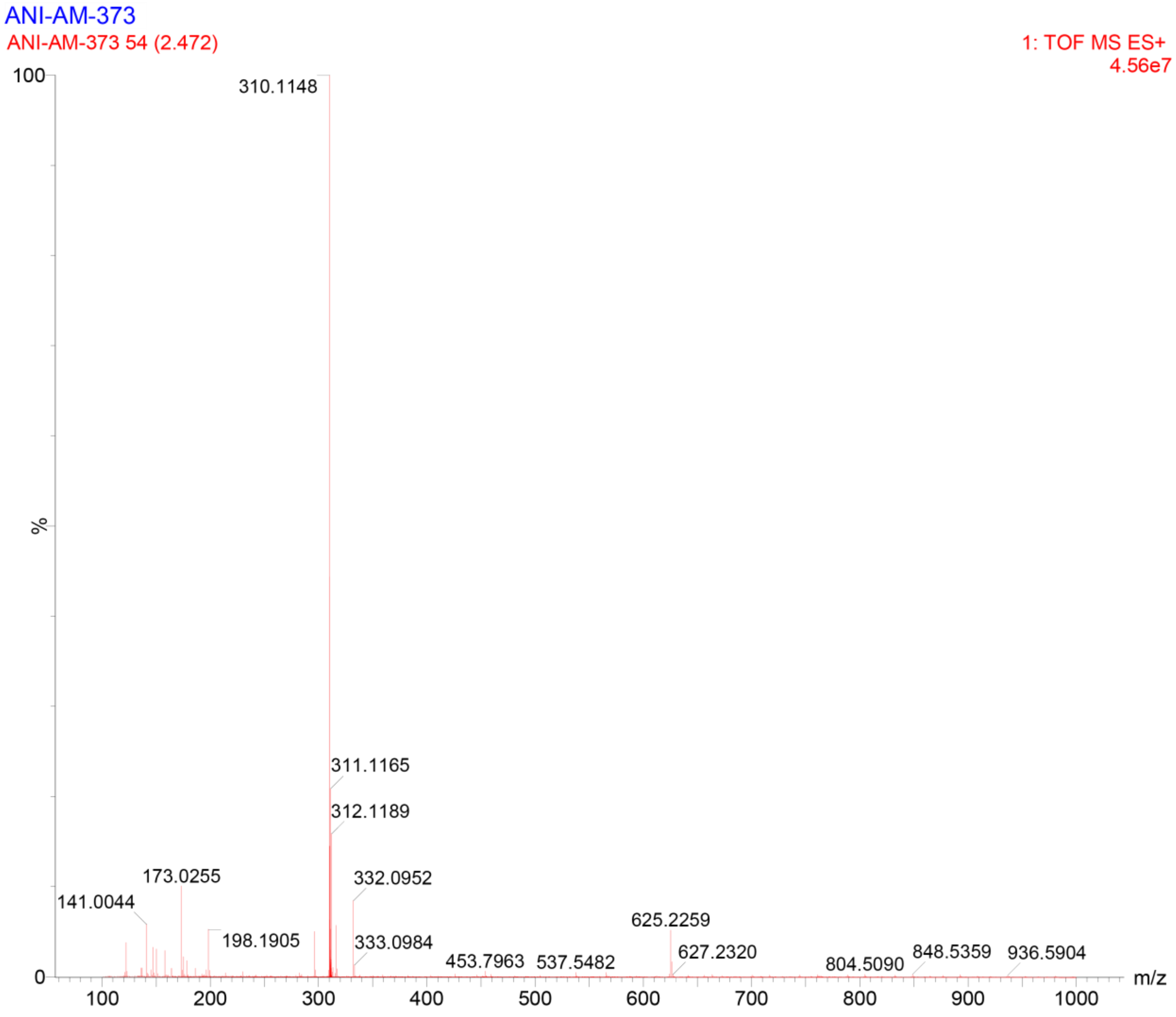

HRMS spectrum of **11l**

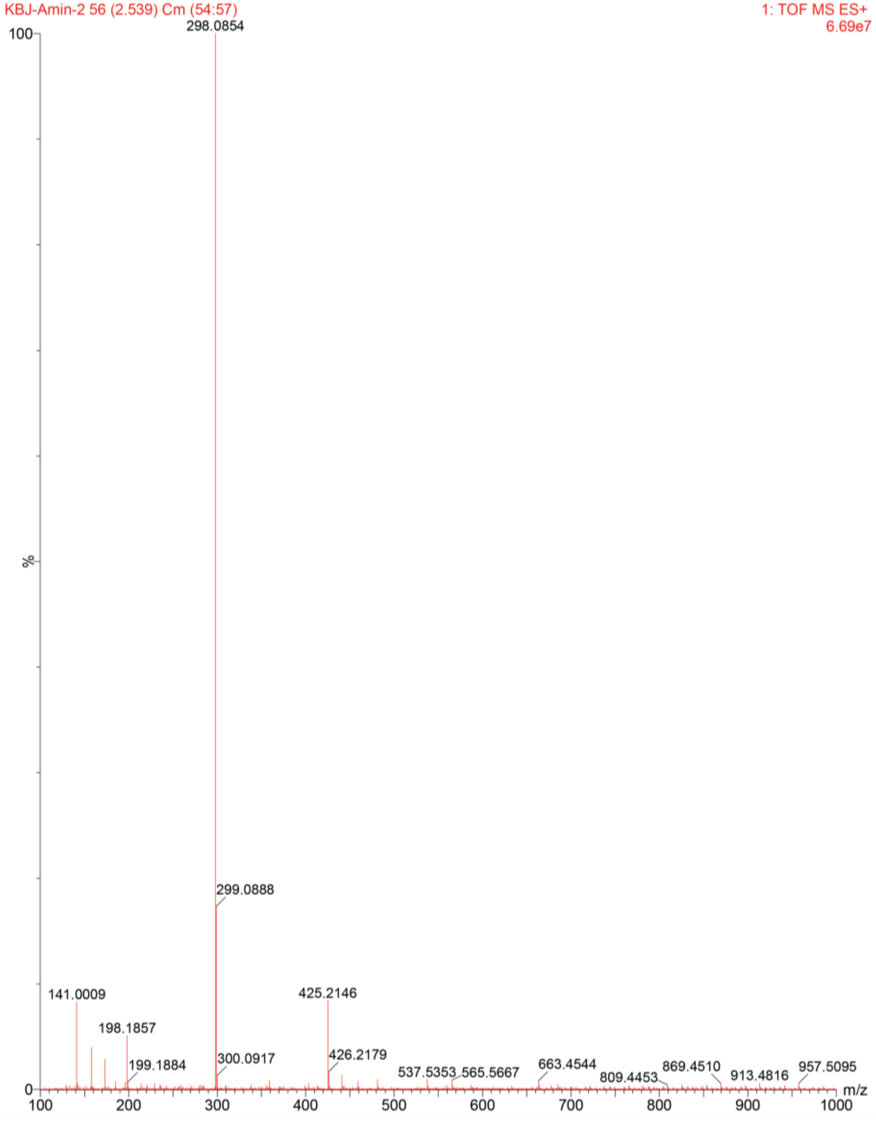

HRMS spectrum of **11m**

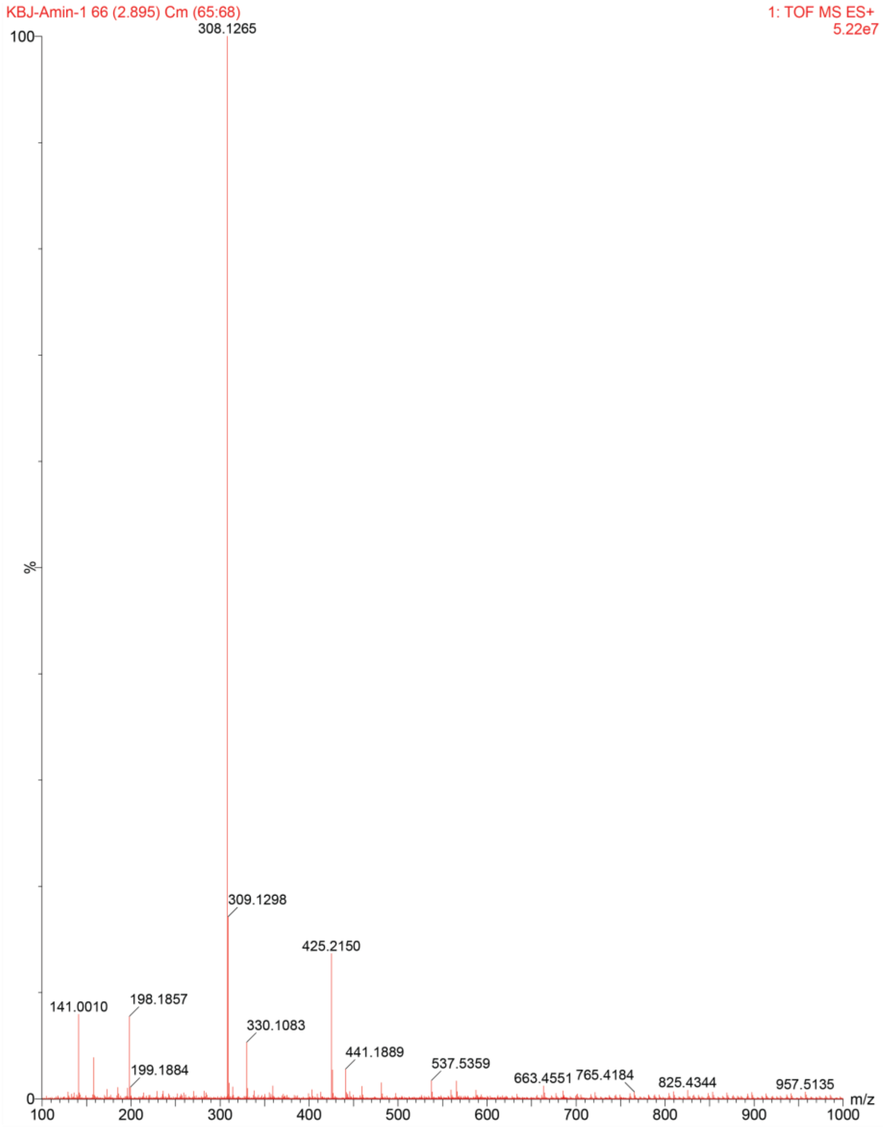

HRMS spectrum of **11n**

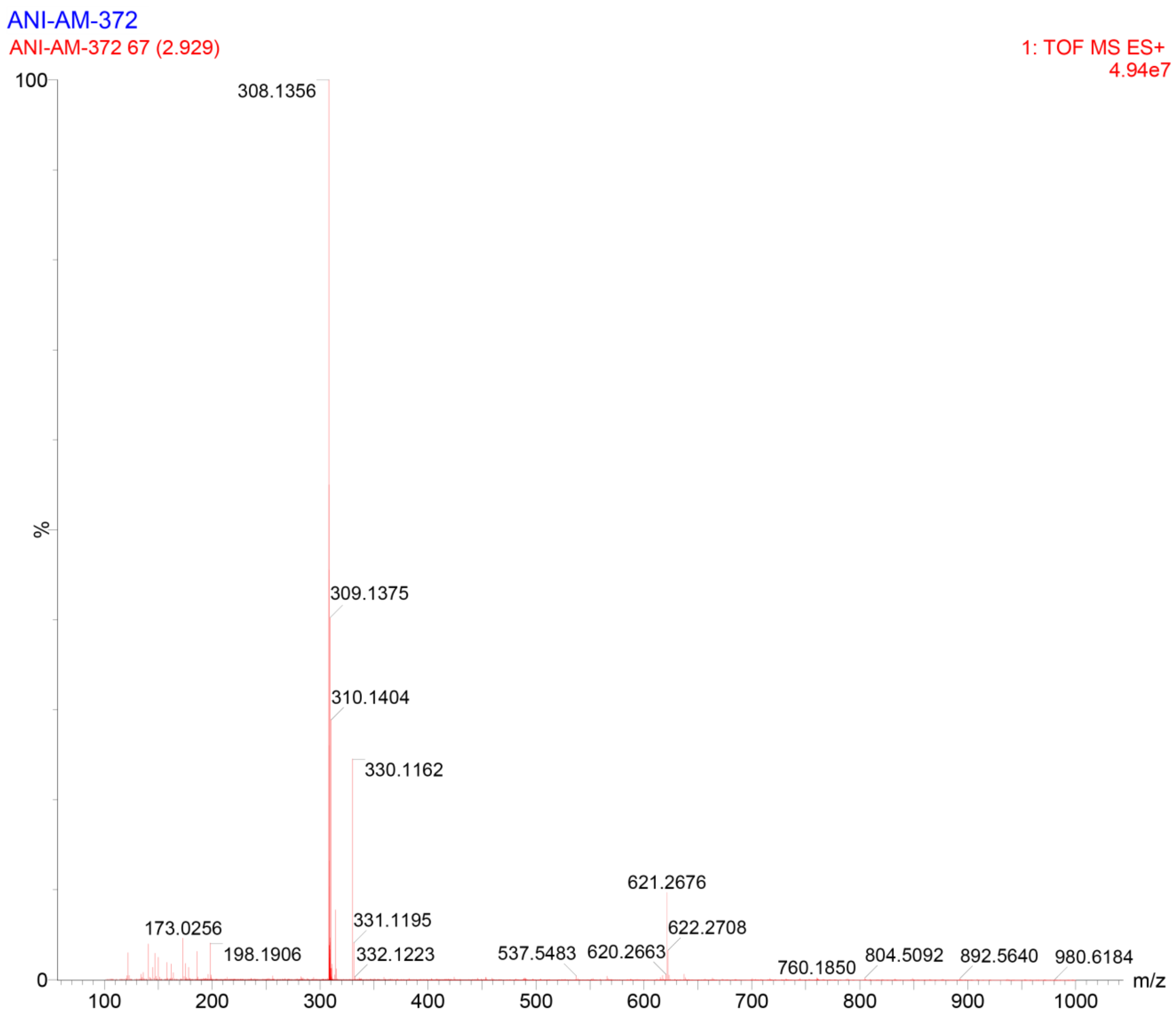

HRMS spectrum of **11o**

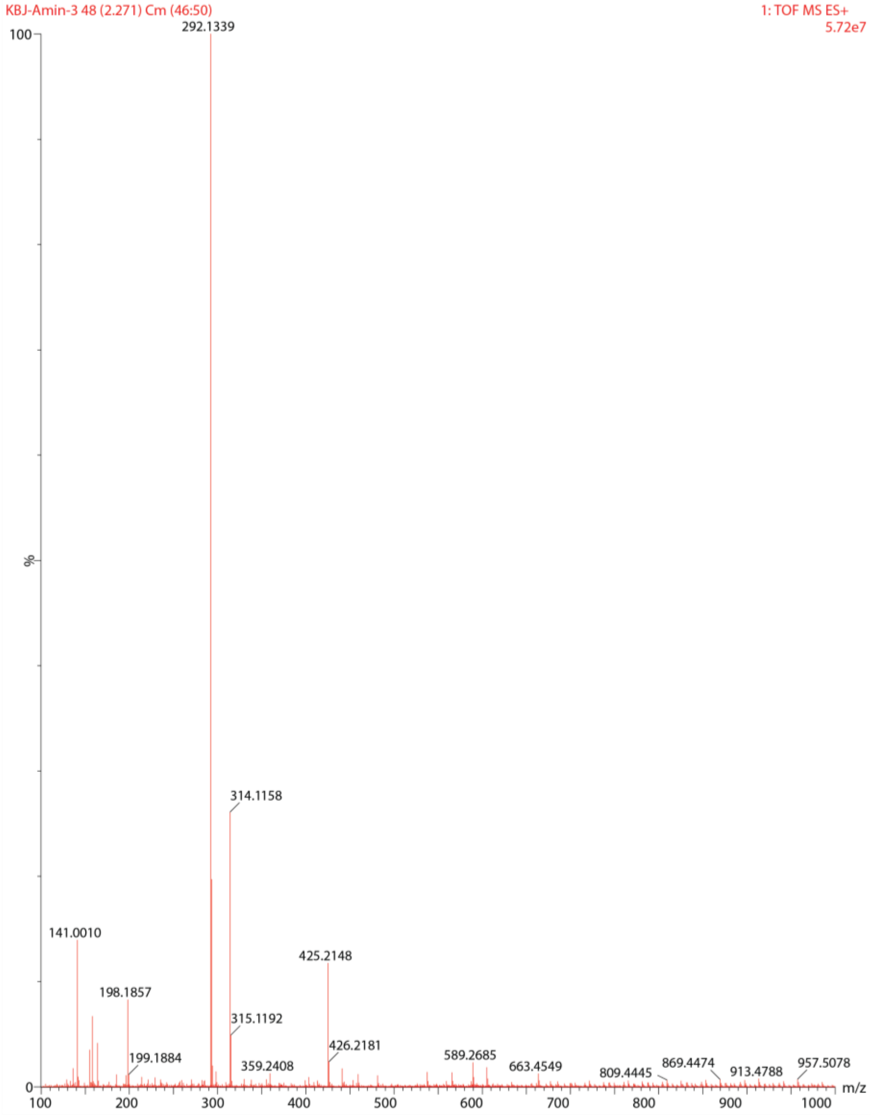

HRMS spectrum of **11s**

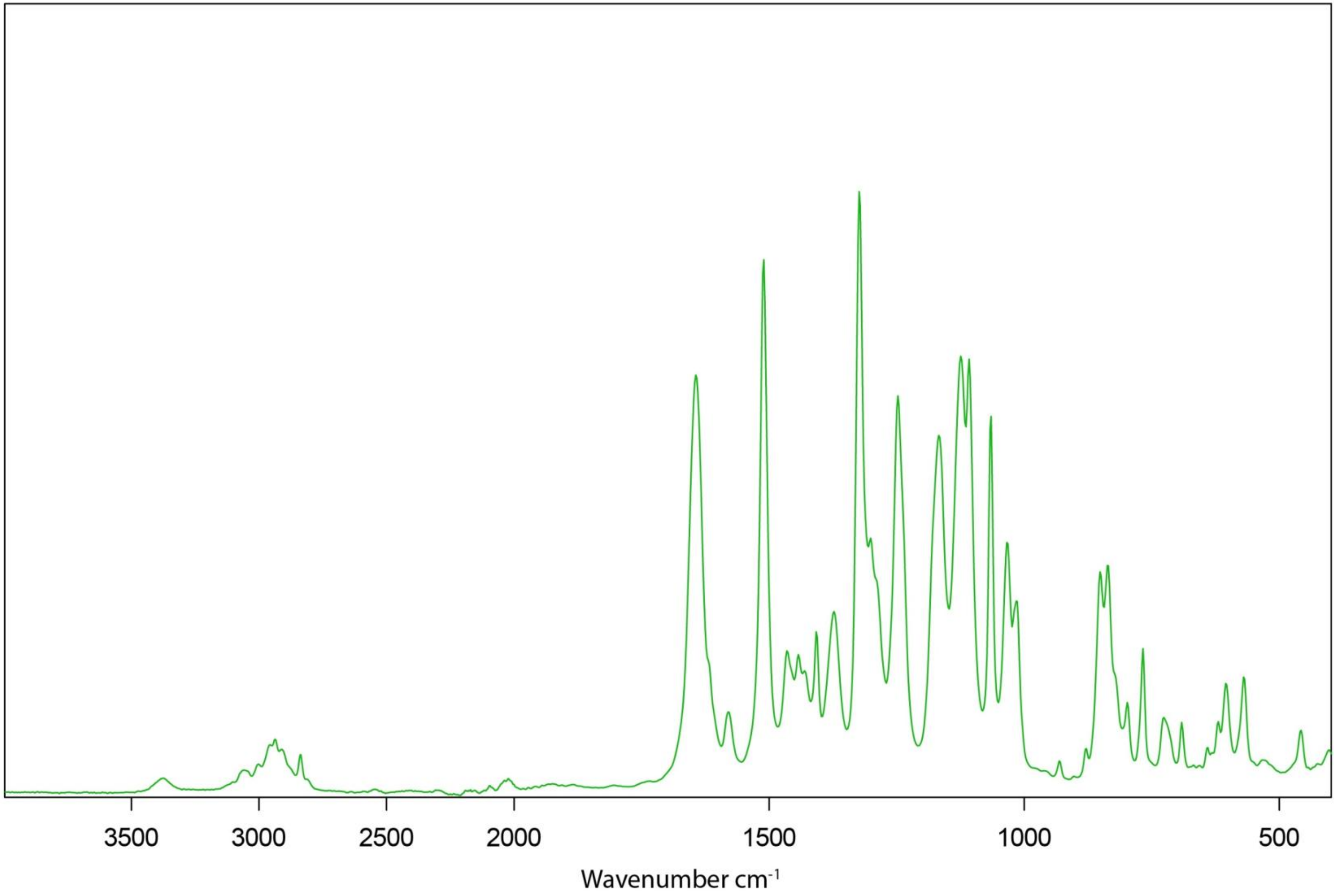

IR spectrum of **11l**

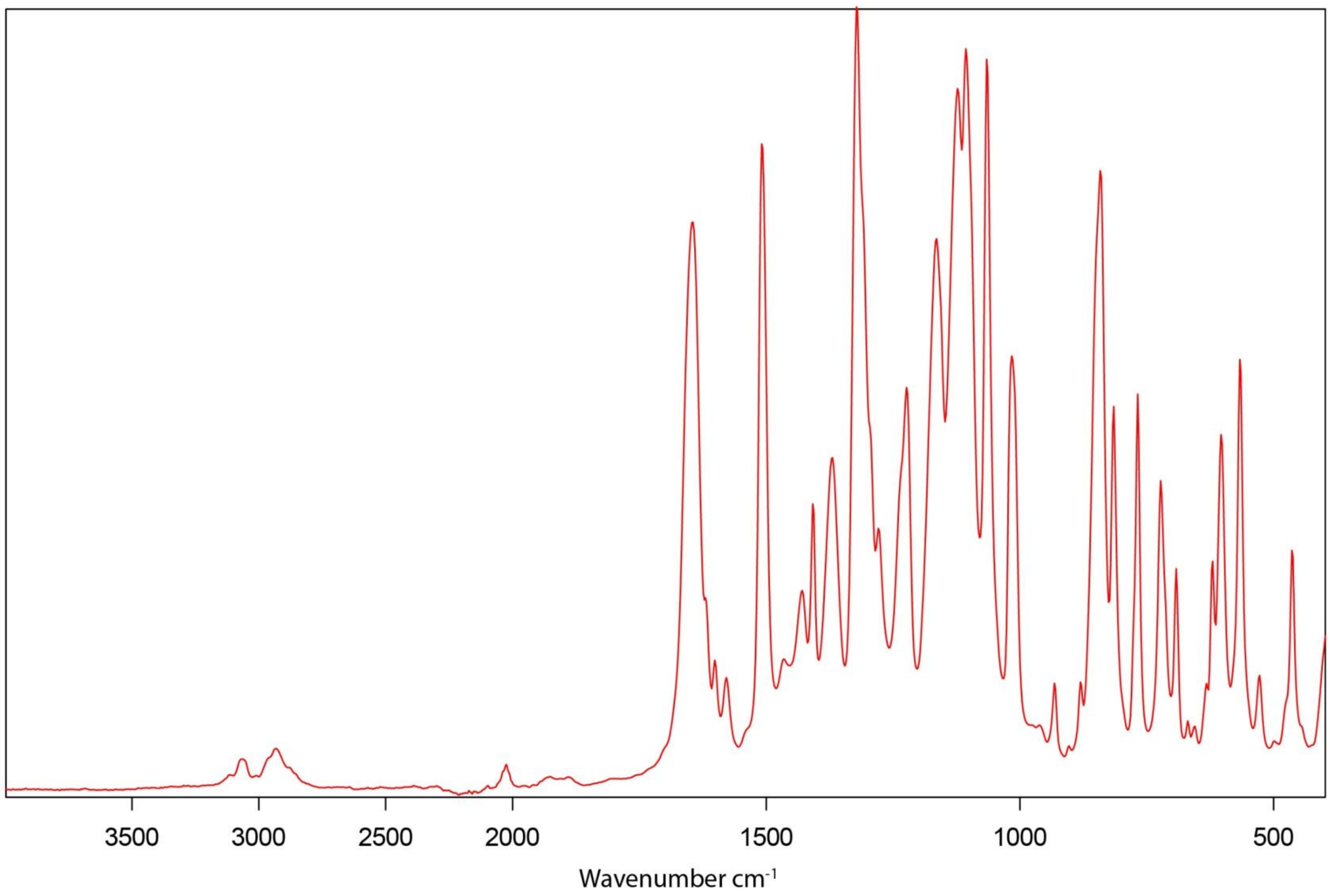

IR spectrum of **11m**

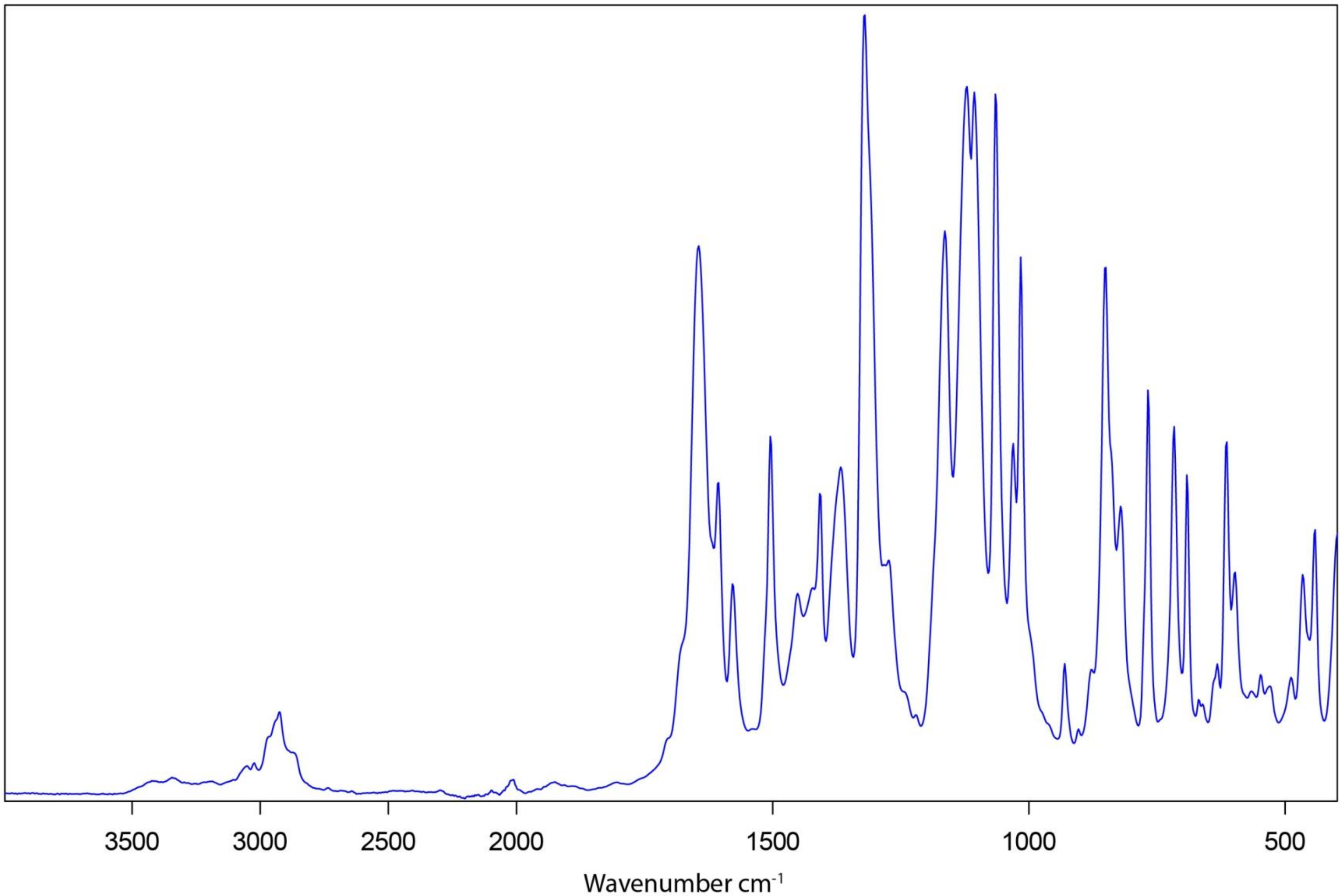

IR spectrum of **11n**

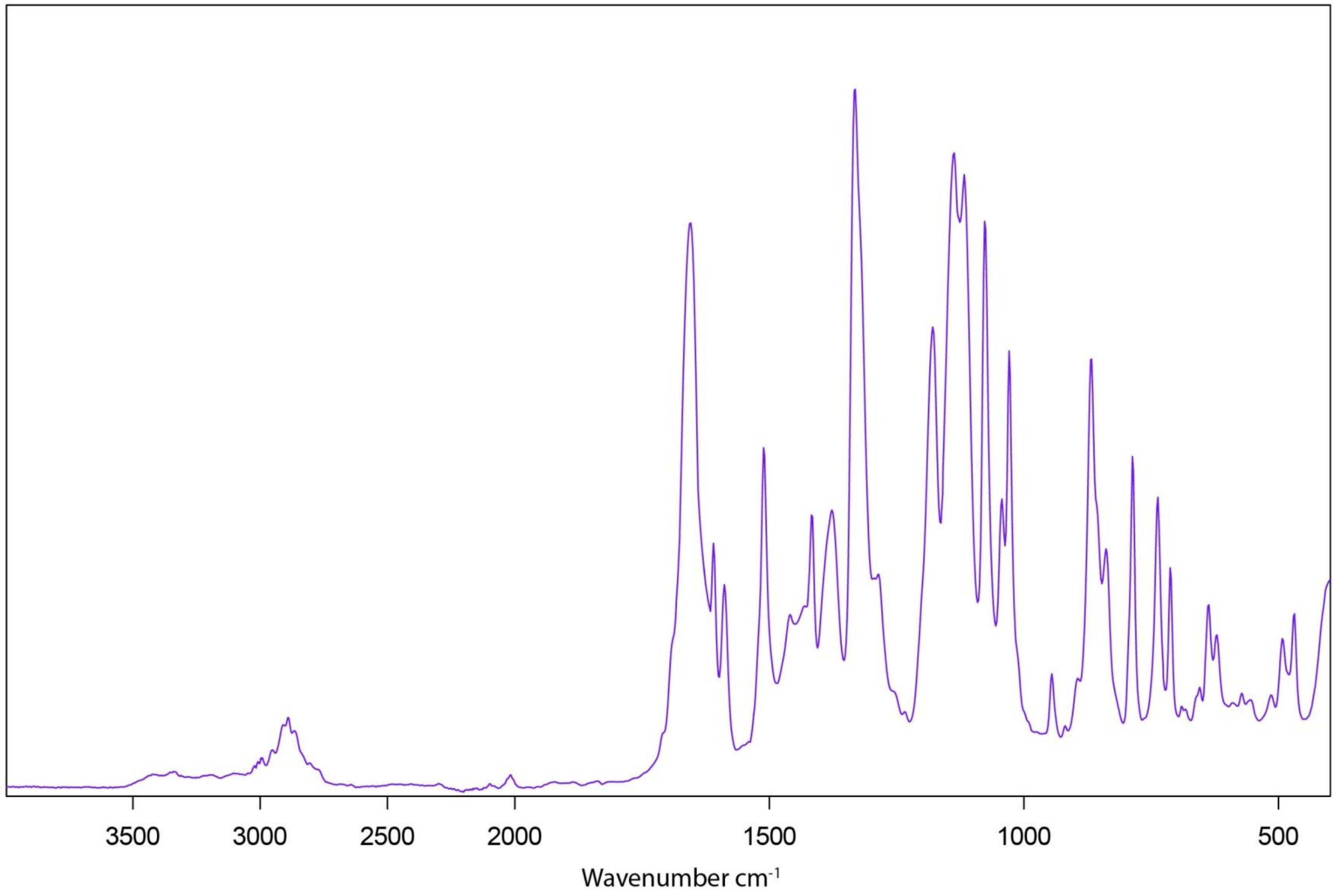

IR spectrum of **11o**

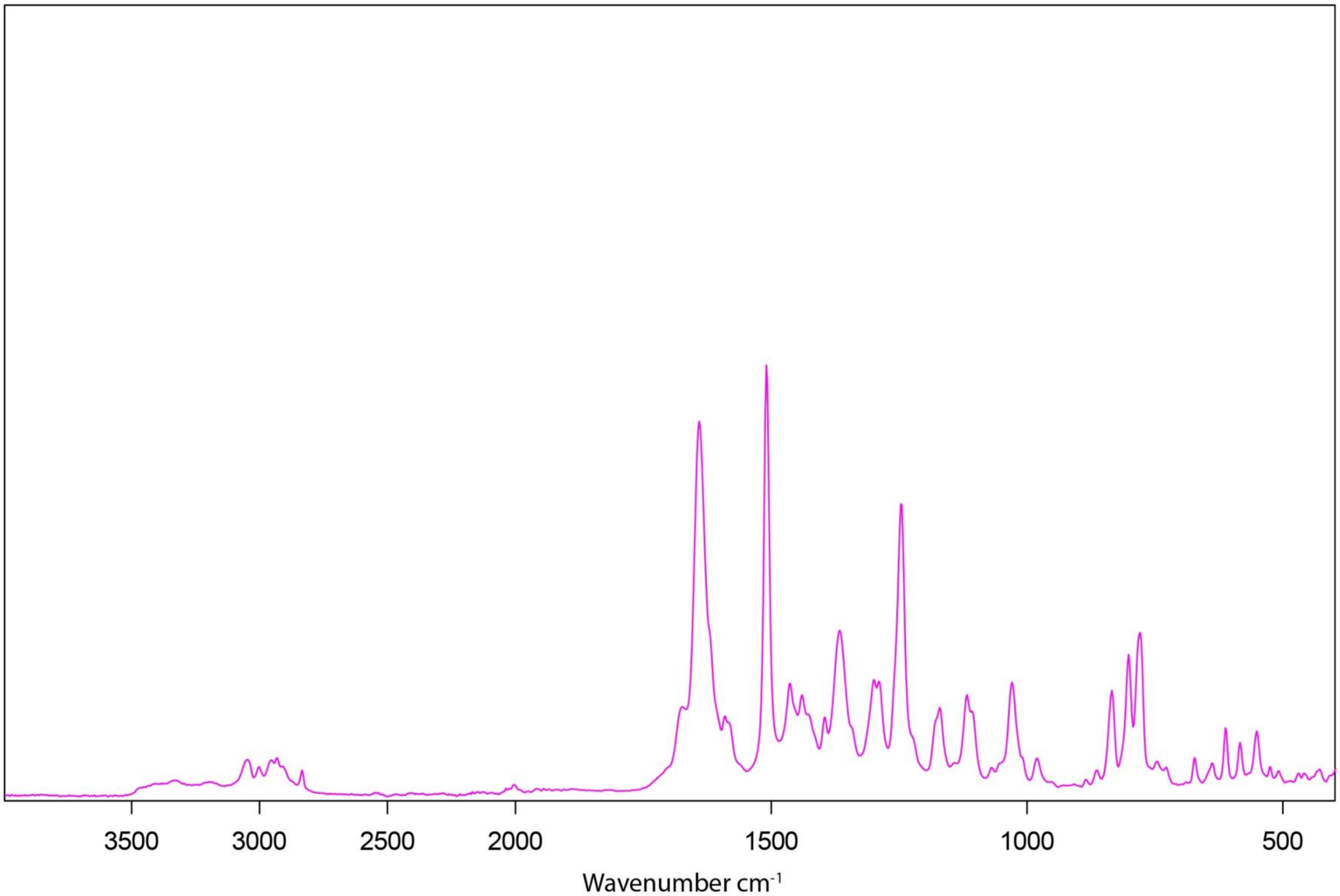

IR spectrum of **11s**

Crystallographic data for the complexes **2**, **4** · 2 DMSO and **3H₂**

Table S1. Crystallographic data for the complexes **2, 4** · 2 DMSO **and 3H₂**

compound	**2**	**4 · 2 DMSO**	**$3H_2$**
Empirical formula	$C_{21}H_{38}ClIrN_4OP_2$	$C_{23}H_{46}Cl_3IrN_4O_3P_2PdS_2$	$C_{35}H_{30}ClIrN_2OP_2$
Formula weight	652.14	957.65	784.70
**Crystal Size (mm^3)**	0.230 x 0.078 x 0.011	0.407 x 0.145 x 0.052	0.189 x 0.61 x 0.049
Wavelength (Å)	0.71073	0.71073	0.71073
Crystal system	Monoclinic	Triclinic	Orthorhombic
Space group	$P\ 2_1/n$	$P\bar{1}$	*P*bca
a (Å)	10.870(3)	8.7278(8)	17.4799(9)
b (Å)	8.037(3)	10.1403(8)	16.3925(9)
c (Å)	14.993(4)	20.1959(17)	21.6284(13)
**α (°)**	90	104.039(3)	90
**β (°)**	104.871(10)	92.766(3)	90
**γ (°)**	90	92.412(3)	90
Cell volume (Å^3)	1266.1(7)	1729.3(3)	6197.4(6)
Z	2	2	8
**Calculated density (g/cm^3)**	1.711	1.839	1.682
**μ(Mo-K_α) [mm^{-1}]**	5.525	4.840	4.535
**θ range (°)**	3.280 – 25.064	2.340 – 28.341	1.946 - 27.967
**θ full**	25.064	25.242	25.242
hkl-indices	-12<=h<=12	-11<=h<=11	-23<=h<=22
	-9<=k<=9	-13<=k<=13	-21<=k<=21

	-17<=l<=17	-26<=l<=26	-28<=l<=28
reflections collected	55282	91184	335234
independent reflections	2230	8621	7451
**R_{int}**	0.0273	0.0407	0.1417
Goodness-of-fit on F2	1.101	1.054	1.028
**Completeness to full *θ***	99.2%	99.8%	99.9%
Absorption correction	‘multi-scan’	‘multi-scan’	‘multi-scan’
Min./ max. transmission	0.3447 / 0.4899	0.2116 / 0.4920	0. 3972 / 0.4917
data / restraints / parameters	2230 / 0 / 156	8621 / 2 / 405	7451 / 0 / 389
R1, wR2on all data	0.0131, 0.0310	0.0172, 0.0412	0.0510, 0.0756
R1, wR2with Io> 2s(Io)	0.0125, 0.0307	0.0165, 0.0408	0.0316, 0.0657
**max diff peak, hole [eÅ^{-3}]**	0.621, -0.404	0.793, -0.911	1.101, -1.148
CCDC	2430139	2430141	2430140

5. References

[1] F. A. Cotton, P. Lahuerta, M. Sanau, W. Schwotzer, *Inorg. Chim. Acta.* **1986**, *120*, 153.

[2] D. B. Grotjahn, J. E. Kraus, H. Amouri, M.-N. Rager, A. L. Cooksy, A. J. Arita, S. A. Cortes-Llamas, A. A. Mallari, A. G. DiPasquale, C. E. Moore, L. M. Liable-Sands, J. D. Golen, L. N. Zakharov, A. L. Rheingold, *J. Am. Chem. Soc*. **2010**, *132*, 7919.

[3] K. Kretschmar, V. Pelmenschikov, M. Kaupp, T. Braun, P. Wittwer, S. Rachor, J. Cardozo, Eur. *J. Inorg. Chem*. **2023**, *26*, e202300099.

[4] a) J.J. de Pater, C.E.P. Maljaars, E. de Wolf, M. Lutz, A.L. Spek, B.J. Deelman, C.J. Elsevier, G. van Koten, *Organometallics*. **2005**, *24*, 5299; b) J. Liu, C. Jacob, K. J. Sheridan, F. Al-Mosule, B. T. Heaton, J. A. Iggo, M. Matthews, J. Pelletier, R. Whyman, J. F. Bickley, A. Steiner, *Dalton Trans*. **2010**, *39*, 7921.

[5] G. Franciò, R. Scopelliti, C. G. Arena, G. Bruno, D. Drommi, F. Faraone, *Organometallics*. **1998**, *17*, 338.

[6] G. M. Sheldrick, SHELXS-96, Program for the Solution of Crystal Structures, University of Göttingen, Göttingen (Germany), **1996**.

[7] G. Sheldrick, *Acta Crystallogr. Sect. A.* **2008**, *64*, 112.

[8] G. Sheldrick, *Acta Crystallogr. Sect. C*. **2015**, *71*, 3.

[9] (a) Y.Q. Qi, S. Liu, Y. Xu, Y. Li, T. Su, H.L. Ni, Y. Gao, W. Yu, P. Cao, P. Hu, K.Q. Zhao, *Org. Lett*. **2022**, *24*, 5023; (b) Y. Ben-Tal, G.C. Lloyd-Jones, *J. Am. Chem. Soc*. **2022**, *144*, 15372; (c) M. Presset, D. Oehlrich, F.Rombouts, G.A. Molander, *J. Org. Chem.* **2013**, *78*, 12837; (d) G. Shi, C. Shao, S. Pan, J. Yu, Y. Zhang, *Org. Lett*. **2015**, *17*, 38.

[10] P. Pracht, F. Bohle, S. Grimme, *Phys. Chem. Chem. Phys*. **2020**, *22*, 7169.

[11] S. Grimme, J. Chem. *Theory Comput*. **2019**, *15*, 2847.

[12] F. Neese, *WIREs Comput Mol Sci*. **2011**, *2*, 73.

[13] F. Neese, *WIREs Comput Mol Sci*. **2022**, *12*.

[14] V. N. Staroverov, G. E. Scuseria, J. Tao, J. P. Perdew, *J. Chem. Phys.* **2003**, *119*, 12129.

[15] F. Weigend, R. Ahlrichs, *Phys. Chem. Chem. Phys*. **2005**, *7*, 3297.

[16] J. D. Rolfes, F. Neese, D. A. Pantazis, *J Comput Chem*. **2020**, *41*, 1842.

[17] D. A. Pantazis, F. Neese, J. Chem. *Theory Comput*. **2009**, *5*, 2229.

[18] E. Caldeweyher, C. Bannwarth, S. Grimme, *J. Chem. Phys.* **2017**, *147*.

[19] E. Caldeweyher, S. Ehlert, A. Hansen, H. Neugebauer, S. Spicher, C. Bannwarth, S. Grimme, *ChemRxiv preprint* **2019**; DOI: 10.26434/chemrxiv.7430216.v2.

[20] A. C. Castro, D. Balcells, M. Repisky, T. Helgaker, M. Cascella, *Inorg. Chem*. **2020**, *59*, 17509.

[21] W. Fang, Q. Deng, M. Xu, T. Tu, *Org. Lett*. **2013**, *15*, 3678.
